# Segmentation-based quality control of structural MRI using the CAT12 toolbox

**DOI:** 10.1093/gigascience/giaf146

**Published:** 2025-11-29

**Authors:** Robert Dahnke, Polona Kalc, Gabriel Ziegler, Julian Grosskreutz, Christian Gaser

**Affiliations:** Department of Psychiatry and Psychotherapy, Jena University Hospital, Jena 07747, Germany; Department of Neurology, Jena University Hospital, Jena 07747, Germany; German Center for Mental Health (DZPG), Jena-Halle-Magdeburg 07743, Germany; Department of Psychiatry and Psychotherapy, Jena University Hospital, Jena 07747, Germany; Department of Neurology, Jena University Hospital, Jena 07747, Germany; University Hospital Magdeburg and DZNE Magdeburg, Institute of Cognitive Neurology and Dementia Research, Magdeburg 39120, Germany; Department of Neurology, University of Lübeck, Lübeck 23538, Germany; Department of Psychiatry and Psychotherapy, Jena University Hospital, Jena 07747, Germany; Department of Neurology, Jena University Hospital, Jena 07747, Germany; German Center for Mental Health (DZPG), Jena-Halle-Magdeburg 07743, Germany

**Keywords:** MRI, brain, quality control, quality assessment, segmentation, motion artifacts

## Abstract

**Background:**

The processing and analysis of magnetic resonance images is highly dependent on the quality of the input data, and systematic differences in quality can consequently lead to loss of sensitivity or biased results. However, varying image properties due to different scanners and acquisition protocols, as well as subject-specific image interferences, such as motion artifacts, can be incorporated in the analysis. A reliable assessment of image quality is therefore essential to identify critical outliers that may bias results.

**Findings:**

Here, we present a quality assessment for structural (T1-weighted) images using tissue classification in the SPM/CAT12 ecosystem. We introduce multiple useful image quality measures, standardize them into quality scales, and combine them into an integrated structural image quality rating to facilitate the interpretation and fast identification of outliers with (motion) artifacts. The reliability and robustness of the measures are evaluated using synthetic and real datasets. Our study results demonstrate that the proposed measures are robust to simulated segmentation problems and variables of interest, such as cortical atrophy, age, sex, brain size, and severe disease-related changes, and might facilitate the separation of motion artifacts based on within-protocol deviations.

**Conclusion:**

The quality control framework presents a simple but powerful tool for the use in research and clinical settings.

## Background

**Figure 1:**
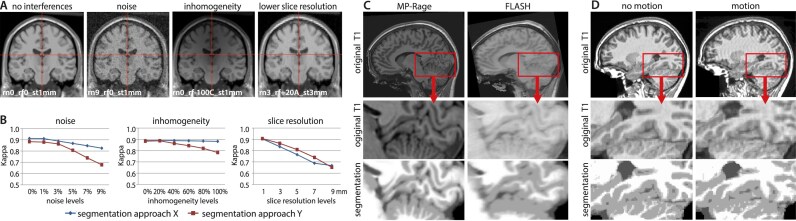
(A) Image properties such as noise, inhomogeneity, and resolution influence the segmentation accuracy. (B) The segmentation accuracy can be quantified by the κ similarity statistic [31], here presented for 2 segmentation approaches on simulated images [8], where larger levels of noise, inhomogeneity, or lower resolutions result in a worse overlap with the full-resolution image without interferences. (C) An illustration in real data with reduced anatomical details in a FLASH protocol [32] or (D) in case of movement artifacts (MR-ART sub-988484 from [9]).

Multicenter magnetic resonance imaging (MRI) studies and data-sharing projects have become increasingly common in cognitive and clinical neuroscience in recent years. The collaboration of several imaging centers, allowing for increased statistical power through larger sample sizes, is especially beneficial for investigating rare diseases and individual differences [1–3]. However, project deviations from the initial research plans (e.g., switching from functional to a structural imaging focus), differences and changes of imaging hardware and software, quality assurance procedures, and the resulting image quality variations may introduce bias in subsequent image processing and statistical analysis [4–7]. In particular, the presence of noise, (motion) artifacts, inhomogeneity, or reduced resolution could affect image processing, even when such interferences are modeled and partially corrected during data processing [8–10] (Fig. 1).

Typically, manual quality control (QC) checks each image for scan-specific interferences (e.g., motion artifacts) by visual inspection to remove outliers [6, 11, 12]. However, manual assessment is time-consuming, highly subjective, and typically relies on project-specific definitions [9]. To make this process more efficient and reliable, automated quality control approaches have been proposed for structural [5, 13, 14], functional (e.g., [15]), and diffusion imaging (e.g., [16]). In addition, the image quality estimates can be used to harmonize imaging data [17–19]. A systematic overview of different QC frameworks has been provided by [20].

In this study, we propose a powerful and easily applicable QC framework for structural (T1-weighted) MRI data within the SPM/CAT12 framework. Earlier versions have been extensively evaluated in [21] and [22]. The proposed QC framework introduces, standardizes, and integrates different quality metrics into a continuous structural image quality rating (SIQR). It supports both automatic and interactive assessments of a preprocessed MRI scan’s suitability for prospective use, as well as the identification of potential outliers within a sample, ensuring unbiased data analysis. All measures and tools are part of the Computational Anatomy Toolbox (CAT) [23–25] of the Statistical Parametric Mapping (SPM) [26–28] software and also available as a standalone version [29]. All additional supporting data are available in the *GigaScience* repository, GigaDB [30].

### Findings

Here we present the rationale for a segmentation-based QC framework, a definition of several quality measures, their standardization into quality scales, and the integrated composite measure, SIQR. We further describe the detection of imaging artifacts based on the within-sample quality and introduce the interactive outlier detection. Finally, we evaluate the proposed measures using simulated and real MRI data.

### Segmentation-based image quality assessment

**Figure 2:**
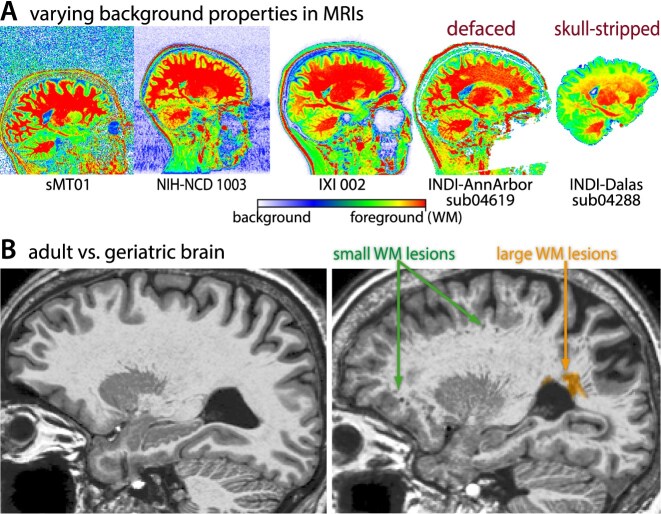
(A) Due to several different background types in real samples, only the brain tissues (excluding the background) were used for the evaluation of image quality. (B) To avoid side effects from age-related changes in volume and structure, the tissue segmentation is optimized to avoid tissue boundaries and perivascular spaces by morphological operations and masking.

For practical reasons, our QC framework uses the raw NIFTI format rather than the original DICOM format, as NIFTI images are more commonly available in public datasets and are more often used as input in data-processing tools [2]. The QC framework relies on an existing conventional or deep learning–based classification of brain tissues, which is usually a prerequisite for subsequent brain image analyses (e.g., [25, 33–35]). All proposed measures are based on image properties primarily within the brain because the background might be affected by anonymization, noise, or artifacts that do not necessarily affect the brain itself (Fig. 2A) [6, 36]. The quality measures are optimized to avoid the evaluation within parts of the brain that are typically affected by aging-related tissue changes, such as white matter hyperintensities, small vessel disease, and perivascular spaces (Fig. 2B). Within the CAT12 toolbox, the QC is the final step of the preprocessing and extends the processing time of a subject by only a few seconds. Alternatively, it can be run separately as an SPM batch for a preexisting tissue segmentation (e.g., by SPM).

The primary conceptualization and evaluation of our proposed QC measures is based on the Brain Web Phantom (BWP) [8], a simulated MRI dataset, which presents a well-established standard for developing and comparing processing methods. The dataset includes images with varying levels of noise, inhomogeneities, and resolution. These image properties affect the segmentation accuracy of MRI processing algorithms (Fig. 1B) and are therefore useful indicators of the quality of input data.

### Quality measures

As our measures have been optimized for use in cognitive and clinical neuroscience studies, the presentation is focused on practicality. A full (technical) description can be found in the Methods section. For intensity-based measures, we use measure-to-contrast ratios instead of contrast-to-measure ratios. This approach ensures that the ratings follow a linear scale rather than a logarithmic one, as defined by the BWP. For a comparison to traditional contrast-to-measure ratio, please see the Methods section.

Several key quality metrics are considered:

- **Noise-to-contrast ratio (NCR)**: This metric estimates image noise by calculating the lowest average local standard deviation of voxel intensities in the bias-corrected image. It is assessed within optimized cerebrospinal fluid (CSF) and white matter (WM) regions and is highly sensitive for other high-frequency artifacts such as motion.
- **Inhomogeneity-to-contrast ratio (ICR):** This measure evaluates intensity variations across the image by calculating the global standard deviation of smoothed intensities within the optimized WM segment.
- **Resolution score (RES):** To account for distortions due to anisotropic resolution, this score is directly computed using the root mean square (RMS) equation.
- **Edge-to-contrast ratio (ECR):** Since resampling or smoothing can degrade voxel resolution, we suggest an additional measure that captures the average slope of intensity changes at the gray matter (GM)/WM boundary. This helps assess the sharpness of tissue interfaces.
- **Full-brain Euler characteristic (FEC):** This metric quantifies the topological integrity of the WM brain interface, helping to detect potential distortions caused by noise and (motion) artifacts.

These measures provide a comprehensive assessment of structural MRI image quality, ensuring that intensity-based distortions, resolution issues, and structural inconsistencies that are relevant for the brain tissue segmentation, thickness estimation, and surface reconstruction are identified and accounted for.

### Standardization of measures

**Figure 3:**
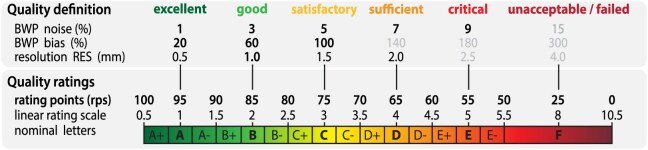
Quality rating system: the percentage, numerical, and character grades were scaled on the basis of the BWP, which represents a standard for evaluation of image-processing methods. It should be noted that excellent ratings are reserved for images with exceptional quality, whereas typical scientific data generally receive “only” good assessments.

Standardization into a normative range can enable simpler comparison across studies and support easier interpretations. To accommodate various international rating systems, we have adopted a linear percentage and a corresponding (alpha-)numeric scaling (Fig. 3, QR_percentage_ = 105–QR_grade_ * 10, QR_grade_ = (105 – QR_percentage_)/10). The quality rating ranges from 0.5 (100 rating points [rps]; grade A^+^) to 10.5 (0 rps; grade F) for highest and lowest image quality, respectively. Numerical values provide a specific rating, whereas letters describe quality ranges (e.g., grade A describes values between 90 and 100 rps). Scaling of the quality measures was performed using half of the BWP dataset, while the other half was used for evaluation (see “Evaluation concept and data”). Although the BWP does not include the simulation of motion artifacts, these are in general comparable to an increase of noise in the BWP dataset by 2 percentage points, as demonstrated in the Result section. In our QC measures, this roughly corresponds to an increase of +1 grade or −10 rps compared to motion-free data. For improved (human) readability, we standardized all measures by applying a simple linear scaling function


(1) \begin{eqnarray*}
{\mathrm{Q}}{{{\mathrm{R}}}_{{\mathrm{grade}}}} &=& {\mathrm{max}}\left( {.5,{\mathrm{min}}\left( {10.5,\left( {{\mathrm{Q}}{{{\mathrm{M}}}_{{\mathrm{grade}}}} - {\mathrm{WQ}}{{{\mathrm{M}}}_{{\mathrm{grade}}}}} \right)/} \right.} \right. \\
&& \left.{\left.{ \left( {{\mathrm{BQ}}{{{\mathrm{M}}}_{{\mathrm{grade}}}} - {\mathrm{WQ}}{{{\mathrm{M}}}_{{\mathrm{grade}}}}} \right)*6 + .5\ } \right)} \right)
\end{eqnarray*}

to transform the original quality measure (QM) into a quality rating (QR), with BQM as the best (95 rps, grade 1) and WQM (45 rps, grade 5) as the worst regular value.

### Integrated structural image quality rating

The SIQR is defined using an exponentially weighted average of multiple quality scores QR_grade_ (see Equation 2). This single composite score integrates various aspects of image quality, providing a robust metric for assessing structural image quality and identifying potential outliers. We excluded the ICR from the composite SIQR measure because most preprocessing methods can handle bias quite well, and the effects of signal intensity changes are already considered by the NCR. Integrating the ICR was therefore contraintuitive, as high field strength resulted in worse ratings that did not fit to the outcome.


(2) \begin{eqnarray*}
{\mathrm{SIQR}} = {{\left( {{\mathrm{mean}}\left( {{{{\left[ {{\mathrm{NCR}},{\mathrm{RES}},{\mathrm{ECR}},{\mathrm{FEC}}} \right]}}^4}} \right)} \right)}^{(1/4)}}
\end{eqnarray*}

To balance the sensitivity to different quality measures while ensuring that the necessary quality conditions are met, we apply an exponentially weighted averaging approach to the graded quality ratings (range 0.5–10.5)—similar to the RMS but using the fourth power and fourth root. This method allows well-rated images to contribute positively without overshadowing critical quality constraints. For more information regarding the weighting-selection process, we direct the reader to the Methods section.

### Sample normalization for outlier detection

So far, each image has been evaluated individually, enabling the detection of outliers with very low resolution or high noise (e.g., those falling below a C rating, such as SIQR <70 rps). However, to identify more subtle issues, such as mild motion artifacts, it is necessary to assess deviations from the ideal quality expected for a given MRI protocol within a specific sample. To achieve this, we estimate the upper quartile of the SIQR percentage scores from images acquired with the same protocol and apply a linear correction (a simple translation, as the values have already been scaled according to the BWP). This normalization results in a standardized SIQR, where values close to zero indicate optimal protocol quality, while higher values highlight potential outliers. To establish a general threshold for detecting quality issues, we employed a receiver operating characteristic (ROC) curve analysis combined with 2-fold cross-validation (splitting the dataset into odd- and even-numbered files based on filenames). This approach was validated using test samples with expert ratings, ensuring robust performance in identifying suboptimal images. The normalized scores were processed using the “Check Sample Homogeneity” tool (described in the next section) and saved as a normalized SIQR (nSIQR) score in the subject’s XML file and in a CSV table.

### Software

**Figure 4:**
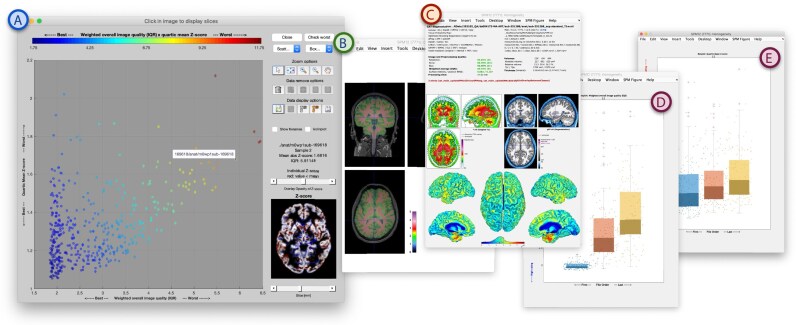
The “Check Sample Homogeneity” Tool in CAT12 for the MR-ART dataset grouped by the amount of motion (see D and E). (A) In the main window, the user can select scans that are ready for analysis. The QC ratings, generated independently during preprocessing, are automatically loaded from the corresponding XML files. Users can interactively explore data points to investigate deviating ratings by viewing the original image with its segmentation overlay (B) or the preprocessing report (C) to remove outliers with image- and processing-related problems or atypical anatomical features. Grouping of multiple scans allows the estimation of a sample-specific nSIQR score and *z*-scores that are added to the individual XML files and stored as a CSV table. The normalization utilizes a scanning site variable to subtract the default protocol quality (defined by the upper quantile), highlighting scans with motion artifacts as outliers (E; see also Fig. 6C, D).

The “Check Sample Homogeneity” tool (Fig. 4) in the CAT12 toolbox supports a guided analysis of large datasets to detect and exclude outliers in anatomy, preprocessing, and image quality from analysis by estimating a sample-specific *z*-score. The tool has been designed in an interactive format with the intention to encourage users to get in touch with their data and carefully decide on the inclusion/exclusion of the images from analyses. Artifacts can result in a systematic bias, often resulting in an underestimation of GM [7]. To ensure the validity of statistical analyses, it is suggested that severe image quality-related outliers are excluded based on normative assessments provided by the toolbox. The quality estimation is also available as the “Image Quality Estimation” SPM batch to process selected structural scans with a fitting brain tissue segmentation (e.g., from SPM). The results for each input image are stored in an XML file and can be used for subsequent analysis steps and potential analysis in relation to effects of interest of a study (such as age).

### Evaluation concept and data

The calibration and testing of our proposed measures were done using simulated images from BWP [8, 37, 38] and the Cortical Aging Phantom (CAP) [39], as well as real data from IXI, ATLAS [40], MR-ART [9], and a test–retest dataset (Table 1).

**Figure 5:**
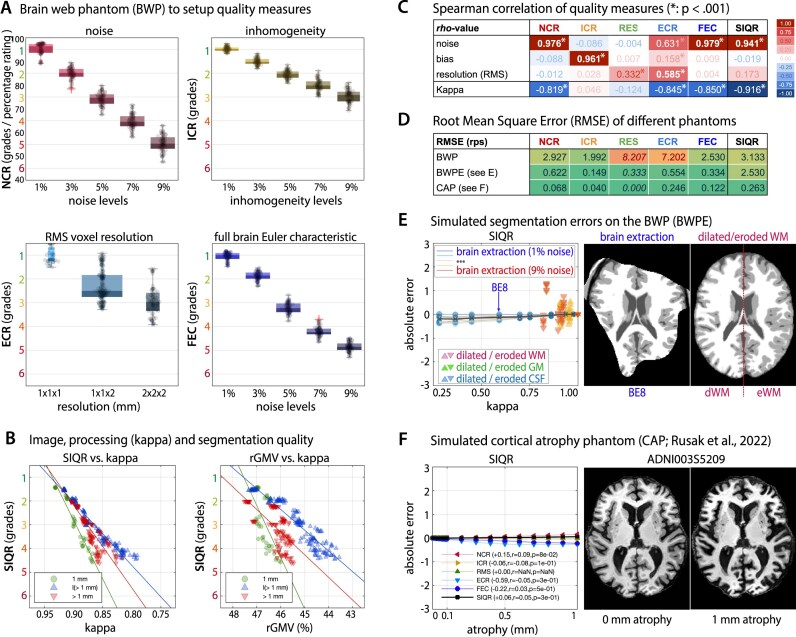
(A) The dependency of quality measures on manipulated levels of noise, inhomogeneity, and resolution using the BWP: NCR, ICR, ECR, and FEC. (B) The SIQR integrates all these measures into 1 score and shows significant associations with segmentation quality (characterized by κ) and the rGMV based on CAT12. (C) Overall, our ratings show specific relationships to their corresponding BWP perturbations but not others and (D) small RMSEs also in the case of simulated segmentation errors (E) or aging (F). Of note, the numerical grading system and percentage system are inversely scaled, where –10 rps correspond to +1 grade and roughly correspond to the emergence of obvious motion artifacts. * See the Supplementary Table S1 for the full table in C.

The BWP dataset consists of simulated data files of varying noise, inhomogeneities, and resolution parameters, which are encoded in the filenames. To create balanced and comprehensive calibration and testing samples containing similar—but not identical—cases, every second data point was assigned alternately to each sample. The BWP calibration data consisted of all odd files (ordered by filename) and were used to scale the quality measures and estimate the weighted averaging described above. The test subset (even files) was used to quantify the relationship between quality ratings and segmentation accuracy. Moreover, we used the BWP to further simulate typical brain extraction and segmentation artifacts (e.g., by erosion/dilation of tissue segments) to test the robustness of the quality measures in case of critical data conditions (see Fig. 5). The CAP described in [39] was used to test the effects of brain atrophy of up to 1 mm on our quality measures.

Although simulated data enable basic evaluation under defined conditions, real data are essential to investigate possible dependencies/biases. The measures were quantified in IXI, ADHD200, and ATLAS datasets to test for possible effects of age, sex, and lesions. Finally, the MR-ART dataset with 148 subjects, each with 3 scans without light, with light, and with severe motion artifacts, along with available expert ratings as well as MRIQC derivatives [13], was used to test the utility of our measures to separate images with motion artifacts and to validate the measures against an established QC framework.

Additionally, we used the Tohoku test–retest (TRT) dataset, which contains 126 T1-weighted scans [41]. All scans were preprocessed, registered, and resliced to a high-resolution template with 0.50 mm isotropic resolution. A median template was used to remove outliers and to create the final ground-truth segmentation by averaging. Finally, we estimated the association of image quality and scan time.

All evaluation scripts are available in the CAT distribution on GitHub and require MATLAB with the statistical toolbox and curve fitting toolbox to run. The required raw data of IXI, CAP, ADHD200, ATLAS, and MR-ART are available from the project-specific websites, as described in Table 1. The preprocessed data and the reorganized images of the BWP(E) and the TRT are available on the *GigaScience* repository, GigaDB [30].

**Table 1:** Short overview of the used datasets

Dataset	*N*	Age (years)]	Sex (% men)	Sites	Description
BWP	600	~30	100%	1	Simulated dataset [8, 42] for basic definition (calibration: odd files) and evaluation (test: even files) of the quality ratings, with 5 levels of noise (1% to 9%), 3 different bias fields with 5 levels (20% to 100%), and 8 resolution levels with a voxel length of 1 and 2 mm.
BWPE		~30	100%	1	BWP data with simulated skull-stripping and segmentation errors (see Fig. 5) to test the robustness of our measures in case of severe processing problems.
CAP	400	39.1–78.3 (70.7 ± 5.4)	50%	1	Simulated atrophy dataset [39, 43] to test for side effects of GM tissue atrophy that correspond to aging of about 100 years.
IXI	554	20–86 (48.5 ± 16.4)	44.8%	3	Brain aging sample to test the effects of age, brain size, and sex (only scans with complete phenotypic data) [44].
ATLAS (R1.2)	304	NA	NA	11	T1 images of subjects with (masked/unmasked) lesions to test the stability in case of severe structural changes [40] (controlled access: [45]).
ADHD200	491		52.9	7	T1 images of healthy subjects of the train dataset (site 2 of 8 is only in the test dataset) [46].
MR-ART	148 * 3	18–75 (30.0 ± 12.8)	35.1%	1	Dataset without and with intended motion artifacts [9, 47].
TRT	6 (127)	~30	100%	1	Various magnetic resonance protocols with a scan time duration from 30 seconds to 11 minutes on a 3T Philips scanner [41]. The scans were selected based on differences in scan time driven by resolution and parallel imaging (SENSE), while ensuring the similarity of other MRI parameters (see supplementary table S2)

## Results

The quality scores were first evaluated on the simulated test data to determine the accuracy of interference quantification and to investigate how robust the measures are in cases of simulated segmentation problems and aging. Furthermore, we used the IXI, ADHD200, and ATLAS datasets to study the effects of aging, sex, brain size, and stroke lesion on our proposed measures of image quality. Additionally, we evaluated the ability to detect images with motion artifacts on the MR-ART dataset, tested the validity of our measures against the MRIQC 0.16.1 derivatives, and demonstrated the application in a test–retest scenario. All measures had been standardized (see Fig. 3) and evaluated on the BWP before focusing on the averaged SIQR score. Of note, obvious subject/scan-specific motion artifacts generally increase the scans’ rating for about 1 grade, which corresponds to a decrease of 10 rps (and +0.5 grade/−5 rps for light artifacts), in comparison to the typical rating achieved by most scans of the same protocol (see Fig. 7). Similar to the Methods section, we focus here on the results pertaining to SIQR and refer the interested reader to the Methods section for a more detailed overview.

### Simulated data

The evaluation on the BWP test dataset showed that most quality ratings have a very high correlation (Spearman’s ρ > .950, *P* < 0.001) with their corresponding perturbation and a very low correlation (Spearman’s ρ < |0.1|) with the other tested perturbations (see table in Fig. 5A, C). This suggests considerable specificity of the proposed quality measures. The combined SIQR score also showed a very strong association with the segmentation quality κ (Spearman’s ρ = –.916, *P* < 0.001) and brain tissue volumes (Spearman’s ρ_CSF/GM/WM_ = –.729/–.647/.805, *P*_CSF/GM/WM_ < 0.001) (Fig. 5B). The root mean square errors (RMSEs) between the expected and measured values of the SIQR were 3.133, 2.530, and 0.263 rps for the BWP test set, the BWP-derived segmentation error test set, and the cortical atrophy phantom (CAP), respectively (Fig. 5D).

Most notable is the quantification of the image resolution, where the simple voxel-based resolution rating RES did not work well in interpolated data (i.e., as expected in 225 of 625 cases), resulting in a lower correlation (Spearman’s ρ = .332) and a high RMSE of 8.207 rps. The edge-based resolution measure (ECR), on the other hand, generally performed better (Spearman’s ρ = .585, *P* < 0.001) but was strongly affected by noise (Spearman’s ρ = .631, *P* < 0.001) and inhomogeneity (Spearman’s ρ = .158, *P* < 0.001) compared with other scores. The tests with simulated segmentation errors suggested that NCR and ICR were extremely robust (Fig. 5E), whereas ECR and especially FEC were quite sensitive to strong (i.e., 1 voxel) over-/underestimations of CSF and WM.

### Real data

**Figure 6:**
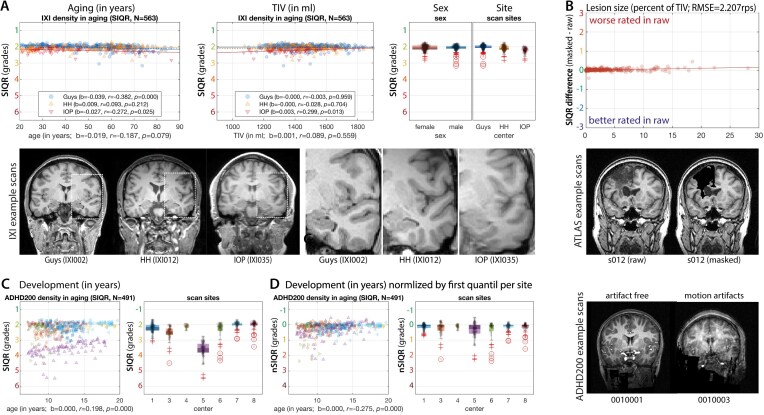
(A) The results of the structural dependency test in the IXI dataset showed that the SIQR measure is independent of age and TIV and only slightly associated with sex (see the Methods section for other quality measures), with an average standard deviation 1.625 rps per site. (B) The results from the ATLAS dataset suggest that severe structural changes in terms of lesions do not significantly affect the SIQR measure when comparing raw versus masked images. (C) SIQR measurements in the ADHD200 dataset for children and young adults acquired by 7 different centers, including scans with motion artifacts. (D) To identify outliers with motion artifacts (over multiple sites), a normalization by the typical protocol quality (defined by the first quantile of the SIQR values per site) can be used, where scans with more than 5/10 rps ratings typically have light/strong motion artifacts, respectively (see MR-ART dataset in Fig. 7). Note that our rating system is designed to assist in identifying cases that require further human evaluation, depending on the needs of the study.

The real data analysis of IXI and ATLAS cohorts suggests that the proposed quality measures were not affected by total intracranial volume (TIV, *r*_SIQR_ = .089, *P*_SIQR_ = 0.559), age (*r*_SIQR_ = −.187, *P*_SIQR_ = 0.079; Fig. 6A), sex (Mann–Whitney *U* test: *U* = 39,125, *Z* = 0.584, *P* = 0.558), or stroke lesions in the ATLAS dataset (Fig. 6B), while sex showed minor effects in IXI. Since IXI rather contains scans without significant motion artifacts, it provides a useful estimate of the typical overall variability in terms of the standard deviation of the SIQR score with 1.625 rps (Guys/HH/IOP = 1.629/1.606/1.641 rps). However, outliers with motion artifacts are often found in children, as shown in the ADHD200 dataset (Fig. 6C), with an average standard deviation of the SIQR scores of 2.078 rps (sites 1, 3–7 = 3.543/1.578/1.493/4.061/1.528/1.314/1.0273 rps).

**Figure 7:**
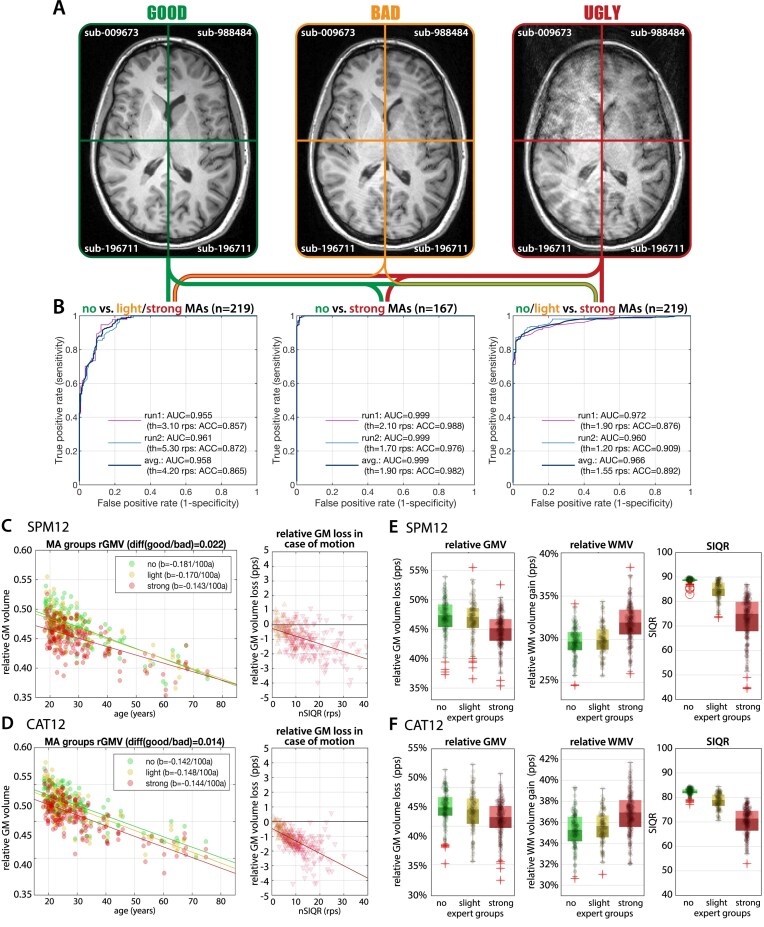
(A) Example images from the MR-ART dataset [9] for 3 different conditions based on expert ratings splitting data into (no), (light), and (strong) motion artifacts (MAs). (B) ROC curves when classifying these groups using SIQR (see the Methods section for ROCs of other quality ratings) with high accuracy (ACC) and AUC. The thresholds (th) of the ROC were estimated on the nSIQR values and applied in a split-half design (run1 vs. run2). We tested 3 options in handling cases with light MAs, where the no versus strong test case was only possible in a smaller subsample (*n* = 167), ignoring the controversial light cases; only 4 cases were misclassified (C; see the supplement for the failed cases for the 2 groupings with light motion artifacts). To separate all motion cases, a threshold of about 5 rps worked best, whereas the separation of strong motion artifacts needed a higher threshold of about 7 to 9 rps. In other words, scans with a rating of 5 to 10 rps lower than the typical quality of the protocol should be checked. (C, D) We further evaluated the relative GM tissue volume change (in relation to aging) in percentage points (pps) of scans with motion artifacts compared to the ones without for each subject processed by SPM and CAT12. As the MR-ART dataset only includes motion-free and affected scans, we estimated the tissue changes in motion cases compared to the one without motion. The results suggest that the expected lower segmentation accuracy for lower image quality leads to GM underestimation and WM overestimation, where motion artifacts of 10 rps are roughly comparable to a GM loss within 5 years. (E, F) The boxplot of the 3 expert-defined motion groups clearly showed the expected GM underestimation, WM overestimation, and SIQR ratings in case of strong motion in SPM and CAT12.

The effects of motion artifacts were evaluated using the MR-ART dataset (Fig. 7A). In order to detect motion artifacts, each score was normalized (by subtracting the first quartile value to consider the typical protocol quality), and an ROC was applied. The measures were tested under 3 conditions, namely comparing (i) no versus severe artifacts, (ii) no versus light + severe artifacts, and (iii) no + light versus severe artifacts (Fig. 7B). The best ROC thresholds to separate good from bad scans in the 3 groups were 4.20/1.90/1.55 rps for the nSIQR. The accuracy of the SIQR, as determined by the ROC (an average over the 3 groups), was 0.902 and 0.899, with an area under curve (AUC) of 0.974 and 0.969 for CAT12 and SPM, respectively. The failure cases where the measure was not in accordance with the expert ratings are available in the supplementary material.

Moreover, we have demonstrated the expected decrease of GMV with aging and in relation to motion artifacts in the MR-ART dataset for CAT12 and SPM25 segmentation (Fig. 7C–F). The results indicate that higher segmentation error (measured by the κ statistic comparing motion-free and motion artifact–containing scans) and hence lower image quality may lead to underestimation of GM and overestimation of WM volume.

**Figure 8:**
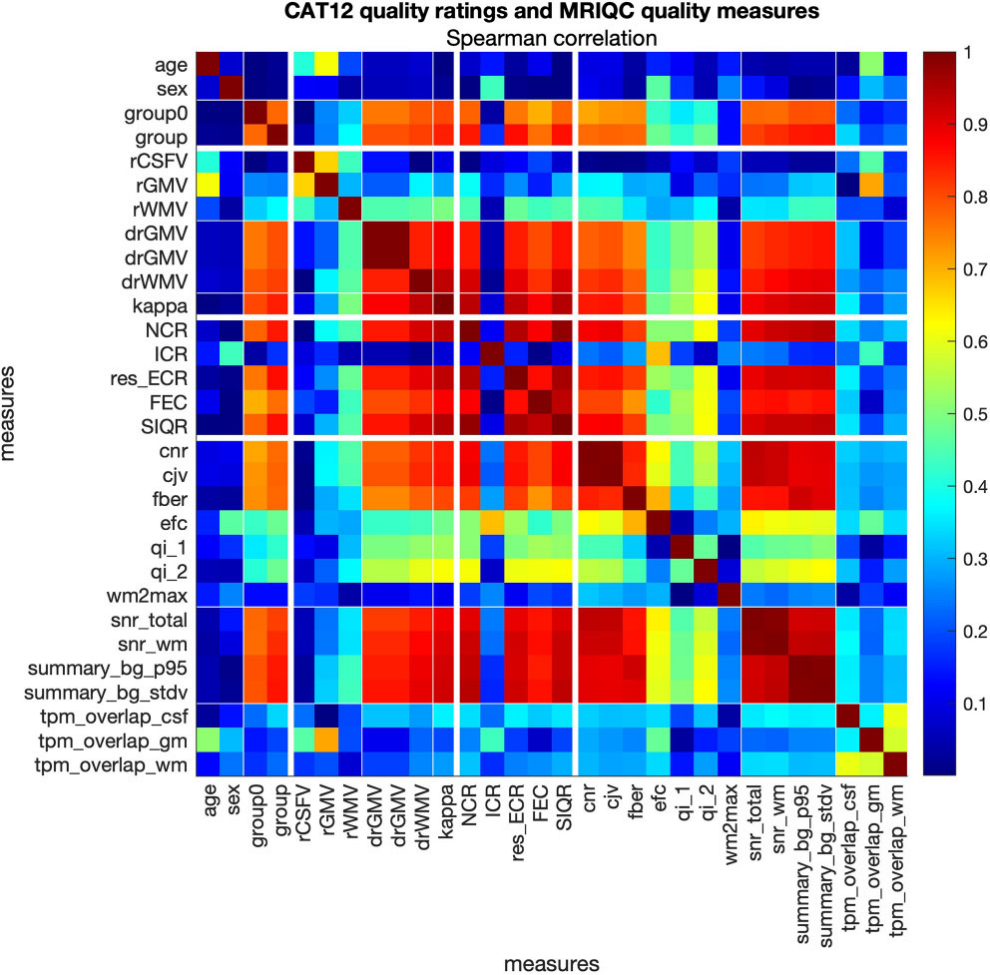
Spearman correlation coefficients between expert rating (i.e., expert rating group: 1, no motion; 2, light motion; 3, severe motion), ΔV (dvol_rel_CGW, volume change in relation to motion-free scan), κ statistic (estimated with respect to the motion-free scan and averaged across all tissues), CAT12 quality ratings (NCR, ICR, res_ECR, FEC, and the weighted average SIQR), and selected MRIQC quality measures in the MR-ART dataset [9] (for full table, see Supplementary Fig. S6).

We further tested the association of our measures with the established measures from the MRIQC 0.16.1 [13] (see [9] for the processing). The results are presented for selected MRIQC measures in Fig. 8. SIQR was highly associated with a signal-to-noise ratio of MRIQC, especially that of white matter (ρ = .927, *P* < 0.001), as well as summary standard deviation of the background (ρ = −.937, *P* < 0.001). For associations with all the measures from MRIQC, see Supplementary Fig. S6.

**Figure 9:**
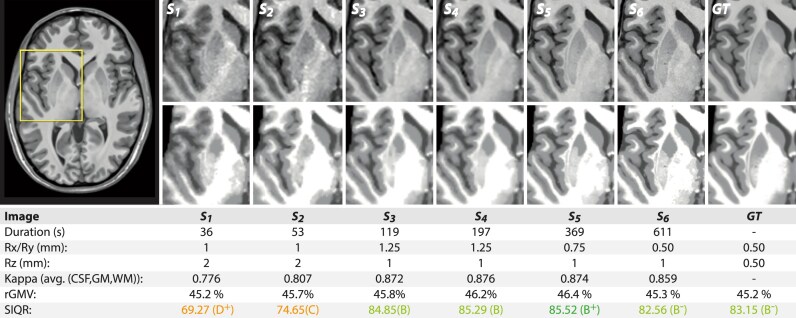
An illustrative example of the test–retest sample analysis showing 6 images with an increasing scan time and image quality. The top and bottom rows represent the intensity-normalized T1 images and the CAT12 segmentation, respectively, with increasing segmentation accuracy in longer scan times when compared to the average ground truth (created from the best 127 test–retest images). The rGMV is slightly underestimated in low-quality data.

Finally, we validated the proposed quality metrics using a scan–rescan test where we inspected the difference in quality scores and segmentation accuracy with regard to scanning time and ground-truth image, respectively (Fig. 9). The expected improvement of image quality was clearly observable in terms of sharper anatomical details and reduced noise. The κ indices, relative gray matter volume (rGMV), and SIQR scores confirmed these visual observations but pointed out further interesting details. The noisy short time scans S1 and S2 showed significantly lower κ, worse SIQR ratings, and smaller GM volumes, whereas the other scans were highly comparable.

## Discussion

Here, we introduced a QC framework for structural (T1-weighted) MRI data. We defined and validated various automated quality ratings based on well-defined BWP image quality features, such as noise, inhomogeneity, and resolution [8], and integrated them into a single SIQR score to facilitate practical applications in the context of clinical and cognitive neuroscience. We further demonstrated that our measures (i) are robust to simulated segmentation problems and cortical atrophy; (ii) are independent of sex and brain size, showing only minor expected associations with chronological age, as well as severe disease-related changes; and (iii) allow the reliable assessment of motion artifacts within a protocol. In artifact-free data, image quality typically varies between 2.5 and 5 rps (0.25–0.5 grade), whereas light or strong artifacts typically result in a reduction of ratings by 5 or 10 rps (equivalent to a 0.5 or 1.0 increase in grades), respectively. T1-weighted images with low-quality ratings might show a systematic underestimation of gray matter volume, first demonstrated in the case of motion artifacts by [7]. Strongly affected data should therefore be excluded from the analyses. In the case of less severe artifacts, the quality rating might be included as a covariate [17, 19] or using weighted least squares [18] during statistical analyses. However, an empirical comparison of the complex statistical effects of alternative approaches to account for automatically generated (i.e., known) quality differences in downstream analysis tasks is still lacking.

The proposed QC framework offers a simple and efficient approach to identify structural MRI scans that are suitable for the prospective use in structural processing tools (especially within SPM/CAT12 framework) and brain imaging analysis in both clinical and research settings. This was also confirmed in previous studies [21, 22, 48] that evaluated the utility of earlier versions of this QC framework.

In the following sections, we discuss further aspects of the development of our SIQR measure and its subordinate quality measures (with regard to existing alternatives), their performance in simulated and real data samples, and their potential for assessing the quality of images from other sequences and modalities.

### SIQR measure development

Various image quality frameworks estimate some of their quality metrics based on information extracted from the image background [13, 14, 49]. In contrast, our approach focuses on estimating quality only within the brain for 3 reasons. First, the background values in public datasets can be corrupted by various defacing and skull-stripping routines [50, 51]. Second, the background may contain artifacts (e.g., motion artifacts from the jaw or tongue) or unwanted properties (e.g., noisy backgrounds in MP2RAGE) [36] that do not necessarily affect the brain, or conversely, the artifacts in the brain do not or are less likely to affect the background. Third, the background does not provide information about image inhomogeneity, tissue contrast, and spatial anatomical resolution [52]. While certain artifacts may be more prominently visible in the background [14], they are of interest to the user only if they also affect the brain. Furthermore, the evaluation of image quality within tissues must take into account structural aspects such as (i) the partial volume effect, where a voxel contains tissue of more than 1 tissue class, and (ii) changes in brain development and aging, such as tissue degeneration due to white matter hyperintensities, small vessel disease, or perivascular spaces [53, 54]. Consequently, the proposed framework adapts these regions of interest by applying specific thresholds and morphological operations to minimize bias from age/disease, as we have demonstrated in the IXI and ATLAS datasets. Moreover, the proposed intensity-based measures are normalized by (minimum) tissue contrast rather than signal intensity, as the separation between brain tissues, especially the GM and WM, is essential for segmentation and surface reconstruction [25, 55].

Our proposed individual quality subscores have largely been established based on well-known image quality aspects of the BWP, which was built to represent the large variability in image quality of structural T1-weighted MR images [8, 56, 57]. By taking into account these predominant aspects of image quality, we have created ratings that are easy to understand, even without a technical background. The ratings were integrated into a single SIQR rating to support the users during the evaluation process. To combine the measures, we have used an RMS-weighted average (of the grades) with a power of 4 rather than 2, to place greater emphasis on the more problematic aspects of image quality. This is relevant because effects of severe problems often cannot be compensated by other factors (e.g., if there are severe motion artifacts, a much higher image resolution typically cannot account for this).

In particular, SIQR is strongly predictive of segmentation accuracy (quantified by the κ measure) and the extracted GM volume, although its quantification is largely independent of structural features. Thus, SIQR can facilitate the estimation of image quality–related variance in individual scans or samples, even for nonexpert. Alternative quality control tools, such as MRIQC [13], might be challenging for novices due to nonstandardized measures that require substantial user experience. Moreover, a normalization using BWP quality features also enables a direct comparison across protocols (see test–retest example), although caution is advised, as the results may be subject to bias by (i) our focus on a segmentation-centered definition of quality, (ii) the population under study, and (iii) project-specific needs or considerations (e.g., optimized magnetgic resonance [MR] parameters to image specific structural changes rather than preprocessing).

### Identification of scans with data anomalies and artifacts

The proposed framework is part of the CAT12 preprocessing and utilizes the CAT12 segmentation, but it could also be used as an independent SPM batch with other segmentation algorithms (e.g., from SPM) [33]. Segmentation routines are widely used for structural brain analyses and have undergone intensive testing to be valid, accurate, and robust for a variety of protocols, individual anatomies, and demographics (e.g., [25, 33, 35]), making them ideal for image quality analysis. By focusing on general global aspects of the scan rather than local ones, problematic structures and areas such as partial volume effect voxels or WM lesions can be omitted, allowing precise, robust, and largely consistent results, even in case of severe classification faults (e.g., failed skull-stripping or misclassification), as tested here under simulated conditions.

Although SIQR could be used for fully automatic outlier detection (see also [21] and [50]), we believe that the huge variability of the type of artifacts, their regional occurrence, and their impact on image processing still require study-specific knowledge and, if possible, a short user inspection—for instance, when locally limited or mild artifacts affect regions that are not relevant to the study (e.g., if the study focuses on frontal regions, cerebellar artifacts from jaw movements are acceptable) or whenever lower preprocessing accuracy is acceptable (e.g., for local alignment of brain surfaces or atlases for other modalities).

Multivariate outlier detection schemes that are typically applied based on the processed data of a sample in the normalized feature space, using similarity analysis of normalized GM data (e.g., the Gram matrix or kernels) in CAT12 (see Software section), can be used to detect outliers with preprocessing problems or highly deviating anatomy. However, the proposed image quality assessments are specifically designed to measure differences of image quality (in native space) rather than segmentation accuracy (in normalized space) or anatomical properties, such as stroke lesions, and can therefore be used in addition to previously mentioned outlier detection schemes to identify cases where image artifacts could bias analysis.

### The role of subordinate quality ratings

While the SIQR composite is sufficient for most analyses, our framework facilitates deeper insights, providing more specific subordinate ratings.

The NCR is not only a very robust measure, as it can be quantified in different regions, but also very sensitive to motion artifacts, and it gives the most relevant values when the image resolution is adequate (<1.5 mm).

The ICR had little to no impact on detecting problematic data (e.g., when testing for segmentation quality κ in the BWP or motion artifacts in MR-ART), as inhomogeneities tend to describe more protocol/scanner-specific aspects and can be corrected fairly well in most protocols [58]. Increased inhomogeneities typically occur in high-field scans without protocol-specific correction schemes. Although possible disadvantages are generally outweighed by superior resolution and higher signal-to-noise ratio, bias correction schemes in preprocessing routines can fail in some cases [59]. It is therefore recommended to retain this measure in a general rating.

The RES rates the voxel size in terms of how good structural features can be imaged. Nevertheless, structures can still be biased by interpolation [10], blurring, noise, or motion artifacts. A real quantification of the sharpness of anatomical structures by our ECR is therefore essential, although it is strongly affected by noise and the segmentation quality compared to other ratings.

The FEC represents our adaptation of surface topology [60, 61]. The measure showed a strong association with noise levels and supports the identification of motion artifacts. However, compared to NCR, it is noisier and depends strongly on the input segmentation and MR protocol. Data with a low spatial resolution or faulty/simplified segmentations with a limited number of details can have fewer defects and result in better ratings. Nevertheless, FEC presents a good extension to the NCR and ECR measurements.

In contrast to MRIQC [13], which provides a variety of raw unscaled measures (with reversely signed scored ones among them), we tried to establish measures that reflect the known specific perturbances and are directly interpretable by applied scientists. Nevertheless, raw quality measures are also available in the XML files, allowing advanced users to perform detailed inspections. All QC measures can be used in statistical analyses or machine learning models according to the study needs [50].

### Evaluation in simulated and real data

Simulated data allow basic validation of methods under expected conditions and comparison with actual ground-truth results. The BWP is a standard for evaluating structural brain image preprocessing [56, 57] and was used here to define and normalize our quality measures and to test their relationship with the segmentation accuracy. Due to the robustness of CAT12, we decided to simulate extreme segmentation problems. The results showed high stability for the NCR and ICR measures, but high susceptibility to error for the ECR and FEC in the case of severe over/underestimation of tissue segments, as both depend on the correct definition of the GM/WM boundary. In the simulated aging phantom [39], the results showed small systematic but negligible changes in the quality measures, which could also be due to small differences in the simulated images (see bias differences in Fig. 5F). Since a lot of our tests relied on BWP, which is limited in its ability to simulate artifacts or new protocols, such as MP2RAGE, new frameworks such as TorchIO [62] present a possible next step for future tests. Nevertheless, an empirical validation on real MRI datasets was necessary to demonstrate the validity and practical benefits of the introduced quality assessments and to avoid overadaptation to synthetic data. Therefore, we used the IXI and ATLAS datasets to demonstrate that SIQR is unaffected by age, sex, head size, or severe structural disease-related changes. In MR-ART, we tested the ability to identify different degrees of motion artifacts and the effects on gray matter segmentation in aging. Overall, we demonstrated the robustness and applicability of our SIQR measure.

### Shortcomings and outlook

The QC measures proposed in this study were designed to be independent of segmentation accuracy and were tested in a variety of protocols. However, useful results can only be expected for valid segmentation inputs (where we focus on CAT12), and highly specific T1-weighted protocols may result in unexpected ratings. Other modalities, such as T2-weighted, proton density–weighted, or fluid-attenuated inversion recovery (FLAIR) images, can be assessed, but the dependance of the SIQR on the separability of CSF, GM, and WM may result in low-quality scores. In addition, our ratings are not designed to assess functional or diffusion data, where more specific tools are available [12, 15, 63]. Although such data can also be used for tissue segmentation, the low GM-WM contrast is challenging, and the resulting segmentations or surfaces are less accurate and possibly biased compared to typical T1-weighted images [33]. It is important to note that preprocessing tools are designed to work reliably even on problematic datasets, and results from these images can often still be used, although these should be interpreted with more caution. Moreover, scanner-specific changes, such as geometric distortion, have not been considered. Consequently, our measures are not designed to monitor scanner properties that require real MRI phantoms [64, 65].

In addition, our scan–rescan results demonstrated instances of comparable segmentation quality, with up to 40% faster scan times. This is particularly relevant for clinical MRI, where cost-effectiveness (short scan times for high patient throughput) presents an essential aspect, and images of adequate, but not exceptional, quality are appropriate for diagnosis [66, 67], simultaneously reducing both financial and environmental costs [68]. On the other hand, using only adequate image quality for certain projects does not eliminate the need for cutting-edge resolution [59], although improperly enhanced image resolution (e.g., 0.5 × 0.5 × 1.5 mm for 1.5 Tesla systems) often leads to increased noise or parallel imaging artifacts that can disturb preprocessing. It is therefore advisable to pilot modified protocols for the preprocessing pipelines you plan to use or follow the established standard protocols (e.g., ADNI [69] and HCP [70]).

## Conclusion

Our fully automatic quality control framework within the SPM/CAT12 ecosystem enables a standardized, accurate, and robust evaluation of large heterogeneous datasets to detect outliers with inadequate image quality using a single-image quality rating: SIQR. Its flexibility, low cost, and simplicity support a wide range of applications and can provide a valuable contribution to quality assurance in clinical practice and research.

## Methods

All the statistical analyses were performed in MATLAB 2024a. The associations between different metrics were calculated using Spearman’s rank correlation (unless stated otherwise), and the Mann–Whitney *U* test was used to compare the quality metrics between men and women.

The following section provides a more technical introduction to our quality measures.

### Normalization and scaling

**Figure 10:**
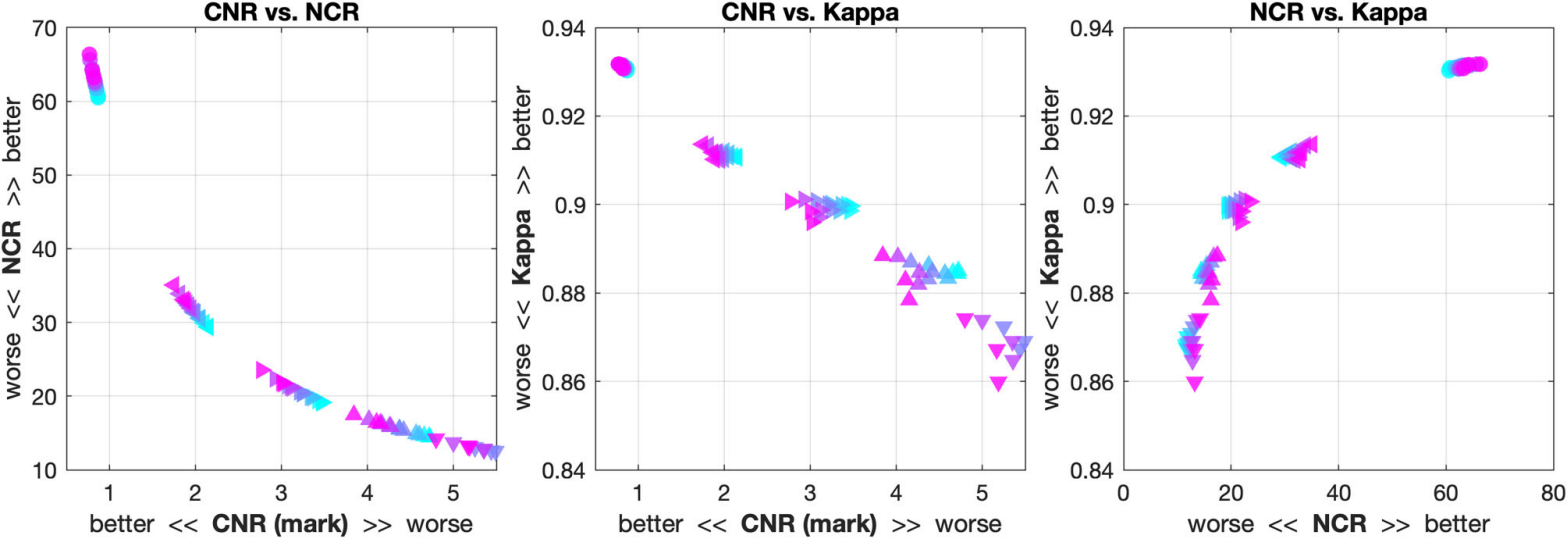
(A) The relation between the classical NCR and contrast-to-noise-ratio (CNR) (A) on the 1-mm BWP data with 1% to 9% noise and 20% to 100% bias. (B) The advantage of the CNR’s linear scaling is clearly visible in its relationship with the preprocessing quality represented by the κ value, where it allows for a better separation in the range of lower image and segmentation quality. (C) The nonlinear relation between NCR and the κ statistic (average of all brain tissues segmented by CAT12), on the other hand, enables a finer separation of high-quality data, which is less useful for detection and quantification of outliers.

As segmentation and surface reconstruction rely heavily on the contrast between tissues, the normalization by contrast rather than the signal intensity allows a better correspondence between image and processing quality. Moreover, contrast-to-measure ratios rather than measure-to-contrast ratios were used as they support linear scaling to the interferences of the BWP and a linear relationship to the κ values of CAT (Fig. 10).

Simple linear scaling function:


\begin{eqnarray*}
&&{{\mathrm{Q}}{{{\mathrm{R}}}_{{\mathrm{grade}}}} = \beta \left( {{\mathrm{Q}}{{{\mathrm{M}}}_{{\mathrm{grade}}}},{\mathrm{BQ}}{{{\mathrm{M}}}_{{\mathrm{grade}}}},{\mathrm{WQ}}{{{\mathrm{M}}}_{{\mathrm{grade}}}}} \right)}\\
&&{\quad \quad \quad \quad\!\!= \max \left( {.5,\min \left( {10.5,\left( {{\mathrm{Q}}{{{\mathrm{M}}}_{{\mathrm{grade}}}} - {\mathrm{WQ}}{{{\mathrm{M}}}_{{\mathrm{grade}}}}} \right)/} \right.} \right.}\\
&&{\left.{\left. {\left( {{\mathrm{BQ}}{{{\mathrm{M}}}_{{\mathrm{grade}}}} - {\mathrm{WQ}}{{{\mathrm{M}}}_{{\mathrm{grade}}}}} \right)*6 + .5\ } \right)} \right)}
\end{eqnarray*}

to transform the original quality measure QM into a quality rating QR, with BQM as the best (95 rps, grade 1) and WQM (45 rps, grade 5) as the worst regular value.

### Noise

The first quality rating characterizes image noise, defined here as the NCR, to describe how well the tissue can be locally separated independent of the protocol-specific tissue contrast. The estimation was specified as the minimum of the average local standard deviation, σ̃, of the bias-corrected image C_bc_ within the optimized WM and CSF regions WMe and CSFe. The values were normalized by the minimum tissue contrast c_min_ and scaled by the results of the linear fit:


\begin{eqnarray*}
{\mathrm{NCR}} = \beta ({\mathrm{min}}(\tilde{\sigma }({{C}_{bc}}({\mathrm{CSFe}})),\tilde{\sigma }({{C}_{bc}}({\mathrm{WMe}}))))/{{c}_{min}},0.0183,0.0868)\quad \left( {{\mathrm{SE}}1} \right)
\end{eqnarray*}

with 0.0130 as the best and 0.0682 as the worst rating of the unscaled measure obtained for the BWP train dataset. For data analysis, the bias-corrected image C_bc_ allows for a more meaningful characterization of the local varying noise level than the original image, since it considers processing problems in areas with low signal intensity and increased noise. The local standard deviation σ̃ was estimated in a 5 × 5 × 5 voxel neighborhood of a voxel and averaged to reduce the influence of remaining inhomogeneities.

The CSF and WM regions, rather than the background, were used because the background can contain interferences that do not affect the brain [6, 36] or could be affected by anonymization of subject features by defacing or brain extraction (Fig. 2A). CSF and WM are beneficial for noise estimation compared to the GM because they (i) cover relatively large and homogeneous areas and (ii) are less affected by partial volume effects and locally varying tissue contrast (e.g., by myelination). However, using only CSF regions often failed in younger subjects and low-resolution data, while the exclusive use of WM led to age-related effects caused by WM lesions or small vessel disease or perivascular spaces. The regions were optimized by an erosion step and additional tissue thresholds to avoid side effects by partial volumes, segmentation method, or WM lesions in elderly subjects that are quite similar to noise or artifacts (Fig. 2B). The minimum tissue contrast c_min_ between CSF, GM, and WM was used because a greater GM-WM contrast led to problems in detecting the CSF-GM and CSF-background boundaries.

### Inhomogeneity

In order to assess intensity inhomogeneity in images (often referred to as bias), the coefficient of joint variation (CJV) [52] proved to be one of the most suitable measures [58]:


\begin{eqnarray*}
{\mathrm{CJV}} = (\sigma ({{{\mathrm{C}}}_{{\mathrm{GM}}}}) + \sigma ({{{\mathrm{C}}}_{{\mathrm{WM}}}}))/|\mu ({{{\mathrm{C}}}_{{\mathrm{GM}}}}) + \mu ({{{\mathrm{C}}}_{{\mathrm{WM}}}})|\ \quad \left( {{\mathrm{SE}}2} \right)
\end{eqnarray*}

However, since it is known that the GM is strongly influenced by partial volumes and locally different GM intensities [53], only the standard deviation σ of the WM is determined here. Similar to the NCR, the minimal tissue contrast is used rather than the GM-WM contrast. To remove noise-driven variance, a Laplacian filter with a Dirichlet boundary condition is applied in the WMe area, resulting in a locally averaged image Cs, which was used to estimate the ICR:


\begin{eqnarray*}
{\mathrm{ICR}} = \beta (\sigma ({{C}_s}({\mathrm{WMe}}))/{{{\mathrm{c}}}_{{\mathrm{min}}}},0.2270,\ 1.3949)\quad \left( {{\mathrm{SE}}3} \right)
\end{eqnarray*}

Since most methods are able to correct strong inhomogeneities almost without loss of segmentation accuracy (e.g., approach X in Fig. 1B) [58], a weaker weighting was used. The worst BWP inhomogeneity level describes a grade C (see Fig. 3) that can be already measured in 3 Tesla data without protocol-based corrections.

### Resolution

The spatial resolution of MRI images plays an important role in obtaining meaningful representations of anatomical structures. For the general assessment of voxel volume and proportion in a single value, we used the RMS notation to define the RES:


\begin{eqnarray*}
{\mathrm{RES}} = \beta \left( {{{{\left( {\left( {{{{\mathrm{x}}}^2} + {{{\mathrm{y}}}^2} + {{{\mathrm{z}}}^2}} \right)/3} \right)}}^{1/2}},0.5,2.5} \right)\quad \left( {{\mathrm{SE}}4} \right)
\end{eqnarray*}

As a consequence of this definition, outliers with exceptionally low resolution in 1 of the 3 dimensions were weighted much higher than outliers with high resolution, resulting in an asymmetric evaluation where similar (isotropic) resolutions are preferred. The quality range was arbitrarily determined to characterize typical resolutions, with a simple scaling step size of 1 grade (10 rps) for another 0.5 mm, with 0.5 mm as an excellent result and 2.5 mm as the lowest-quality limit close to the average cortical thickness in humans.

For the principal evaluation, we tested RES by reducing and reinterpolating the tissue label map of the BWP to quantify the loss of information by Cohen’s κ [31]. RES yielded a higher Spearman’s correlation coefficient than the simple voxel mean RESM (ρ_RES_ = 0.994; ρ_RESM_ = 0.965; with RESM = *β*((x + y + z)/3, 0.5, 2.5). Although RES provides a good description of resolution under normal conditions, it has the major limitation that it does not quantify the true anatomical level of detail (i.e., how well fine structures are defined and how sharp the boundaries are).

The ECR is the average gradient ∇ of the GM/WM boundary (outlined by the segmentation and masked for extreme gradients, e.g., between CSF and WM and blood vessels) and normalized by the minimum tissue contrast and scaled similarly to the RES rating. It allows an evaluation of structural resolution independent of resampling or smoothing.


\begin{eqnarray*}
{\mathrm{ECR}} = \beta \left( {\nabla {\mathrm{WM }},0.0202,0.1003} \right)\ \quad \left( {{\mathrm{SE}}5} \right)
\end{eqnarray*}

**Figure 11:**
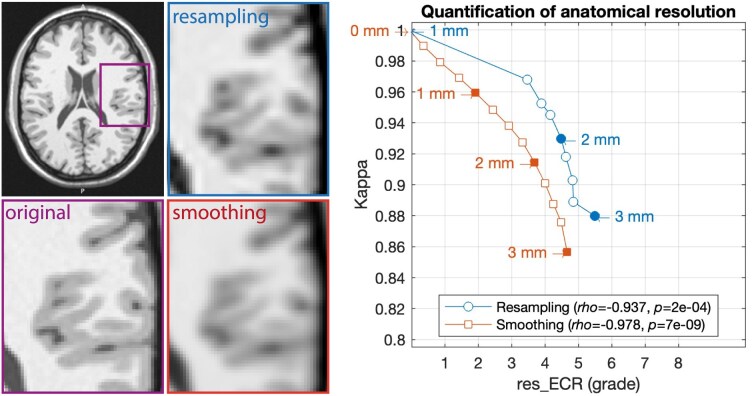
The effects of the simulated reduced resolution by resampling (downsampling to low resolution, followed by upsampling to original resolution) and Gaussian smoothing on the segmentation accuracy (quantified by κ) for the ECR resolution measure. Quantifying only by voxel resolution (RMS resolution score) would give the same value (grade 2 for 1-mm data) for all test cases (results for the BWP with 1% noise, 20% inhomogeneity field A, and isotropic 1-mm resolution). It is also obvious that there is a large step between the original resolution and the first resampled resolution, which describes the general information loss of data resampling and would be different in real data or if input data with a higher ground-truth resolution were used (i.e., test bias). In addition, quantification in low-resolution data (below 2 mm) becomes increasingly difficult due to the highly folded and thin cortical band (sampling theorem).

To test the quantification of anatomical rather than image resolution, spatial details were removed by resampling (downsampling to 1.25:0.25:3.00 mm and resampling to 1.00 mm) and smoothing (0:0.25:3.00 mm) a BWP image with 1% noise, 20% inhomogeneity of field A, and 1-mm resolution. The parameter test range was defined by the resolution of the BWP, the minimum smoothing resolution (0.2 mm for 1.0-mm data), and the average cortical thickness of 2.5 mm. The resulting images were then segmented to quantify changes using Cohen’s κ. In both cases, the final voxel resolution remains constant, so that the voxel-based RMS resolution measurements are identical even though the images become blurred and κ decreases (see Fig. 11). In contrast, our new ECR measure allows quantification of both test cases, although quantification of GM/WM edge strength and tissue contrast introduces further variance (*r* > 0.98, *P* < 7e-07).

However, there are several limitations of the measure itself, but also of the test design: (i) the BWP is limited in its anatomical details, supporting only 1-mm resolution with some partial volume effect; (ii) linear/spline resampling and smoothing affect the measures differently; and (iii) κ only quantifies segmentation accuracy, but not the quality of more complex surface reconstruction (e.g., Hausdorff distance to the gold standard surface) that could be used. Nevertheless, ECR already represents a significant step forward in quantifying image detail in real data.

### Surface topology

In order to approximate the surface topology in a reasonable time, the FEC was estimated at a resolution of 2 mm. The whole-brain WM surface was used rather than the typical neocortical hemispheres of most surface pipelines. To account for partial volume effects at the lower resolution, 2 WM surfaces were generated at thresholds of 0.25 and 0.75. As we observed more defects in children due to the thin developing WM structures, we used a maximum filter to extend and stabilize the surface creation.


\begin{eqnarray*}
{\mathrm{FEC}} = \beta \left( {{\mathrm{EC}},130,470} \right)\quad \left( {{\mathrm{SE}}6} \right)
\end{eqnarray*}

where the Euler characteristic (EC) is defined as EC = V − E + F, with V as number of vertices, E as the number of edges, and F as the number of faces of the created brain surface.

### Averaging

To obtain a meaningful composite measure of SIQR, we tested the mean, median, maximum, and 4 variants of the exponentially weighted averages with regard to their performance on the BWP and MR-ART dataset (Spearman’s correlation between tissue volume/κ and SIQR). To quantify the effect of interferences, we estimated the volume difference ΔV and the κ value to the artifact-free case (averaged across all tissue classes), where volume changes and κ statistics should be highly associated with the quality rating (Supplementary Table S2). We finally selected the power 4 function as it is more sensitive to outliers.

## Availability and Requirements

Project name: Quality Metrics of the Computational Anatomy Toolbox (CAT12)

Project homepage: https://neuro-jena.github.io/cat, https://github.com/ChristianGaser/cat12

Operating systems: Linux, Mac OS, Windows

Programming language: MATLAB

Other requirements: Statistical Parametric Mapping (SPM) https://www.fil.ion.ucl.ac.uk/spm/, https://github.com/spm

License: GNU GPL version 2 or higher

RRID:SCR_019184

Processing was done under Mac OS using MATLAB 2024a, SPM25, CAT12.8.2 R2166-R2890 (segmentation), and CAT12.9 R2890 (quality control).

## Availability of Supporting Source Code and Requirements

All additional supporting data and evaluation scripts are available in the *GigaScience* repository, GigaDB [30]. The framework is a part of the CAT12 toolbox [23, 24], which is part of SPM25 [26, 27].

Although SPM and CAT do not require additional toolboxes, the code used to generate the results and figures is available in the “catQC” subdirectory of CAT12 and the *GigaScience* repository and requires MATLAB 2024 or later with the “Statistics and Machine Learning Toolbox” and the “Curve Fitting Toolbox.”

Project name: Quality Metrics of the Computational Anatomy Toolbox (CAT12)

Project homepage: https://neuro-jena.github.io/cat, https://github.com/ChristianGaser/cat12

License: GPL-2.0 license

SciCrunch: RRID:SCR_019184

**System requirements**

Operating systems: Linux, Mac OS, (Windows)

Programming language: MATLAB

Package management: SPM25: https://www.fil.ion.ucl.ac.uk/spm/, https://github.com/spm

CAT12: https://neuro-jena.github.io/cat, https://github.com/ChristianGaser/cat12

**Hardware requirements:** Computer with more than 4 GB RAM that supports MATLAB

## Supplementary Material

giaf146_Supplemental_Files

giaf146_Authors_Response_To_Reviewer_Comments_Original_Submission

giaf146_Authors_Response_To_Reviewer_Comments_Revision_1

giaf146_Supplemental_File

giaf146_GIGA-D-25-00085_Original_Submission

giaf146_GIGA-D-25-00085_Revision_1

giaf146_GIGA-D-25-00085_Revision_2

giaf146_Reviewer_1_Report_Original_Submission Chris Foulon -- 5/3/2025

giaf146_Reviewer_2_Report_Revision_1 Oscar Esteban -- 9/16/2025

giaf146_Reviewer_4_Report_Revision_1 Laura Caquelin -- 9/24/2025

giaf146_Reviewer_4_Report_Revision_2 Laura Caquelin -- 11/7/2025

giaf146_Revision_2_Report_Original_Submission Oscar Esteban -- 5/5/2025

giaf146_Revision_3_Report_Original_Submission Cyril Pernet, PhD -- 5/11/2025

giaf146_Revision_4_Report_Original_Submission Laura Caquelin -- 6/10/2025

## Data Availability

MRI RAW data are available by the original providers given in Table 1. The ATLAS dataset V1.2 [40] is under controlled access, and users need to fill out an accessibility form to get the data [71]. The scan-rescan dataset is, apart from the selected scans, not publicly available but can be requested from Benjamin Thyreau from Tohoku University. All additional supporting data and evaluation scripts are available in the *GigaScience* repository, GigaDB [30]. The QC framework is a part of the CAT12 toolbox [23, 23], which is part of SPM25 [26, 27]. Although SPM and CAT do not require additional toolboxes, the code used to generate the results and figures (available in the “catQC” subdirectory of CAT12 and the *GigaScience* repository) requires MATLAB 2024 or later with “Statistics and Machine Learning Toolbox” and the “Curve Fitting Toolbox.”

## Additional Files

**Supplementary Fig. S1**. (A) Boxplots of different averaging functions on the expert grouping of the MR-ART dataset. Higher powers allow the outliers to be stronger and result in more appropriate ratings. (B) Higher powers also result in a more linear relationship between the image quality and segmentation quality κ (estimated for all scans with the motion artifacts of a subject in relation to their motion-free scan). (C) Pearson’s correlation plots of different combined ratings with different powers and the average volume loss (subplot 1 on the BWP and 2 on MR-ART) and κ loss (subplot 3 on the BWP and 4 on MR-ART) and for a simple mean of volume and κ loss in both cases (subplot 5). The black line includes all measures; the colored lines present all cases while leaving out one of the measures. The combination of *NCR+res_RMS* represented our classical IQR rating that was now extended by the edge-constrast ratio (res_ECR) and the fast Euler characteristic (FEC). In both cases, we observed that the inhomogeneity-to-contrast ratio (ICR) was contraindicative in describing the image quality, as higher scan quality is mostly driven by low noise and high resolution that are generally better in high-field scans, whereas the higher bias can be corrected quite well by state-of-the-art approaches such as SPM or CAT12.

**Supplementary Fig. S2**. We simulated the effects of segmentation accuracy for brain extraction problems (A) and for tissue over- and underestimation (B). Effects of skull-stripping problems are shown as lines in C, while the segmentation problems are shown as a scatterplot. Our quality scores are almost unaffected by severe skull-stripping problems (κ < 0.6), as even small regions are sufficient for global estimation. Tissue classification errors are more challenging, even if the reduced accuracy is quite small, because the contrast estimation is biased. In general, problematic cases tend to underestimate the image quality, which indirectly helps to identify serious preprocessing problems.

**Supplementary Fig. S3**. Changes in current quality scores (A) for the phantoms presented in Rusak et al. (2022) (B), which simulated neocortical atrophy of up to 1 mm in 20 subjects from the ADNI database, where a 0.01-mm cortical loss represents 1 year of healthy aging. As only the neocortical thickness is changed (C), similar outcomes in the measures are expected regardless of the simulated atrophy rate. However, the slightly different inhomogeneity and noise pattern in the CSF could introduce further bias in the evaluation.

**Supplementary Fig. S4**. Changes of all quality ratings (NCR, ICR, ECR, FEC, and SIQR) and the relative GM volume (rGMV) for aging (left), total intracranial volume (TIV, center), and sex (right).

**Supplementary Fig. S5**. Changes of all quality ratings (NCR, ICR, ECR, FEC, and SIQR) in aging (A) and grouped by the expert rating (B). (C) shows the ROC analysis of each quality rating to separate the expert rated motion groups. The change of the *relative GM volume* (**rGMV**) for aging is shown in (D).

**Supplementary Fig. S6**. Spearman’s correlation coefficient matrix representing the associations between CAT12 and MRIQC quality measures in the MR-ART dataset (Nárai et al. 2022), as well as age, sex, group0 (i.e., scan name/condition, e.g., instructed motion), group (final expert rating), relative CSF/GM/WM volume (rCSFV/rGMV/rWMV), ΔV (dvol_rel_CGW, volume change in relation to motion-free scan), and κ statistic (estimated to the motion-free scan and averaged over all tissues).

**Supplementary Fig. S7**. Example slice of the 4 **false-positive** cases from the MR-ART datasets that failed in the outlier detection in **no vs. severe artifacts**. They were classified by our rating as severe motion cases, whereas experts assessed them as motion-free. There was 1 false-negative case in the separation for no vs. severe artifacts.

**Supplementary Fig. S8**. (A) Example slices of the **false-negative** cases from the MR-ART dataset (35 of 42) that failed in the outlier detection in **no vs. slight/severe artifacts** and were classified by our measure as acceptable but failed in the expert grouping (EG) with a light (EG = 2) or severe (EG = 3) motion artifact. (B) Example slices with **false-positive** cases from the MR-ART dataset that failed in the outlier detection in **no vs. slight/severe artifacts** and were classified by our measure as unacceptable but as motion-free by the expert.

**Supplementary Fig. S9**. (A) Example slices with **false-negative cases** from the MR-ART datasets that failed in the outlier detection in **no/light vs. severe artifacts** and were classified by our measure as acceptable (no/light motion) but labeled as severe (EG = 3) by the experts. (B) Example slices with **false-positive** cases from the MR-ART dataset (35 of 47) that failed in the outlier detection in **no/light vs. severe artifacts** and were classified by our measure as unacceptable (severe motion) but as artifact free (EG = 1) or with slight motion artifact (EG = 2) by the expert.

**Supplementary Table S1**. Spearman correlation coefficients with p-values in the upper and lower part of the table, respectively.

**Supplementary Table S2**. Parameters of the full Tohoku dataset with the selected scans.

## Abbreviations

AUC: area under curve; BWP: Brain Web Phantom; CAP: Cortical Aging Phantom; CAP: Cortical Atrophy Phantom; CAT: Computational Anatomy Toolbox; CJV: coefficient of joint variation; CSF: cerebrospinal fluid; ECR: edge contrast ratio; FEC: full-brain Euler characteristic; FLAIR: fluid-attenuated inversion recovery; GM: gray matter; ICR: inhomogeneity contrast ratio; MRI: magnetic resonance imaging; NCR: noise contrast ratio; nSIQR: normalized structural image quality rating; QC: quality control; QM: quality measure; QR: quality rating; RES: resolution score; rGMV: relative gray matter volume; RMS: root mean square; RMSE: root mean square error; ROC: receiver operating characteristic; rps: rating points; SIQR: structural image quality rating; SPM: Statistical Parametric Mapping; TIV: total intracranial volume; TRT: Tohoku test–retest; WM: white matter.
