# Supplementary material for: Segmentation-based quality control of structural MRI using the CAT12 toolbox
Source: Gigascience. 2025 Nov 29;14:giaf146. doi: 10.1093/gigascience/giaf146 (PMC12758382; doi:10.1093/gigascience/giaf146)
Supplement: giaf146_Supplemental_Files [file giaf146_supplemental_files.zip › Fig 7 to fig 9, Table s2.pdf]

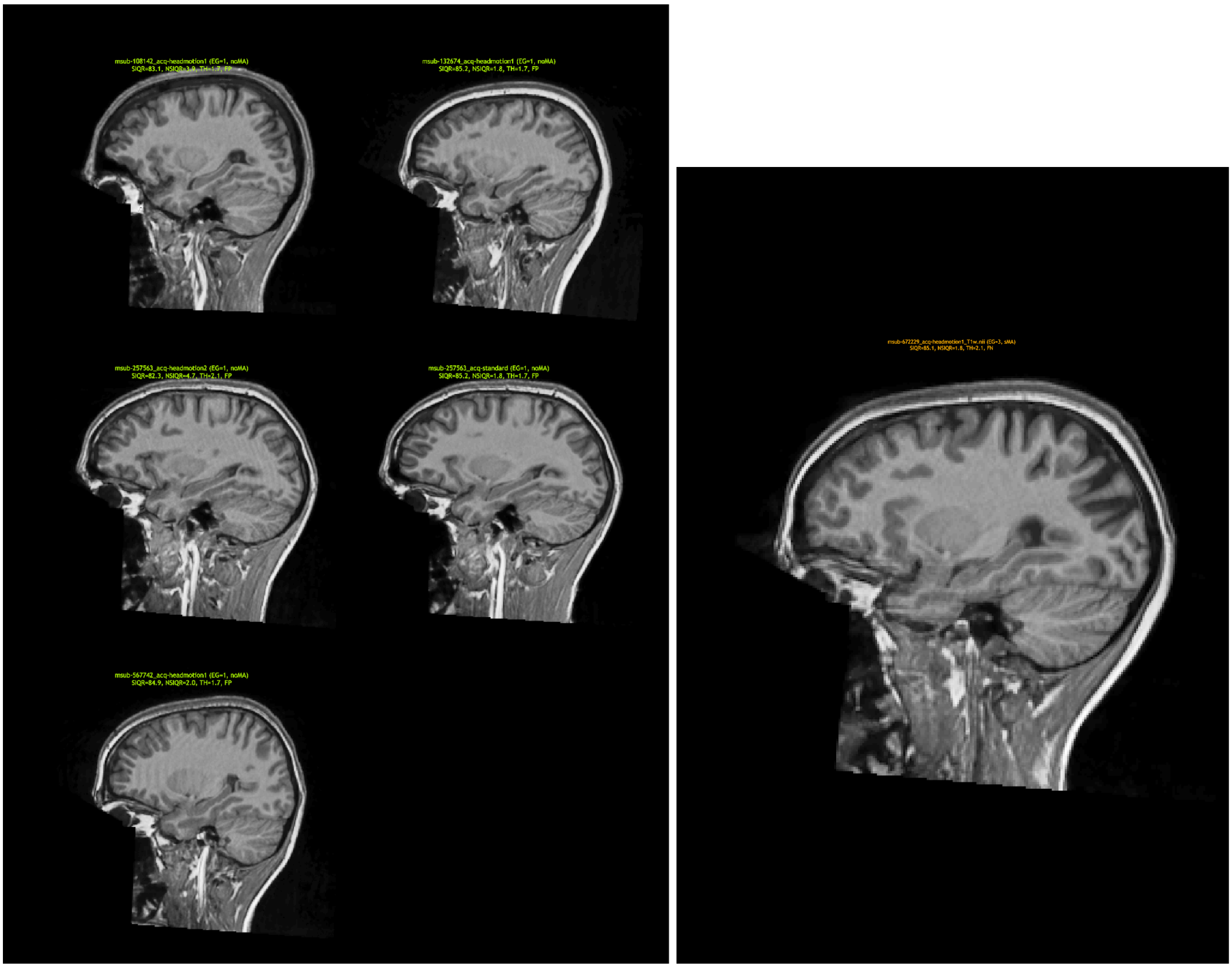

Figure S7: Example slice of the four **false-positive** cases from the MR-ART datasets that failed in the outlier detection between **no vs. severe artifacts.** They were classified by our rating as severe motion cases, whereas experts assessed them as motion-free. There were one false-negative cases in the separation between no vs. severe artifacts.

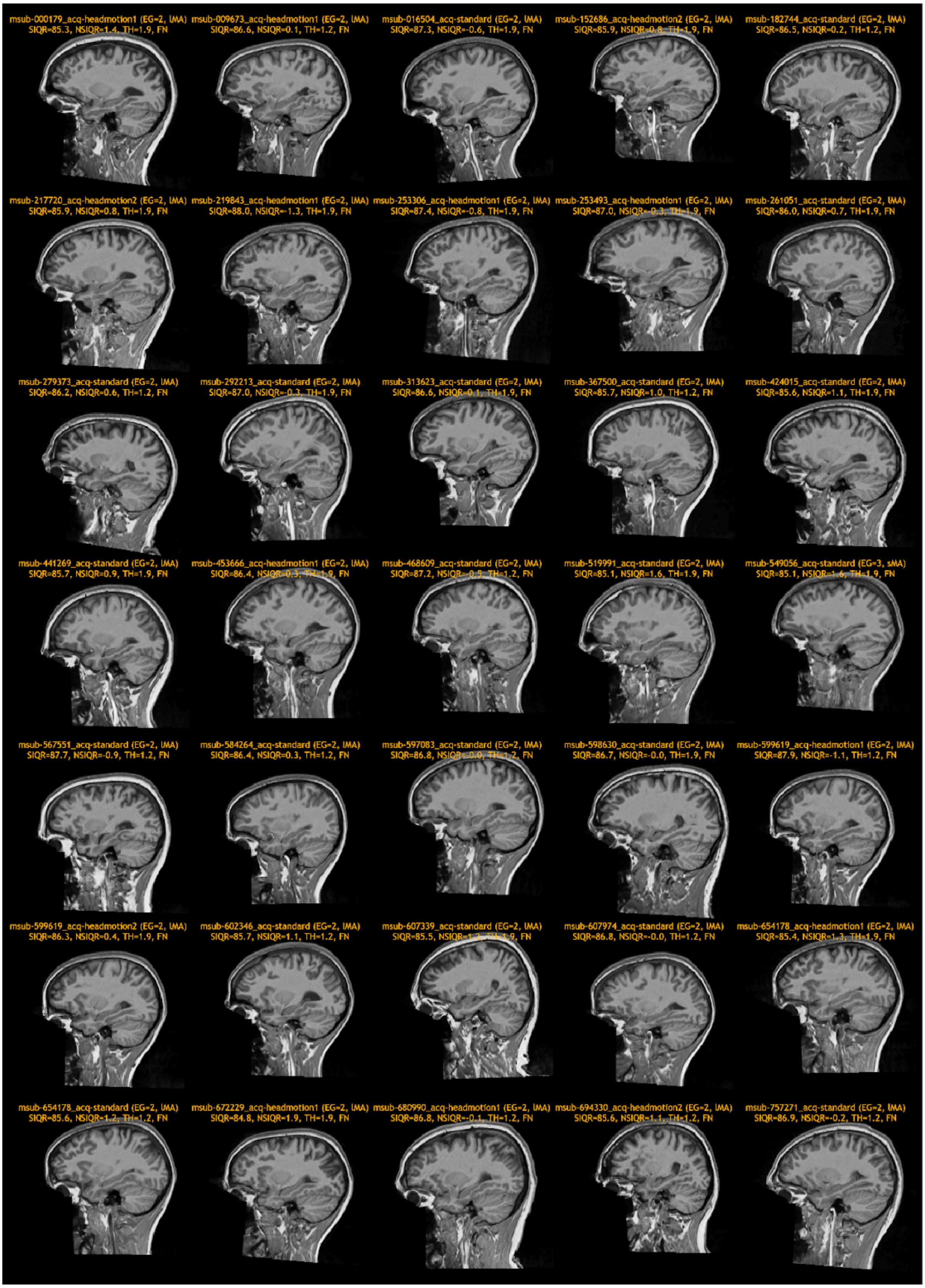

Figure S8a: Example slices of the **false-negative** cases from the MR-ART dataset (35 of 42) that failed in the outlier detection between **no vs. slight/severe artifacts** and were classified by our measure as acceptable, but failed in the expert grouping (EG) with a light (EG=2) or severe (EG=3) motion artifact rating.

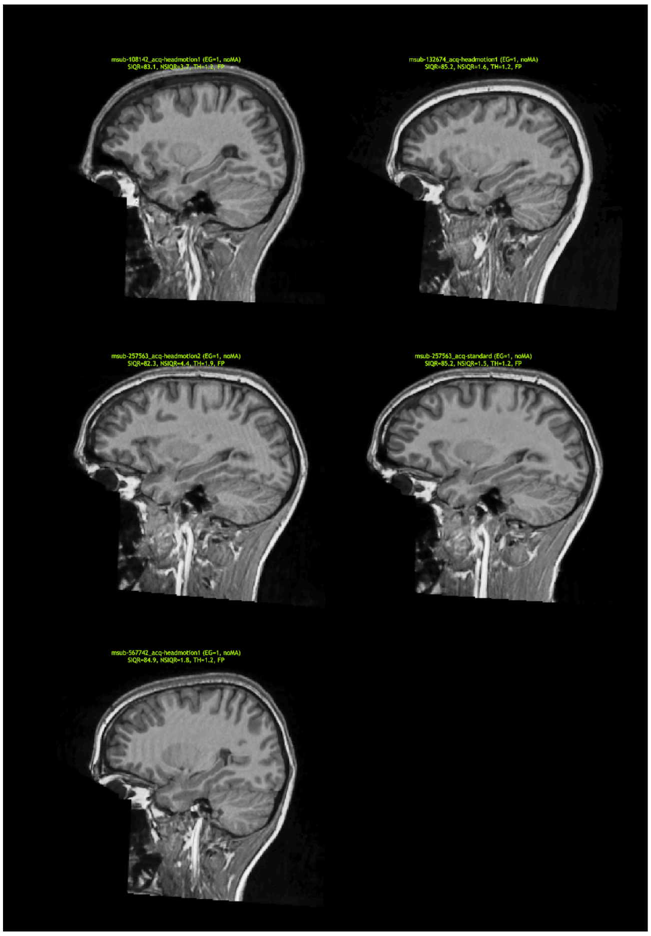

Figure S8b: Example slices with **false-positive** cases from the MR-ART dataset that failed in the outlier detection between **no vs. slight/severe artifacts** and were classified by our measure as unacceptable, but as motion-free by the expert.

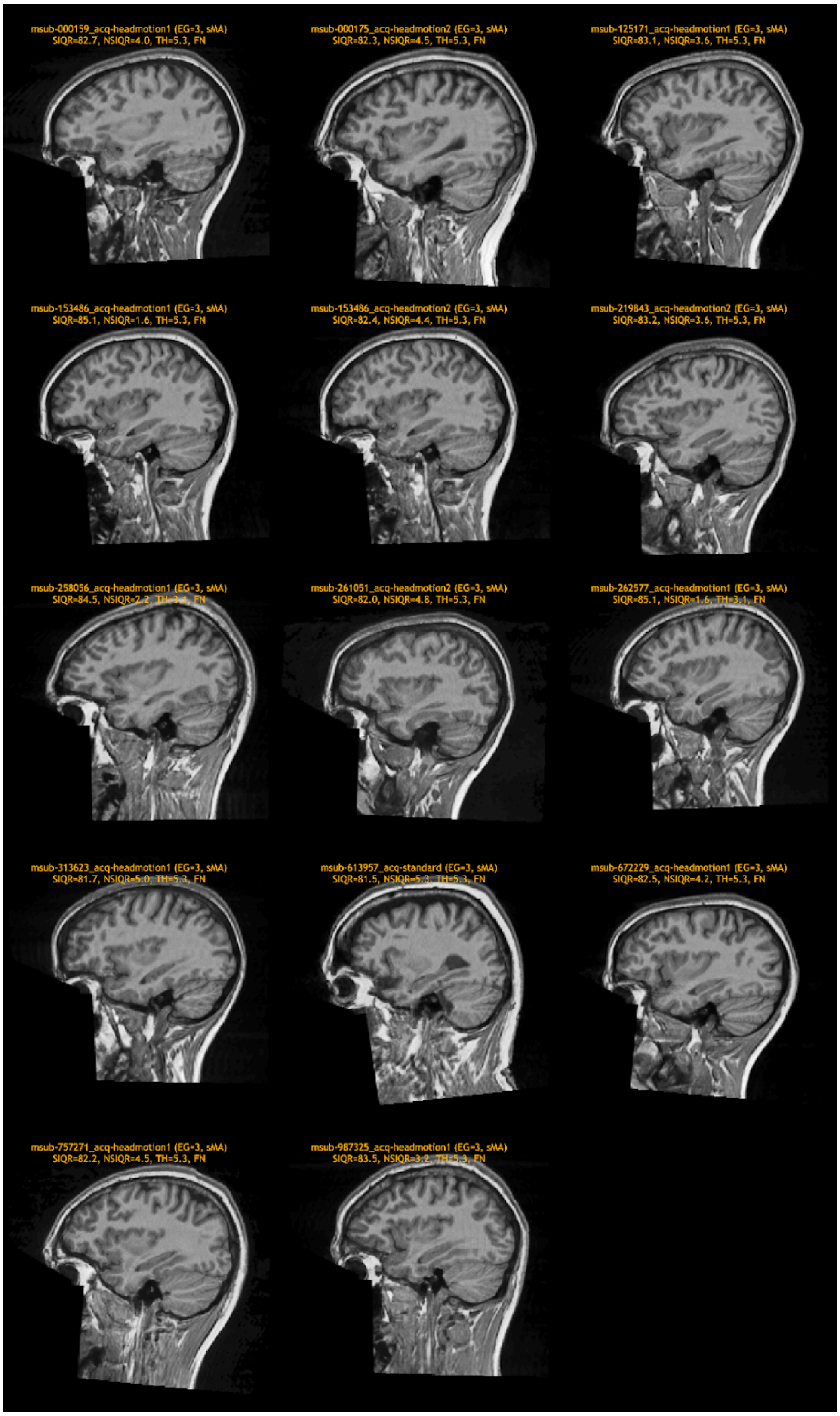

Figure S9a: Example slices with **false-negative cases** from the MR-ART datasets that failed in the outlier detection between **no/light vs. severe artifacts** and were classified by our measure as acceptable (no/light motion) but labeled as severe (EG=3) rating by the experts.

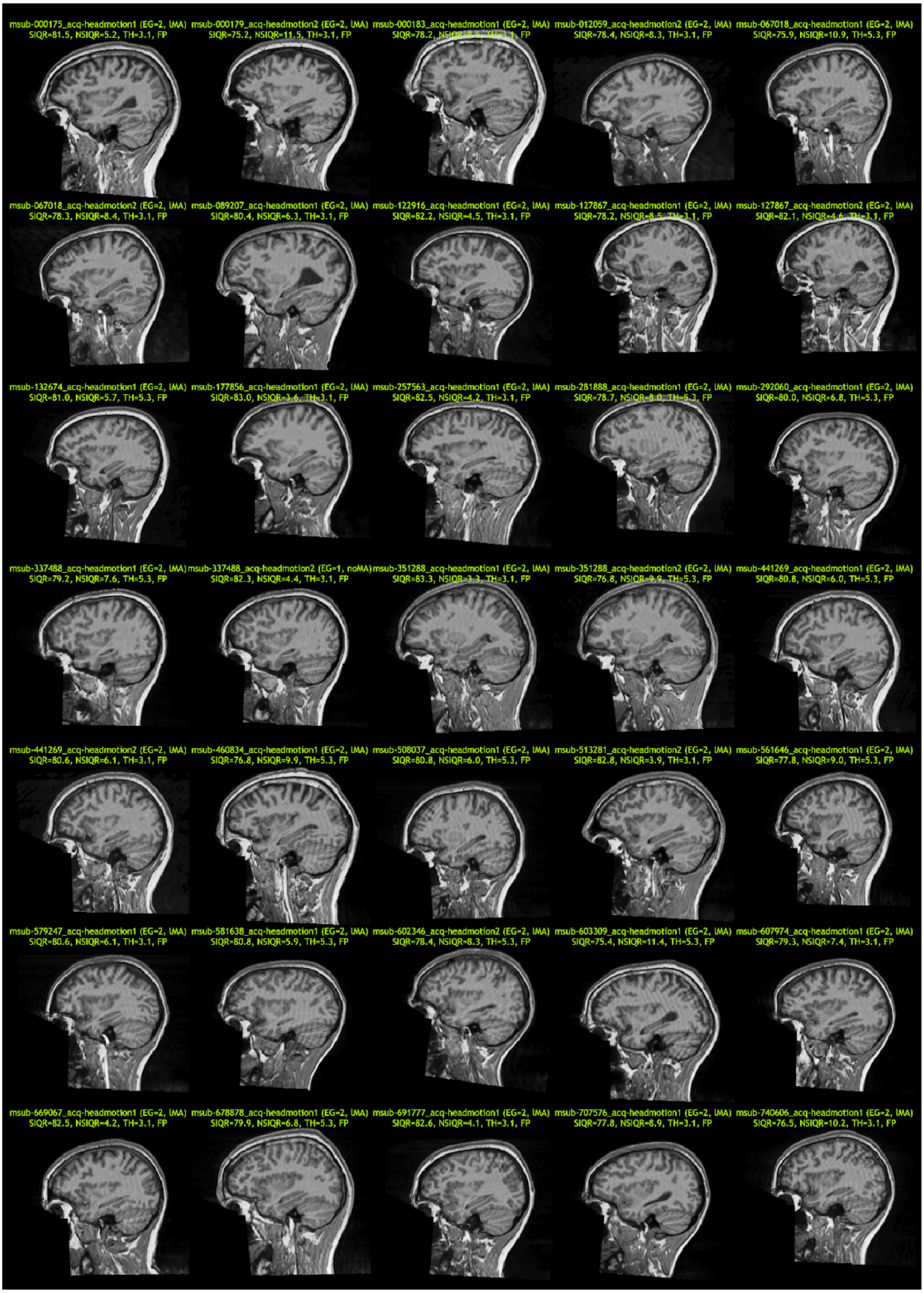

Figure S9b: Example slices with **false-positive** cases from the MR-ART dataset (35 of 47) that failed in the outlier detection between **no/light vs. severe artifacts** and were classified by our measure as unacceptable (severe motion) but as artifact free (EG=1) or with slight motion artifact (EG=2) by the expert.

Table S2: Parameters of the full Tohoku dataset with the selected scans.

mprage	orientation	SENSE	TFE	TI	voxdim0	voxdim1	voxdim2	durationSec
20120701_112011MPRAGE10SENSEs1401a1014.nii	A	2;1;1	1800	845	1	1	1	295
20120701_112011MPRAGE10SENSEs2601a1026.nii	A	2;1;1	1800	845	1	1	1	295
20120701_112011MPRAGE1SENSEs1001a1010.nii	A	1.5;1;1	2100	812	1	1	1	369
20120701_112011MPRAGE1SENSEs1701a1017.nii	A	1.5;1;1	2100	812	1	1	1	369
20120701_112011MPRAGE2SENSEs1101a1011.nii	A	2;1;1	2100	812	1	1	1	276
20120701_112011MPRAGE2SENSEs1801a1018.nii	A	2;1;1	2100	812	1	1	1	276
20120701_112011MPRAGE3SENSEs1901a1019.nii	A	1.5;1;1	1850	711	1	1	1	197
20120701_112011MPRAGE3SENSEs301a1003.nii	A	1.5;1;1	1850	711	1	1	1	197
20120701_112011MPRAGE4SENSEs2001a1020.nii	A	2;1;1	1850	711	1	1	1	147
20120701_112011MPRAGE4SENSEs401a1004.nii	A	2;1;1	1850	711	1	1	1	147
20120701_112011MPRAGE5SENSEs2101a1021.nii	A	1.5;1;1	1500	711	1	1	1	160
20120701_112011MPRAGE5SENSEs501a1005.nii	A	1.5;1;1	1500	711	1	1	1	160
20120701_112011MPRAGE6SENSEs2201a1022.nii	A	2;1;1	1500	711	1	1	1	119
20120701_112011MPRAGE6SENSEs601a1006.nii	A	2;1;1	1500	711	1	1	1	119
20120701_112011MPRAGE7SENSEs2301a1023.nii	A	2;1;1	1500	575	2	2	1	83
20120701_112011MPRAGE7SENSEs701a1007.nii	A	2;1;1	1500	575	2	2	1	83
20120701_112011MPRAGE8SENSEs2401a1024.nii	A	2;1;1	2100	575	2	2	1	116
20120701_112011MPRAGE8SENSEs801a1008.nii	A	2;1;1	2100	575	2	2	1	116
20120701_112011MPRAGE9SENSEs2501a1025.nii	A	2;1;1	2100	711	1	1	1	167
20120701_112011MPRAGE9SENSEs901a1009.nii	A	2;1;1	2100	711	1	1	1	167
20120707_124735MPRAGE14SENSEs3401a1034.nii	A	2;1;1	1800	811	1	1	1	237
20120707_124735MPRAGE14SENSEs501a1005.nii	A	2;1;1	1800	811	1	1	1	237
20120707_124735MPRAGE14SENSEs901a1009.nii	A	2;1;1	1800	811	1	1	1	237
20120707_124735MPRAGE15SENSEs601a1006.nii	A	2.5;1;1	1800	866	1	1	1	190
20120707_124735MPRAGE17SENSEs1001a1010.nii	A	1.5;1;1	1800	710	1	1	1	239
20120707_124735MPRAGE18SENSEs1101a1011.nii	A	1.5;1;1	1800	865	1	1	1	316
20120707_124735MPRAGE18SENSEs3601a1036.nii	A	1.5;1;1	1800	865	1	1	1	316
20120707_124735MPRAGE19SENSEs1201a1012.nii	A	1.5;1;1	2500	726	1	1	1	331
20120707_124735MPRAGE1woSENSEs3501a1035.nii	A	1;1;1	2500	695	1	1	1	481
20120707_124735MPRAGE20SENSEs1301a1013.nii	A	2;1;1	1800	850	1	1	1	237
20120707_124735MPRAGE21SENSEs3801a1038.nii	A	1.5;1;1	1800	798	1	1	1	239
20120707_124735MPRAGE22SENSEs3701a1037.nii	A	1.5;1;1	1800	863	1	1	1	316
20120707_124735MPRAGEdcnTr1800CLEARs3301a1033.nii	A	1;1;1	1800	715	1	1	1	347
20120707_124735veryshortMPRAGESENSEs2901a1029.nii	A	4.5;1;1	784	402	1	1	2	36
20120707_124735veryshortMPRAGESENSEs3901a1039.nii	A	4.5;1;1	784	402	1	1	2	36
20120725_115520veryshortMPRAGESENSEs1001a1010.nii	A	4.5;1;1	784	402	1	1	2	36
20120725_115520veryshortMPRAGESENSEs401a1004.nii	A	4.5;1;1	784	402	1	1	2	36
20120729_114553veryshortMPRAGESENSEs301a1003.nii	A	4.5;1;1	784	402	1	1	2	36
20120729_114553veryshortMPRAGESENSEs801a1008.nii	A	4.5;1;1	784	402	1	1	2	36
20120802_113755MPRAGE22SENSEs401a1004.nii	A	2;1;1	1700	863	0	0	1	353
20120802_113755MPRAGE23SENSEs901a1009.nii	A	2;1;1	1700	842	1	1	1	581
20120802_113755MPRAGE24SENSEs1201a1012.nii	A	1;1;1	1600	808	1	1	1	611
20120802_113755MPRAGE25SENSEs1501a1015.nii	A	3;1;1	1505	764	1	1	1	299
20120808_113817MPRAGE26SENSEs1101a1011.nii	A	2.5;1;1	1750	867	1	1	1	491
20120808_113817shorterMPRAGESENSEs901a1009.nii	A	4;1;1	1113	572	1	1	2	53
20120808_113817veryshortMPRAGESENSEs301a1003.nii	A	4.5;1;1	784	402	1	1	2	36
20120811_153828MPRAGE26SENSEs501a1005.nii	A	2.5;1;1	1750	867	1	1	1	491
20120811_153828MPRAGEdcnTr1800CLEARs601a1006.nii	A	1;1;1	1800	715	1	1	1	347
20120822_115825MPRAGE18SENSEs501a1005.nii	A	1.5;1;1	1800	865	1	1	1	316
20120822_115825MPRAGE27SENSEs1001a1010.nii	S	2;1;1.5	1800	883	1	1	1	237
20120822_115825MPRAGE28SENSEs801a1008.nii	S	1.5;1;1.5	2200	1077	1	1	1	422
20120822_115825MPRAGE29SENSEs1101a1011.nii	S	2;1;1.5	1800	887	1	1	1	190
20120902_164438MPRAGE14ti1kSENSEs601a1006.nii	A	2;1;1	1800	1000	1	1	1	237
20120902_164438MPRAGE18ti960SENSEs701a1007.nii	A	1.5;1;1	1800	950	1	1	1	316
20120914_182400MPRAGEasVISTA28SENSEs501a1005.nii	S	2;1;2	2200	612	1	1	1	282
20120920_182515MPRAGEasVISTA28TI1600SENSEs401a1004.nii	S	2;1;2	2200	1600	1	1	1	283
20120924_174146MPRAGEasVISTA28TFE1600TI1200SEs1001a1010.nii	S	2;1;2	1800	1200	1	1	1	232
20120926_181803shorterMPRAGE1200TISENSEs801a1008.nii	A	4;1;1			1	1	2	53
20120930_112053MPRAGEasV10SENSEs2901a1029.nii	S	2;1;2	2200	900	1	1	1	283
20120930_112053MPRAGEasV11SENSEs3201a1032.nii	S	2;1;2	1700	900	1	1	1	219
20120930_112053MPRAGEasV12SENSEs3401a1034.nii	S	2;1;2	1400	800	1	1	1	180
20120930_112053MPRAGEasV13SENSEs2801a1028.nii	S	2;1;2	1300	750	1	1	1	167
20120930_112053MPRAGEasV1SENSEs1901a1019.nii	S	2;1;2	1900	1300	1	1	1	245
20120930_112053MPRAGEasV1SENSEs301a1003.nii	S	2;1;2	1900	1300	1	1	1	245
20120930_112053MPRAGEasV2SENSEs401a1004.nii	S	2;1;2	2200	1300	1	1	1	283
20120930_112053MPRAGEasV3SENSEs501a1005.nii	S	2;1;2	2600	1300	1	1	1	334

20120930_112053MPRAGEasV4SENSEs601a1006.nii	S	2;1;2	2200	1200	1	1	1	283
20120930_112053MPRAGEasV5SENSEs701a1007.nii	S	2;1;2	2200	1400	1	1	1	283
20120930_112053MPRAGEasV6SENSEs801a1008.nii	S	2;1;2	2200	1600	1	1	1	283
20120930_112053MPRAGEasV7SENSEs2401a1024.nii	S	2;1;2	1900	1100	1	1	1	244
20120930_112053MPRAGEasV7SENSEs3001a1030.nii	S	2;1;2	1900	1100	1	1	1	244
20120930_112053MPRAGEasV8SENSEs2501a1025.nii	S	2;1;2	1900	900	1	1	1	244
20120930_112053MPRAGEasV8SENSEs3301a1033.nii	S	2;1;2	1900	900	1	1	1	244
20120930_112053MPRAGEasV9SENSEs3101a1031.nii	S	2;1;2	1900	700	1	1	1	244
20121006_143435MPGEsagml1SENSEs301a1003.nii	S	2;1;2	1800	900	1	1	1	208
20121006_143435MPGEsagml2SENSEs401a1004.nii	S	2;1;2	1400	900	1	1	1	162
20121006_143435MPGEsagml3SENSEs501a1005.nii	S	2;1;2	2100	900	1	1	1	242
20121006_143435MPRAGE30CLEARs901a1009.nii	A	1;1;1	1800	900	1	1	1	347
20121006_143435MPRAGE31SENSEs1001a1010.nii	A	2;1;1	1800	900	1	1	1	179
20121006_143435MPRAGE32SENSEs1101a1011.nii	A	1.5;1;1	1600	900	1	1	1	212
20121014_121738MPGEsagml1SENSEs1201a1012.nii	S	2;1;2	1800	900	1	1	1	208
20121014_121738MPGEsagml1SENSEs301a1003.nii	A	2;1;2	1800	900	1	1	1	208
20121014_121738MPGEsagml2SENSEs1301a1013.nii	S	2;1;2	1400	900	1	1	1	162
20121014_121738MPGEsagml2SENSEs401a1004.nii	A	2;1;2	1400	900	1	1	1	162
20121014_121738MPGEsagml3SENSEs1401a1014.nii	S	2;1;2	2100	900	1	1	1	242
20121014_121738MPGEsagml3SENSEs501a1005.nii	A	2;1;2	2100	900	1	1	1	242
20121014_121738MPGEsagml4SENSEs1501a1015.nii	S	2;1;2	1800	1200	1	1	1	208
20121014_121738MPGEsagml4SENSEs601a1006.nii	A	2;1;2	1800	1200	1	1	1	208
20121020_132933MPGEsagml1SENSEs601a1006.nii	S	2;1;2	1800	900	1	1	1	208
20121020_132933MPGEsagml4SENSEs501a1005.nii	S	2;1;2	1800	1200	1	1	1	208
20121020_132933MPGEsagml5SENSEs901a1009.nii	A	2;1;2	1800	1300	1	1	1	208
20121020_132933MPRAGE1woSENSEs301a1003.nii	A	1;1;1	2500	713	1	1	1	481
20121020_132933MPRAGEasV11SENSEs701a1007.nii	S	2;1;2	1700	900	1	1	1	219
20121020_132933MPRAGEasV14SENSEs801a1008.nii	S	1;1;2	1700	900	1	1	1	428
20121028_122334MPGEsagml3SENSEs701a1007.nii	S	2;1;2	2100	900	1	1	1	242
20121028_122334MPGEsagml4SENSEs501a1005.nii	S	2;1;2	1800	1200	1	1	1	208
20121028_122334MPGEsagml6SENSEs301a1003.nii	S	2;1;2	2500	750	1	1	1	288
20121028_122334MPRAGE1woSENSEs601a1006.nii	A	1;1;1	2500	750	1	1	1	481
20121029_151839MPGEsagml1SENSEs501a1005.nii	S	2;1;2	1800	900	1	1	1	208
20121029_151839MPGEsagml2SENSEs301a1003.nii	S	2;1;2	1400	900	1	1	1	162
20121029_151839MPGEsagml3SENSEs701a1007.nii	S	2;1;2	2100	900	1	1	1	242
20121029_151839MPGEsagml7SENSEs601a1006.nii	S	1.5;1;1.5	1800	800	1	1	1	278
20121029_151839MPGEsagml8SENSEs401a1004.nii	S	2;1;2	1400	750	1	1	1	162
20121029_151839MPRAGE14SENSEs901a1009.nii	A	2;1;1	1800	800	1	1	1	237
20121029_151839MPRAGE33SENSEs801a1008.nii	A	1;1;1	1800	800	1	1	1	462
20121118_091051MPRAGE1woSENSEs301a1003.nii	A	1;1;1	2500	695	1	1	1	481
20121118_091051MPRAGEdcnSENSESENSEs401a1004.nii	A	2;1;1	2500	695	1	1	1	248
20121118_091051MPRAGE1woSENSEGRregularCLEs501a1005.nii	A	1;1;1	2500	1140	1	1	1	482
20121118_091051MPRAGEdcnSENSEGRregularCLEARs601a1006.nii	A	1;1;1	2500	1140	1	1	1	482
20121118_091051MPGEsagml1SENSEs701a1007.nii	S	2;1;2	1800	900	1	1	1	208
20121118_091051MPGEsagml8SENSEs801a1008.nii	S	2;1;2	1400	750	1	1	1	162
20121118_091051MPRAGEasV12SENSEs901a1009.nii	S	2;1;2	1400	800	1	1	1	180
20130211_091630MPGEsagml084SENSEs1101a1011.nii	S	2;1;2	1800	800	1	1	1	237
20130211_091630MPGEsagml099SENSEs1301a1013.nii	S	2;1;2	1800	800	1	1	1	208
20130211_091630MPGEsagml09SENSEs1201a1012.nii	S	2;1;2	1800	800	1	1	1	222
20130211_091630MPGEsagml1NSA2SENSEs601a1006.nii	S	2;1;2	1800	900	1	1	1	415
20130211_091630MPGEsagml1SENSEs501a1005.nii	S	2;1;2	1800	700	1	1	1	208
20130211_091630MPGEsagml1TI700SENSEs801a1008.nii	S	2;1;2	1800	700	1	1	1	208
20130211_091630MPGEsagml35SENSEs301a1003.nii	S	1.5;1;2	2100	900	1	1	1	324
20130211_091630MPGEsameConDcnSENSEs401a1004.nii	S	1.5;1;1.25	2500	706	1	1	1	331
20130211_091630MPRAGE1woSENSEs701a1007.nii	S	1;1;1	2500	706	1	1	1	481
20130213_192623MPGEsagml9SENSEs301a1003.nii	S	1.25;1;1.25	1800	880	1	1	1	379
20130219_183335MPGEsagml842SENSEs401a1004.nii	S	2;1;2	2100	750	1	1	1	276
20130219_183335MPRAGE2SENSEs501a1005.nii	A	2;1;2	2100	800	1	1	1	276
