# Supplementary material for: Segmentation-based quality control of structural MRI using the CAT12 toolbox
Source: Gigascience. 2025 Nov 29;14:giaf146. doi: 10.1093/gigascience/giaf146 (PMC12758382; doi:10.1093/gigascience/giaf146)
Supplement: giaf146_Supplemental_Files [file giaf146_supplemental_files.zip › figS2.pdf]

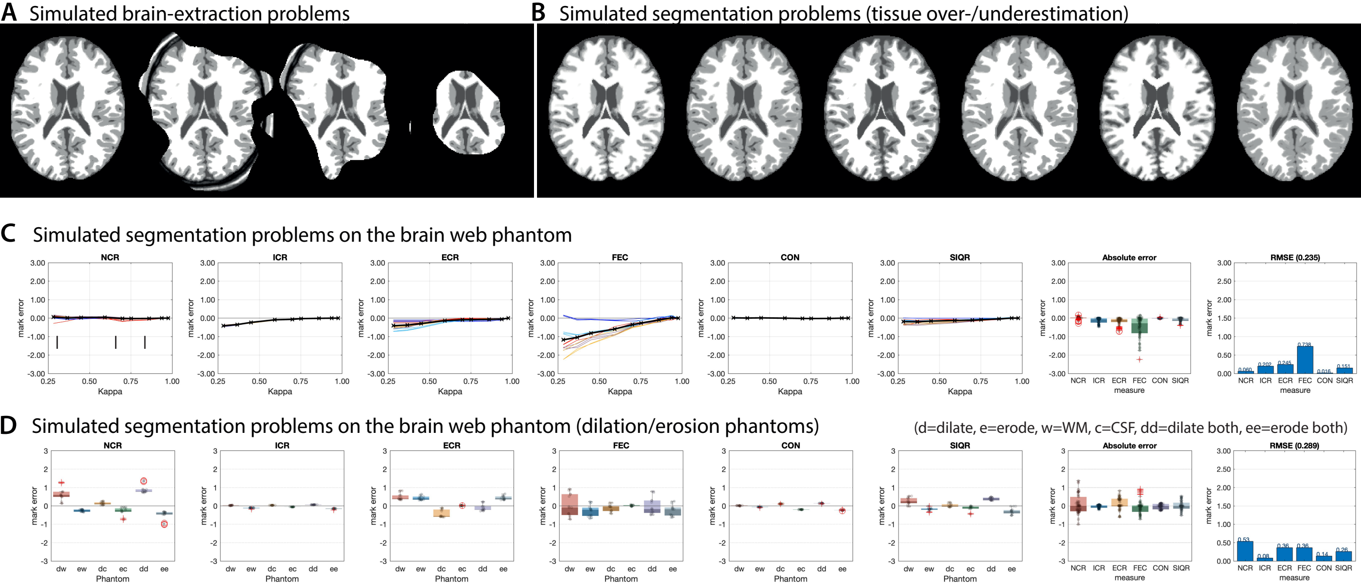
A Simulated brain-extraction problems
B Simulated segmentation problems (tissue over-/underestimation)
C Simulated segmentation problems on the brain web phantom
NCR
ICR
ECR
FEC
CON
SIQR
Absolute error
RMSE (0.235)
mark error
Kappa
measure
0.060
0.202
0.245
0.738
0.016
0.151
D Simulated segmentation problems on the brain web phantom (dilation/erosion phantoms)
(d=dilate, e=erode, w=WM, c=CSF, dd=dilate both, ee=erode both)
RMSE (0.289)
dw
ew
dc
ec
dd
ee
Phantom
0.53
0.08
0.36
0.36
0.14
0.26
