# Supplementary material for: Segmentation-based quality control of structural MRI using the CAT12 toolbox
Source: Gigascience. 2025 Nov 29;14:giaf146. doi: 10.1093/gigascience/giaf146 (PMC12758382; doi:10.1093/gigascience/giaf146)
Supplement: giaf146_Supplemental_Files [file giaf146_supplemental_files.zip › tabS1.pdf]

p/rho	noise	bias	rRMS	NCR	ICR	RES	ECR	FEC	IQR	SIQR	Kappa	rCSFV	rGMV	rWMV
noise		0.031	-0.008	**0.976**	-0.086	-0.004	0.631	**0.979**	0.897	**0.941**	-0.857	-0.716	-0.705	0.851
bias	6E-01		0.015	-0.088	**0.961**	0.007	0.158	0.009	-0.087	-0.019	-0.034	-0.190	0.104	0.018
rRMS	9E-01	8E-01		-0.012	0.028	0.332	**0.585**	0.004	0.167	0.173	-0.413	-0.003	-0.288	0.220
NCR	5E-226	1E-01	8E-01		-0.175	-0.118	0.596	0.977	0.875	0.923	-0.819	-0.667	-0.730	0.851
ICR	1E-01	8E-191	6E-01	1E-03		-0.118	0.146	-0.091	-0.213	-0.134	0.046	-0.104	0.085	-0.009
RES	9E-01	9E-01	3E-10	3E-02	3E-02		0.001	-0.050	0.223	0.143	-0.124	0.152	0.259	-0.229
ECR	6E-39	3E-03	1E-32	5E-34	7E-03	1E+00		0.612	0.548	0.632	-0.845	-0.505	-0.837	0.836
FEC	1E-234	9E-01	9E-01	5E-229	9E-02	4E-01	3E-36		0.901	0.950	-0.850	-0.722	-0.697	0.849
IQR	2E-121	1E-01	2E-03	4E-108	8E-05	4E-05	6E-28	0.000		0.979	-0.889	-0.717	-0.569	0.735
SIQR	5E-160	7E-01	1E-03	8E-142	1E-02	8E-03	3E-39	3E-172	0.000		-0.916	-0.729	-0.647	0.805
Kappa	4E-99	5E-01	2E-15	2E-83	4E-01	2E-02	1E-93	1E-95	0.000	1E-135		0.733	0.780	-0.893
rCSFV	2E-54	4E-04	1E+00	6E-45	6E-02	5E-03	3E-23	9E-56	0.000	2E-57	2E-58		0.428	-0.700
rGMV	3E-52	5E-02	7E-08	1E-57	1E-01	1E-06	3E-90	1E-50	0.000	1E-41	2E-70	2E-16		-0.924
rWMV	3E-96	7E-01	4E-05	3E-96	9E-01	2E-05	7E-90	3E-95	0.000	3E-78	5E-119	4E-51	2E-142	
