# Supplementary figures and images for: Segmentation-based quality control of structural MRI using the CAT12 toolbox
Source: Gigascience. 2025 Nov 29;14:giaf146. doi: 10.1093/gigascience/giaf146 (PMC12758382; doi:10.1093/gigascience/giaf146)

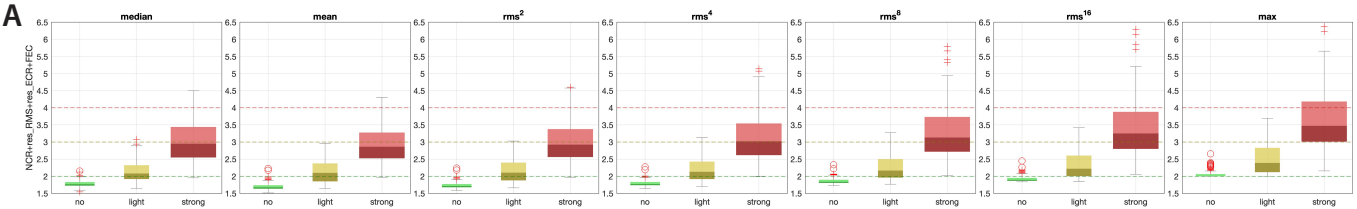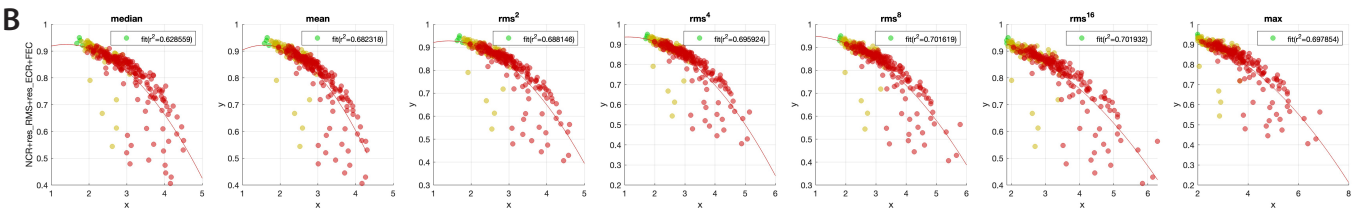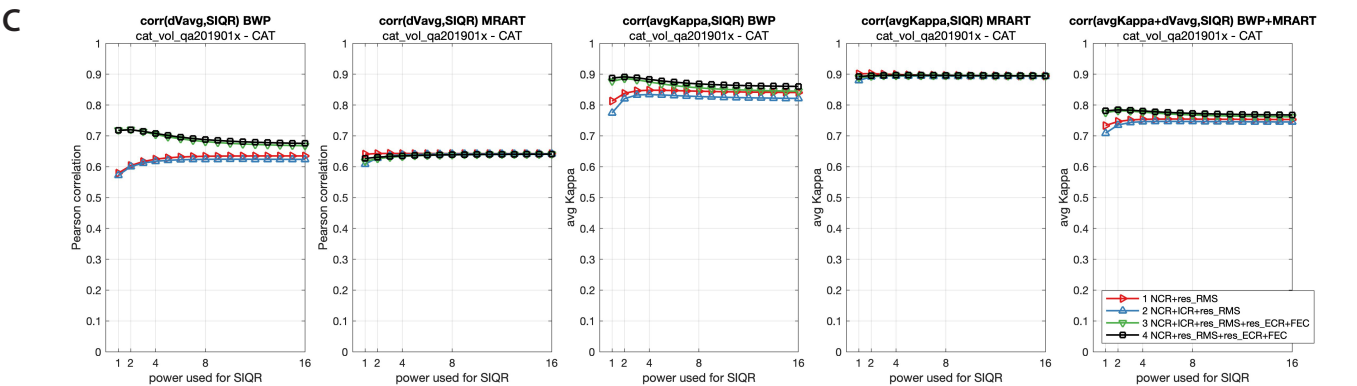

Supplement: giaf146_Supplemental_Files [file giaf146_supplemental_files.zip › figS1.pdf]

**A**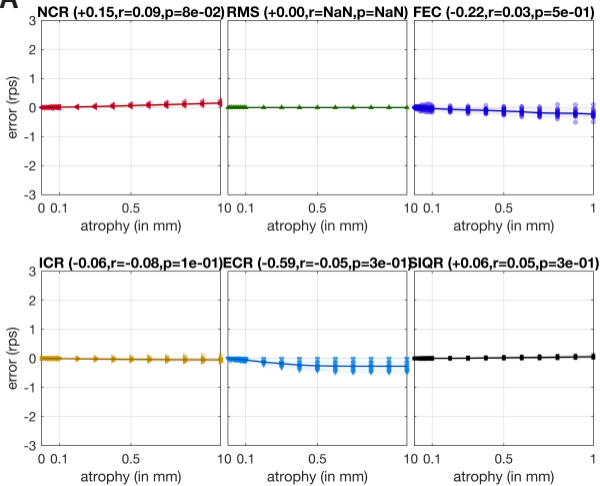**B**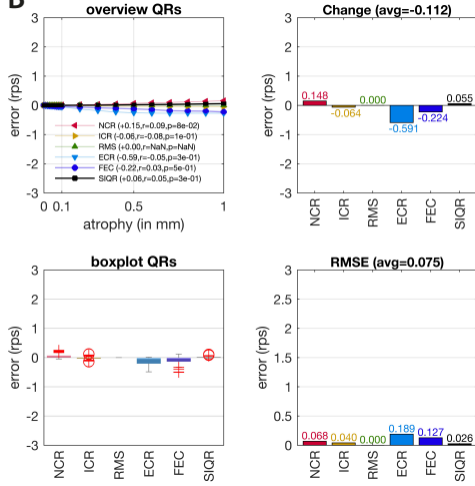**C**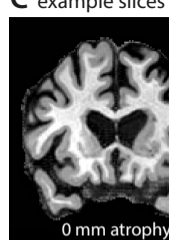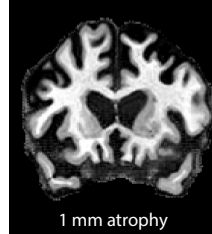

Supplement: giaf146_Supplemental_Files [file giaf146_supplemental_files.zip › figS3.pdf]

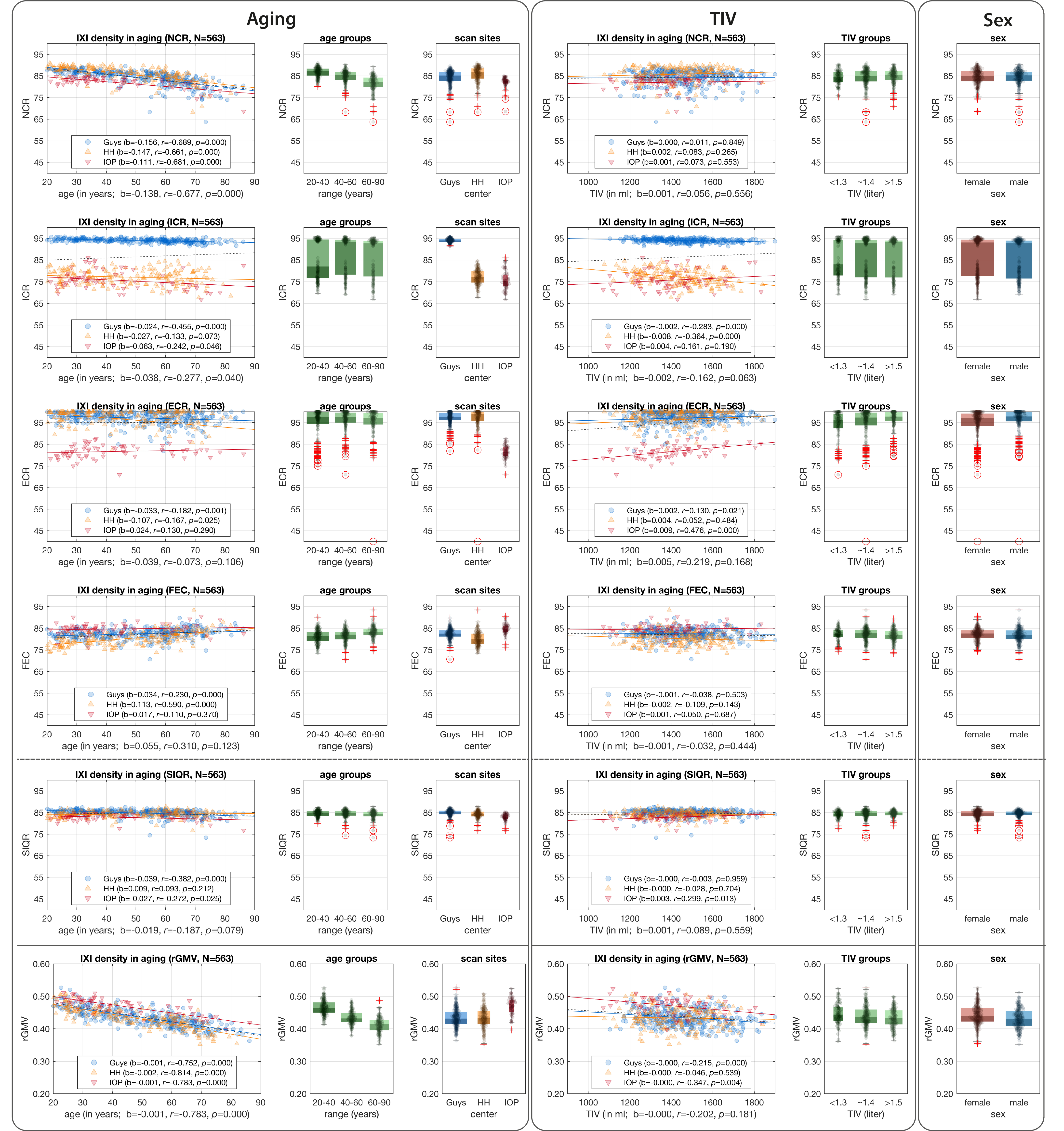

Supplement: giaf146_Supplemental_Files [file giaf146_supplemental_files.zip › figS4.png]

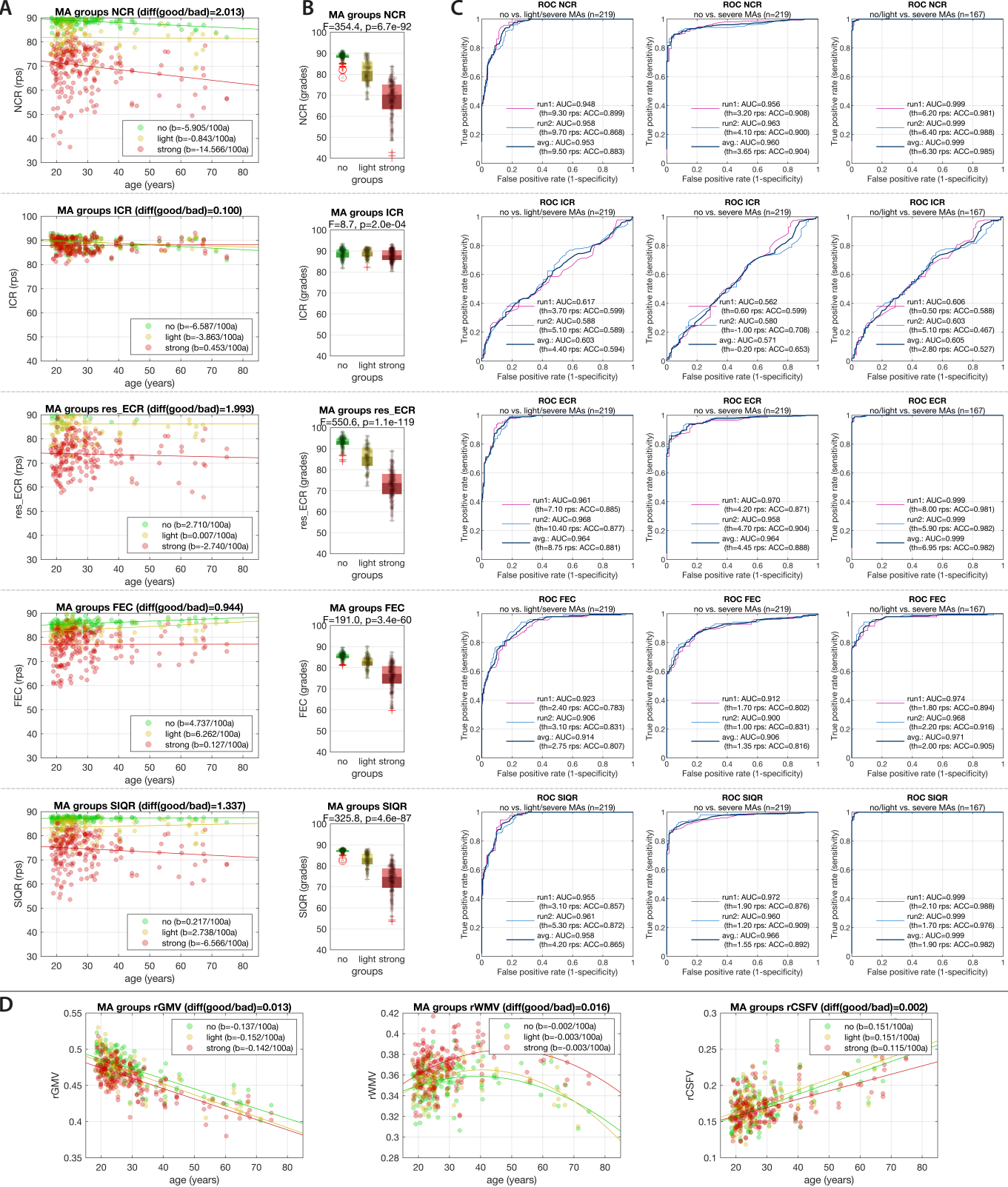

Supplement: giaf146_Supplemental_Files [file giaf146_supplemental_files.zip › figS5.pdf]

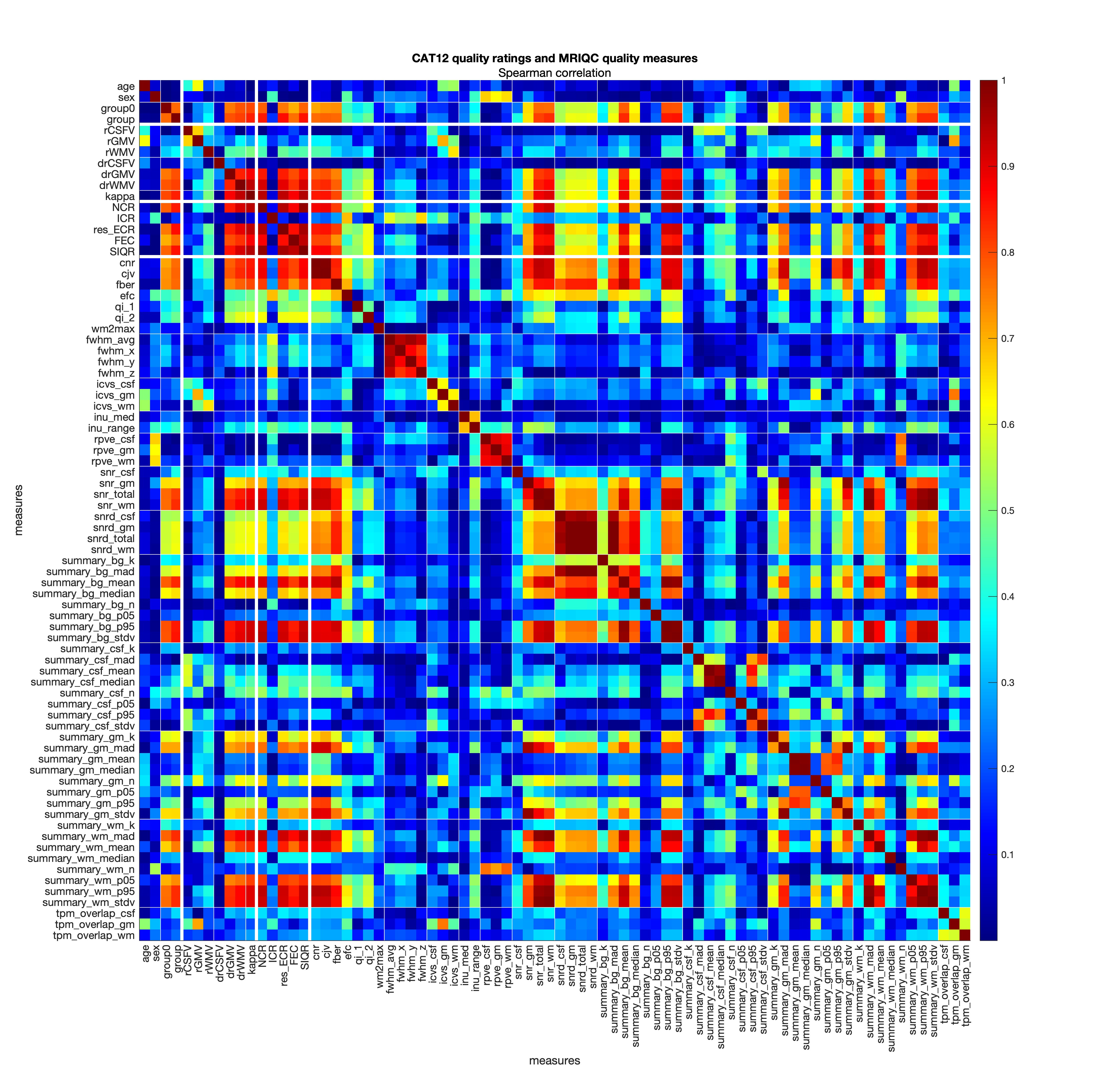

Supplement: giaf146_Supplemental_Files [file giaf146_supplemental_files.zip › figS6.jpg]
