# Supplementary material for: Segmentation-based quality control of structural MRI using the CAT12 toolbox
Source: Gigascience. 2025 Nov 29;14:giaf146. doi: 10.1093/gigascience/giaf146 (PMC12758382; doi:10.1093/gigascience/giaf146)
Supplement: giaf146_GIGA-D-25-00085_Revision_1 [file giaf146_giga-d-25-00085_revision_1.pdf]

GigaScience
Segmentation-Based Quality Control of Structural MRI using the CAT12 Toolbox
--Manuscript Draft--

Manuscript Number:	GIGA-D-25-00085R1
Full Title:	Segmentation-Based Quality Control of Structural MRI using the CAT12 Toolbox
Article Type:	Technical Note
Funding Information:	Deutsche Forschungsgemeinschaft (556008132) — Dr.-Ing. Robert Dahnke<br>Research Council of Finland (351849) — Prof. Dr. rer. nat Christian Gaser
Abstract:	Background: The processing and analysis of magnetic resonance images is highly dependent on the quality of the input data, and systematic differences in quality can consequently lead to loss of sensitivity or biased results. However, varying image properties due to different scanners and acquisition protocols, as well as subject-specific image interferences, such as motion artifacts, can be incorporated in the analysis. A reliable assessment of image quality is therefore essential to identify critical outliers that may bias results.<br>Findings: Here we present a quality assessment for structural (T1-weighted) images using tissue classification in the SPM/CAT12 ecosystem. We introduce multiple useful image quality measures, standardize them into quality scales and combine them into an integrated structural image quality rating to facilitate the interpretation and fast identification of outliers with (motion) artifacts. The reliability and robustness of the measures are evaluated using synthetic and real datasets. Our study results demonstrate that the proposed measures are robust to simulated segmentation problems and variables of interest such as cortical atrophy, age, sex, brain size and severe disease-related changes, and might facilitate the separation of motion artifacts based on within-protocol deviations.<br>Conclusion: The quality control framework presents a simple but powerful tool for the use in research and clinical settings.
Corresponding Author:	Robert Dahnke<br>Jena University Hospital: Universitatsklinikum Jena<br>Jena, GERMANY
Corresponding Author Secondary Information:	
Corresponding Author's Institution:	Jena University Hospital: Universitatsklinikum Jena
Corresponding Author's Secondary Institution:	
First Author:	Robert Dahnke
First Author Secondary Information:	
Order of Authors:	Robert Dahnke<br>Polona Kalc<br>Gabriel Ziegler<br>Julian Grosskreutz<br>Christian Gaser
Order of Authors Secondary Information:	
Response to Reviewers:	See also revision letter PDF.<br><br>Reviewer response:<br>Reviewer #1:<br>The article presents a valuable effort towards standardising quality control methods and their evaluation. However, too many choices seem arbitrary without sufficient

Powered by Editorial Manager® and ProduXion Manager® from Aries Systems Corporation

justification, and too many sections are unclear. Overall, the quality of the work cannot be fully assessed in the current state of the manuscript, and major revisions are needed to correct that. There is also not enough comparison (one) with other methods and no way of evaluating whether these measures are relevant to actual downstream imaging uses. Additionally, the article's goal is highly unclear and led me to think the segmentation measures were part of the QC pipeline until I read the discussion ... Nothing until the discussion explains that the segmentation measures are used to evaluate the single SIQR score output of the QC pipeline.

Response: Thank you very much for your feedback. Although the use of the segmentation in the conceptualisation of the QC tool is stated in the manuscript's title, we acknowledge that the playful reference to the movie title probably obscured the scope of this work. We have therefore decided to change the title of the revised manuscript.

We also agree that various arbitrary choices were made during the development of the tool, and we would like to point out that these choices were motivated by practical experience with data, which is why there are limited references for many of them. Nevertheless, we have tried to address most of the points raised in your review and have rewritten various parts of the manuscript accordingly.

Comments:

"All measures and tools are part of the Computational Anatomy Toolbox (CAT; https://neuro-jena.github.io//cat, Gaser et al., 2024) of the Statistical Parametric Mapping (SPM; http://www.fil.ion.ucl.ac.uk/spm, Ashburner et al. 2002) software and also available as a standalone version (https://neuro-jena.github.io/enigma-cat12/#standalone)." I cannot really expect everyone to avoid Matlab tools. Still, Matlab is a drag to the development of scalable tools nowadays (every system admin's nightmare is to have to try to make Matlab tools run on high-performance computing servers).

Response: We agree with you that the mentioned dependency on Matlab is not in line with open science principles. We are therefore currently developing tools that do not rely on Matlab. Nevertheless, we would like to point out that both SPM and CAT12 (and the QC tool) can be run as standalone versions (also on a high-performing clusters), and that the concept described here (simple use case specified grading, specific evaluation aspects) can be transferred to other tools, programming languages or platforms. However, we feel that the complete change of the programming language and platform would be beyond the scope of this manuscript.

"such as noise, inhomogeneities, and resolution (Figure 1B)." At this point in the article, it's a bit unclear how that works in Figure 1B.

Response: We have tried to improve the clarity in the revised part. Figure 1B represents how two segmentation algorithms are affected by varying levels of noise, inhomogeneities and resolution from the BWP dataset (revised part in italic):

"(B) The segmentation accuracy can be quantified by the kappa similarity statistic (Cohen, 1960), here presented for two segmentation approaches on simulated images (AubertBroche et al., 2006), where larger levels of noise, inhomogeneity, or lower resolutions result in a worse overlap with the full-resolution image without interference."

"It is assessed within optimized cerebrospinal fluid (CSF) and white matter (WM) regions." Then, the NCR relies on the segmentation, right? What if the segmentation fails?

Response: Correct. As you pointed out, the NCR and all other measures rely on segmentation. All image assessments rely to some extent on some kind of segmentation, which defines regions to quantify the measure and scaling. This can fail or bias the (quality) analysis. However, tissue segmentations are broadly available, accurate, and robust, and severe segmentation failures in CAT12 can be identified using covariance analysis of the segmentation as mentioned in the Section "Software" (Figure 4) and Discussion (p. 21):

"Multivariate outlier detection schemes that are typically applied based on the processed data of a sample in the normalized feature space, using similarity analysis of normalized GM data (e.g. the Gram matrix or kernels) in CAT12 (see Software section), can be used to detect outliers with preprocessing problems or highly deviating anatomy."

In addition, if the segmentation fails completely, the specific scan cannot really be used in the analysis and its quality measure is not needed anyway.

Powered by Editorial Manager® and ProduXion Manager® from Aries Systems Corporation

	Oh, most of the measures actually rely on the segmentation. Are segmentation errors accounted for in the tool? I am thinking specifically about "abnormal" brains that can be difficult for segmentation algorithms. At least at this point of the article, it's not clear.<br>Response: Yes, as the title of the paper and the header of this section state, our measures rely on segmentation. Hopefully, changing the title will make this clearer from the outset. Nevertheless, we have attempted to clarify this at the end of the section “Segmentation-based Image Quality Assessment”:<br>“The [BWP] dataset includes images with varying levels of noise, inhomogeneities, and resolution. These image properties affect the segmentation accuracy of MRI processing algorithms (Figure 1B), and are therefore useful indicators of the quality of input data.“<br><br>"To accommodate various international rating systems, we have adopted a linear percentage and a corresponding (alpha-)numeric scaling." this doesn't match the complexity of the following explanation about the rather arbitrary range. I think a much more international and understandable rating would have been a 0 to 1 range. A 0.5 to 10.5 range is not helping users at all. As the rating is linear, I am struggling to see the added value of this choice.<br>Response: Thank you very much for sharing with us your view. We would disagree that our range is arbitrary. In fact, by examining the school grading systems across the world it was evident that the underlying match between percentage and grades is relatively consistent (though with regional differences), with numerical grades often falling between 1-10 (though the positive and negative endpoints differ based on the countries’ historical background) that are partially also represented by letter grades or alternatively a percentage systems (range 0% to 100% or in other words 0 to 1). Typically, 50-60% of the points determine the threshold to pass the test. Our grading system therefore leverages the known classification. As the tool or its versions has been in use for some years and many users are familiar with it, we are concerned that changing it would cause more confusion than anything else. In addition, we use a root-mean-square like approach to combine the different ratings, which rely on this scaling to weight worse values stronger.<br>We further specified the rating system and referenced the extended description in the Method part.<br><br>"Although the BWP does not include the simulation of motion artifacts, these are in general comparable to an increase of noise in the BWP dataset by 2 percentage points." Maybe that should be justified with a reference? "in general" might be a bit light to justify not having a direct measure for something presented as important (motion artefacts) in the introduction and goal of the tool.<br>I think the absence of a noise estimation in the QC ratings should be more thoroughly justified.<br>Response: Thank you for mentioning this. We agree with you that it would be useful to have a reference to support our claim. However, as we are not aware of any specific research examining this particular topic, we have relied on our experience with data here. Additionally, although our measure development and evaluation relied on BWP, which does not include motion artifacts, we addressed this issue by testing our measures on the MR-ART dataset, which does include them (pp. 10-11). Moreover, to demonstrate our statement, we added a boxplot representing differences in SIQR between conditions with various amounts of motion in the MR-ART dataset (Figure 7). Regarding the comment about the absence of noise estimation in the QC rating, we would like to point out that the noise is considered in our rating as “noise-to-contrast ratio” (NCR).<br><br><br>"To balance the sensitivity to different quality measures while ensuring that the necessary quality conditions are met, we apply an exponentially weighted averaging approach — similar to the root mean square (RMS) but using the fourth power and fourth root." Why is there no justification or references for these arbitrary choices? Why not the fifth root or tenth root? Why the square root and not an exponential or any other function?<br>Response: Thank you for raising this issue. While we agree with you that our choice seems arbitrary from the text, it actually stems from our tests. Artifacts cannot be compensated by other image features and we therefore focused on the exponential (root-mean-square-error-like) weighting to increase the differentiation of the outliers. In

case of motion artifacts, we observed that even heavy motion only slightly reduced the average rating, so we used a higher power to increase the weighting of a single negative rating. The effect of motion artifacts on image analyses is mostly driven by the outcome of the processing, which we quantified in the BWP (and later also the MR-ART dataset) by Kappa and a systematic underestimation of GM. We tested several exponential weighting options. In the composite measure presented in the unrevised manuscript (where the inhomogeneity measure was included), there was a clear preference for the weighting of power 4 (see the image and table R1 below). However, in the revised version (with exclusion of the inhomogeneity rating), the strong preference for the weighting 4 is not as obvious. Nevertheless, the correlations alone are not a clear indicator. They are varying by tissue class, the segmentation, the combined ratings, as well as the dataset. We have focused here on the MR-ART dataset; however, many other datasets are available, but could not be included in the article. Therefore, we here provided the evidence in this dataset, while taking into account further tests that were run with datasets that were not included in this manuscript. Below is the figure representing the differentiation between groups of scans with different degrees of motion artifacts, with higher power weightings differentiating the groups better. Nevertheless, too high weightings became too sensitive for the worst feature.
As stated before, advanced users have the option to use the individual measures and get their own impression of the data. We here wanted to provide a simple human readable composite score to assess the quality of a scan.
Below is the figure representing the differentiation between groups of scans with different degrees of motion artifacts, with higher power weightings differentiating the groups better.

To sum up:
“To quantify the effect of interferences, we estimated the volume difference (ΔV) and the Kappa value to the artifact-free case (averaged over all tissue classes), where volume changes and Kappa statistics should be highly associated with the quality rating (Table 2). We finally selected the power 4 function as it is more sensitive to outliers.”

Table R1: Results from version 201901

	Vol-BWP-avg	Vol-MRART-avg	Kappa-BWP-avg	Kappa-MRART-avg	avg
median	0.45575	0.63246	0.75774	0.89138	0.68434
mean	0.45575	0.63246	0.75774	0.89138	0.68434
rms^2	0.46668	0.63406	0.77304	0.89229	0.69152
rms^4	0.48033	0.63088	0.78391	0.88453	0.69491
rms^8	0.49132	0.62275	0.78591	0.86738	0.69184
rms^{16}	0.49507	0.61576	0.78415	0.85265	0.68691
max	0.49616	0.6112	0.78241	0.84319	0.68324

"Sample Normalization for Outlier Detection" It is unclear whether this is systematically applied or not. Is it a separate measure, or is it aggregated into another score? That measure could be relevant in many cases but could also be really bad in some specific cases (for example, historical data where the "ideal" quality would probably be well below standards.
Response: We agree that this section could be described better and have introduced changes to the text to make it clearer (see pp. 9). The sample normalization results in another sample-specific rating (NSIQR - normalised SIQR) used to test how well the outliers (e.g. in the MR-ART dataset) can be identified. The rating (as described in the outlier detection) is part of the “Check Sample Homogeneity Tool” for sample evaluation before statistical analysis, and is also stored in the XML files for each subject with an extra field that saves basic information about the data (path, datetime, number).

Powered by Editorial Manager® and ProduXion Manager® from Aries Systems Corporation

Overall, our measures are defined by the typical ranges that structural MRI currently supports and that could potentially be used for tissue segmentation and surface reconstruction. As the BWP can be seen as a historical element, the principle definitions of noise, inhomogeneity and resolution still fit to today's structural data. We have tested here with a broad variability of data available from larger and smaller projects. However, the challenging part are ultra-high (7 Tesla) resolution images, where protocols are still in development and some public datasets suffer from interferences (e.g. extreme bias) that will probably play only a minor role in future standard acquisitions.

"raw (co-registered)" Well, it is not raw if it's co-registered. I suggest reformulation to avoid confusion with actual raw images.
Response: Thank you for pointing out this issue, we have omitted the wording in the revised manuscript. Although raw data (defined as the output of the Dicom conversion) present the default, researchers often use the term “raw” even if it includes preprocessing (e.g., resliced/resampled, defacing, skull-stripping, denoised, bias-corrected, or intensity normalized data) in different contexts.

The "Evaluation Concept and Data" section is very unclear. The need for a training-testing scheme is not explained, and the scheme itself is very arbitrary (choosing odd and even numbered files ordered by filenames). How does that splitting strategy help with generalisation? Why that specific split? Why not another? How do we know that split is not biased?
Response: We agree that this description appears arbitrary and we refined this section. Briefly, the split was used to define and scale the measures on another subset of data. We opted for a simple odd-even split since the BWP parameters are included in the filenames. This means that selecting every second data point provides a balanced and comprehensive sample that includes similar, but not identical, cases.

Finally, the selection of 6 scans also seems completely arbitrary. Overall, this section does not provide enough information to justify the seemingly arbitrary choices.
Response: Thanks for pointing this out. The dataset used for this analysis is highly diverse with regards to different scanning parameters and was not acquired by us. Six scans with different scan times, resolution, and parallel imaging, but similar with regard to other imaging parameters (TR/RE times) were selected only to illustrate the working of the QC on an example. We have added our selection criteria for the scans in the description of the samples.

"Of note, obvious subject/scan-specific motion artifacts generally increase the scans' rating for about 1 grade, which corresponds to a decrease of 10 rps (and +0.5 grade / -5 rps for light artifacts), in comparison to the typical rating achieved by the majority of scans of the same protocol." This is incredibly vague! How are readers supposed to evaluate the quality control measures with this information?
Response: We agree that from this statement, the users cannot evaluate the quality measures, nevertheless, we have tried to provide evidence in our manuscript to showcase the validity and reliability of the measures. The readers can rely on the numerical output of the toolbox to decide about inclusion or exclusion of the scan. The above statement was meant as a general observation and not as evidence, as we currently have no measure or classification for motion artifacts.

Discussion:
"as this is more relevant for segmentation and surface reconstruction (Ashburner et al., 2005)." A lot of work has been done in these domains in 20 years; this reference, however solid, is not enough to justify that choice. This might not be relevant with the methods developed in the last 20 years.
Response: Thank you for this observation. We updated the references in this part of the manuscript:
“Moreover, the proposed intensity-based measures are normalised by (minimum) tissue contrast rather than signal intensity, as the separation between brain tissues, especially the GM and WM, is essential for segmentation and surface reconstruction (Fischl et al. 2012, Gaser et al., 2025).“

Powered by Editorial Manager® and ProduXion Manager® from Aries Systems Corporation

"with a power of 4 rather than 2, to place greater emphasis on the more problematic aspects of image quality." Still not enough to justify that choice. The authors failed to convince me that one single score is better than reporting all the measures significantly, as different quality measures will influence different tasks. A very practical example is the fact that the vast majority of acquisitions in clinical settings, the resolution is anisotropic (though less with T1 images nowadays, historical datasets will still have it). This anisotropy is not necessarily an issue for human diagnosis, for example; however, aggregating all the scores in one might hide that a low-quality measurement might not affect the specific downstream task. Coupled with the lack of justification for the factor scalings, this choice of a single score is a significant negative point for the tool.
Response: Indeed, we agree that for particular use cases, the specific (raw/unscaled) measures are preferable and can also be used. We do not object to that and provide the unscaled measures as an output in xml files. However, the composite measure was designed to meet the needs of the non-advanced users of structural preprocessing tools (for T1w-based segmentation and surface reconstruction) to get a simple estimate of the quality of their data to identify critical cases. We extended the discussion:
"Nevertheless, raw quality measures are also available in the XML files, allowing advanced users to perform detailed inspections." (p.24)
With regard to the lack of justification for scaling, we have addressed this in the Averaging section in the Method part.

Data availability:
Where can the sources of these specific tools be accessed?
Response: The raw data is available from the original sources, whereas processed data is available from the Gigascience server. We improved the data and code availability report in the revised manuscript.

Reviewer #2:
Technical Note GIGA-D-25-00085 introduces a segmentation-based quality control (QC) framework for T1-weighted structural MRI integrated into the CAT12 toolbox. The approach defines five interpretable image quality metrics—noise-to-contrast ratio (NCR), inhomogeneity-to-contrast ratio (ICR), resolution score (RES), edge-to-contrast ratio (ECR), and full-brain Euler characteristic (FEC)—which are combined into a composite Structural Image Quality Rating (SIQR). The tool aims to provide a standardized, interpretable scoring system for identifying poor-quality scans, with validation across simulated datasets and real-world imaging data.
Strengths
The manuscript addresses a critical need in neuroimaging by presenting an automated, interpretable, and practical framework for quality control of T1-weighted structural MRI. By integrating multiple segmentation-derived metrics into a single Structural Image Quality Rating (SIQR), the approach enables fast, standardized assessment of image quality. The tool is embedded in the widely used CAT12/SPM ecosystem, facilitating adoption, and it is validated across a range of synthetic and real-world datasets. The scoring system is designed with user accessibility in mind, offering a clear grading scale and robust detection of motion-related artifacts, making it particularly well-suited for use in large-scale research and clinical imaging settings.

Weaknesses

1. **Ambiguity of scope and segmentation dependency.**
A fundamental issue with the manuscript is its failure to clearly define the proposed QC framework's intended scope. If it is intended as a general-purpose image quality assessment tool, then several limitations become critical: its reliance on accurate tissue segmentation (1), its omission of background signal (2), its restricted validation within the CAT12 pipeline (3), and its lack of demonstrated interoperability with other workflows or populations. The method's reliability across different segmentation tools (e.g., FreeSurfer, FSL, SynthSeg) or in anatomically atypical populations (e.g., pediatric, lesioned brains) is untested (4). Conversely, if the framework is intended as a CAT12-specific internal QC tool, then the presentation is misleading. The inclusion of cross-tool benchmarks (e.g., MRIQC) (5), the use of generalized grading schemes, and the claims of robustness (6) give the impression of broader applicability. In this

Powered by Editorial Manager® and ProduXion Manager® from Aries Systems Corporation

narrower interpretation, some concerns (e.g., pipeline generalization) would be less pressing, but others—such as the MRIQC comparison—become more problematic and unjustified. The manuscript would benefit greatly from explicitly stating whether the goal is a broadly applicable QC solution or a targeted add-on for CAT12 workflows.

Response: We appreciate your constructive feedback. In the revised version, we tried to implement the points that you raised as much as we could. The scope of our manuscript was not to present the tool as a general-purpose image quality assessment tool and we have hopefully made this clearer in the revised version. We outline early on that the tool is useful only for evaluation of structural T1-weighted images used for tissue segmentation and surface reconstruction within the SPM/CAT12 ecosystem. We address your other concerns below:

Reliance on accurate tissue segmentation

We see the potential disadvantage of relying on tissue segmentation. We acknowledge that the segmentation can fail, and we have simulated extreme cases of segmentation problems to test their effect on the quality measures (results available in the Supplementary material). These perturbances affect the variation in the quality measures; however, this variation is generally smaller than that caused by typical (light) motion artifacts. We have mentioned the potential problems with segmentation also in the Discussion, where we pointed out that severe segmentation problems can be identified by using covariance analysis of the segmentation.

(“Multivariate outlier detection schemes that are typically applied based on the processed data of a sample in the normalized feature space, using similarity analysis of normalized GM data (e.g. the Gram matrix or kernels) in CAT12 (see Software section), can be used to detect outliers with preprocessing problems or highly deviating anatomy. However, the proposed image quality assessments are specifically designed to measure differences of image quality (in native space) rather than segmentation accuracy (in normalized space) or anatomical properties, such as stroke lesions, and can therefore be used in addition to previously mentioned outlier detection schemes to identify cases where image artifacts could bias analysis.” p.21)

Omission of background

We agree that the omission of background does not allow our QC tool to find certain artifacts that are prominent in the background. However, the main purpose of our QC tool is to determine the usefulness of the image for the prospective brain analysis. If the artifacts such as ghosting and wrap-around affect large areas of brain tissue, they are accounted for in the quality measure, but not if they are only in the background as this is of no use to the final analysis. As we mentioned in Discussion: “For instance, in cases when locally limited or mild artefacts affect regions that are not relevant to the study (e.g., if the study focuses on frontal regions, cerebellar artefacts from jaw movements are acceptable) or whenever lower preprocessing accuracy is acceptable (e.g., for local alignment of brain surfaces or atlases for other modalities).”

Restricted validation to CAT12

Although the analysis was focused on CAT12, we also presented the results in SPM segmentation (Figure 7) as both of these tools come from a similar ecosystem. Since our scope was not to provide an overarching image quality control tool, we did not focus on comparison with segmentation from other tools. We specified a scope in the revised manuscript.

Test in other populations and lesioned brains

The test in lesioned brain was performed in the ATLAS database (Figure 6B). We also tested the toolbox internally on data of children, however, we did not present the results in the current manuscript. We added the analysis on children in the revised version (Figure 6C & D) and used the children sample to better explain our outlier detection scheme.

Inclusion of comparison to MRIQC

We acknowledge that the comparison to the MRIQC might have appeared unfair and that was not our intention (we expand upon this below at #4). We have corrected this section in the manuscript.

Claims about generalized grading schemes, robustness

We believe that the claims about the usefulness and robustness still hold within the

	limited scope of the SPM/CAT framework and hope the revised manuscript properly addresses your concerns.<br><br>2. **Lack of compliance with GigaScience reproducibility standards.**<br>The manuscript does not currently meet GigaScience's data and code availability requirements. The code used to generate results and figures is not publicly accessible—only available upon request—which directly conflicts with the journal's expectations for open, reproducible research. Similarly, while the data are drawn from public sources, the manuscript lacks direct links, accession numbers, or DOIs for the datasets used, and provides no clarity on data preprocessing or analysis scripts. There is also no reference to licensing for the CAT12 toolbox or the code used in the study, and no reproducibility capsule (e.g., containerized environment, workflow script) is offered. These omissions limit the transparency and reusability of the work and must be addressed to comply with the FAIR principles and GigaScience's editorial policies.<br>Response: The code and data are available, but the Gigascience FTP was set up during the submission process and were therefore not included in the main text. We improved this in the revised version.<br><br>3. **Mischaracterization of background-based IQMs**.<br>In the "SIQR measure development" section, the manuscript states: "Image quality measures are commonly estimated from the image background (Mortamed et al., 2008; Esteban et al., 2017)." This statement is factually incorrect and conceptually misleading. First, the citation is incorrect—Mortamed should be Mortamet (2009). Second, it misrepresents tools like MRIQC, where most quality metrics are computed within brain tissue, including CJV, SNR, and contrast-based measures. Third, the authors entirely omit recent work (e.g., Pizarro et al., 2016; Provins et al., 2025\) showing that artifacts such as ghosting, wrap-around, and motion often manifest more clearly in the background, due to the nature of Fourier reconstruction. By excluding background regions, the proposed method may miss artifacts that are visible but lie outside the segmented brain, and the trade-offs of this design decision are not discussed. The rationale based on defacing is only partial: defacing typically removes the face, not the broader background, where artifact signals often dominate. The statement as written oversimplifies QC practices and signals a bias toward justifying the framework's internal constraints rather than engaging with the full methodological landscape.<br><br>**References:**<br>Provins, C., … Esteban, O. (2025). Removing facial features from structural MRI images biases visual quality assessment PLOS Biology. doi:[10.1371/journal.pbio.3003149](https://journals.plos.org/plosbiology/article?id=10.1371/journal.pbio.3003149) (OA).<br>Pizarro RA, et al. (2016). Automated quality assessment of structural magnetic resonance brain images based on a supervised machine learning algorithm. Front Neuroinf. 10\. doi:[10.3389/fninf.2016.00052](https://doi.org/10.3389/fninf.2016.00052).<br>Response: Thank you for the correction, we have updated the statement in the revised manuscript. Nevertheless, we would argue that even if the artifacts that you mention are more clearly visible in the background, if they affect the brain, they are also visible in the brain tissue segmentation and our measures account for that. As we have shown in the revised version of the manuscript, the SIQR measure is highly associated with the summary statistics of the background from MRIQC. Moreover, it is not only defacing, but also other varying properties of the available structural images that limit the extraction of quality measures from the background (e.g., different backgrounds, for instance in MP2RAGE protocols, skull-stripped data etc.).<br>4. **Underdeveloped and opaque benchmarking against MRIQC.**<br>The benchmarking against MRIQC is reported only in the Results section, with no corresponding description in the Methods. It is surprising that MRIQC is not mentioned by name until page 14, despite the Esteban et al. (2017) reference appearing earlier in a different context. This suggests that the treatment of MRIQC—a widely adopted, general-purpose QC tool—has not been as thorough or fair as would be desirable. Key methodological details are missing: the authors do not explain how MRIQC was executed, how specific features (e.g., snr_wm, cjv) were selected, or whether a multivariate classifier was considered. Given that MRIQC's full model leverages multiple features simultaneously, limiting the comparison to univariate metrics weakens

the validity of the claim that SIQR outperforms existing approaches. A more balanced, transparent benchmarking setup would strengthen the manuscript considerably. This benchmarking also mentions an "SPM12-based" QC performance but does not clarify how and why this comparison is made.
Response: Thank you for pointing this out. Perhaps this became unclear during the analysis and writing-up process, but as we did not intend to provide an alternative to MRIQC in the form of a general-purpose tool, the comparison was not included in the Method section. The analysis was primarily intended to test the performance of our measures in the MR-ART dataset with the available expert ratings. However, we noticed that the MRIQC measures were also available and additionally assessed the validity against the measures from a related tool. We agree that the initial version of the manuscript presented an unfair and underreported comparison, so we have updated and rephrased the relevant section.
Regarding the use of SPM, we included it to check if the measure is useful also for another input segmentation, where we selected the Unified Segmentation from SPM as it is directly available in the same ecosystem. This allowed us to test the expected atrophy in case of motion also for another tool.

5. **No analysis of failure cases.**
The manuscript does not present examples of false positives or false negatives—cases where SIQR fails to align with visual inspection or known ground truth. Without understanding when and why the metric fails, users cannot judge the risk of misclassification or apply it conservatively in sensitive datasets.
Response: Thank you for this suggestion. We have included this part in the Supplement.

Minor Issues
* Figure 7 could benefit from clearer annotation of thresholds and misclassified cases to help interpret the ROC curves.
Response: Thank you for your comment. We have tried to improve the readability of the figure.

* While the title *"The Good, the Bad, and the Ugly"* is a play on the classic western film, this informal or humorous reference may be perceived as inappropriate in a scientific context—especially for a methods paper intended to support standardization and reproducibility. The title does not convey the technical scope or scientific contribution of the work, which may undermine its visibility and perceived rigor. A more descriptive and neutral title—e.g., "Segmentation-Based Quality Control of Structural MRI using the CAT12 Toolbox"—would better reflect the content and purpose of the manuscript.
Response: We appreciate this helpful comment. We changed the title to better represent the content of the paper.

* While the authors validate their approach against synthetic degradations and segmentation-derived kappa scores, they do not sufficiently leverage human expert QC ratings. Greater engagement with visual QC standards would make the case for SIQR's practical value more compelling.
Response: Expert ratings from MR-ART were used and we have stated this more clearly in the revised manuscript.

I was given access to the supporting data but chose not to proceed with reproducibility checks at this stage, as the manuscript does not currently meet GigaScience's basic standards for code and data transparency. I look forward to reviewing a revised version that clearly defines the scope of the method, improves methodological transparency, and brings the manuscript into compliance with the journal's reproducibility and FAIR data principles.

Best regards,

Oscar Esteban, Ph. D.
Research and Teaching FNS Fellow
Dept. of Radiology, CHUV, University of Lausanne

Reviewer #3:

The paper describes an alternative way to QC T1w images with 2 major innovations: a different set of metrics not relying on background and a global score that combines those metrics. In addition, all of this is integrated in a well maintained toolbox allowing easy usage.

I only have suggestions (ie it does not have to be all done) as the overall paper is well written, easy to follow and analyses well conducted.
Thank you very much for your positive feedback. We have included your suggestions in the Supplement.

P6 NCR: it can be nice to demonstrate how it performs compared to traditional CNR (mean of the white matter intensity values minus the mean of the gray matter intensity values divided by the standard deviation of the values outside the brain) -- differs markedly because of background difference for sure, since you have plenty of test images you could show that more clearly (later in the method, based on what criteria/reason 'local' is defined as 5*5*5?)
Response: Thank you for your suggestion. We have added the comparison of CNR/NCR to the Kappa statistics to the Methods section (Figure 10).

P7 ECR should capture something similar to Entropy Focus Criterion, would be nice to provide a direct comparison
Response: Indeed, the measures are associated (rho = -0.5292) but the ICR was even a bit higher correlated (rho = -0.6852). We added the correlation matrix to the Supplement (Figure S6).

P8 typo, you meant equation 2
Response: Thank you for pointing it out. We have corrected the typo.

P8 SIQR I'm guessing you have experimented with the power function - maybe a side note to share your experience of why or how it works better than eg square
Response: We added the evaluation and further description to the supplement (Figure S1).

Dr Cyril Pernet

Reviewer #4:
Reproducibility report for: The Good, the Bad, and the Ugly: Segmentation-Based Quality Control of Structural Magnetic Resonance Images
Journal: GigaScience
ID number/DOI: GIGA-D-25-00085
Reviewer(s): Laura Caquelin, Department of Clinical Neuroscience, Karolinska Institutet, Sweden [Worked on reproducing the results and wrote the report]
Tobias Wängberg, Department of Clinical Neuroscience, Karolinska Institutet, Sweden [Worked on reproducing the results]

1.Summary of the Study
The study addresses how variability in magnetic resonance images quality, especially from motion artifacts or scanner differences, can affect structural image analysis. It proposes a quality assessment framework for T1-weighted images based on tissue classification and standardized image quality measures. The method is shown to be robust across datasets and conditions, helping to detect outliers and control for motion-related artifacts.

2.Scope of reproducibility

According to our assessment the primary objective is: to develop and validate a standardized framework for assessing the quality of structural (T1-weighted) MRI images, enabling the detection of artifacts on simulated data.

- Outcome: Quantitative quality ratings derived from image properties such as noise-to-contrast ratio (NCR), inhomogeneity-to-contrast ratio (ICR), resolution score (RES), and edge-to-contrast ratio (ECR) and Full-brain Euler characteristic (FEC) combined into a Structural Image Quality Rating (SIQR).

- Analysis method outcome: Not precised in the manuscript, but with the Matlab script we identified that the quality scores were correlated using Spearman's rank correlation, and statistical significance was assessed using p-values computed using MATLAB's built-in method.

- Main result: Results are presented in Figure 5. "The evaluation on the BWP test dataset showed that most quality ratings have a very high correlation (rho > .950, p < .001) with their corresponding perturbation and a very low correlation (rho < |0.1|) with the other tested perturbations (see table in Figure 5A & C). This suggests considerable specificity of the proposed quality measures. The combined SIQR score also showed a very strong association with the segmentation quality kappa (rho = -.913, p < .001) and brain tissue volumes (rhoCSF/GM/WM = -.472/-.484/.736, pCSF/GM/WM < .001) (Figure 5B). […]
The edge-based resolution measure ECR, on the other hand, generally performed better (rho = .828, p < .001), but was more affected by noise (rho = .306, p < .001) and inhomogeneity (rho = .223, p < .001) than other scores."

3.Availability of Materials
a.Data
- Data availability: Open
- Data completeness: Complete, all data necessary to reproduce main results are available
- Access Method: Private journal dropbox but also available on Github repository
- Repository: https://github.com/ChristianGaser/cat12
-Data quality: Structured
b.Code
- Code availability: Share in the private journal dropbox but also open
- Programming Language(s): Matlab
- Repository link: https://github.com/ChristianGaser/cat12
- License: GPL-2.0 License
- Repository status: Public
- Documentation: Readme file

----

4. Computational environment of reproduction analysis

- Operating system for reproduction: MacOS 15.5 (reviewer 1) and MacOS 15.1 (reviewer 2)
- Programming Language(s): Matlab
- Code implementation approach: Using shared code
- Version environment for reproduction: Matlab R2024b Update 6 (24.2.2923080) - Trial version

----

5. Results

5.1Original study results
- Results 1: Figure 5 C (see screenshot)

5.2Steps for reproduction

Powered by Editorial Manager® and ProduXion Manager® from Aries Systems Corporation

->Finding how to reproduce the results
- Issue 1: The methods section lacks sufficient detail regarding the statistical methodology, and the relevant information is not fully provided in the GitHub repository.
-- Resolved: A message has been sent to the authors requesting further clarification on the methodology and additional resources (scripts/data) needed to reproduce the results. The script to reproduce the results is "cat_tst_qa_bwpmaintest.m".

-> Reproduce the results using the "cat_tst_qa_bwpmaintest.m" script.
- Issue 2: To run the script "cat_tst_qa_bwpmaintest.m", the "eva_vol_calcKappa" function is missing.
-- Resolved: The script was shared and added to the Github repository.
- Issue 3: While running the script, the following error message encountered:
Assigning to 0 elements using a simple assignment statement is not supported. Consider using comma-separated list assignment.

```
Error in cat_tst_qa_bwpmaintest (line 481)
     default.QS{find(cellfun('isempty',strfind(default.QS(:,2),'FEC'))==0),4} = [100, 850];
```

-- Resolved: This error stops the execution of the script. After discussion with the authors, the exact cause of the error encountered at line 480 was not directly identified. We exchanged and compared our environments at the point just before the error occurred and observed notable differences between them. Our environment is almost empty. The authors identified that the default variable is missing from our environment, even though it is referenced at line 437 by a call to the cat_stat_marks function.
We confirmed that all required dependencies were installed (including Statistics toolbox, SPM and CAT12), and that we had access to all the necessary data.
To ensure the issue was not due to user error, the code was independently executed by two reviewers. The error was consistently reproduced in both cases. About the setup, I specified to the authors:
"To summarize my setup:
*I have installed SPM, CAT, and the Statistics Toolbox.
*I downloaded all datasets from the GigaScience server.
*I also downloaded the IXI T1 data, but I've only kept the version available on the GigaScience server in my working directory. Is the version from GigaScience sufficient? I had presumed that this dataset was pre-processed and ready to use, so I ignored the time-consuming pre-processing step. Your last email seems to confirm this point."

The authors answered that:
« Yes, this is correct. However, both directories have to be combined so that the original IXI images and the processing files are included. »

In an attempt to proceed, we modified the portion of the code that triggered the error:

```
######
% FEC
   FECpos = find(cellfun('isempty',strfind(default.QS(:,2),'FEC'))==0);
   try
     warning off;
     [Q.fit.FEC, Q.fit.FECstat]  = robustfit(Q.FECgt(M,1),Q.FECo(M,1));
     warning on;
     if ~isempty(FECpos)
       default.QS{FECpos,4} = round([Q.fit.FEC(1) + Q.fit.FEC(2), Q.fit.FEC(1) +
Q.fit.FEC(2) * 6], -1);
     end

   catch
     Q.fit.FEC = [nan nan]; Q.fit.FECstat = struct('coeffcorr',nan(2,2),'p',nan(2,2));
     if ~isempty(FECpos)
       default.QS{FECpos,4} = [100 850];
     end
   end
#####
```

Following this adjustment, the end of the script "cat_tst_qa_bwpmaintest.m" ran without

Powered by Editorial Manager® and ProduXion Manager® from Aries Systems Corporation

	issue and generated output results:<br><br>Finally, the error was identified after numerous exchanges with the authors. The function "cat_stat_marks", available in the Github repository, was not shared in the FTP server. With this function added, the script runs correctly. Please note that the link to the Github repository where the software code can be found is not specified in the manuscript.<br><br>-> Compare the results reproduced and the original results<br>- Issue 4: Discrepancy between reproduced results, output results provided by the authors and the original results shown in Figure 5C.<br>-- Unresolved: We reproduced the figures and the corresponding output table using the modified "cat_tst_qa_bwpmaintest.m" script. We ran the script using the only default QC version selected in the script ("cat_vol_qa201901x"). By comparing our output with the result files shared by the authors, we were able to confirm that we had executed the correct pipeline.<br>However, we encountered a discrepancy: neither the generated file in our run (tst_cat_col_qa201901x_irBWPC_HC_T1_pn9_rf100pC_vx200x200x200rptable.csv) nor the corresponding file provided by the authors (outputs from BWPmain_full_202504) matched the numerical values presented in Figure 5C of the manuscript.<br>We contacted the authors to clarify whether the default QC version used in the script was indeed the one produce the figure. In response, they confirmed:<br><br>"All figures should show the results of this QC version although I had the plan to run a final check update after the reviewer comments (the figures are finally arranged in Adobe Illustrator)."<br><br>Therefore, although the correct version of the QC was used, the differences in the results shown in Figure 5C remain unexplained. This issue is still unresolved.<br>Response: Thank you for pointing this out. As mentioned in the initial communication with the editor, we estimated the values for the table from the images preprocessed with an earlier CAT12 version. Unfortunately, a part of preprocessed scans was accidentally deleted during the upload of the images to the Gigascience server, and had to be reprocessed, for which a current CAT12 version was used. The differences in the values were therefore expected (albeit not to a drastic extent), but we decided to correct the figures after the review process. We now updated the values in the figures and tables. Of note, the QC version that the reviewers used is the same as used for the manuscript.<br><br><br>5.3 Statistical comparison Original vs Reproduced results<br>- Results: Screenshot of reproduced tst_cat_vol_qa201901x_irBWPC_HC_T1_pn9_rf100pC_vx200x200x200_rptable.csv table<br><br>- Comments: Several p-values in the reproduced results appear as exactly 0 (0.00000000e+00), which is unlikely from a statistical point of view. It is possible that these values are just extremely small and were rounded down. However, this could also point a problem in the script. Further investigation would be needed to determine the cause.<br>Response: Thank you for this observation. The result of 0.00000000e+00 signifies a statistically significant result. Due to the smallness of the value, however, the number cannot be represented by a computer (i.e., arithmetic underflow). This is a common phenomenon in statistical programs. Because the probability of getting such or more extreme results when the null hypothesis is true cannot be 0, we reported this result as p<.0001.<br><br>- Errors detected: Values in Figure 5C do not correspond to those provided by the authors in the FTP server in the files (tst_cat_vol_qa201901x_irBWPC_HC_T1_pn9_rf100pC_vx200x200x200_rptable.csv). Multiple inconsistencies were observed, suggesting potential errors in the manuscript figure or mismatches between file versions (see file Comparison_original_rptable_vs_fig5C_data.csv for comparison).

Powered by Editorial Manager® and ProduXion Manager® from Aries Systems Corporation

	Response: Thank you for pointing this out. As mentioned before, we estimated the values for the table from the images preprocessed with an earlier CAT12 version, but had to reprocess the images due to the accidental deletion of files during upload. A newer version of CAT12 was used for the preprocessing for the images on the server. The differences in the values were therefore expected (albeit not to a drastic extent), but we decided to correct the figures after the review process. We now updated the values in the figures and tables.<br>(Screenshot of Figure 5C)<br><br>(Screenshot of the original output corresponding to the Figure 5C)<br><br>- Statistical Consistency: The reproduced correlation table (tst_cat_vol_qa201901x_irBWPC_HC_T1_pn9_rf100pC_vx200x200x200_rptable.csv). differs from the original in terms of r-values and p-values.<br>Compared to the Figure 5C, the reproduced r-values do not all match those shown in the figure. P-values cannot be directly compared to Figure 5C, as they are represented by a color gradient without a scale or legend, making direct comparison impossible.<br>Response: Thank you for pointing this out - the same comment applies as above. We added the full correlation table with rho and p-values in the supplement.<br>--<br><br>6. Conclusion<br>- Summary of the computational reproducibility review<br>The computational reproducibility of the main result we identified for the study is partially achieved. After several technical issues related to missing functions, I was able to execute the script to reproduce values of Figure 5C ("cat_tst_qa_bwpmaintest.m") and obtain ouput results. However, discrepancies were observed when comparing the reproduced results (tst_cat_col_qa201901x_irBWPC_HC_T1_pn9_rf100pC_vx200x200x200rptable.csv) to both:<br>- the output file provided by the authors, and<br>- the original results presented in figure 5C of the manuscript.<br>Notably, the output file p...
**Additional Information:**	
**Question**	**Response**
Are you submitting this manuscript to a special series or article collection?	No
**Experimental design and statistics**<br><br>Full details of the experimental design and statistical methods used should be given in the Methods section, as detailed in our Minimum Standards Reporting Checklist. Information essential to interpreting the data presented should be made available in the figure legends.<br><br>Have you included all the information requested in your manuscript?	Yes
**Resources**<br><br>A description of all resources used,	Yes

including antibodies, cell lines, animals and software tools, with enough information to allow them to be uniquely identified, should be included in the Methods section. Authors are strongly encouraged to cite Research Resource Identifiers (RRIDs) for antibodies, model organisms and tools, where possible.<br><br>Have you included the information requested as detailed in our Minimum Standards Reporting Checklist?	
**Availability of data and materials**<br><br>All datasets and code on which the conclusions of the paper rely must be either included in your submission or deposited in publicly available repositories (where available and ethically appropriate), referencing such data using a unique identifier in the references and in the “Availability of Data and Materials” section of your manuscript.<br><br>Have you have met the above requirement as detailed in our Minimum Standards Reporting Checklist?	Yes
GigaScience has policies and guidelines in place for the use of generative AI-writing tools such as ChatGPT. If you have used such writing tools to assist with writing the manuscript this must be declared and cited in the text. Authors should not list AI-writing tools and other AI-assisted technologies as an author or co-author and should acknowledge that they are fully responsible for text generated or refined by AI-writing tools.<p><br><br>A summary of use (particularly in the introduction or among methods) needs to be included at the end of the paper, and the outputs should also be included as a supplementary file hosted in GigaDB or other open repositories. Please read our guidelines for more information. <p>	No

Powered by Editorial Manager® and ProduXion Manager® from Aries Systems Corporation

By submitting to GigaScience, you are aware of the journal's AI-writing tools policy, and if you have declared use of such tools below, you have acknowledged this where appropriate in your manuscript and have made a summary of use and outputs available. <p> <b>AI-assisted writing tools have been used in the preparation of this manuscript?	

Powered by Editorial Manager® and ProduXion Manager® from Aries Systems Corporation

Manuscript

Click here to access/download;Manuscript;QC_V4_gigascience_R1.pdf

Segmentation-Based Quality Control of Structural MRI using the CAT12 Toolbox

Robert Dahnke [1,2,3,*], Polona Kalc [1,2], Gabriel Ziegler [4], Julian Grosskreutz [5,6], Christian Gaser [1,2,3]

1 - Department of Neurology, Jena University Hospital, Jena, Germany

2 - Department of Psychiatry and Psychotherapy, Jena University Hospital, Jena, Germany

3 - German Center for Mental Health (DZPG), Jena-Halle-Magdeburg, Germany,

4 - University Hospital Magdeburg and DZNE Magdeburg, Magdeburg, Germany

5 - Department of Neurology, Jena University Hospital, Jena, Germany

6 - Precision Neurology, Department of Neurology, University of Lübeck, Lübeck, Germany

* - corresponding author

Abstract

Background: The processing and analysis of magnetic resonance images is highly dependent on the quality of the input data, and systematic differences in quality can consequently lead to loss of sensitivity or biased results. However, varying image properties due to different scanners and acquisition protocols, as well as subject-specific image interferences, such as motion artifacts, can be incorporated in the analysis. A reliable assessment of image quality is therefore essential to identify critical outliers that may bias results.

Findings: Here we present a quality assessment for structural (T1-weighted) images using tissue classification in the SPM/CAT12 ecosystem. We introduce multiple useful image quality measures, standardize them into quality scales and combine them into an integrated structural image quality rating to facilitate the interpretation and fast identification of outliers with (motion) artifacts. The reliability and robustness of the measures are evaluated using synthetic and real datasets. Our study results demonstrate that the proposed measures are robust to simulated segmentation problems and variables of interest such as cortical atrophy, age, sex, brain size and severe disease-related changes, and might facilitate the separation of motion artifacts based on within-protocol deviations.

Conclusion: The quality control framework presents a simple but powerful tool for the use in research and clinical settings.

Keywords

MRI, brain, quality control, quality assessment, segmentation, motion artifacts

List of abbreviations

BWP	Brain Web Phantom
CAT	Computational Anatomy Toolbox
CSF	CerebroSpinal Fluid
GM	Gray Matter
ICR	Inhomogeneity Contrast Ratio
NCR	Noise Contrast Ratio
MRI	Magnetic Resonance Imaging
SPM	Statistical Parametric Mapping
RES	Root mean square Resolution Rating
ECR	Edge Contrast Ratio
FEC	Full-brain Euler Characteristic
QC	Quality Control
SIQR	Structural Image Quality Rating
TIV	Total Intracranial Volume
WM	White Matter

Background

Multicentre *magnetic resonance imaging* (MRI) studies and data-sharing projects have become increasingly common in cognitive and clinical neuroscience in recent years. The collaboration of several imaging centers, allowing for increased statistical power through larger sample sizes, is especially beneficial for investigating rare diseases and individual differences (Bethlehem et al., 2022; Markiewicz et al., 2021; Wiseman et al., 2019). However, project deviations from the initial research plans (e.g., switching from functional to a structural imaging focus), differences and changes of imaging hardware and software, quality assurance procedures, and the resulting image quality variations may introduce bias in subsequent image processing and statistical analysis (Ai et al., 2021; Bottani et al., 2022; Kruggel et al., 2010; Reuter et al., 2015). In particular, the presence of noise, (motion) artifacts, inhomogeneity, or reduced resolution could affect image processing, even when such interferences are modeled and partially corrected during data processing (AubertBroche et al., 2006; Nárai et al., 2022; Tian et al., 2021; Figure 1).

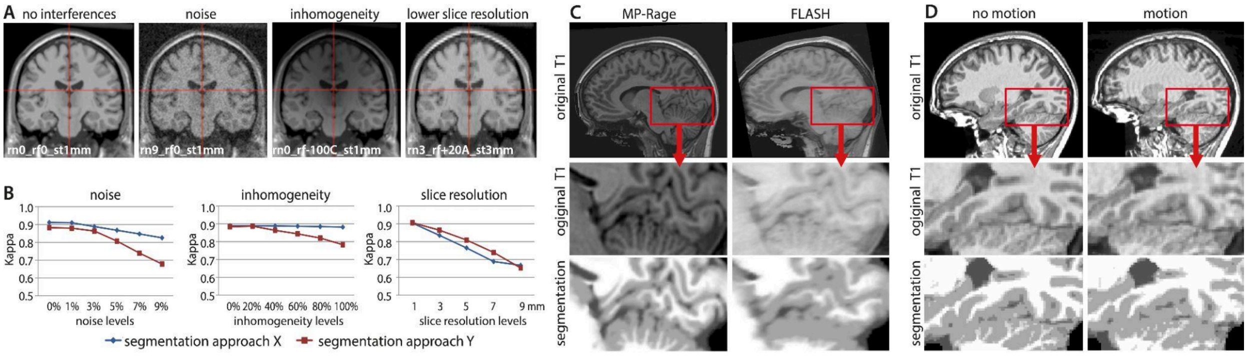

Figure 1: (A) Image properties such as noise, inhomogeneity, and resolution influence the segmentation accuracy. (B) The segmentation accuracy can be quantified by the kappa similarity statistic (Cohen, 1960), here presented for two segmentation approaches on simulated images (AubertBroche et al., 2006), where larger levels of noise, inhomogeneity, or lower resolutions result in a worse overlap with the full-resolution image without interferences. (C) Shows an illustration in real data with reduced anatomical details in a FLASH protocol (Kempton et al., 2011) or (D) in case of movement artifacts (MR-ART sub-988484 from Nárai et al., 2022).

Typically, manual *quality control* (**QC**) checks each image for scan-specific interferences (e.g., motion artifacts) by visual inspection to remove outliers (Keshavan et al., 2018, Kruggel et al., 2010;

Nakua et al., 2023). However, manual assessment is time-consuming, highly subjective, and typically relies on project-specific definitions (Nárai et al., 2022). To make this process more efficient and reliable, automated quality control approaches have been proposed for structural (Bottani et al., 2022; Esteban et al., 2017; Mortamet et al., 2009), functional (e.g., Christodoulou et al., 2013), and diffusion imaging (e.g., Maximov et al., 2021). In addition, the image quality estimates can be used to harmonize imaging data (Garcia-Dias et al., 2020; Lutti et al., 2022; Pomponio et al., 2019). A systematic overview of different QC frameworks has been provided by Hendriks et al. (2024).

In this study, we propose a powerful and easily applicable QC framework for structural (T1-weighted) MRI data within the SPM/CAT12 framework. Earlier versions have been extensively evaluated in Gilmore et al. (2021) and Ma et al. (2022). The proposed QC framework introduces, standardizes and integrates different quality metrics into a continuous *structural image quality rating* (**SIQR**). It supports both automatic and interactive assessments of a preprocessed MRI scan's suitability for prospective use, as well as the identification of potential outliers within a sample, ensuring unbiased data analysis. All measures and tools are part of the *Computational Anatomy Toolbox* (**CAT;** https://neuro-jena.github.io//cat, Gaser et al., 2024) of the *Statistical Parametric Mapping* (**SPM;** http://www.fil.ion.ucl.ac.uk/spm, Ashburner et al. 2002) software and also available as a standalone version (https://neuro-jena.github.io/enigma-cat12/#standalone).

Findings

Here we present the rationale for a segmentation-based QC framework, a definition of several quality measures, their standardization into quality scales and the integrated composite measure *Structural Image Quality Rating* (**SIQR**). We further describe the detection of imaging artifacts based on the within-sample quality and introduce the interactive outlier detection. Finally, we evaluate the proposed measures using simulated and real MRI data.

Segmentation-based Image Quality Assessment

For practical reasons, our QC framework uses the raw NIFTI format rather than the original DICOM format, as NIFTI images are more commonly available in public datasets and are more often used as input in data processing tools (Markiewicz et al., 2021). The QC framework relies on an existing conventional or deep-learning based classification of brain tissues, which is usually a prerequisite for subsequent brain image analyses (e.g., Ashburner et al., 2005, Gaser et al., 2024, Mendrik et al., 2015, Billot et al., 2023). All proposed measures are based on image properties primarily within the brain because the background might be affected by anonymization, noise or artifacts that do not necessarily affect the brain itself (Figure 2A; Kruggel et al., 2010; Marques et al., 2010). The quality measures are optimized to avoid the evaluation within parts of the brain that are typically affected by aging-related tissue changes, such as white matter hyperintensities, small vessel disease and perivascular spaces (Figure 2B). Within the CAT12 toolbox, the QC is the final step of the preprocessing and extends the processing time of a subject by only a few seconds. Alternatively, it can be run separately as an SPM batch for a pre-existing tissue segmentation, e.g., by SPM. ~~other algorithms.~~

The primary conceptualization and evaluation of our proposed QC measures is based on the *Brain Web Phantom* (**BWP**, AubertBroche et al., 2006), a simulated MRI dataset, which presents a well-established standard for developing and comparing processing methods. ~~for typical image properties such as noise, inhomogeneities, and resolution (Figure 1B).~~ The dataset includes images with varying levels of noise, inhomogeneities, and resolution. These image properties affect the segmentation accuracy of MRI processing algorithms (Figure 1B), and are therefore useful indicators of the quality of input data.

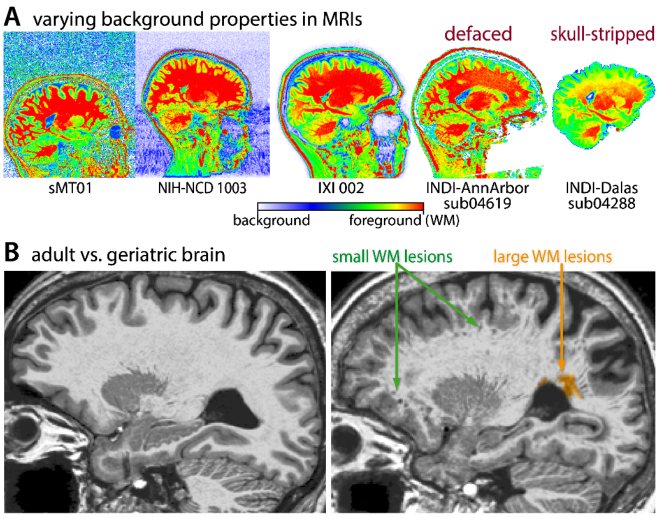

Figure 2: (A) Due to several different background types in real samples, only the brain tissues (excluding the background) were used for the evaluation of image quality. (B) To avoid side effects from age-related changes in volume and structure, the tissue segmentation is optimized to avoid tissue boundaries and perivascular spaces by morphological operations and masking.

Quality Measures

As our measures have been optimized for use in cognitive and clinical neuroscience studies, the presentation is focused on practicality. A full (technical) description can be found in the Methods section. For intensity-based measures, we use measure-to-contrast ratios instead of contrast-to-measure ratios. This approach ensures that the ratings follow a linear scale rather than a logarithmic one, as defined by the Brain Web Phantom. For a comparison to traditional contrast-to-measure ratio, please see the Method section.

Several key quality metrics are considered:

- **Noise-to-contrast ratio (NCR)**: This metric estimates image noise by calculating the lowest average local standard deviation of voxel intensities in the bias-corrected image. It is assessed within optimized cerebrospinal fluid (CSF) and white matter (WM) regions and is highly sensitive for other high-frequency artifacts such as motion.

- **Inhomogeneity-to-contrast ratio (ICR)**: This measure evaluates intensity variations across the image by calculating the global standard deviation of smoothed intensities within the optimized WM segment.
- **Resolution score (RES)**: To account for distortions due to anisotropic resolution, this score is directly computed using the root mean square (RMS) equation.
- **Edge-to-contrast ratio (ECR)**: Since resampling or smoothing can degrade voxel resolution, we suggest an additional measure which captures the average slope of intensity changes at the gray matter (GM)/WM boundary. This helps assess the sharpness of tissue interfaces.
- **Full-brain Euler characteristic (FEC)**: This metric quantifies the topological integrity of the WM brain interface, helping to detect potential distortions caused by noise and (motion) artifacts.

These measures provide a comprehensive assessment of structural MRI image quality, ensuring that intensity-based distortions, resolution issues, and structural inconsistencies that are relevant for the brain tissue segmentation, thickness estimation and surface reconstruction are identified and accounted for.

Standardization of Measures

Standardization into a normative range can enable simpler comparison across studies and support easier interpretations. To accommodate various international rating systems, we have adopted a linear percentage and a corresponding (alpha-)numeric scaling. (Figure 3, $QR_{percentage} = 105 - QR_{grade} * 10$, $QR_{grade} = (105 - QR_{percentage}) / 10$). The quality rating ranges from 0.5 (100 *rating points* (**rps**); grade A^{+}) to 10.5 (0 rps; grade F) for highest and lowest image quality, respectively. Numerical values provide a specific rating, whereas letters describe quality ranges, e.g., grade A describes values between 90 and 100 rps. Scaling of the quality measures was performed using half of the BWP dataset, while the other half was used for evaluation (see Evaluation Concept and Data).

Although the BWP does not include the simulation of motion artifacts, these are in general comparable to an increase of noise in the BWP dataset by 2 percentage points as demonstrated in the Result section. In our QC measures, this roughly corresponds to an increase of +1 grade or -10 rps compared to motion-free data. For improved (human) readability, we standardized all measures by applying a simple linear scaling function

$$QR_{grade} = \max(.5, \min(10.5 , (QM_{grade} - WQM_{grade}) / (BQM_{grade} - WQM_{grade}) * 6 + .5)) \quad (1)$$

to transform the original quality measure QM into a quality rating QR, with BQM as the best (95 rps, grade 1) and WQM (45 rps, grade 5) as the worst regular value.

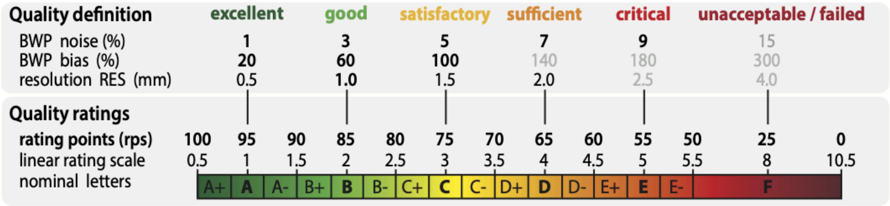

Figure 3: Quality rating system: The percentage, numerical, and character grades were scaled on the basis of the BWP, which represents a standard for evaluation of image processing methods. It should be noted that excellent ratings are reserved for images with exceptional quality, whereas typical scientific data generally receives "only" good assessments.

Integrated Structural Image Quality Rating

The structural image quality rating (SIQR) is defined using an exponentially weighted average of multiple quality scores QR_{grade} (see Equation 2 below). This single composite score integrates various aspects of image quality, providing a robust metric for assessing structural image quality and identifying potential outliers. We excluded the inhomogeneity score (ICR) from the composite SIQR measure because most preprocessing methods can handle bias quite well, and the effects of signal-intensity changes are already considered by the NCR. Integrating the ICR was therefore contraintuitive, as high field strength resulted in worse ratings that did not fit to the outcome.

SIQR = (*mean*([NCR, ~~ICR,~~ RES, ECR, FEC]^{4}))$^{(1/4)}$ (2)

To balance the sensitivity to different quality measures while ensuring that the necessary quality conditions are met, we apply an exponentially weighted averaging approach to the graded quality ratings (range 0.5 to 10.5) — similar to the root mean square (RMS) but using the fourth power and fourth root. This method allows well-rated images to contribute positively without overshadowing critical quality constraints. For more information regarding the weighting-selection process, we direct the reader to the Method section.

Sample Normalization for Outlier Detection

So far, each image has been evaluated individually, enabling the detection of outliers with very low resolution or high noise (e.g., those falling below a C rating, such as SIQR < 70 rps). However, to identify more subtle issues—such as mild motion artifacts—it is necessary to assess deviations from the ideal quality expected for a given MRI protocol within a specific sample. To achieve this, we estimate the upper quartile of the SIQR percentage scores from images acquired with the same protocol and apply a linear correction (a simple translation, as the values have already been scaled according to the BWP). This normalization results in a standardized SIQR, where values close to zero indicate optimal protocol quality, while higher values highlight potential outliers. To establish a general threshold for detecting quality issues, we employed a *receiver operating characteristic* (**ROC**) curve analysis combined with 2-fold cross-validation (splitting the dataset into odd- and even-numbered files based on filenames). This approach was validated using test samples with expert ratings, ensuring robust performance in identifying suboptimal images. The normalized scores were processed using the "*Check Sample Homogeneity*" tool (described in the next section) and saved as an nSIQR (normalized structural image quality rating) score in the subject's XML file, and in a CSV table.

Software

The "Check Sample Homogeneity" tool (Figure 4) in the CAT12 toolbox supports a guided analysis of large datasets to detect and exclude outliers in anatomy, preprocessing and image quality from analysis by estimating a sample-specific z-score. The tool has been designed in an interactive format with the intention to encourage users to get in touch with their data and carefully decide on the in-/exclusion of the images from analyses. Artifacts can result in a systematic bias, often resulting in an underestimation of GM (Reuter et al., 2015). To ensure the validity of statistical analyses, it is suggested that severe image quality-related outliers are excluded based on normative assessments provided by the toolbox. The quality estimation is also available as "*Image Quality Estimation*" SPM batch to process selected ~~raw (co-registered)~~ structural scans with a fitting brain tissue segmentation (e.g., from SPM). The results for each input image are stored in an XML file and can be used for subsequent analysis steps and potential analysis in relation to effects of interest of a study (such as age).

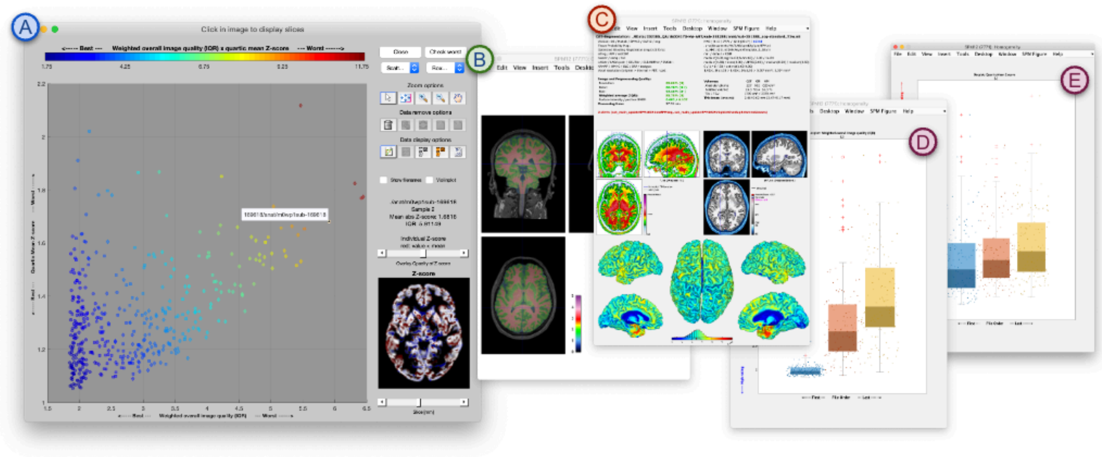

Figure 4: Shown is the "~~Check Covariance~~Check Sample Homogeneity" Tool in CAT12 for the MR-ART dataset grouped by the amount of motion (see D and E). A) In the main window, the user can select scans that are ready for analysis. The QC ratings, generated independently during preprocessing, are automatically loaded from the corresponding XML files. Users can interactively explore data points to investigate deviating ratings by viewing the original image with its segmentation overlay (B) or the preprocessing report (C) to remove outliers with image- and processing-related problems or atypical anatomical features. Grouping of multiple scans allows the estimation of a sample-specific normalised SIQR score (nSIQR) and z-scores that are added to the individual XML files and stored as a CSV table. The normalisation utilizes a scanning site variable to subtract the default protocol quality (defined by the upper quantil), highlighting scans with motion artifacts as outliers (F, see also figure 6 C and D).

Evaluation Concept and Data

The calibration and testing of our proposed measures was done using simulated images from the *Brain Web Phantom* (BWP; Cocosco et al., 1997, Collins et al., 1998, AubertBroche et al., 2006) and the *cortical aging phantom* (CAP; Rusak et al., 2022), as well as real data from IXI, ATLAS (Liev et al., 2018), MR-ART (Nárai et al., 2022), and a test-retest dataset (Table 1).

The BWP dataset consists of simulated data files of varying noise, inhomogeneities, and resolution parameters, which are encoded in the filenames. To create balanced and comprehensive calibration and testing samples containing similar—but not identical—cases, every second data point was assigned alternately to each sample. The BWP-calibration data consisted of all odd files (ordered

by filename) and were used to scale the quality measures and estimate the weighted averaging described above. The test subset (even files) was used to quantify the relationship between quality ratings and segmentation accuracy. Moreover, we used the BWP to further simulate typical brain-extraction and segmentation artifacts (e.g., by erosion/dilation of tissue segments) to test the robustness of the quality measures in case of critical data conditions (see Figure 5). The *cortical aging phantom* described in Rusak et al. (2022) was used to test the effects of brain atrophy of up to 1 mm on our quality measures.

Although simulated data enable basic evaluation under defined conditions, real data are essential to investigate possible dependencies/biases. The measures were quantified in IXI, ADHD200, and ATLAS datasets to test for possible effects of age, sex, and lesions. Finally, the MR-ART dataset with 148 subjects, each with 3 scans without, with light, and with severe motion artifacts, and available expert ratings as well as MRIQC derivatives (Esteban et al., 2017), was used to test the utility of our measures to separate images with motion artifacts, and to validate the measures against an established QC framework.~~in a split-half test design~~

Additionally, we used the Tohoku *test-retest* (**TRT**) dataset, which contains 126 T1-weighted scans (Thyreau et al., 2013). All scans were preprocessed, registered, and resliced to a high-resolution template with 0.50 mm isotropic resolution. A median template was used to remove outliers and to create the final ground truth segmentation by averaging. Finally, we estimated the association of image quality and scan time. ~~Finally, six scans were selected based on their scan time and image characteristics to evaluate the influence of image quality on segmentation accuracy.~~

All evaluation scripts are available in the CAT distribution on Github and require Matlab with the statistical toolbox to run. The required raw data of IXI, CAP, ADHD200, ATLAS, MR-ART are available from the project specific websites as described in Table 1. The preprocessed data and the reorganized images of the BWP(E) and the TRT are available on the GigaScience server (files.gigadb.org/dahnke2025_qc).

Table 1: Short overview of the used datasets.

Dataset	*n*	Age [years]	Sex [%men]	Sites	Description
BWP	600	~30	100%	1	Simulated dataset (AubertBroche et al., 2006; https://brainweb.bic.mni.mcgill.ca/) for basic definition (calibration: odd files) and evaluation (test: even files) of the quality ratings, with 5 levels of noise (1% to 9%), 3 different bias fields with 5 levels (20% to 100%), and 8 resolution levels with a voxel-length of 1 and 2 mm.
BWPE		~30	100%	1	BWP data with simulated skull-stripping and segmentation errors (see Figure 5) to test the robustness of our measures in case of severe processing problems.
CAP	400	39.1-78.3 (70.7±5.4)	50%	1	Simulated atrophy dataset (Rusak et al., 2022; https://doi.org/10.25919/4ycc-fc11) to test for side effects of GM tissue atrophy that correspond to aging of about 100 years.
IXI	554	20–86 (48.5±16.4)	44.8%	3	Brain aging sample to test the effects of age, brain size, and sex (only scans with complete phenotypic data; http://www.brain-development.org/).
ATLAS (R1.2)	304	NA	NA	11	T1 images of subjects with (masked/unmasked) lesions to test the stability in case of severe structural changes (Liev et al., 2018) (controlled access: https://fcon_1000.projects.nitrc.org/indi/retro/atlas.html).
ADHD200	491		52.9	7	T1 images of healthy subjects of the train dataset (site 2 of 8 is only in the test dataset). https://fcon_1000.projects.nitrc.org/indi/adhd200/
MR-ART	148*3	18-75 (30.0±12.8)	35.1%	1	Dataset without and with intended motion artifacts (Nárai et al., 2022; https://openneuro.org/datasets/ds004173/versions/1.0.2).
TRT	6 (127)	~30	100%	1	Various MR protocols with a scan time duration from 30 s to 11 minutes on a 3T Philips scanner (Thyreau et al., 2013). The scans were selected based on differences in scan time driven by resolution and parallel imaging (SENSE), while ensuring the similarity of other MRI parameters (see supplementary table S2)

Results

The quality scores were first evaluated on the simulated test data in order to determine the accuracy of interference quantification and to investigate how robust the measures are in cases of simulated segmentation problems and aging. Furthermore, we used the IXI, ADHD200, and ATLAS datasets to study the effects of aging, sex, brain size, and stroke lesion on our proposed measures of image quality. Additionally, we evaluated the ability to detect images with motion artifacts on the MR-ART dataset, tested the validity of our measures against the MRIQC 0.16.1 derivatives, and demonstrated the application in a test-retest scenario. All measures had been standardized (see Figure 3) and evaluated on the BWP before focusing on the averaged SIQR score. Of note, obvious subject/scan-specific motion artifacts generally increase the scans' rating for about 1 grade, which corresponds to a decrease of 10 rps (and +0.5 grade / -5 rps for light artifacts), in comparison to the typical rating achieved by the majority of scans of the same protocol (see Figure 7). Similarly to the method section, we focus here on the results pertaining to SIQR and refer the interested reader to the Methods section for a more detailed overview.

Simulated data

The evaluation on the BWP test dataset showed that most quality ratings have a very high correlation (Spearman's rho > .950, p < .001) with their corresponding perturbation and a very low correlation (Spearman's rho < |0.1|) with the other tested perturbations (see table in Figure 5A & C). This suggests considerable specificity of the proposed quality measures. The combined SIQR score also showed a very strong association with the segmentation quality kappa (Spearman's rho = –.916, p < .001) and brain tissue volumes (Spearman's $rho_{CSF/GM/WM}$ = –.729/–.647/.805, $p_{CSF/GM/WM}$ < .001) (Figure 5B). The root mean square errors (**RMSE**) between the expected and measured values of the SIQR were 3.133, 2.530, and 0.263 rps for the BWP testset, the BWP-derived segmentation error testset, and the cortical atrophy phantom, respectively (Figure 5D).

Most notable is the quantification of the image resolution, where the simple voxel-based resolution rating RES did not work well in interpolated data (i.e., as expected in 225 out of 625 cases), resulting in a lower correlation (Spearman's rho = .332) and a high RMSE of 8.207 rps. The edge-based resolution measure ECR, on the other hand, generally performed better (Spearman's rho = .585, p < .001), but was strongly affected by noise (Spearman's rho = .631, p < .001) and inhomogeneity (Spearman's rho = .158, p < .001) than other scores. ~~Overall, low quality data was typically found to have higher error rates than high quality images, i.e., the RMSE of the SIQR for images with <5% noise was 2.200 rps compared to 3.417 rps for images with >5% noise.~~ The tests with simulated segmentation errors suggested that NCR and ICR were extremely robust (Figure 5E), whereas ECR and especially FEC were quite sensitive to strong (i.e., 1 voxel) over-/underestimations of CSF and WM.

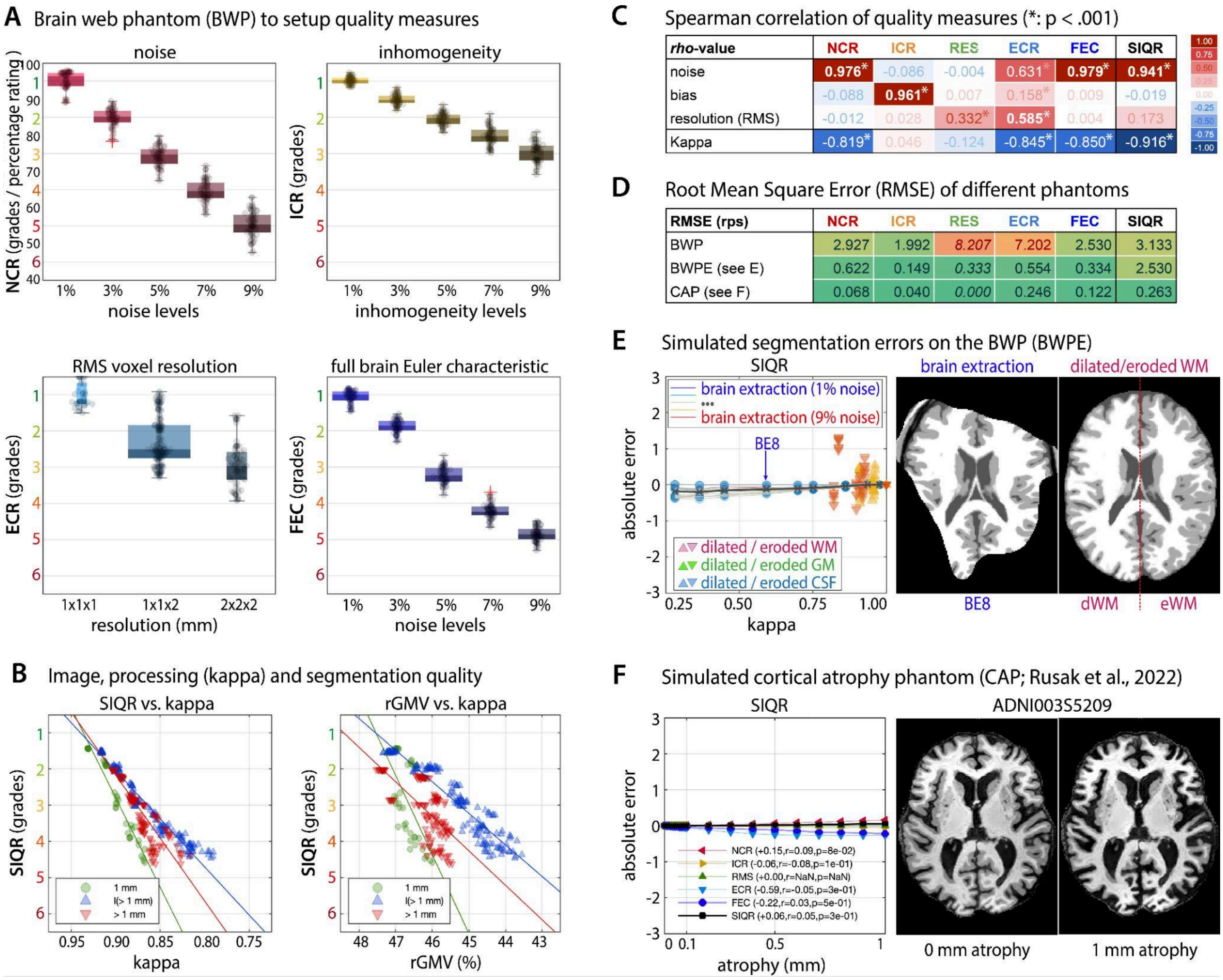

rho-value	NCR	ICR	RES	ECR	FEC	SIQR
noise	**0.976***	-0.086	-0.004	0.631*	**0.979***	**0.941***
bias	-0.088	**0.961***	0.007	0.158*	0.009	-0.019
resolution (RMS)	-0.012	0.028	0.332*	**0.585***	0.004	0.173
Kappa	-0.819*	0.046	-0.124	-0.845*	-0.850*	-0.916*

RMSE (rps)	NCR	ICR	RES	ECR	FEC	SIQR
BWP	2.927	1.992	*8.207*	7.202	2.530	3.133
BWPE (see E)	0.622	0.149	*0.333*	0.554	0.334	2.530
CAP (see F)	0.068	0.040	*0.000*	0.246	0.122	0.263

Figure 5: (A) Shown is the dependency of quality measures on manipulated levels of noise, inhomogeneity and resolution using the Brain Web Phantom (BWP): NCR (noise-to-contrast rating), ICR (inhomogeneity-to-contrast rating), ECR (edge-to-contrast rating), and FEC (full brain Euler characteristic). (B) The structural image quality rating (SIQR) integrates all these measures into one score and shows significant associations with segmentation quality (characterized by kappa) and the relative gray matter volume (rGMV) based on CAT12. (C) Overall, our ratings show specific relationships to their corresponding BWP perturbations but not others and (D) small root mean square errors (RMSE) also in the case of simulated segmentation errors (E) or aging (F). Of note, the numerical grading system and percentage system are inversely scaled, where –10 rps correspond to +1 grade and roughly correspond to the emergence of obvious motion artifacts.

* See the supplementary Table S1 for the full table in C.

Real data

The real data analysis of IXI and ATLAS cohorts suggests that the proposed quality measures were not affected by *total intracranial volume* (**TIV,** r_{SIQR}=.089, p_{SIQR}=.559), age (r_{SIQR}=-.187, p_{SIQR}=.079; Figure 6A), sex (Mann-Whitney-U-Test: U = 39125, Z=0.584, *p*=0.558), or stroke lesions in the ATLAS dataset (Figure 6B), while sex showed minor effects in IXI . Since IXI rather contains scans without significant motion artifacts, it provides a useful estimate of the typical overall variability in terms of the standard deviation of the SIQR score with 1.625 rps (Guys/HH/IOP = 1.629/1.606/1.641 rps). However, outliers with motion artifacts are often found in children, as shown in the ADHD200 dataset (Figure 6C) with an average standard deviation of the SIQR scores of 2.078 rps (Site 1,3-7 = 3.543/1.578/1.493/4.061/1.528/1.314/1.0273 rps).

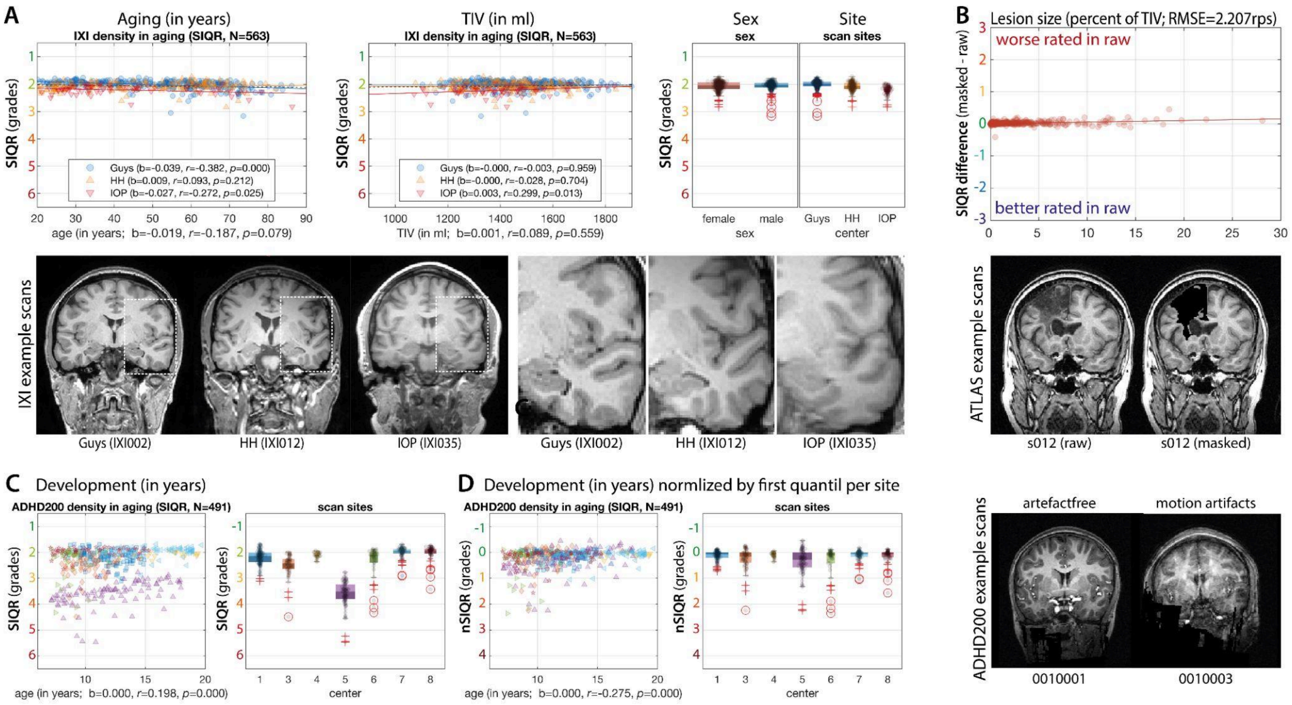

Figure 6: (A) The results of the structural dependency test in IXI dataset showed that the SIQR measure is independent from age and TIV, and only slightly associated with sex (see the Methods section for other quality measures) with an average standard deviation 1.625 rps per site. (B) The results from the ATLAS dataset suggest that severe structural changes in terms of lesions do not significantly affect the SIQR measure when comparing raw vs. masked images. (C) SIQR measurements in the ADHD200 dataset for children and young adults acquired by 7 different centers including scans with motion artifacts. (D) To identify outliers with motion artifacts (over multiple sites), a normalization by the typical protocol quality (defined by the first quantil of the SIQR values per site) can be used, where scans with more than 5/10 rps ratings typically have light/strong motion artifacts, respectively (see MR-ART dataset in figure 7). Note that our rating system is designed to assist in identifying cases that require further human evaluation, depending on the needs of the study.

The effects of motion artifacts were evaluated using the MR-ART dataset (Figure 7A)~~, which also allowed the comparison to the quality measures of MRIQC 0.16.1 (Esteban et al., 2017)~~. In order to detect motion artifacts, each score was normalized (by subtracting the first quartile value to consider the typical protocol quality) and a *Receiver Operating Characteristic* (**ROC**) was applied. The measures were tested under 3 conditions, namely comparing (i) no vs. severe artifacts, (ii) no vs. light+severe artifacts, and (iii) no+light vs. severe artifacts (Figure 7B). The best ROC thresholds

to separate good from bad scans in the 3 groups were 4.20/1.90/1.55 rps for the normalized SIQR ~~and standard deviations of 1.441 and 3.041 rps in the no artifact and no+light artifact groups, respectively~~. The accuracy of the SIQR as determined by the ROC (an average over the three groups) was 0.902 and 0.899, with an *area under curve* (**AUC**) of 0.974 and 0.969 for CAT12 and SPM12, respectively. The failure cases where the measure was not in accordance with the expert ratings are available in the supplementary material.

Moreover, we have demonstrated the expected decrease of GMV with aging and in relation to motion artifacts in the MR-ART dataset for CAT12 and SPM25 segmentation (Figures 7C-F). The results indicate that higher segmentation error (measured by the Kappa statistic comparing motion-free and motion-artefact-containing scans) and hence lower image quality may lead to underestimation of GM and overestimation of WM volume.

~~This was slightly better than the best performing alternative metrics from MRIQC measures especially *snr_wm*, *snr_total*, *cjv* and *cnr* (see Esteban et al., 2017) that achieved average accuracies over the three groups of 0.878/0.879/0.850/0.845 and an AUC of 0.961/0.952/0.937/0.930, respectively.~~ We further tested the association of our measures with the established measures from the MRIQC 0.16.1 (Esteban et al., 2017; see Nárai et al., 2022 for the processing). The results are presented for selected MRIQC measures in Figure 8. SIQR was highly associated with signal to noise ratio of MRIQC, especially that of white matter (rho = .927, p < .001), as well as summary standard deviation of the background (rho = -.937 , p < .001). For associations with all the measures from MRIQC see supplemental figure S6.

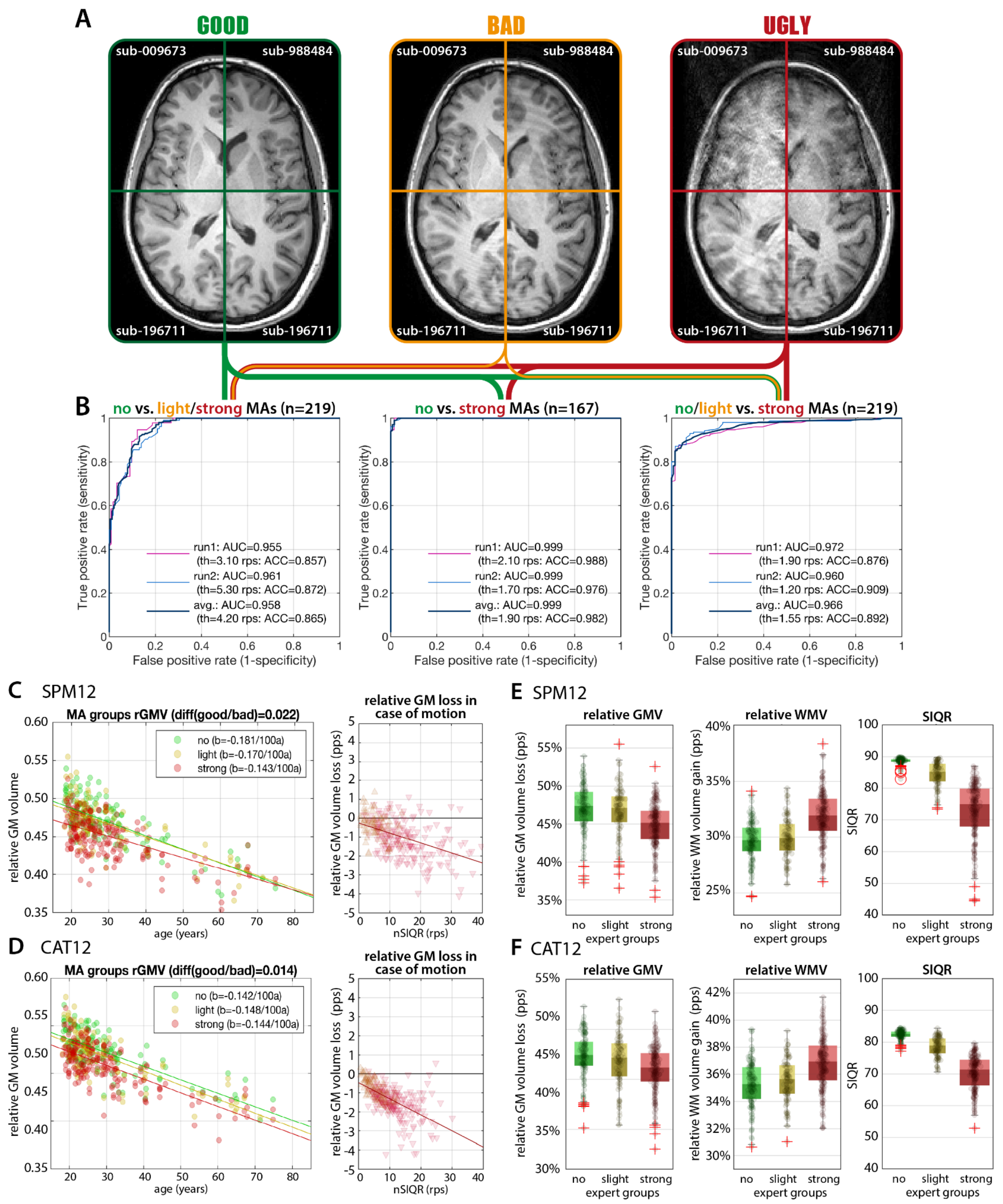

Figure 7: (A) Example images from the MR-ART dataset (Nárai et al., 2022) for three different conditions based on expert ratings splitting data into (no), (light), and (strong) motion artifacts (MAs). (B) Shows the Receiver Operating Characteristic (**ROC**) curves when classifying these groups using SIQR (see the Methods section for ROCs of other quality ratings) with high accuracy (**ACC**) and area under curve (**AUC**). The thresholds (th) of the ROC were estimated on the normalised SIQR values (nSIQR) and applied in a split-half design (run1 vs. run2).

We tested three options in handling cases with light MAs, where the no vs. strong test case was only possible in a smaller subsample (n=167), ignoring the controversial light cases, only 4 cases were misclassified (C; see the Supplement for the failed cases for the two grouping with light motion artifacts). To separate all motion cases, a threshold of about 5 rps worked best, whereas the separation of strong motion artifacts needed a higher threshold of about 7 to 9 rps. In other words, scans with a rating of 5 to 10 rps lower than the typical quality of the protocol should be checked. (C & D) We further evaluated the relative GM tissue volume change (in relation to aging) in percentage points (pps) of scans with motion artifacts compared to the ones without for each subject processed by SPM and CAT12. As the MR-ART dataset only includes motion-free and affected scans, we estimated the tissue changes in motion cases compared to the one without motion. The results suggest that the expected lower segmentation accuracy for lower image quality ~~might~~ leads to GM under- and WM overestimation, where motion artifacts of 10 rps are roughly comparable to a GM loss within 5 years. (E & F) The boxplot of the 3 expert defined motion groups clearly showed the expected GM underestimation, WM overestimation, and SIQR ratings in case of strong motion in SPM and CAT12.

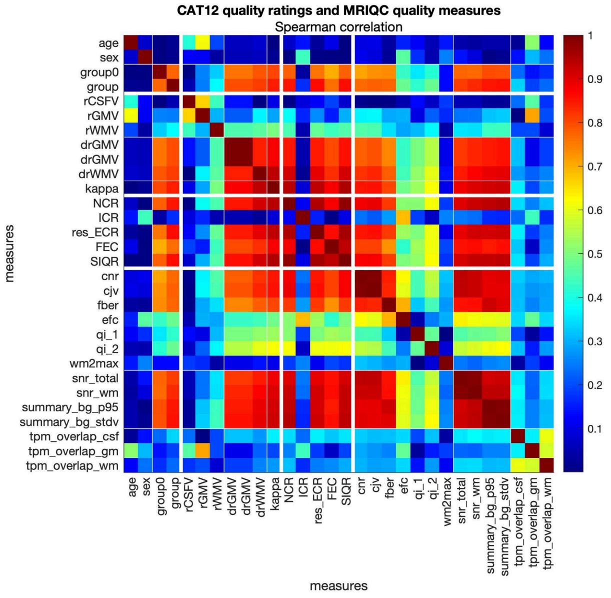

Figure 8: Spearman correlation coefficients between expert rating (i.e. expert rating group: 1 - no motion, 2 - light motion, 3 - severe motion), ΔV (dvol_rel_CGW, volume change in relation to motion-free scan), Kappa statistic (estimated w.r.t. the motion-free scan and averaged across all tissues), CAT12 quality ratings (NCR, ICR, res_ECR, FEC, and the weighted average SIQR), and selected MRIQC quality measures in MR-ART dataset (Nárai et al., 2022; for full table see supplemental figure S6).

Finally, we validated the proposed quality metrics using a scan-rescan test where we inspected the difference in quality scores and segmentation accuracy with regard to scanning time and ground truth image, respectively ~~including six images consisting of a series with gradually increased quality (and scan-time) as well as the ground truth image~~ (Figure 8). The expected improvement of image quality was clearly observable in terms of sharper anatomical details and reduced noise. The kappa indices, rGMVs, and SIQR scores confirmed these visual observations, but pointed out further interesting details. The noisy short-time scans S1 and S2 showed significantly lower kappa, worse SIQR ratings, and smaller GM volumes, whereas the other scans were highly comparable.

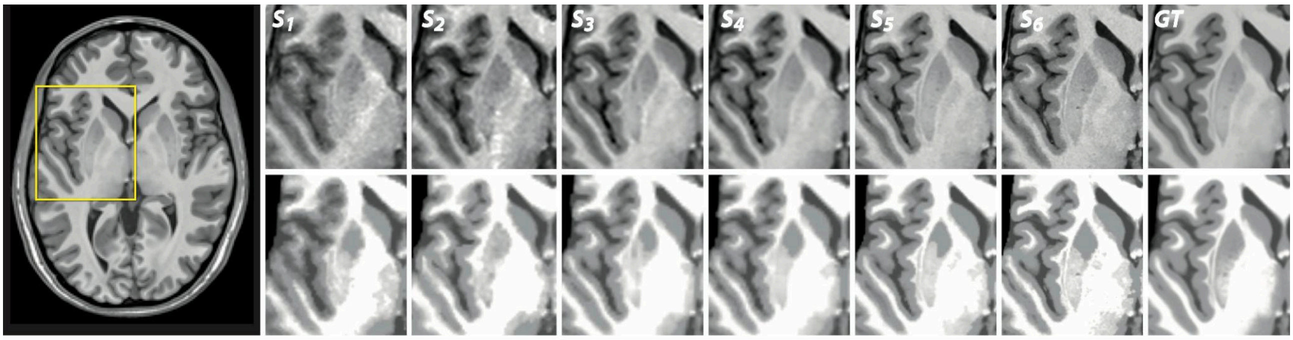

Image	S_1	S_2	S_3	S_4	S_5	S_6	GT
Duration (s)	36	53	119	197	369	611	-
Rx/Ry (mm):	1	1	1.25	1.25	0.75	0.50	0.50
Rz (mm):	2	2	1	1	1	1	0.50
Kappa (avg. (CSF,GM,WM)):	0.776	0.807	0.872	0.876	0.874	0.859	-
rGMV:	45.2 %	45.7%	45.8%	46.2%	46.4 %	45.3 %	45.2 %
SIQR:	69.27 (D$^+$)	74.65(C)	84.85(B)	85.29 (B)	85.52 (B$^+$)	82.56 (B$^-$)	83.15 (B$^-$)

Figure 9: An illustrative example of the test-retest sample analysis showing 6 images with an increasing scan-time and image quality. The top and bottom rows represent the intensity normalized T1 images and the CAT12 segmentation, respectively, with increasing segmentation accuracy in longer scan times ~~images/segmentations progressively converging (higher kappa)~~ when compared to the average ground truth (created from the best 127 test-retest images). The relative GM volume (rGMV) is slightly underestimated in low quality data.

Discussion

Here we introduced a QC framework for structural (T1-weighted) MRI data. We defined and validated various automated quality ratings based on well-defined BWP image quality features

such as noise, inhomogeneity, and resolution (Aubert-Broche et al., 2006) and integrated them into a single ~~useful~~ SIQR score to facilitate practical applications in context of clinical and cognitive neuroscience. We further demonstrated that our measures (i) are robust to simulated segmentation problems and cortical atrophy; (ii) are independent from sex, and brain size, showing only minor expected associations with chronological age, as well as severe disease-related changes; and (iii) allow the reliable assessment of motion artifacts within a protocol. In artifact-free data, image quality typically varies between 2.5 and 5 rps (0.25-0.5 grade), whereas light or strong artifacts typically result in a reduction of ratings by 5 or 10 rps (equivalent to a 0.5 or 1.0 increase in grades), respectively. T1-weighted images with low quality ratings might show a systematic underestimation of gray matter volume, first demonstrated in case of motion artifacts by Reuter et al. (2015). Strongly affected data should therefore be excluded from the analyses. In case of less severe artifacts, the quality rating might be included as a covariate (Garcia-Dias et al., 2020; Pomponio et al., 2019) or using weighted least squares (Lutti et al., 2022) during statistical analyses. However, an empirical comparison of the complex statistical effects of alternative approaches to account for automatically generated (i.e. known) quality differences in downstream analysis tasks is still lacking.

The proposed QC framework offers a simple and efficient approach to identify structural MRI scans that are suitable for the prospective use in structural processing tools (especially within SPM/CAT12 framework) and brain imaging analysis in both clinical and research settings. This was also confirmed in previous studies by Gilmore et al. (2021), Hoffstaedter et al. (2024) and Ma et al. (2022), that evaluated the utility of earlier versions of this QC framework.

In the following sections, we discuss further aspects of the development of our SIQR measure and its subordinate quality measures (with regard to existing alternatives), their performance in simulated and real data samples, and their potential for assessing the quality of images from other sequences and modalities.

SIQR measure development

Various image quality frameworks estimate some of their quality metrics based on information extracted from the image background (Esteban et al., 2017, Mortamet et al., 2009, Pizarro et al., 2016). In contrast, our approach focuses on estimating quality only within the brain for three reasons. First, the background values in public datasets can be corrupted by various defacing and skull-stripping routines (Bhalerao et al., 2022, Rubbert et al., 2022). Second, the background may contain artefacts (e.g., motion artefacts from the jaw or tongue) or unwanted properties (e.g., noisy backgrounds in MP2RAGE; Marques et al., 2010) that do not necessarily affect the brain, or conversely, the artefacts in the brain do not/are less likely to affect the background. Third, the background does not provide information about image inhomogeneity, tissue contrast and spatial anatomical resolution (Likar et al., 2001). While certain artefacts may be more prominently visible in the background (Mortamet et al., 2009), they are of interest to the user only if they also affect the brain. Furthermore, the evaluation of image quality within tissues must take into account structural aspects such as (i) the partial volume effect, where a voxel contains tissue of more than one tissue class, and (ii) changes in brain development and aging, such as tissue degeneration due to white matter hyperintensities, small vessel disease or perivascular spaces (Westlye et al., 2010; Lynch et al., 2024). Consequently, the proposed framework adapts these regions of interest by applying specific thresholds and morphological operations to minimize bias from age/disease, as we have demonstrated in IXI and ATLAS datasets. Moreover, the proposed intensity-based measures are normalised by (minimum) tissue contrast rather than signal intensity, as the separation between brain tissues, especially the GM and WM, is essential for segmentation and surface reconstruction (Fischl et al. 2012, Gaser et al., 2025).

Our proposed individual quality subscores have largely been established based on well-known image quality aspects of the BWP, which was built to represent the large variability in image quality of structural T1-weighted MR images (Aubert-Broche et al., 2006; Luo et al., 2022; Tönnes et al., 2024). By taking into account these predominant aspects of image quality, we have created

ratings that are easy to understand, even without a technical background. The ratings were integrated into a single SIQR rating to support the users during the evaluation process. To combine the measures, we have used an RMS-weighted average (of the grades) with a power of 4 rather than 2, to place greater emphasis on the more problematic aspects of image quality. This is relevant because effects of severe problems can often not be compensated by other factors, e.g. if there are severe motion artefacts, a much higher image resolution can typically not account for this.

In particular, SIQR is strongly predictive of segmentation accuracy (quantified by the kappa measure) and the extracted GM volume although its quantification is largely independent from structural features. Thus, SIQR can facilitate the estimation of image quality-related variance in individual scans or samples even for non-expert. Alternative quality control tools, such as MRIQC (Esteban et al., 2017), might be challenging for novices due to non-standardized measures that require substantial user experience. Moreover, a normalisation using BWP quality features also enables a direct comparison across protocols (see test-retest example ~~above~~), though caution is advised, as the results may be subject to bias by (i) our focus on a segmentation-centred definition of quality, (ii) the population under study and (iii) project-specific needs or considerations (e.g., optimised MR parameters to image specific structural changes rather than pre-processing).

Identification of scans with data anomalies and artifacts

The proposed framework is part of the CAT12 preprocessing and utilizes the CAT12 segmentation, but could also be used as an independent SPM batch with other segmentation algorithms, e.g. from SPM (Ashburner et al., 2005) ~~or SynthSeg (Billot et al., 2023)~~. Segmentation routines are widely used for structural brain analyses and have undergone intensive testing to be valid, accurate and robust for a variety of protocols, individual anatomies, and demographics (e.g., Ashburner et al., 2005, Mendrik et al., 2015, Gaser et al., 2024), making them ideal for image quality analysis. By focusing on general global aspects of the scan rather than local ones, problematic structures and areas such as partial volume effect voxels or WM lesions can be omitted, allowing precise, robust,

and largely consistent results even in case of severe classification faults (e.g., failed skull-stripping or miss-classification), as tested here under simulated conditions.

Although SIQR could be used for fully automatic outlier detection (see also Gilmore et al. (2021) and Bhalerao et al. (2024)), we believe that the huge variability of type of artefacts, their regional occurrence and their impact on image processing still require study-specific knowledge and, if possible, a short user inspection. For instance, in cases when locally limited or mild artefacts affect regions that are not relevant to the study (e.g., if the study focuses on frontal regions, cerebellar artefacts from jaw movements are acceptable) or whenever lower preprocessing accuracy is acceptable (e.g., for local alignment of brain surfaces or atlases for other modalities).

Multivariate outlier detection schemes that are typically applied based on the processed data of a sample in the normalized feature space, using similarity analysis of normalized GM data (e.g. the Gram matrix or kernels) in CAT12 (see Software section), can be used to detect outliers with preprocessing problems or highly deviating anatomy. However, the proposed image quality assessments are specifically designed to measure differences of image quality (in native space) rather than segmentation accuracy (in normalized space) or anatomical properties, such as stroke lesions, and can therefore be used in addition to previously mentioned outlier detection schemes to identify cases where image artifacts could bias analysis.

The role of subordinate quality ratings

While the SIQR composite is sufficient for most analyses, our framework facilitates deeper insights providing more specific subordinate ratings.

The *noise-to-contrast ratio* (**NCR**) is not only a very robust measure as it can be quantified in different regions, it is also very sensitive to motion artefacts and gives the most relevant values when the image resolution is adequate (<1.5 mm). ~~In our comparisons NCR supports a slightly better artifact separation than the best performing MRIQC measures, such as *snr_wm*, *snr_total*, *cjv* and *cnr*, which also evaluate noise differences.~~

The *inhomogeneity-to-contrast ratio* (**ICR**) had little to no impact on detecting problematic data (e.g., when testing for segmentation quality kappa in the BWP or motion artifacts in MR-ART), as inhomogeneities tend to describe more protocol/scanner specific aspects and can be corrected fairly well in most protocols (Belaroussi et al., 2006). Increased inhomogeneities typically occur in high-field scans without protocol specific correction schemes. Although possible disadvantages are generally outweighed by superior resolution and higher signal-to-noise ratio, bias correction schemes in preprocessing routines can fail in some cases (Feinberg et al., 2023). It is therefore recommended to retain this measure in a general rating.

The *resolution score* (**RES**) rates the voxel size in terms of how good structural features can be imaged. Nevertheless, structures can still be biased by interpolation (Tian et al., 2021), blurring, noise or motion artifacts. A real quantification of the sharpness of anatomical structures by our *edge-to-contrast ratio* (**ECR**) is therefore essential, although it is strongly affected by noise and the segmentation quality compared to other ratings.

The full-brain Euler characteristic (**FEC**) represents our adaptation of surface topology (Backhausen et al., 2016, Rosen et al., 2018). The measure showed strong association with noise levels and supports the identification of motion artifacts. However, compared to NCR it is more noisy and depends strongly on the input segmentation and MR protocol. Data with low spatial resolution or faulty/simplified segmentations with limited amount of details can have less defects and result in better ratings. Nevertheless, FEC presents a good extension to the NCR and ECR measurements.

In contrast to MRIQC (Esteban et al., 2017) that provides a variety of raw unscaled measures (with reversely signed scored ones among them), we tried to establish measures that reflect the known specific perturbances and are directly interpretable by applied scientists. Nevertheless, raw quality measures are also available in the XML files, allowing advanced users to perform detailed inspections. All QC measures can be used in statistical analyses or machine learning models according to the study needs (Bhalerao et al., 2024).

Evaluation in simulated and real data

Simulated data allow basic validation of methods under expected conditions and comparison with actual ground truth results. The BWP is a standard for evaluation of structural brain image preprocessing (Luo et al., 2022; Tönnes et al., 2024) and was used here to define and normalise our quality measures and to test their relationship with the segmentation accuracy. Due to the robustness of CAT12, we decided to simulate extreme segmentation problems. The results showed high stability for the NCR and ICR measures, but high susceptibility to error for the ECR and FEC in the case of severe over/underestimation of tissue segments, as both depend on the correct definition of the GM/WM boundary. In the simulated aging phantom (Rusak et al., 2022), the results showed small systematic but negligible changes in the quality measures, which could also be due to small differences in the simulated images (see bias differences in Figure 5F). Since a lot of our tests relied on BWP, which is limited in its ability to simulate artifacts or new protocols, such as MP2RAGE, new frameworks such as TorchIO (Pérez-García et al., 2021) present a possible next step for future tests. Nevertheless, an empirical validation on real MRI datasets was necessary to demonstrate the validity and practical benefits of the introduced quality assessments and to avoid over-adaptation to synthetic data. Therefore, we used the IXI and ATLAS datasets to demonstrate that SIQR is unaffected by age, sex, head size, or severe structural disease-related changes. In MR-ART, we tested the ability to identify different degrees of motion artifacts and the effects on gray matter segmentation in aging. Overall, we demonstrated the robustness and applicability of our SIQR measure.

Shortcomings and outlook

The QC measures proposed in this study were designed to be independent from segmentation accuracy and were tested in a variety of protocols. However, useful results can only be expected for valid segmentation inputs (where we focus on CAT12), and highly specific T1-weighted protocols

may result in unexpected ratings. Other modalities such as T2-weighted, proton density weighted or FLAIR images can be assessed, however, the dependance of the SIQR on separability of CSF, GM and WM may result in low quality scores. In addition, our ratings are not designed to assess functional or diffusion data where more specific tools are available (Christodoulou et al., 2013; Roalf et al., 2016; Nakua et al., 2023). Although such data can also be used for tissue segmentation, the low GM-WM contrast is challenging and the resulting segmentations or surfaces are less accurate and possibly biased compared to typical T1-weighted images (Ashburner et al., 2005). It is important to note that preprocessing tools are designed to work reliably even on problematic datasets, and that results from these images can often still be used, though these should be interpreted with more caution. Moreover, scanner-specific changes, such as geometric distortion, have not been considered. Consequently, our measures are not designed to monitor scanner properties that require real MRI phantoms (Belli et al., 2016; Davids et al., 2014).

In addition, our scan-rescan results demonstrated instances of comparable segmentation quality with up to 40% faster scan-times. This is particularly relevant for clinical MRI, where cost-effectiveness (short scan times for high patient throughput) presents an essential aspect, and images of adequate, but not exceptional, quality are appropriate for diagnosis (Jhaveri et al., 2015; Rofsky et al., 2015), simultaneously reducing both financial and environmental costs (Chaban et al., 2024). On the other hand, using only adequate image quality for certain projects does not eliminate the need for cutting-edge resolution (Feinberg et al., 2023), though improperly enhanced image resolution, e.g., 0.5×0.5×1.5 mm for 1.5 Tesla systems, often leads to increased noise or parallel imaging artifacts that can disturb preprocessing. It is therefore advisable to pilot modified protocols for the preprocessing pipelines you plan to use or follow the established standard protocols, e.g., ADNI (Arani et al., 2024) and HCP (Van Essen et al., 2013).

Conclusion

Our fully automatic quality control framework within the SPM/CAT12 ecosystem enables a standardized, accurate, and robust evaluation of large heterogeneous datasets to detect outliers

with inadequate image quality using a single image quality rating SIQR. Its flexibility, low-cost, and simplicity support a wide range of applications and can provide a valuable contribution to quality assurance in clinical practice and research.

Methods

All the statistical analyses were performed in MATLAB 2024a. The associations between different metrics were calculated using Spearman's rank correlation (unless stated otherwise), and Mann-Whitney U test was used to compare the quality metrics between men and women.

The following section provides a more technical introduction to our quality measures.

Normalisation and scaling

As segmentation as well as surface reconstruction rely heavily on the contrast between tissues, the normalization by contrast rather than the signal intensity allows a better correspondence between image and processing quality. Moreover, *contrast-to-measure ratios* rather than *measure-to-contrast ratios* were used as they support linear scaling to the interferences of the BWP and a linear relationship to the Kappa values of CAT (Figure 10).

Simple linear scaling function:

$$QR_{grade} = \beta(QM_{grade}, BQM_{grade}, WQM_{grade})$$

$$= \max(.5, \min(10.5 , (QM_{grade} - WQM_{grade}) / (BQM_{grade} - WQM_{grade}) * 6 + .5))$$

to transform the original quality measure QM into a quality rating QR, with BQM as the best (95 rps, grade 1) and WQM (45 rps, grade 5) as the worst regular value.

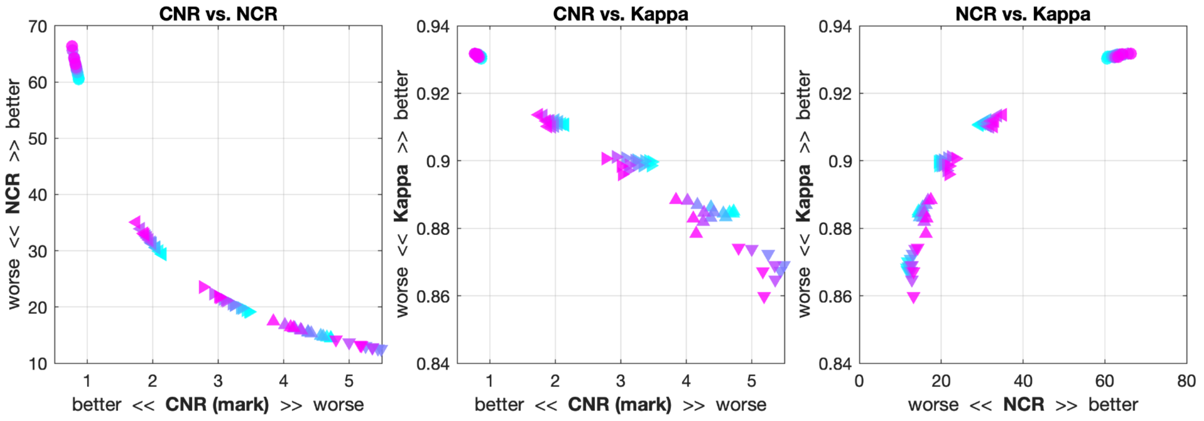

Figure 10: (A) Shown is the relation between the classical *noise-to-contrast ratio (NCR)* and contrast-to-noise-ration (CNR) (A) on the 1 mm BWP data with 1% to 9% noise and 20 to 100% bias. (B) The advantage of the CNR's linear scaling is clearly visible in its relationship with the preprocessing quality represented by Kappa value, where it allows for a better separation in the range of lower image and segmentation quality. (C) The nonlinear relation between NCR and Kappa statistic (average of all brain tissues segmented by CAT12), on the other hand, enables a finer separation of high-quality data, which is less useful for detection and quantification of outliers.

Noise

The first quality rating characterizes image noise defined here as *noise-to-contrast ratio* (**NCR**), to describe how well the tissue can be locally separated independent of the protocol-specific tissue contrast. The estimation was specified as the minimum of the average local standard deviation, $\tilde{\sigma}$, of the bias corrected image C_{bc} within the optimized *white matter* (**WM**) and *cerebrospinal fluid* (**CSF**) regions WMe and CSFe. The values were normalized by the minimum tissue contrast c_{min} and scaled by the results of the linear fit:

$$\text{NCR} = \beta(\, min(\, \tilde{\sigma}\,(C_{bc}(\text{CSFe})),\, \tilde{\sigma}(C_{bc}(\text{WMe})))\,)\, /\, c_{min}\,,\ 0.0183,\ \ 0.0868\,) \qquad \text{(SE1)}$$

with 0.0130 as the best, and 0.0682 as the worst rating of the unscaled measure obtained for the BWP train dataset. For data analysis, the bias corrected image C_{bc} allows for a more meaningful characterization of the local varying noise level than the original image, since it considers processing problems in areas with low signal intensity and increased noise. The local standard

deviation $\tilde{\sigma}$ was estimated in a 5x5x5 voxel neighborhood of a voxel and averaged to reduce the influence of remaining inhomogeneities.

The CSF and WM regions, rather than the background, were used because the background can contain interferences that do not affect the brain (Kruggel et al., 2010; Marques et al., 2010), or could be affected by anonymization of subject-features by defacing or brain extraction (Figure 2A). CSF and WM are beneficial for noise estimation compared to the *gray matter* (**GM**) because (i) they cover relatively large and homogenous areas and (ii) are less affected by partial volume effects and locally varying tissue contrast (e.g. by myelination). However, using only CSF regions often failed in younger subjects and low-resolution data, while the exclusive use of WM led to age-related effects caused by WM lesions or small vessel disease or perivascular spaces. The regions were optimized by an erosion step and additional tissue thresholds to avoid side effects by partial volumes, segmentation method, or WM lesions in elderly subjects that are quite similar to noise or artifacts (Figure 2B). The minimum tissue contrast c_{min} between CSF, GM, and WM was used because a greater GM-WM contrast led to problems in detecting the CSF-GM and CSF-background boundaries.

Inhomogeneity

In order to assess intensity inhomogeneity in images (often referred to as bias), the *coefficient of joint variation* (**CJV**; Likar et al., 2001) proved to be one of the most suitable measures (Belaroussi et al., 2006):

$$CJV = (\sigma(C_{GM}) + \sigma(C_{WM})) \ / \ | \mu(C_{GM}) + \mu(C_{WM})| \qquad \text{(SE2)}$$

However, since it is known that the GM is strongly influenced by partial volumes and locally different GM intensities (Westlye et al., 2010), only the standard deviation σ of the WM is determined here. Similar to the NCR, the minimal tissue contrast is used rather than the GM-WM contrast. To remove noise driven variance, a Laplacian filter with Dirichlet boundary condition is

applied in the WMe area, resulting in a locally averaged image Cs, which was used to estimate the *inhomogeneity-to-contrast ratio* (**ICR**):

$$\text{ICR} = \beta(\ \sigma(C_s(\text{WMe})\ )\ /\ c_{\min}\ , 0.2270,\ \ 1.3949\ ) \qquad \text{(SE3)}$$

Since most methods are able to correct strong inhomogeneities almost without loss of segmentation accuracy (e.g., approach X in Figure 1B; Belaroussi et al., 2006), a weaker weighting was used. The worst BWP inhomogeneity level describes a grade C (see Figure 3) that can be already measured in 3 Tesla data without protocol-based corrections.

Resolution

The spatial resolution of MRI images plays an important role in obtaining meaningful representations of anatomical structures. For the general assessment of voxel volume and proportion in a single value, we used the RMS notation to define the *RMS voxel resolution* (**RES**):

$$\text{RES} = \beta(\ (\ (x^2 + y^2 + z^2)\ /\ 3)^{1/2}, 0.5, 2.5) \qquad \text{(SE4)}$$

As a consequence of this definition, outliers with exceptionally low resolution in one of the three dimensions were weighted much higher than outliers with high resolution, resulting in an asymmetric evaluation where similar (isotropic) resolutions are preferred. The quality range was arbitrarily determined to characterize typical resolutions, with a simple scaling step size of 1 grade (10 rps) for another 0.5 mm, with 0.5 mm as an excellent result and 2.5 mm as the lowest quality limit close to the average cortical thickness in humans.

For the principal evaluation, we tested RES by reducing and re-interpolating the tissue label map of the BWP to quantify the loss of information by Cohen's kappa (Cohen, 1960). RES yielded higher Spearman correlation coefficient than the simple voxel mean RESM (rho_{RES} = 0.994; rho_{RESM} = 0.965; with RESM = β((x + y + z)/3, 0.5, 2.5). Although RES provides a good description of resolution under normal conditions, it has the major limitation that it does not quantify the true anatomical level of detail, i.e. how well fine structures are defined and how sharp the boundaries are.

The *edge-to-contrast ratio* (**ECR**) is the average gradient ∇ of the GM/WM boundary (outlined by the segmentation and masked for extreme gradients, e.g. between CSF and WM and blood vessels) and normalized by the minimum tissue contrast and scaled similarly to the RES rating. It allows an evaluation of structural resolution independent of resampling or smoothing.

$$\mathrm{ECR} = \beta(\nabla \mathrm{WM}, 0.0202, 0.1003) \quad \text{(SE5)}$$

To test the quantification of anatomical rather than image resolution, spatial details were removed by resampling (downsampling to 1.25:0.25:3.00 mm and resampling to 1.00 mm) and smoothing (0:0.25:3.00 mm) a BWP image with 1% noise, 20% inhomogeneity of field A, and 1 mm resolution. The parameter test range was defined by the resolution of the BWP, the minimum smoothing resolution (0.2 mm for 1.0 mm data) and the average cortical thickness of 2.5 mm. The resulting images were then segmented to quantify changes using Cohen's kappa. In both cases, the final voxel resolution remains constant, so that the voxel-based RMS resolution measurements are identical even though the images become blurred and kappa decreases (see Figure 11). In contrast, our new ECR measure allows quantification of both test cases, although quantification of GM/WM edge strength and tissue contrast introduces further variance ($r>0.98$, $p<7e-07$).

However, there are several limitations of the measure itself, but also of the test design: (i) the BWP is limited in its anatomical details, supporting only 1 mm resolution with some partial volume effect, (ii) linear/spline resampling and smoothing affect the measures differently, (iii) kappa only quantifies segmentation accuracy, but not the quality of more complex surface reconstruction (e.g. Hausdorff distance to the GT surface) that could be used. Nevertheless, ECR already represents a significant step forward in quantifying image detail in real data.

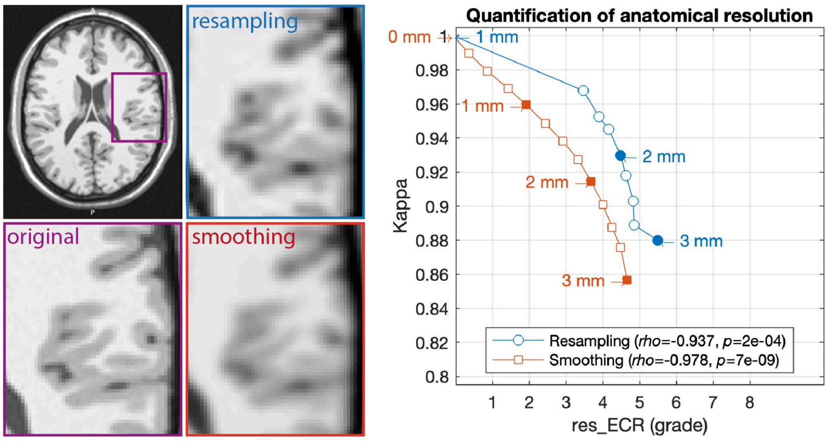

Figure 11: Shown are the effects of the simulated reduced resolution by resampling (downsampling to low resolution, followed by upsampling to original resolution) and Gaussian smoothing on the segmentation accuracy (quantified by kappa) for the *edge-to-contrast ratio* (**ECR**) resolution measure. Quantifying only by voxel resolution (RMS resolution score) would give the same value (grade 2 for 1 mm data) for all test cases (results for the BWP with 1% noise, 20% inhomogeneity field A and isotropic 1 mm resolution). It is also obvious that there is a large step between the original resolution and the first resampled resolution, which describes the general information loss of data resampling, and would be different in real data or if input data with higher ground truth resolution were used (i.e. test bias). In addition, quantification in low resolution data (below 2 mm) becomes increasingly difficult due to the highly folded and thin cortical band (sampling theorem).

Surface topology

In order to approximate the surface topology in a reasonable time, the *full-brain Euler characteristic* (**FEC**) was estimated at a resolution of 2 mm. The whole-brain WM surface was used rather than the typical neocortical hemispheres of most surface pipelines. To account for partial volume effects at the lower resolution, two WM surfaces were generated at thresholds of 0.25 and 0.75. As we observed more defects in children due to the thin developing WM structures, we used a maximum filter to extend and stabilize the surface creation.

$$FEC = \beta(EC , 130, 470) \qquad (SE6)$$

where the Euler characteristic EC is defined as EC = V - E + F with V as number of vertices, E as the number of edges, and F as the number of faces of the created brain surface.

Averaging

To obtain a meaningful composite measure of SIQR, we tested the mean, median, maximum, and four variants of the exponentially weighted averages with regard to their performance on the BWP and MR-ART dataset (Spearman's correlation between tissue volume/Kappa and SIQR). To quantify the effect of interferences, we estimated the volume difference ΔV and the Kappa value to the artifact-free case (averaged across all tissue classes), where volume changes and Kappa statistics should be highly associated with the quality rating (Table 2). We finally selected the power 4 function as it is more sensitive to outliers.

Acknowledgement

We want to thank Dr. Daniel Gülmar for his helpful comments, Benjamin Thyreau from the Tohoku University for the scan-rescan dataset, and all other projects providing publicly available data (even if not presented here). This manuscript reflects the views of the authors and may not reflect the opinions or views of the different projects.

IXI was made possible by the research grants of action charity, the Engineering and Physical Sciences Research Council (EPSRC GR/S21533/02), and the Medical Research Council.

Data and materials

MRI data is available by the original providers given in Table 1. The ATLAS dataset V1.2 (Liev et al., 2018) is under controlled access and users need to fill out an accessibility form to get the data

(https://goo.gl/forms/KwCljKSLWbbHWalD2). The scan-rescan dataset is not publicly available but can be requested from Benjamin Thyreau from the Tohoku University. The QC functions are part of the CAT12 toolbox (Gaser et al., 2024, https://neuro-jena.github.io/cat/).

Availability and requirements

- Project name: (Quality Metric of the) Computation Anatomy Toolbox (CAT)
- Project home page: https://neuro-jena.github.io/cat/
- Operating system(s): Linux, Mac OS, Windows
- Programming language: MATLAB™
- Other requirements: Statistical Parametric Mapping (SPM), R7771 https://www.fil.ion.ucl.ac.uk/spm/
- License: GNU GPL version 2 or higher
- RRID: SCR_019184
- Bio.tools ID: -

Processing was done under Mac OS using MATLAB 2024a, SPM25, CAT12.8.2 R2166-R2890 (segmentation) and CAT12.9 R2890 (quality control).

References

Ai, L., Craddock, R.C., Tottenham, N., Dyke, J.P., Lim, R., Colcombe, S., Milham, M., Franco, A.R., 2021. Is it time to switch your T1W sequence? Assessing the impact of prospective motion correction on the reliability and quality of structural imaging. NeuroImage 226, 117585. doi:10.1016/j.neuroimage.2020.117585

Arani, A., Borowski, B., Felmlee, J, Reid, R.I., Thomas, D.L., Gunter, J.L., Stables, L., Buckner, R.L., Jung, Y., Tosun, D., Weiner, M., Jack, Jr. C.R., for the Alzheimer's Disease Neuroimaging Initiative. Design and validation of the ADNI MR protocol. Alzheimer's Dement. 2024; 20: 6615–6621. https://doi.org/10.1002/alz.14162

Ashburner, J. and Friston, K. J. (2000). Voxel-based morphometry–the methods. *NeuroImage*, 11(6 Pt 1):805–821.

Ashburner, J. and Friston, K. J. (2005). Unified segmentation. *NeuroImage*, 26(3):839–851.

Aubert-Broche, B., Evans, A. C., and Collins, D. L. (2006). A new improved version of the realistic digital brain phantom. *NeuroImage*, 32(1):138–145.

Backhausen, L. L., Herting, M. M., Buse, J., Roessner, V., Smolka, M. N., & Vetter, N. C. (2016). Quality Control of Structural MRI Images Applied Using FreeSurfer-A Hands-On Workflow to Rate Motion Artifacts. Frontiers in neuroscience, 10, 558. https://doi.org/10.3389/fnins.2016.00558

Belaroussi, B., Milles, J., Carme, S., Zhu, Y. M., and Benoit-Cattin, H. (2006). Intensity non-uniformity correction in MRI: Existing methods and their validation. Medical Image Analysis, 10(2):234–246.

Belli, G., Busoni, S., Ciccarone, A., Coniglio, A., Esposito, M., Giannelli, M., Mazzoni, L. N., Nocetti, L., Sghedoni, R., Tarducci, R., Zatelli, G., Anoja, R. A., Belmonte, G., Bertolino, N., Betti, M., Biagini, C., Ciarmatori, A., Cretti, F., Fabbri, E., Fedeli, L., Filice, S., Fulcheri, C. P. L., Gasperi, C., Mangili, P. A., Mazzocchi, S., Meliadò, G., Morzenti, S., Noferini, L., Oberhofer, N., Orsingher, L., Paruccini, N., Princigalli, G., Quattrocchi, M., Rinaldi, A., Scelfo, D., Freixas, G. V., Tenori, L., Zucca, I., Luchinat, C., Gori, C., Gobbi, G., and Italian Association of Physics in Medicine (AIFM) Working Group on MR Intercomparison (2016). Quality assurance multicenter comparison of different MR scanners for quantitative diffusion-weighted imaging. *JMRI*, 43(1):213–219.

Bethlehem, R.A.I., Seidlitz, J., White, S.R., Vogel, J.W., Anderson, K.M., Adamson, C., Adler, S., Alexopoulos, G.S., Anagnostou, E., Areces-Gonzalez, A., Astle, D.E., Auyeung, B., Ayub, M., Bae, J., Ball, G., Baron-Cohen, S., Beare, R., Bedford, S.A., Benegal, V., Beyer, F., Blangero, J., Blesa Cábez, M., Boardman, J.P., Borzage, M., Bosch-Bayard, J.F., Bourke, N., Calhoun, V.D., Chakravarty, M.M., Chen, C., Chertavian, C., Chetelat, G., Chong, Y.-S., Cole, J.H., Corvin, A., Costantino, M., Courchesne, E., Crivello, F., Cropley, V.L., Crosbie, J., Crossley, N., Delarue, M., Delorme, R., Desrivieres, S., Devenyi, G.A., Di Biase, M.A., Dolan, R., Donald, K.A., Donohoe, G., Dunlop, K., Edwards, A.D., Elison, J.T., Ellis, C.T., Elman, J.A., Eyler, L., Fair, D.A., Feczko, E., Fletcher, P. C., Fonagy, P., Franz, C.E., Galan-Garcia, L., Gholipour, A., Giedd, J., Gilmore, J.H., Glahn, D.C., Goodyer, I.M., Grant, P.E., Groenewold, N.A., Gunning, F.M., Gur, R.E., Gur, R.C., Hammill, C.F., Hansson, O., Hedden, T., Heinz, A., Henson, R.N., Heuer, K., Hoare, J., Holla, B., Holmes, A.J., Holt, R., Huang, H., Im, K., Ipser, J., Jack, C.R., Jackowski, A.P., Jia, T., Johnson, K.A., Jones, P.B., Jones, D.T., Kahn, R.S., Karlsson, H., Karlsson, L., Kawashima, R., Kelley, E.A., Kern, S., Kim, K.W., Kitzbichler, M.G., Kremen, W.S., Lalonde, F.M., Landeau, B., Lee, S., Lerch, J., Lewis, J.D., Li, J., Liao, W., Liston, C., Lombardo, M.V., Lv, J., Lynch, C., Mallard, T.T., Marcelis, M., Markello, R.D., Mathias, S.R., Mazoyer, B., McGuire, P., Meaney, M.J., Mechelli, A., Medic, N., Misic, B., Morgan, S.E., Mothersill, D., Nigg, J., Ong, M.Q.W., Ortinau, C., Ossenkoppele, R., Ouyang, M., Palaniyappan, L., Paly, L., Pan, P.M.,

Pantelis, C., Park, M.M., Paus, T., Pausova, Z., Paz-Linares, D., Pichet Binette, A., Pierce, K., Qian, X., Qiu, J., Qiu, A., Raznahan, A., Rittman, T., Rodrigue, A., Rollins, C.K., Romero-Garcia, R., Ronan, L., Rosenberg, M.D., Rowitch, D.H., Salum, G.A., Satterthwaite, T.D., Schaare, H.L., Schachar, R.J., Schultz, A.P., Schumann, G., Schöll, M., Sharp, D., Shinohara, R.T., Skoog, I., Smyser, C.D., Sperling, R.A., Stein, D.J., Stolicyn, A., Suckling, J., Sullivan, G., Taki, Y., Thyreau, B., Toro, R., Traut, N., Tsvetanov, K.A., Turk-Browne, N.B., Tuulari, J.J., Tzourio, C., Vachon-Presseau, É., Valdes-Sosa, M.J., Valdes-Sosa, P.A., Valk, S.L., van Amelsvoort T, Vandekar, S.N., Vasung, L., Victoria, L.W., Villeneuve, S., Villringer, A., Vértes, P.E., Wagstyl, K., Wang, Y.S., Warfield, S.K., Warrier, V., Westman, E., Westwater, M.L., Whalley, H.C., Witte, A.V., Yang, N., Yeo, B., Yun, H., Zalesky, A., Zar, H.J., Zettergren, A., Zhou, J.H., Ziauddeen, H., Zugman, A., Zuo, X.N., 3R-BRAIN, AIBL, Alzheimer's Disease Neuroimaging Initiative, Alzheimer's Disease Repository Without Borders Investigators, CALM Team, Cam-CAN, CCNP, COBRE, cVEDA, ENIGMA Developmental Brain Age Working Group, Developing Human Connectome Project, FinnBrain, Harvard Aging Brain Study, IMAGEN, KNE96, Mayo Clinic Study of Aging, NSPN, POND, PREVENT-AD Research Group, VETSA, Bullmore, E.T., Alexander-Bloch, A.F., 2022. Brain charts for the human lifespan. Nature 604, 525–533. doi:10.1038/s41586-022-04554-y

Billot, B, Greve D.N., Puonti O., Thielscher A, Van Leemput K, Fischl B, Dalca A.V., and Iglesias J.E.. (2023). SynthSeg: Segmentation of brain MRI scans of any contrast and resolution without retraining, Medical Image Analysis, Volume 86, 2023, 102789, ISSN 1361-8415, https://doi.org/10.1016/j.media.2023.102789.

Bhalerao, G.V., Parekh, P., Saini, J., Venkatasubramanian, G., John, J.P., ADBS consortium, 2022. Systematic evaluation of the impact of defacing on quality and volumetric assessments on T1-weighted MR-images. J Neuroradiol 49, 250–257. doi:10.1016/j.neurad.2021.03.001

Bhalerao, G., Gillis, G., Dembele, M., Suri, S., Ebmeier, K., Klein, J., Hu, M., Mackay, C., Griffanti, L. 2024. Automated quality control of T1-weighted brain MRI scans for clinical research: methods comparison and design of a quality prediction classifier. medRxiv 2024.04.12.24305603; doi: https://doi.org/10.1101/2024.04.12.24305603

Bottani, S., Burgos, N., Maire, A., Wild, A., Ströer, S., Dormont, D., Colliot, O., APPRIMAGE Study Group, 2022. Automatic quality control of brain T1-weighted magnetic resonance images for a clinical data warehouse. Medical Image Analysis 75, 102219. doi:10.1016/j.media.2021.102219

Chaban, Y.V., Vosshenrich, J., McKee, H., Gunasekaran, S., Brown, M.J., Atalay, M.K., Heye, T., Markl, M., Woolen, S.A., Simonetti, O.P. and Hanneman, K. (2024). Environmental Sustainability and MRI: Challenges, Opportunities, and a Call for Action. J Magn Reson Imaging, 59: 1149-1167. https://doi.org/10.1002/jmri.28994

Christodoulou, A. G., Bauer, T. E., Kiehl, K. A., Feldstein Ewing, S. W., Bryan, A. D., & Calhoun, V. D. (2013). A quality control method for detecting and suppressing uncorrected residual motion in fMRI studies. Magnetic resonance imaging, 31(5), 707–717. https://doi.org/10.1016/j.mri.2012.11.007

Cocosco, C.A., Kollokian, V., Kwan, R.K.-S., Evans, A.C. (1997). *BrainWeb: Online Interface to a 3D MRI Simulated Brain Database*. NeuroImage, vol.5, no.4, part 2/4, S425, 1997 -- Proceedings of 3-rd International Conference on Functional Mapping of the Human Brain, Copenhagen, May 1997.

Cohen, J. (1960). A Coefficient of Agreement for Nominal Scales. *Educational and Psychological Measurement April 1960 vol. 20 no. 1 37-46*.

Collins, D.L., Zijdenbos, A.P., Kollokian, V., Sled, J.G., Kabani, N.J., Holmes, C.J., Evans, A.C. (1998). *Design and Construction of a Realistic Digital Brain Phantom*. IEEE Transactions on Medical Imaging, vol.17, No.3, p.463--468, June 1998.

Davids, M., Zöllner, F. G., Ruttorf, M., Nees, F., Flor, H., Schumann, G., Schad, L. R., and Consortium, t. I. (2014). Fully-automated quality assurance in multi-center studies using MRI phantom measurements. *MRI*, 32(6):771–780.

Esteban, O., Birman, D., Schaer, M., Koyejo, O.O., Poldrack, R.A., Gorgolewski, K.J., 2017. MRIQC: Advancing the automatic prediction of image quality in MRI from unseen sites. PLoS ONE 12, e0184661. doi:10.1371/journal.pone.0184661

Feinberg, D. A., Beckett, A. J. S., Vu, A. T., Stockmann, J., Huber, L., Ma, S., Ahn, S., Setsompop, K., Cao, X., Park, S., Liu, C., Wald, L. L., Polimeni, J. R., Mareyam, A., Gruber, B., Stirnberg, R., Liao, C., Yacoub, E., Davids, M., Bell, P., … Dietz, P. (2023). Next-generation MRI scanner designed for ultra-high-resolution human brain imaging at 7 Tesla. Nature methods, 20(12), 2048–2057. https://doi.org/10.1038/s41592-023-02068-7

Fischl B. (2012). FreeSurfer. NeuroImage, 62(2), 774–781. https://doi.org/10.1016/j.neuroimage.2012.01.021

Gaser, C., Dahnke, R., Thompson, P. M., Kurth, F., Luders, E., & The Alzheimer's Disease Neuroimaging Initiative (2024). CAT: a computational anatomy toolbox for the analysis of structural MRI data. GigaScience, 13, giae049. https://doi.org/10.1093/gigascience/giae049

Garcia-Dias, R., Scarpazza, C., Baecker, L., Vieira, S., Pinaya, W.H.L., Corvin, A., Redolfi, A., Nelson, B., Crespo-Facorro, B., McDonald, C., Tordesillas-Gutiérrez, D., Cannon, D., Mothersill, D., Hernaus, D., Morris, D., Setién-Suero, E., Donohoe, G., Frisoni, G., Tronchin, G., Sato, J., Marcelis, M., Kempton, M., van Haren, N.E.M., Gruber, O., McGorry, P., Amminger, P., McGuire, P., Gong, Q., Kahn, R.S., Ayesa-Arriola, R., van Amelsvoort, T., Ortiz-García de la Foz, V., Calhoun, V., Cahn, W., Mechelli, A., 2020. Neuroharmony: A new tool for harmonizing volumetric MRI data from unseen scanners. NeuroImage 220, 117127. doi:10.1016/j.neuroimage.2020.117127

Gilmore, A. D., Buser, N. J., Hanson, J. L. (2021). Variations in structural MRI quality significantly impact commonly used measures of brain anatomy. *Brain Inf*. 2021;8(1):7-15. doi:10.1186/s40708-021-00128-2.

Hendriks, J., Mutsaerts, H. J., Joules, R., Peña-Nogales, Ó., Rodrigues, P. R., Wolz, R., Burchell, G. L., Barkhof, F., & Schrantee, A. (2024). A systematic review of (semi-)automatic quality control of T1-weighted MRI scans. Neuroradiology, 66(1), 31–42. https://doi.org/10.1007/s00234-023-03256-0

Jhaveri, K. (2015). Image quality versus outcomes. *JMRI*, 41(4):866–869.

Kempton, M. J., Underwood, T. S. A., Brunton, S., Stylios, F., Schmechtig, A., Ettinger, U., Smith, M. S., Lovestone, S., Crum, W. R., Frangou, S., Williams, S. C. R., and Simmons, A. (2011). A comprehensive testing protocol for MRI

neuroanatomical segmentation techniques: Evaluation of a novel lateral ventricle segmentation method. *NeuroImage*, 58(4):1051–1059.

Keshavan, A., Datta, E., M McDonough, I., Madan, C.R., Jordan, K., Henry, R.G., 2018. Mindcontrol: A web application for brain segmentation quality control. NeuroImage 170, 365–372. doi:10.1016/j.neuroimage.2017.03.055.

Kruggel, F., Turner, J., Muftuler, L. T., and Initiative, A. D. N. (2010). Impact of scanner hardware and imaging protocol on image quality and compartment volume precision in the ADNI cohort. *NeuroImage*, 49(3):2123–2133.

Liew, S.L.., Anglin, J., Banks, N. et al. A large, open source dataset of stroke anatomical brain images and manual lesion segmentations. Sci Data 5, 180011 (2018). https://doi.org/10.1038/sdata.2018.11

Likar, B., Viergever, M. A., and Pernus, F. (2001). Retrospective correction of MR intensity inhomogeneity by information minimization. IEEE Transactions on Medical Imaging, 20(12):1398–1410.

Luo, Y., Zhou, L., Zhan, B., Fei, Y., Zhou, J., Wang, Y., & Shen, D. (2022). Adaptive rectification based adversarial network with spectrum constraint for high-quality PET image synthesis. Medical image analysis, 77, 102335. https://doi.org/10.1016/j.media.2021.102335

Lutti, A., Corbin, N., Ashburner, J., Ziegler, G., Draganski, B., Phillips, C., Kherif, F., Callaghan, M. F., & Di Domenicantonio, G. (2022). Restoring statistical validity in group analyses of motion-corrupted MRI data. Human brain mapping, 43(6), 1973–1983. https://doi.org/10.1002/hbm.25767

Lynch, K. M., Sepehrband, F., Toga, A. W., & Choupan, J. (2023). Brain perivascular space imaging across the human lifespan. NeuroImage, 271, 120009. https://doi.org/10.1016/j.neuroimage.2023.120009

Markiewicz, C.J., Gorgolewski, K.J., Feingold, F., Blair, R., Halchenko, Y.O., Miller, E., Hardcastle, N., Wexler, J., Esteban, O., Goncavles, M., Jwa, A., Poldrack, R., 2021. The OpenNeuro resource for sharing of neuroscience data. doi:10.7554/eLife.71774

Ma, Z., Reich, D.S., Dembling, S., Duyn, J.H., Koretsky, A.P., 2022. Outlier detection in multimodal MRI identifies rare individual phenotypes among more than 15,000 brains. HBM 43, 1766–1782. doi:10.1002/hbm.25756

Marques, J. P., Kober, T., Krueger, G., van der Zwaag, W., Van de Moortele, P.-F., and Gruetter, R. (2010). MP2RAGE, a self bias-field corrected sequence for improved segmentation and T1-mapping at high field. *NeuroImage*, 49(2):1271–1281.

Mendrik, A. M., Vincken, K. L., Kuijf, H. J., Breeuwer, M., Bouvy, W. H., de Bresser, J., Alansary, A., de Bruijne, M., Carass, A., El-Baz, A., Jog, A., Katyal, R., Khan, A. R., van der Lijn, F., Mahmood, Q., Mukherjee, R., van Opbroek, A., Paneri, S., Pereira, S., Persson, M., Rajchl, M., Sarikaya, D., Smedby, Ö., Silva, A. C., Vrooman, H. A., Vyas, S., Wang, C., Zhao, L., Biessels, G. J., and Viergever, M. A. (2015). MRBrainS Challenge: Online Evaluation Framework for Brain Image Segmentation in 3T MRI Scans. *Comput Intell Neurosci*, 2015(4-5):813696–16.

Mortamet, B., Bernstein, M. A., Jack Jr., C. R., Gunter, J. L., Ward, C., Britson, P. J., Meuli, R., Thiran, J.-P., Krueger, G., and Initiative, A. D. N. (2009). Automatic quality assessment in structural brain magnetic resonance imaging. *MRI*, 62(2):365–372.

Mu, Y., Li, Q., & Zhang, Y. (2019). White Matter Segmentation Algorithm for DTI Images Based on Super-Pixel Full Convolutional Network. Journal of medical systems, 43(9), 303. https://doi.org/10.1007/s10916-019-1431-1

Nakua, H., Hawco, C., Forde, N. J., Joseph, M., Grillet, M., Johnson, D., Jacobs, G. R., Hill, S., Voineskos, A. N., Wheeler, A. L., Lai, M. C., Szatmari, P., Georgiades, S., Nicolson, R., Schachar, R., Crosbie, J., Anagnostou, E., Lerch, J. P., Arnold, P. D., & Ameis, S. H. (2023). Systematic comparisons of different quality control approaches applied to three large pediatric neuroimaging datasets. NeuroImage, 274, 120119. https://doi.org/10.1016/j.neuroimage.2023.120119

Nárai, Á., Hermann, P., Auer, T., Kemenczky, P., Szalma, J., Homolya, I., Somogyi, E., Vakli, P., Weiss, B., Vidnyánszky, Z., 2022. Movement-related artefacts (MR-ART) dataset of matched motion-corrupted and clean structural MRI brain scans. Sci. Data 9, 1–6. doi:10.1038/s41597-022-01694-8

Pérez-García, F., Sparks, R., & Ourselin, S. (2021). TorchIO: A Python library for efficient loading, preprocessing, augmentation and patch-based sampling of medical images in deep learning. Computer methods and programs in biomedicine, 208, 106236. https://doi.org/10.1016/j.cmpb.2021.106236

Pizarro, R. A., Cheng, X., Barnett, A., Lemaitre, H., Verchinski, B. A., Goldman, A. L., Xiao, E., Luo, Q., Berman, K. F., Callicott, J. H., Weinberger, D. R., & Mattay, V. S. (2016). Automated quality assessment of structural magnetic resonance brain images based on a supervised machine learning algorithm. Frontiers in Neuroinformatics, 10, 52. https://doi.org/10.3389/fninf.2016.00052

Pomponio, R., Erus, G., Habes, M., Doshi, J., Srinivasan, D., Mamourian, E., Bashyam, V., Nasrallah, I.M., Satterthwaite, T.D., Fan, Y., Launer, L.J., Masters, C.L., Maruff, P., Zhuo, C., Völzke, H., Johnson, S.C., Fripp, J., Koutsouleris, N., Wolf, D.H., Gur, R., Gur, R., Morris, J., Albert, M.S., Grabe, H.J., Resnick, S.M., Bryan, R.N., Wolk, D.A., Shinohara, R.T., Shou, H., Davatzikos, C., 2020. Harmonization of large MRI datasets for the analysis of brain imaging patterns throughout the lifespan. NeuroImage 208, 116450. doi:10.1016/j.neuroimage.2019.116450

Reuter, M., Tisdall, M. D., Qureshi, A., Buckner, R. L., van der Kouwe, A. J. W., and Fischl, B. R. (2015). Head motion during MRI acquisition reduces gray matter volume and thickness estimates. *NeuroImage*, 107:107–115.

Roalf, D. R., Quarmley, M., Elliott, M. A., Satterthwaite, T. D., Vandekar, S. N., Ruparel, K., Gennatas, E. D., Calkins, M. E., Moore, T. M., Hopson, R., Prabhakaran, K., Jackson, C. T., Verma, R., Hakonarson, H., Gur, R. C., and Gur, R. E. (2016). The impact of quality assurance assessment on diffusion tensor imaging outcomes in a large-scale population-based cohort. NeuroImage, 125:903–919.

Rofsky, N. M. (2015). The importance of image quality: in the eyes of the beholder? *JMRI*, 41(4):861–865.

Rosen A.F.G., Roalf D.R., Ruparel K., Blake J., Seelaus K., Villa L.P., Ciric R., Cook P.A., Davatzikos C., Elliott M.A., Garcia de La Garza A., Gennatas E.D., Quarmley M., Schmitt J.E., Shinohara R.T., Tisdall M.D., Craddock R.C., Gur R.E., Gur R.C.,

Theodore D. Satterthwaite T.D., (2018). Quantitative assessment of structural image quality, NeuroImage, Volume 169, Pages 407-418, ISSN 1053-8119, https://doi.org/10.1016/j.neuroimage.2017.12.059.

Rusak, F., Santa Cruz, R., Lebrat, L., Hlinka, O., Fripp, J., Smith, E., Fookes, C., Bradley, A. P., Bourgeat, P., & Alzheimer's Disease Neuroimaging Initiative (2022). Quantifiable brain atrophy synthesis for benchmarking of cortical thickness estimation methods. Medical image analysis, 82, 102576. https://doi.org/10.1016/j.media.2022.102576

Rubbert, C., Wolf, L., Turowski, B., Hedderich, D.M., Gaser, C., Dahnke, R., Caspers, J., Alzheimer's Disease Neuroimaging Initiative, 2022. Impact of defacing on automated brain atrophy estimation. Insights Imaging 13, 54–11. doi:10.1186/s13244-022-01195-7

Tönnes, C., Licht, C., Schad, L. R., & Zöllner, F. G. (2023). VirtMRI: A Tool for Teaching MRI. Journal of medical systems, 47(1), 110. https://doi.org/10.1007/s10916-023-02004-4

Thyreau, B., Taki, Y., Yokota, S., Hashizume, H., and Kawashima, R. (2013). Practical impact of MRI parameters on the VoxelBased-Morphometry measures in SPM. In HBM, editor, *HBM*, Seattle. HBM.

Tian, Q., Bilgic, B., Fan, Q., Ngamsombat, C., Zaretskaya, N., Fultz, N.E., Ohringer, N.A., Chaudhari, A.S., Hu, Y., Witzel, T., Setsompop, K., Polimeni, J.R., Huang, S.Y., 2021. Improving in vivo human cerebral cortical surface reconstruction using data-driven super-resolution. Cereb Cortex 31, 463–482. doi:10.1093/cercor/bhaa237

Van Essen DC, Smith SM, Barch DM, et al. The WU-Minn Human Connectome Project: an overview. *NeuroImage*. 2013;80:62-79. doi:10.1016/j.neuroimage.2013.05.041.

Westlye, L. T., Walhovd, K. B., Dale, A. M., Bjørnerud, A., Due-Tønnessen, P., Engvig, A., Grydeland, H., Tamnes, C. K., Ostby, Y., and Fjell, A. M. (2010). Differentiating maturational and aging-related changes of the cerebral cortex by use of thickness and signal intensity. *NeuroImage*, 52(1):172–185.

Wiseman, S.J., Meijboom, R., Valdés-Hernández, M.D.C., Pernet, C., Sakka, E., Job, D., Waldman, A.D., Wardlaw, J.M., 2019. Longitudinal multi-centre brain imaging studies: guidelines and practical tips for accurate and reproducible imaging endpoints and data sharing. Trials 20, 1–10. doi:10.1186/s13063-018-3113-6

Data and Code Availability

Data used in the preparation of this manuscript are publicly available (although registration and approval may be required), and via the GigaScience FTP server (files.gigadb.org/dahnke2025_qc). The framework is a part of the CAT12 toolbox (https://neuro-jena.github.io//cat). The code used to generate the results and figures is available in the *catQC* subdirectory.

Ethics Statement

The study relies on publicly available datasets that were collected by complying to ethical standards.

Consent for publication

Not applicable

Competing interests

All authors declare that they have no conflicts of interest.

Funding

This work was funded by the *Deutsche Forschungsgemeinschaft* (DFG) Nr. 417649423 and grant 351849 from the Research Council of Finland under the frame of ERA PerMed ("Pattern-Cog").

Authorship Contributions

Robert Dahnke: Methodology, Software, Validation, Formal analysis, Investigation, Resources, Data Curation, Writing - Original Draft, Writing - Review & Editing, Visualization, Project administration. **Polona Kalc:** Writing - Review & Editing. **Gabriel Ziegler:** Writing - Review & Editing. **Julian Großkreutz:** Writing - Review & Editing, Funding acquisition. **Christian Gaser:** Methodology, Software, Writing - Review & Editing, Supervision, Project administration, Funding acquisition.

Supplementary data

Detailed scaling, normalization, and averaging

The individual image quality measures were averaged to create the SIQR composite measure. For this, we tested multiple options of weighting the individual measures. Since image artifacts (e.g., motion artifacts) cannot be compensated by other positive factors of image quality, we focused on testing a root-mean-square-error-like (exponential) weighting of different power to better separate the outliers. We observed that the simple averaging of quality measures was not as strongly impacted in case of strong motion artifacts. We therefore decided for a higher power weighting to increase the impact of single bad ratings on the composite score (Figure S1). The effect of motion artifacts on image analyses is mostly driven by the outcome of the preprocessing, which we quantified in the BWP (by using Kappa statistics) and in the MR-ART dataset as a systematic underestimation of GM volume. Based on our tests, we finally selected a weighting power of 4 as our tests indicated higher correlations to Kappa and sensitivity to tissue changes. Overall, the performance of SQIR depends on segmentation, QC-version, and included ratings. Excluding the voxel-size-based resolution rating (res_RMS, orange line on the graph) gives advantages in the interpolated BWP cases, whereas ignoring the Euler number results in lower correlation. In real data, the exclusion of the NCR results in lower correlation.

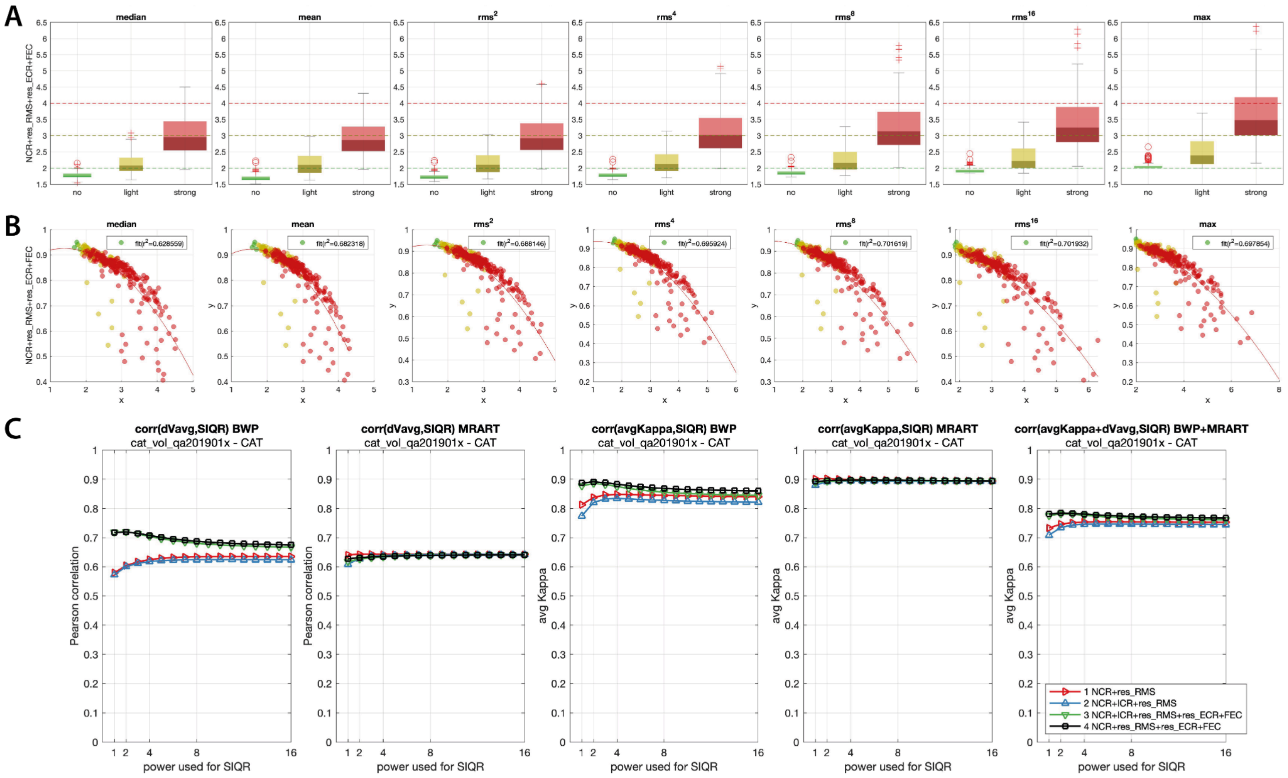

Figure S1: (A) Shown are boxplots of different averaging functions on the expert grouping of the MR-ART dataset. Higher powers allow the outliers stronger and result in more appropriate ratings. **(B)** Higher powers also result in a more linear relationship between the image quality and segmentation quality Kappa (estimated for all scans with the motion artifacts of a subject in relation to their motion-free scan). **(C)** Pearson's correlation plots of different combined ratings with different powers and the average volume loss (subplot 1 on the BWP and 2 on MR-ART) and Kappa loss (subplot 3 on the BWP and 4 on MR-ART), and for a simple mean of volume and Kappa loss in both cases (subplot 5). The black line includes all measures; the colored lines present all cases while leaving out one of the measures. The combination of *NCR+res_RMS* represented our classical IQR rating that was now extended by the *edge-constrast ratio* (res_ECR) and the *fast Euler characteristic* (FEC). In both cases, we observed that the *inhomogeneity-to-contrast ratio* (ICR) was contraindicative in describing the image quality, as higher scan quality is mostly driven by low noise and high resolution that are generally better in high-field scans, whereas the higher bias can be corrected quite well by state-of-the-art approaches such as SPM or CAT12.

Detailed BWP Results

As CAT produced highly accurate segmentations even in problematic BWP cases, we simulated severe segmentation errors to test the robustness of our measures in exceptional situations. We simulated problems in skull stripping and tissue over/underestimation in 15 BWP cases with 1 to 9% noise, 1 mm resolution, and 20% inhomogeneity of 3 fields. To test the influence of preprocessing problems, we simulated severe distortions due to skull-stripping (i.e., missing brain/additional head) and tissue segmentation errors (e.g., WM overestimation/underestimation). To simulate inaccurate skull-stripping, a strongly smoothed map of random values and distance information was used to add/remove tissues to the segmentation. We then modified the tissue boundaries by eroding/dilating the tissue segments, using a maximum/minimum filter to preserve the partial volume effect (dWM, eWM, eCSF, dCSF, eWM & eCSF). Although these changes do not affect the kappa values as much as skull stripping, the changes in GM volume are extreme and comparable to thickness changes of approximately 1 mm (Figure S2B). In all cases, the variance of the measures was positively correlated with the noise of the BWP, i.e. quantification of noisy images is again more error-prone. Nevertheless, most of our measures are quite robust even to severe errors with kappa values below 0.5. Finally, this also means that our image quality measures do not (directly) quantify the quality of the preprocessing itself (i.e., segmentation accuracy). However, given that lower image quality leads to lower processing quality, it can be assumed that processing accuracy is generally lower for low quality input and that further user intervention is required (e.g., to check and remove problematic scans).

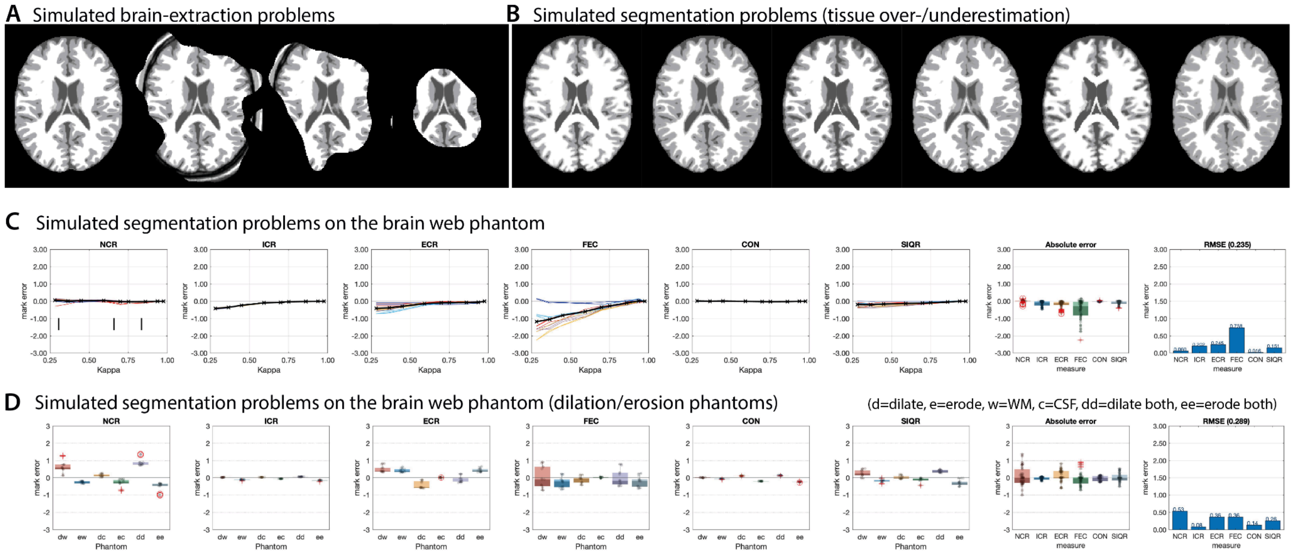

Figure S2: We simulated the effects of segmentation accuracy for brain extraction problems (A) and for tissue over- and underestimation (B). Effects of skull-stripping problems are shown as lines in C, while the segmentation problems are shown as scatterplot. Our quality scores are almost unaffected by severe skull stripping problems (kappa < 0.6), as even small regions are sufficient for global estimation. Tissue classification errors are more challenging, even if the reduced accuracy is quite small, because the contrast estimation is biased. In general, problematic cases tend to underestimate the image quality, which indirectly helps to identify serious pre-processing problems.

Detailed Aging Phantom Results

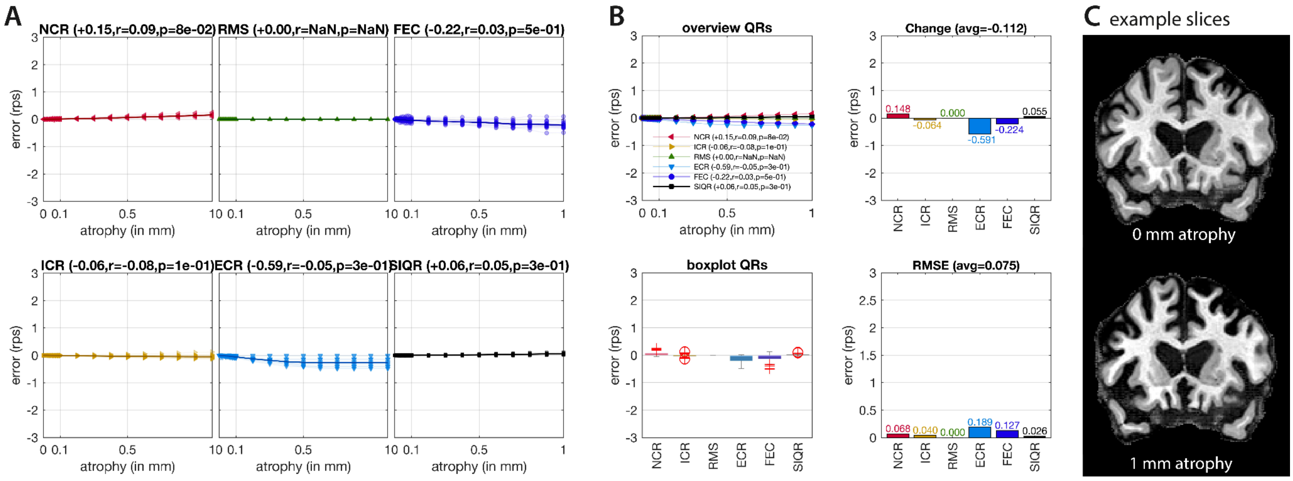

Figure S3: Changes in current quality scores (A) for the phantoms presented in (Rusak et al., 2022, C), which simulated neocortical atrophy of up to 1 mm in 20 subjects from the ADNI database, where 0.01 mm cortical loss represents 1 year of healthy ageing. As only the neocortical thickness is changed (C), similar outcomes in the measures are expected regardless of the simulated atrophy rate. However, the slightly different inhomogeneity and noise pattern in the CSF could introduce further bias in the evaluation.

Table S1: Spearman correlation coefficients with p-values in the upper and lower part of the table, respectively.

p/rho	noise	bias	rRMS	NCR	ICR	RES	ECR	FEC	IQR	SIQR	Kappa	rCSFV	rGMV	rWMV
noise		0.031	-0.008	**0.976**	-0.086	-0.004	0.631	**0.979**	0.897	**0.941**	-0.857	-0.716	-0.705	0.851
bias	6E-01		0.015	-0.088	**0.961**	0.007	0.158	0.009	-0.087	-0.019	-0.034	-0.190	0.104	0.018
rRMS	9E-01	8E-01		-0.012	0.028	0.332	**0.585**	0.004	0.167	0.173	-0.413	-0.003	-0.288	0.220
**NCR**	5E-226	1E-01	8E-01		-0.175	-0.118	0.596	0.977	0.875	0.923	-0.819	-0.667	-0.730	0.851
**ICR**	1E-01	8E-191	6E-01	1E-03		-0.118	0.146	-0.091	-0.213	-0.134	0.046	-0.104	0.085	-0.009
**RES**	9E-01	9E-01	3E-10	3E-02	3E-02		0.001	-0.050	0.223	0.143	-0.124	0.152	0.259	-0.229
**ECR**	6E-39	3E-03	1E-32	5E-34	7E-03	1E+00		0.612	0.548	0.632	-0.845	-0.505	-0.837	0.836
**FEC**	1E-234	9E-01	9E-01	5E-229	9E-02	4E-01	3E-36		0.901	0.950	-0.850	-0.722	-0.697	0.849
**IQR**	2E-121	1E-01	2E-03	4E-108	8E-05	4E-05	6E-28	0.000		0.979	-0.889	-0.717	-0.569	0.735
**SIQR**	5E-160	7E-01	1E-03	8E-142	1E-02	8E-03	3E-39	3E-172	0.000		-0.916	-0.729	-0.647	0.805
**Kappa**	4E-99	5E-01	2E-15	2E-83	4E-01	2E-02	1E-93	1E-95	0.000	1E-135		0.733	0.780	-0.893
**rCSFV**	2E-54	4E-04	1E+00	6E-45	6E-02	5E-03	3E-23	9E-56	0.000	2E-57	2E-58		0.428	-0.700
**rGMV**	3E-52	5E-02	7E-08	1E-57	1E-01	1E-06	3E-90	1E-50	0.000	1E-41	2E-70	2E-16		-0.924
**rWMV**	3E-96	7E-01	4E-05	3E-96	9E-01	2E-05	7E-90	3E-95	0.000	3E-78	5E-119	4E-51	2E-142	

Detailed Real Data Results - IXI

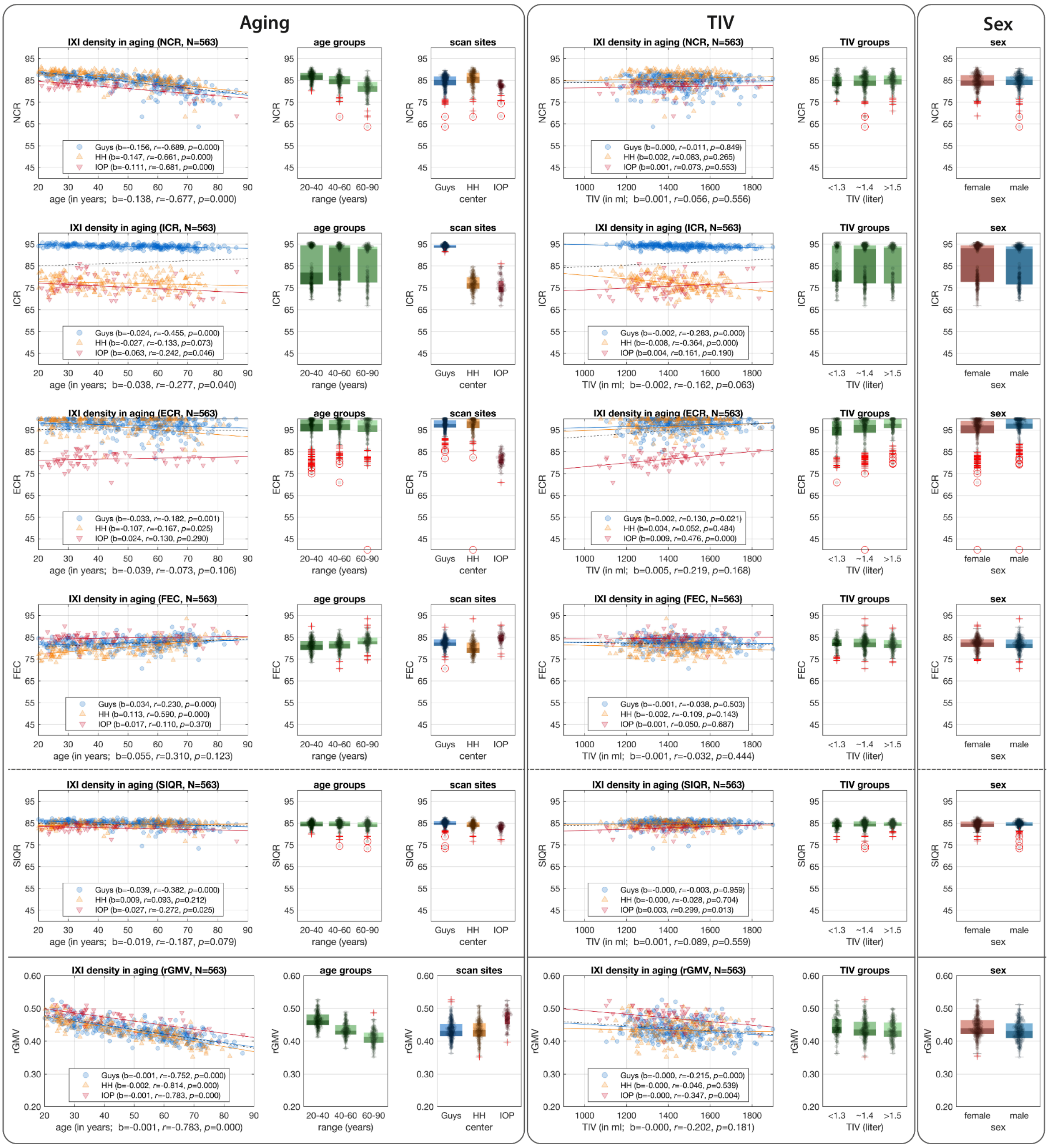

Figure S4: Shown are the changes of all quality ratings (NCR, ICR, ECR, FEC, and SIQR) and the relative GM volume (rGMV) for aging (left), total intracranial volume (TIV, center), and sex (right).

Detailed Real Data Results - MR-ART

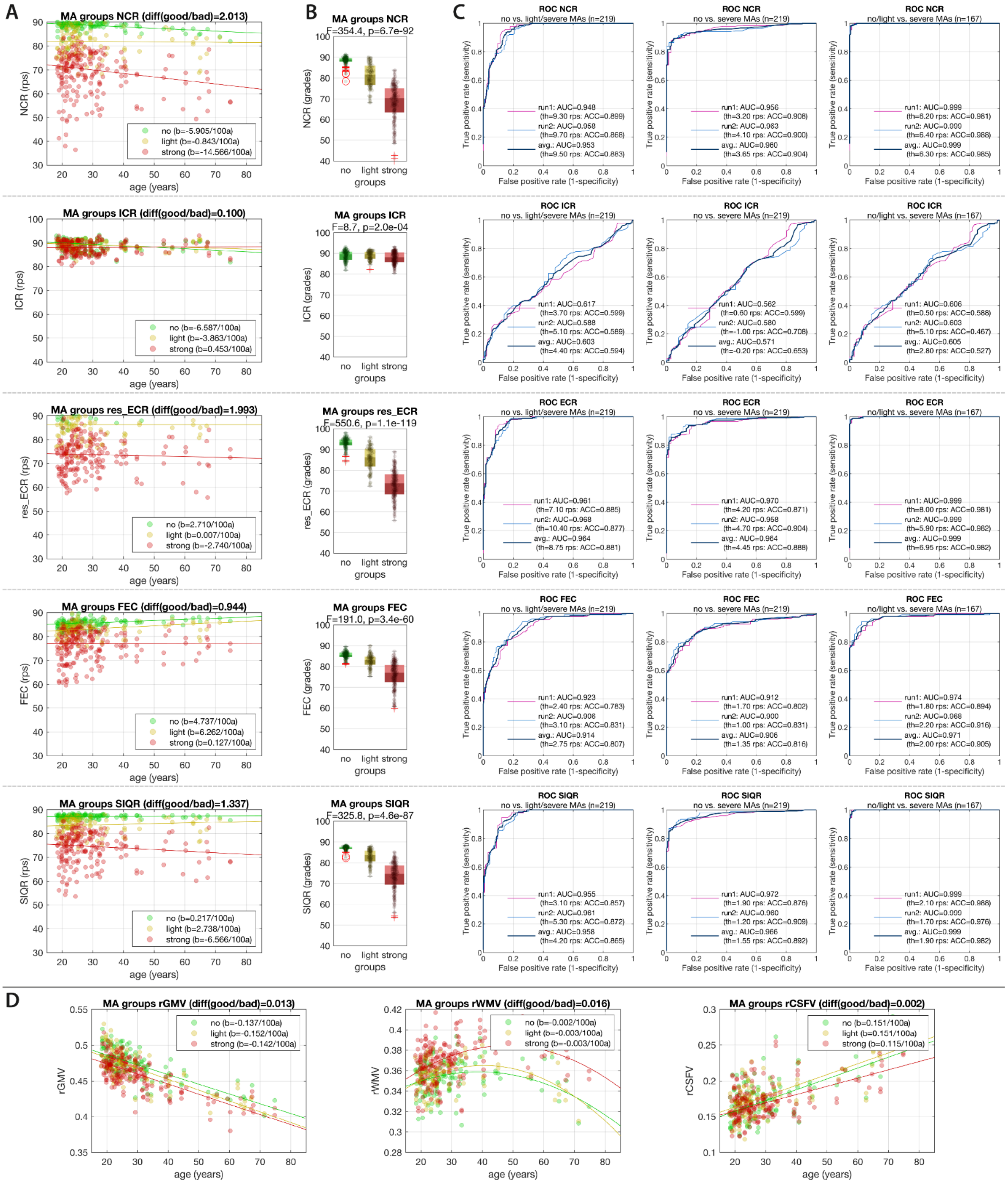

Figure S5: Shown are the changes of all quality ratings (NCR, ICR, ECR, FEC, and SIQR) in aging (A) and grouped by the expert rating (B). (C) shows the ROC analysis of each quality rating to separate the expert rated motion groups. The change of the *relative GM volume* (**rGMV**) for aging is shown in (D).

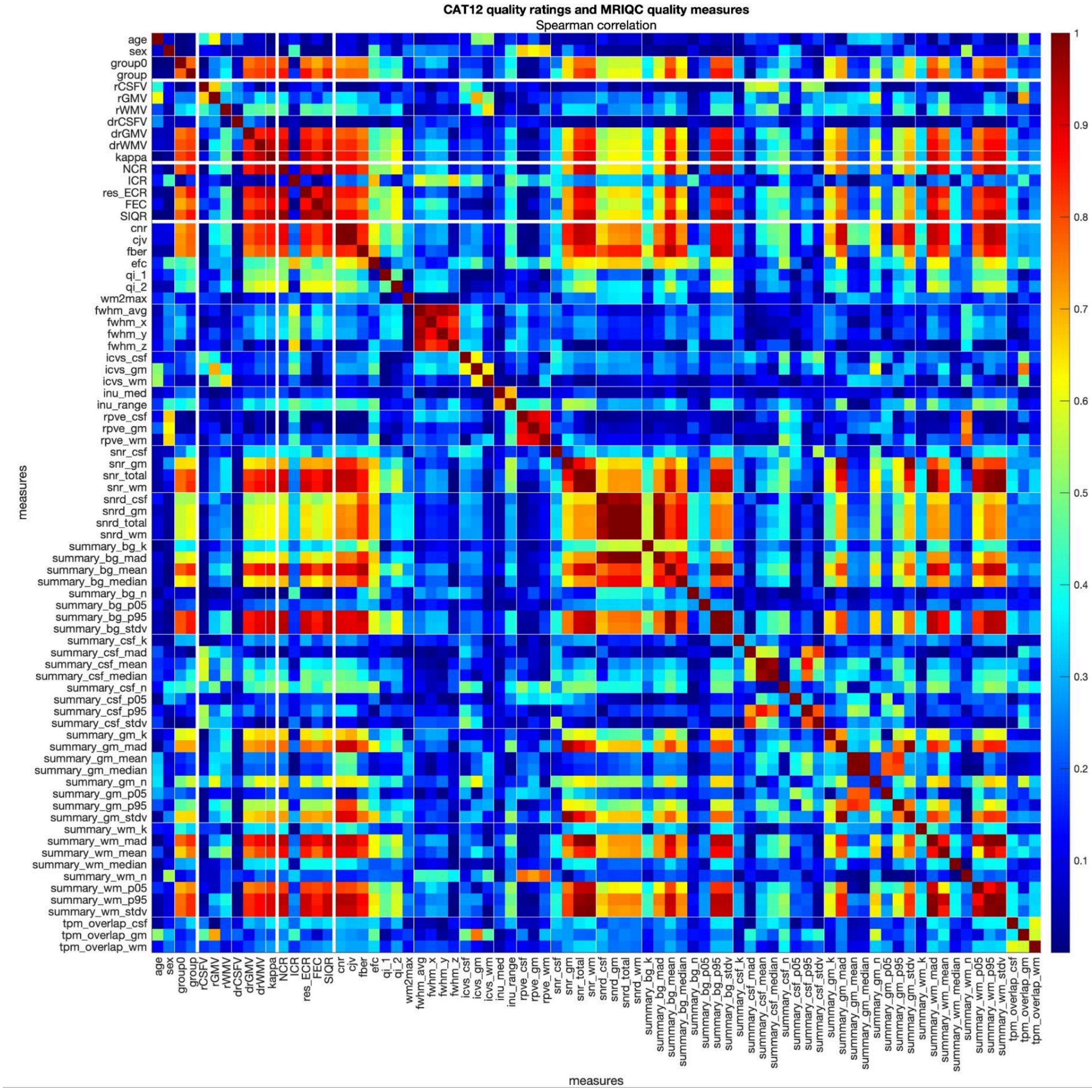

Figure S6: Spearman's correlation coefficient matrix representing the associations between CAT12 and MRIQC quality measures in the MR-ART dataset (Nárai et al., 2022), as well as age, sex, group0 (i.e., scan name/condition, e.g. instructed motion), group (final expert rating), relative CSF/GM/WM volume (rCSFV/rGMV/rWMV), ΔV (dvol_rel_CGW, volume change in relation to motion-free scan), and kappa statistic (estimated to the motion-free scan and averaged over all tissues).

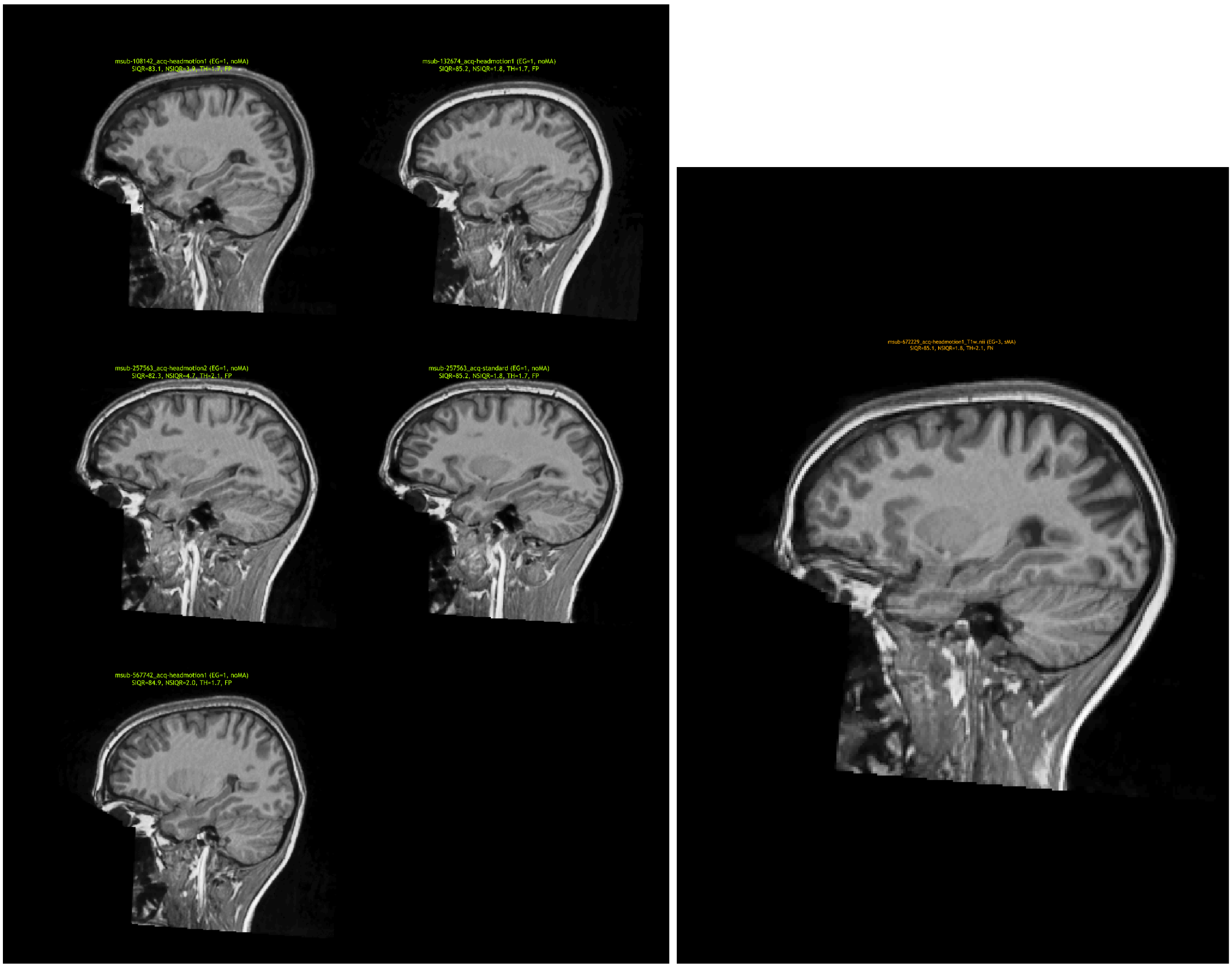

Figure S7: Example slice of the four **false-positive** cases from the MR-ART datasets that failed in the outlier detection between **no vs. severe artifacts.** They were classified by our rating as severe motion cases, whereas experts assessed them as motion-free. There were one false-negative cases in the separation between no vs. severe artifacts.

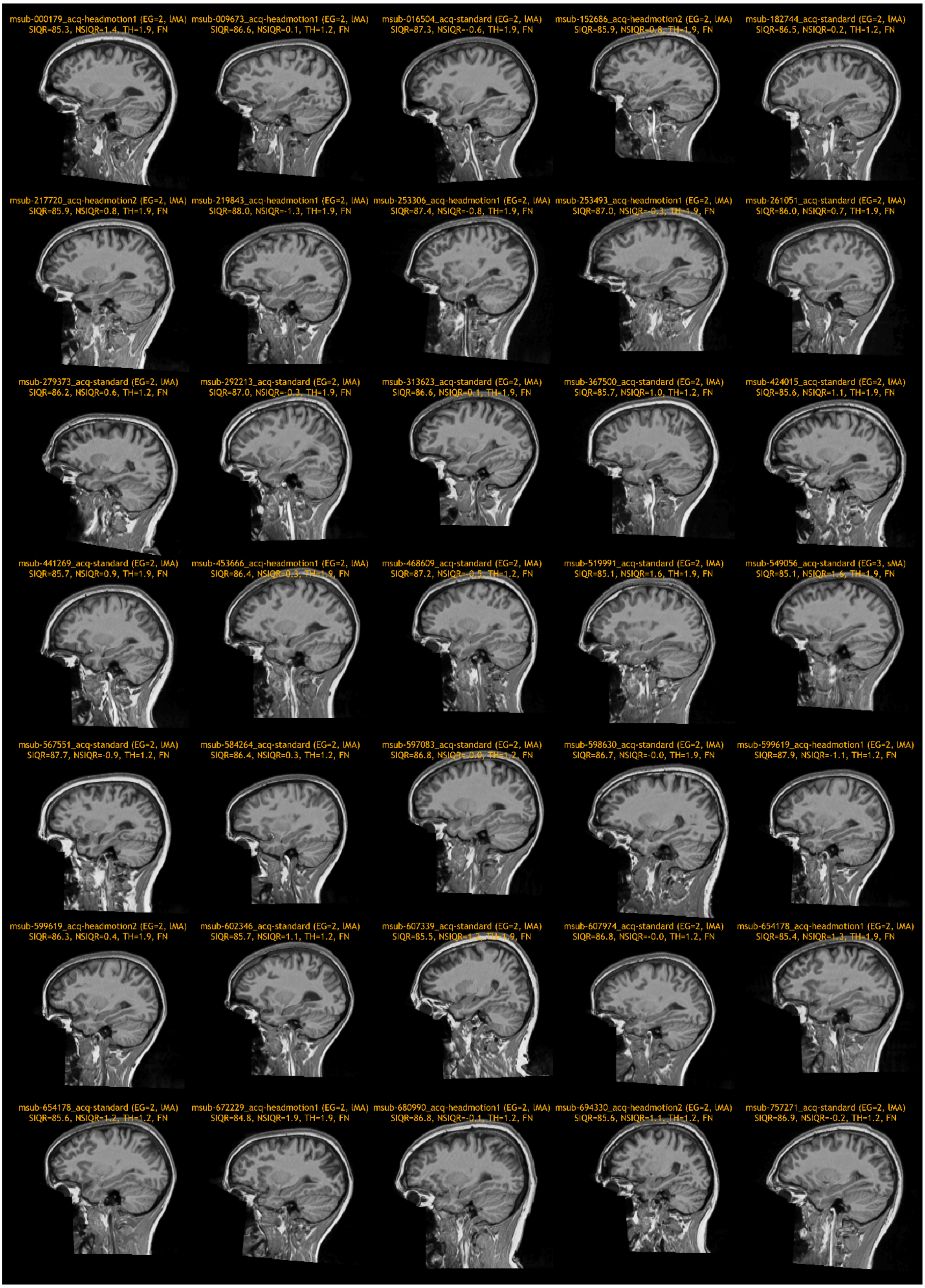

Figure S8a: Example slices of the **false-negative** cases from the MR-ART dataset (35 of 42) that failed in the outlier detection between **no vs. slight/severe artifacts** and were classified by our measure as acceptable, but failed in the expert grouping (EG) with a light (EG=2) or severe (EG=3) motion artifact rating.

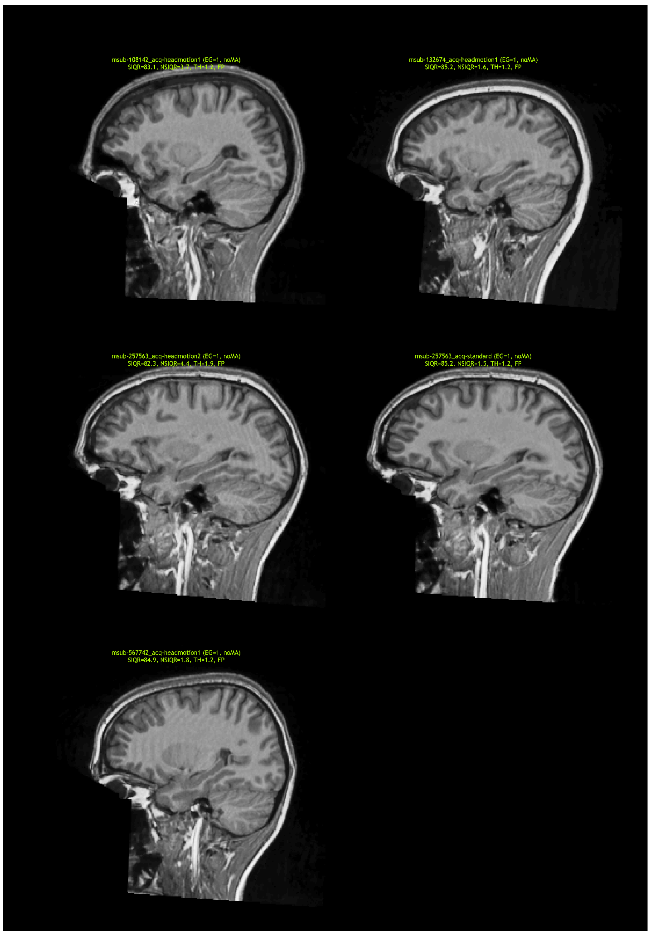

Figure S8b: Example slices with **false-positive** cases from the MR-ART dataset that failed in the outlier detection between **no vs. slight/severe artifacts** and were classified by our measure as unacceptable, but as motion-free by the expert.

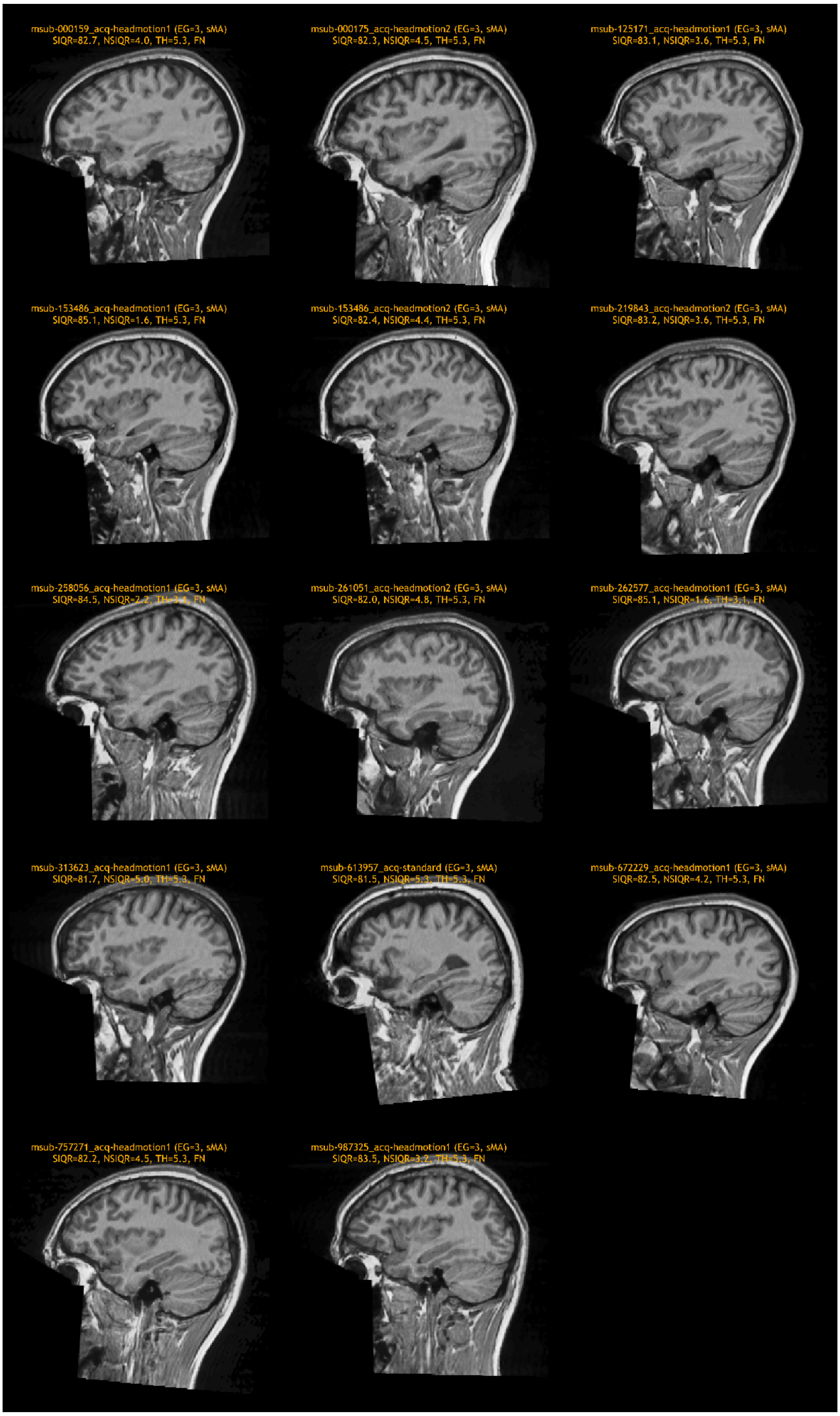

Figure S9a: Example slices with **false-negative cases** from the MR-ART datasets that failed in the outlier detection between **no/light vs. severe artifacts** and were classified by our measure as acceptable (no/light motion) but labeled as severe (EG=3) rating by the experts.

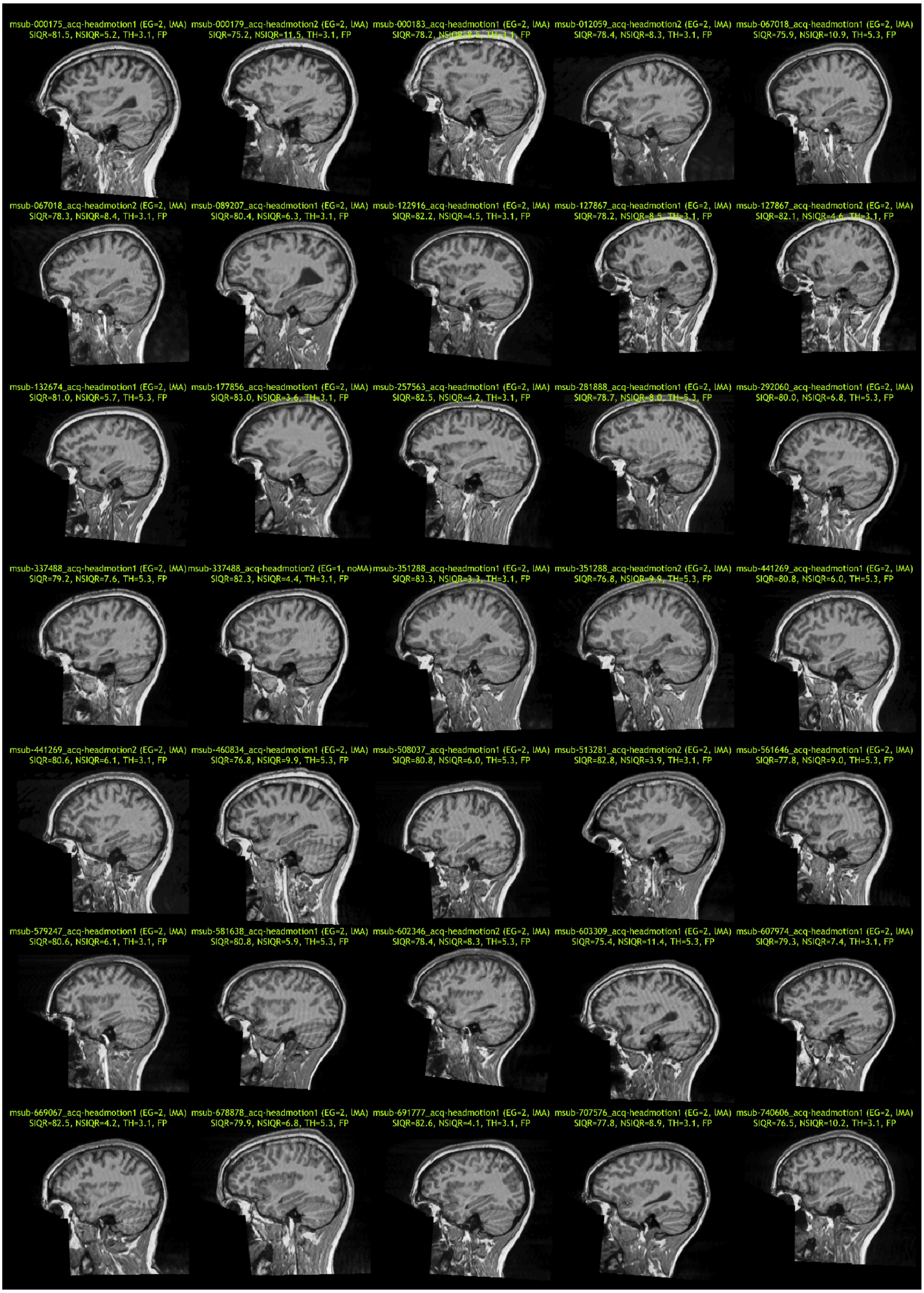

Figure S9b: Example slices with **false-positive** cases from the MR-ART dataset (35 of 47) that failed in the outlier detection between **no/light vs. severe artifacts** and were classified by our measure as unacceptable (severe motion) but as artifact free (EG=1) or with slight motion artifact (EG=2) by the expert.

Table S2: Parameters of the full Tohoku dataset with the selected scans.

mprage	orientation	SENSE	TFE	TI	voxdim0	voxdim1	voxdim2	durationSec
20120701_112011MPRAGE10SENSEs1401a1014.nii	A	2;1;1	1800	845	1	1	1	295
20120701_112011MPRAGE10SENSEs2601a1026.nii	A	2;1;1	1800	845	1	1	1	295
20120701_112011MPRAGE1SENSEs1001a1010.nii	A	1.5;1;1	2100	812	1	1	1	369
20120701_112011MPRAGE1SENSEs1701a1017.nii	A	1.5;1;1	2100	812	1	1	1	369
20120701_112011MPRAGE2SENSEs1101a1011.nii	A	2;1;1	2100	812	1	1	1	276
20120701_112011MPRAGE2SENSEs1801a1018.nii	A	2;1;1	2100	812	1	1	1	276
20120701_112011MPRAGE3SENSEs1901a1019.nii	A	1.5;1;1	1850	711	1	1	1	197
20120701_112011MPRAGE3SENSEs301a1003.nii	A	1.5;1;1	1850	711	1	1	1	197
20120701_112011MPRAGE4SENSEs2001a1020.nii	A	2;1;1	1850	711	1	1	1	147
20120701_112011MPRAGE4SENSEs401a1004.nii	A	2;1;1	1850	711	1	1	1	147
20120701_112011MPRAGE5SENSEs2101a1021.nii	A	1.5;1;1	1500	711	1	1	1	160
20120701_112011MPRAGE5SENSEs501a1005.nii	A	1.5;1;1	1500	711	1	1	1	160
20120701_112011MPRAGE6SENSEs2201a1022.nii	A	2;1;1	1500	711	1	1	1	119
20120701_112011MPRAGE6SENSEs601a1006.nii	A	2;1;1	1500	711	1	1	1	119
20120701_112011MPRAGE7SENSEs2301a1023.nii	A	2;1;1	1500	575	2	2	1	83
20120701_112011MPRAGE7SENSEs701a1007.nii	A	2;1;1	1500	575	2	2	1	83
20120701_112011MPRAGE8SENSEs2401a1024.nii	A	2;1;1	2100	575	2	2	1	116
20120701_112011MPRAGE8SENSEs801a1008.nii	A	2;1;1	2100	575	2	2	1	116
20120701_112011MPRAGE9SENSEs2501a1025.nii	A	2;1;1	2100	711	1	1	1	167
20120701_112011MPRAGE9SENSEs901a1009.nii	A	2;1;1	2100	711	1	1	1	167
20120707_124735MPRAGE14SENSEs3401a1034.nii	A	2;1;1	1800	811	1	1	1	237
20120707_124735MPRAGE14SENSEs501a1005.nii	A	2;1;1	1800	811	1	1	1	237
20120707_124735MPRAGE14SENSEs901a1009.nii	A	2;1;1	1800	811	1	1	1	237
20120707_124735MPRAGE15SENSEs601a1006.nii	A	2.5;1;1	1800	866	1	1	1	190
20120707_124735MPRAGE17SENSEs1001a1010.nii	A	1.5;1;1	1800	710	1	1	1	239
20120707_124735MPRAGE18SENSEs1101a1011.nii	A	1.5;1;1	1800	865	1	1	1	316
20120707_124735MPRAGE18SENSEs3601a1036.nii	A	1.5;1;1	1800	865	1	1	1	316
20120707_124735MPRAGE19SENSEs1201a1012.nii	A	1.5;1;1	2500	726	1	1	1	331
20120707_124735MPRAGE1woSENSEs3501a1035.nii	A	1;1;1	2500	695	1	1	1	481
20120707_124735MPRAGE20SENSEs1301a1013.nii	A	2;1;1	1800	850	1	1	1	237
20120707_124735MPRAGE21SENSEs3801a1038.nii	A	1.5;1;1	1800	798	1	1	1	239
20120707_124735MPRAGE22SENSEs3701a1037.nii	A	1.5;1;1	1800	863	1	1	1	316
20120707_124735MPRAGEdcnTr1800CLEARs3301a1033.nii	A	1;1;1	1800	715	1	1	1	347
20120707_124735veryshortMPRAGESENSEs2901a1029.nii	A	4.5;1;1	784	402	1	1	2	36
20120707_124735veryshortMPRAGESENSEs3901a1039.nii	A	4.5;1;1	784	402	1	1	2	36
20120725_115520veryshortMPRAGESENSEs1001a1010.nii	A	4.5;1;1	784	402	1	1	2	36
20120725_115520veryshortMPRAGESENSEs401a1004.nii	A	4.5;1;1	784	402	1	1	2	36
20120729_114553veryshortMPRAGESENSEs301a1003.nii	A	4.5;1;1	784	402	1	1	2	36
20120729_114553veryshortMPRAGESENSEs801a1008.nii	A	4.5;1;1	784	402	1	1	2	36
20120802_113755MPRAGE22SENSEs401a1004.nii	A	2;1;1	1700	863	0	0	1	353
20120802_113755MPRAGE23SENSEs901a1009.nii	A	2;1;1	1700	842	1	1	1	581
20120802_113755MPRAGE24SENSEs1201a1012.nii	A	1;1;1	1600	808	1	1	1	611
20120802_113755MPRAGE25SENSEs1501a1015.nii	A	3;1;1	1505	764	1	1	1	299
20120808_113817MPRAGE26SENSEs1101a1011.nii	A	2.5;1;1	1750	867	1	1	1	491
20120808_113817shorterMPRAGESENSEs901a1009.nii	A	4;1;1	1113	572	1	1	2	53
20120808_113817veryshortMPRAGESENSEs301a1003.nii	A	4.5;1;1	784	402	1	1	2	36
20120811_153828MPRAGE26SENSEs501a1005.nii	A	2.5;1;1	1750	867	1	1	1	491
20120811_153828MPRAGEdcnTr1800CLEARs601a1006.nii	A	1;1;1	1800	715	1	1	1	347
20120822_115825MPRAGE18SENSEs501a1005.nii	A	1.5;1;1	1800	865	1	1	1	316
20120822_115825MPRAGE27SENSEs1001a1010.nii	S	2;1;1.5	1800	883	1	1	1	237
20120822_115825MPRAGE28SENSEs801a1008.nii	S	1.5;1;1.5	2200	1077	1	1	1	422
20120822_115825MPRAGE29SENSEs1101a1011.nii	S	2;1;1.5	1800	887	1	1	1	190
20120902_164438MPRAGE14ti1kSENSEs601a1006.nii	A	2;1;1	1800	1000	1	1	1	237
20120902_164438MPRAGE18ti960SENSEs701a1007.nii	A	1.5;1;1	1800	950	1	1	1	316
20120914_182400MPRAGEasVISTA28SENSEs501a1005.nii	S	2;1;2	2200	612	1	1	1	282
20120920_182515MPRAGEasVISTA28TI1600SENSEs401a1004.nii	S	2;1;2	2200	1600	1	1	1	283
20120924_174146MPRAGEasVISTA28TFE1600TI1200SEs1001a1010.nii	S	2;1;2	1800	1200	1	1	1	232
20120926_181803shorterMPRAGE1200TISENSEs801a1008.nii	A	4;1;1			1	1	2	53
20120930_112053MPRAGEasV10SENSEs2901a1029.nii	S	2;1;2	2200	900	1	1	1	283
20120930_112053MPRAGEasV11SENSEs3201a1032.nii	S	2;1;2	1700	900	1	1	1	219
20120930_112053MPRAGEasV12SENSEs3401a1034.nii	S	2;1;2	1400	800	1	1	1	180
20120930_112053MPRAGEasV13SENSEs2801a1028.nii	S	2;1;2	1300	750	1	1	1	167
20120930_112053MPRAGEasV1SENSEs1901a1019.nii	S	2;1;2	1900	1300	1	1	1	245
20120930_112053MPRAGEasV1SENSEs301a1003.nii	S	2;1;2	1900	1300	1	1	1	245
20120930_112053MPRAGEasV2SENSEs401a1004.nii	S	2;1;2	2200	1300	1	1	1	283
20120930_112053MPRAGEasV3SENSEs501a1005.nii	S	2;1;2	2600	1300	1	1	1	334

20120930_112053MPRAGEasV4SENSEs601a1006.nii	S	2;1;2	2200	1200	1	1	1	283
20120930_112053MPRAGEasV5SENSEs701a1007.nii	S	2;1;2	2200	1400	1	1	1	283
20120930_112053MPRAGEasV6SENSEs801a1008.nii	S	2;1;2	2200	1600	1	1	1	283
20120930_112053MPRAGEasV7SENSEs2401a1024.nii	S	2;1;2	1900	1100	1	1	1	244
20120930_112053MPRAGEasV7SENSEs3001a1030.nii	S	2;1;2	1900	1100	1	1	1	244
20120930_112053MPRAGEasV8SENSEs2501a1025.nii	S	2;1;2	1900	900	1	1	1	244
20120930_112053MPRAGEasV8SENSEs3301a1033.nii	S	2;1;2	1900	900	1	1	1	244
20120930_112053MPRAGEasV9SENSEs3101a1031.nii	S	2;1;2	1900	700	1	1	1	244
20121006_143435MPGEsagml1SENSEs301a1003.nii	S	2;1;2	1800	900	1	1	1	208
20121006_143435MPGEsagml2SENSEs401a1004.nii	S	2;1;2	1400	900	1	1	1	162
20121006_143435MPGEsagml3SENSEs501a1005.nii	S	2;1;2	2100	900	1	1	1	242
20121006_143435MPRAGE30CLEARs901a1009.nii	A	1;1;1	1800	900	1	1	1	347
20121006_143435MPRAGE31SENSEs1001a1010.nii	A	2;1;1	1800	900	1	1	1	179
20121006_143435MPRAGE32SENSEs1101a1011.nii	A	1.5;1;1	1600	900	1	1	1	212
20121014_121738MPGEsagml1SENSEs1201a1012.nii	S	2;1;2	1800	900	1	1	1	208
20121014_121738MPGEsagml1SENSEs301a1003.nii	A	2;1;2	1800	900	1	1	1	208
20121014_121738MPGEsagml2SENSEs1301a1013.nii	S	2;1;2	1400	900	1	1	1	162
20121014_121738MPGEsagml2SENSEs401a1004.nii	A	2;1;2	1400	900	1	1	1	162
20121014_121738MPGEsagml3SENSEs1401a1014.nii	S	2;1;2	2100	900	1	1	1	242
20121014_121738MPGEsagml3SENSEs501a1005.nii	A	2;1;2	2100	900	1	1	1	242
20121014_121738MPGEsagml4SENSEs1501a1015.nii	S	2;1;2	1800	1200	1	1	1	208
20121014_121738MPGEsagml4SENSEs601a1006.nii	A	2;1;2	1800	1200	1	1	1	208
20121020_132933MPGEsagml1SENSEs601a1006.nii	S	2;1;2	1800	900	1	1	1	208
20121020_132933MPGEsagml4SENSEs501a1005.nii	S	2;1;2	1800	1200	1	1	1	208
20121020_132933MPGEsagml5SENSEs901a1009.nii	A	2;1;2	1800	1300	1	1	1	208
20121020_132933MPRAGE1woSENSEs301a1003.nii	A	1;1;1	2500	713	1	1	1	481
20121020_132933MPRAGEasV11SENSEs701a1007.nii	S	2;1;2	1700	900	1	1	1	219
20121020_132933MPRAGEasV14SENSEs801a1008.nii	S	1;1;2	1700	900	1	1	1	428
20121028_122334MPGEsagml3SENSEs701a1007.nii	S	2;1;2	2100	900	1	1	1	242
20121028_122334MPGEsagml4SENSEs501a1005.nii	S	2;1;2	1800	1200	1	1	1	208
20121028_122334MPGEsagml6SENSEs301a1003.nii	S	2;1;2	2500	750	1	1	1	288
20121028_122334MPRAGE1woSENSEs601a1006.nii	A	1;1;1	2500	750	1	1	1	481
20121029_151839MPGEsagml1SENSEs501a1005.nii	S	2;1;2	1800	900	1	1	1	208
20121029_151839MPGEsagml2SENSEs301a1003.nii	S	2;1;2	1400	900	1	1	1	162
20121029_151839MPGEsagml3SENSEs701a1007.nii	S	2;1;2	2100	900	1	1	1	242
20121029_151839MPGEsagml7SENSEs601a1006.nii	S	1.5;1;1.5	1800	800	1	1	1	278
20121029_151839MPGEsagml8SENSEs401a1004.nii	S	2;1;2	1400	750	1	1	1	162
20121029_151839MPRAGE14SENSEs901a1009.nii	A	2;1;1	1800	800	1	1	1	237
20121029_151839MPRAGE33SENSEs801a1008.nii	A	1;1;1	1800	800	1	1	1	462
20121118_091051MPRAGE1woSENSEs301a1003.nii	A	1;1;1	2500	695	1	1	1	481
20121118_091051MPRAGEdcnSENSESENSEs401a1004.nii	A	2;1;1	2500	695	1	1	1	248
20121118_091051MPRAGE1woSENSEGRregularCLEs501a1005.nii	A	1;1;1	2500	1140	1	1	1	482
20121118_091051MPRAGEdcnSENSEGRregularCLEARs601a1006.nii	A	1;1;1	2500	1140	1	1	1	482
20121118_091051MPGEsagml1SENSEs701a1007.nii	S	2;1;2	1800	900	1	1	1	208
20121118_091051MPGEsagml8SENSEs801a1008.nii	S	2;1;2	1400	750	1	1	1	162
20121118_091051MPRAGEasV12SENSEs901a1009.nii	S	2;1;2	1400	800	1	1	1	180
20130211_091630MPGEsagml084SENSEs1101a1011.nii	S	2;1;2	1800	800	1	1	1	237
20130211_091630MPGEsagml099SENSEs1301a1013.nii	S	2;1;2	1800	800	1	1	1	208
20130211_091630MPGEsagml09SENSEs1201a1012.nii	S	2;1;2	1800	800	1	1	1	222
20130211_091630MPGEsagml1NSA2SENSEs601a1006.nii	S	2;1;2	1800	900	1	1	1	415
20130211_091630MPGEsagml1SENSEs501a1005.nii	S	2;1;2	1800	700	1	1	1	208
20130211_091630MPGEsagml1TI700SENSEs801a1008.nii	S	2;1;2	1800	700	1	1	1	208
20130211_091630MPGEsagml35SENSEs301a1003.nii	S	1.5;1;2	2100	900	1	1	1	324
20130211_091630MPGEsameConDcnSENSEs401a1004.nii	S	1.5;1;1.25	2500	706	1	1	1	331
20130211_091630MPRAGE1woSENSEs701a1007.nii	S	1;1;1	2500	706	1	1	1	481
20130213_192623MPGEsagml9SENSEs301a1003.nii	S	1.25;1;1.25	1800	880	1	1	1	379
20130219_183335MPGEsagml842SENSEs401a1004.nii	S	2;1;2	2100	750	1	1	1	276
20130219_183335MPRAGE2SENSEs501a1005.nii	A	2;1;2	2100	800	1	1	1	276

Click here to access/download;Figure;fig1.pdf

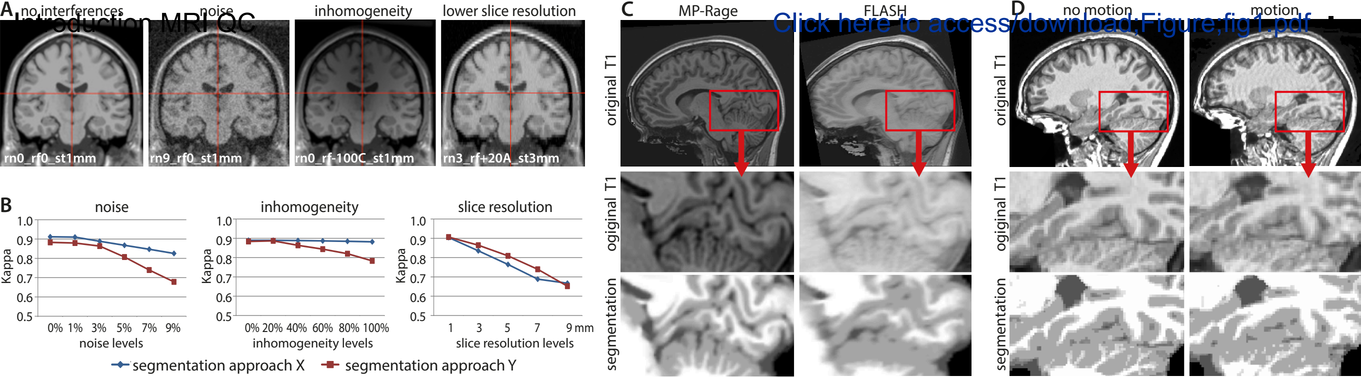

QC evaluation espects
Click here to access/download;Figure

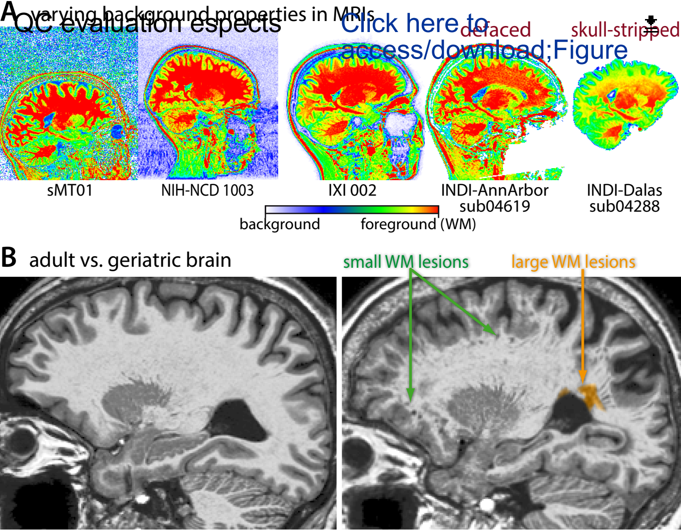

QC rating table

Click here to access/download;Figure

Quality definition	excellent	good	satisfactory	sufficient	critical	unacceptable / failed
BWP noise (%)	1	3	5	7	9	15
BWP bias (%)	20	60	100	140	180	300
resolution RES (mm)	0.5	1.0	1.5	2.0	2.5	4.0

Quality ratings

**rating points (rps)**	**100**	**95**	**90**	**85**	**80**	**75**	**70**	**65**	**60**	**55**	**50**	**25**	**0**
linear rating scale	0.5	1	1.5	2	2.5	3	3.5	4	4.5	5	5.5	8	10.5

nominal letters: A+, **A**, A-, B+, **B**, B-, C+, **C**, C-, D+, **D**, D-, E+, **E**, E-, **F**

Click here to access/download;Figure;fig4.pdf

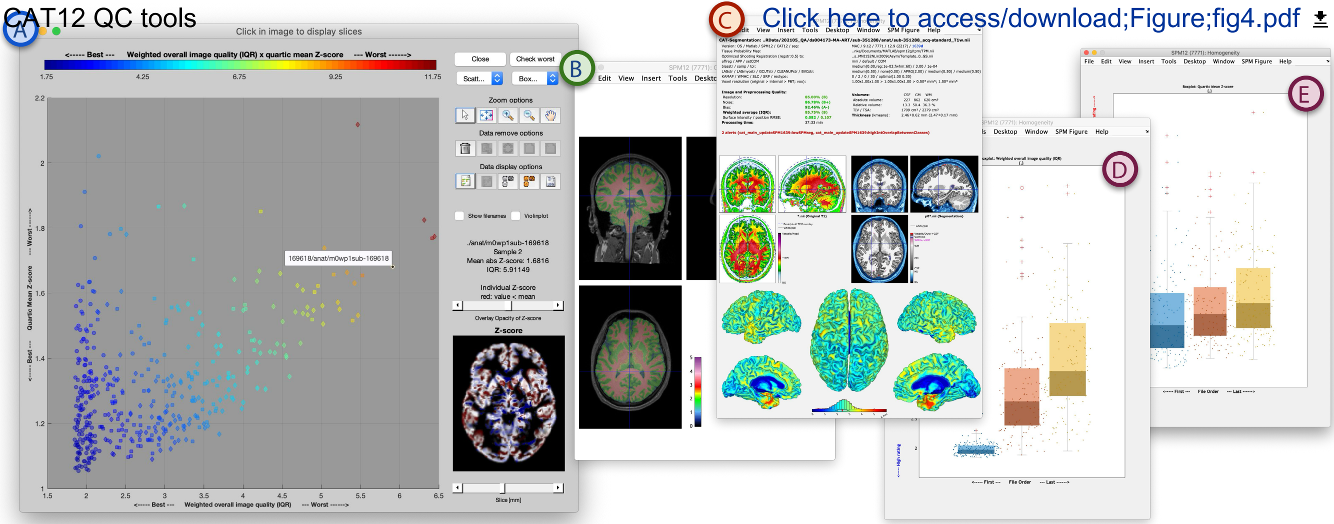

Click here to access/download;Figure;fig5.pdf

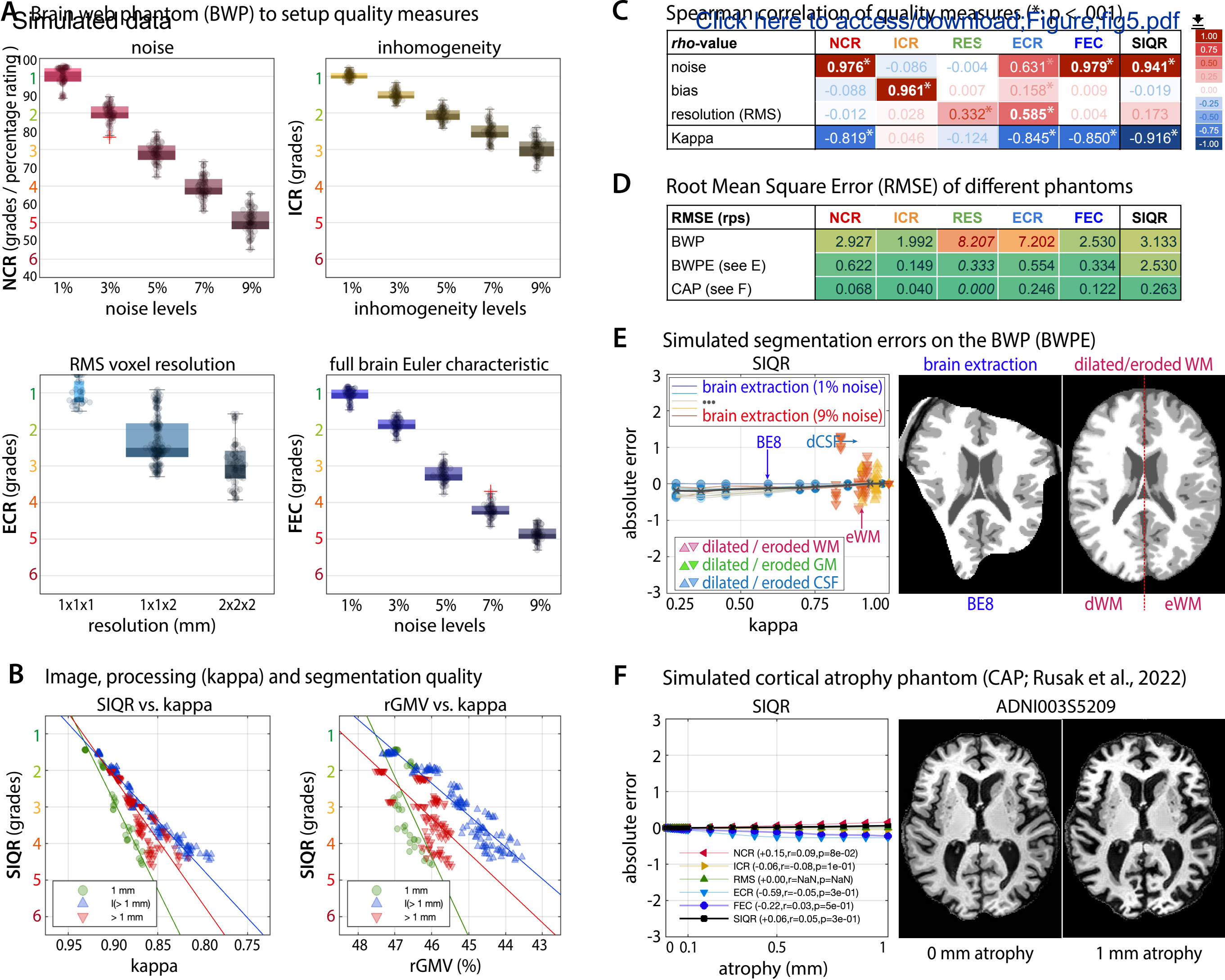

C Spearman correlation of quality measures (*: p < .001)

rho-value	NCR	ICR	RES	ECR	FEC	SIQR
noise	0.976*	-0.086	-0.004	0.631*	0.979*	0.941*
bias	-0.088	0.961*	0.007	0.158*	0.009	-0.019
resolution (RMS)	-0.012	0.028	0.332*	0.585*	0.004	0.173
Kappa	-0.819*	0.046	-0.124	-0.845*	-0.850*	-0.916*

D Root Mean Square Error (RMSE) of different phantoms

RMSE (rps)	NCR	ICR	RES	ECR	FEC	SIQR
BWP	2.927	1.992	*8.207*	7.202	2.530	3.133
BWPE (see E)	0.622	0.149	*0.333*	0.554	0.334	2.530
CAP (see F)	0.068	0.040	*0.000*	0.246	0.122	0.263

Click here to access/download;Figure;fig6.pdf

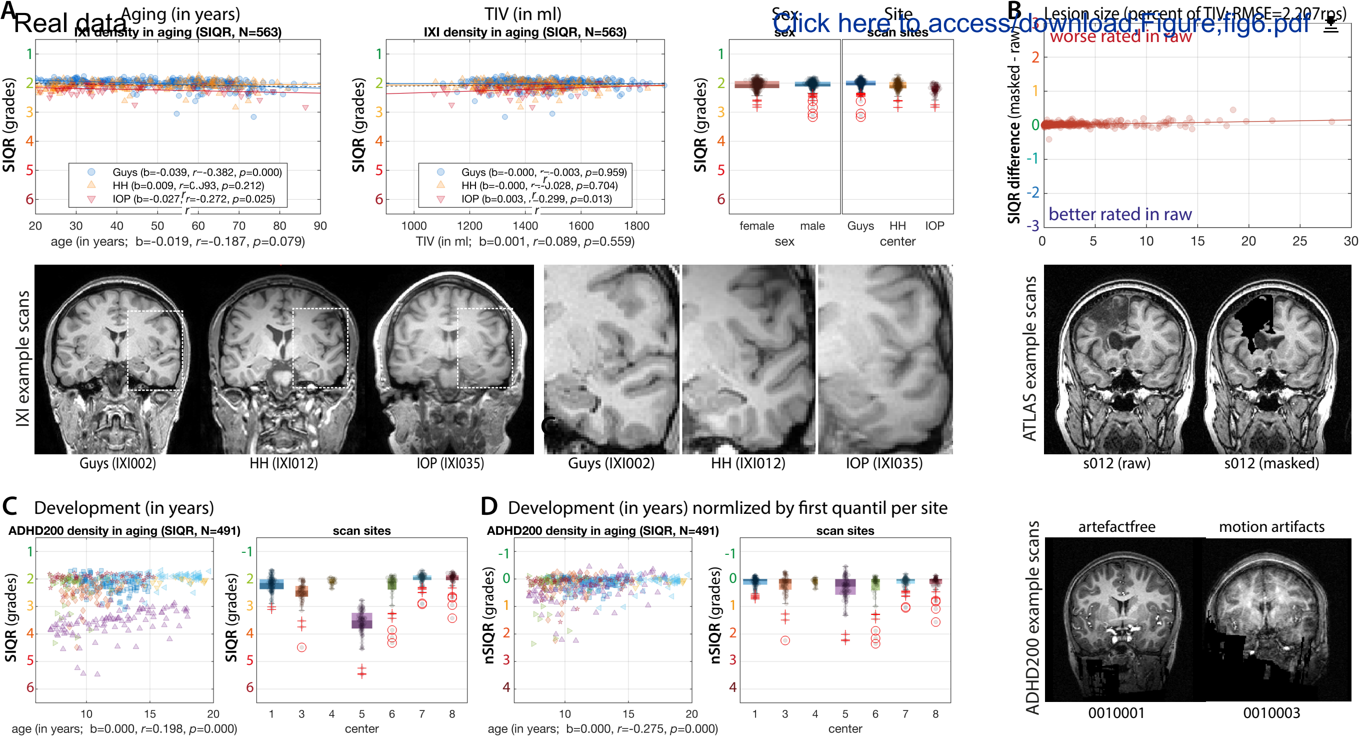

Click here to access/download;Figure;fig7.pdf

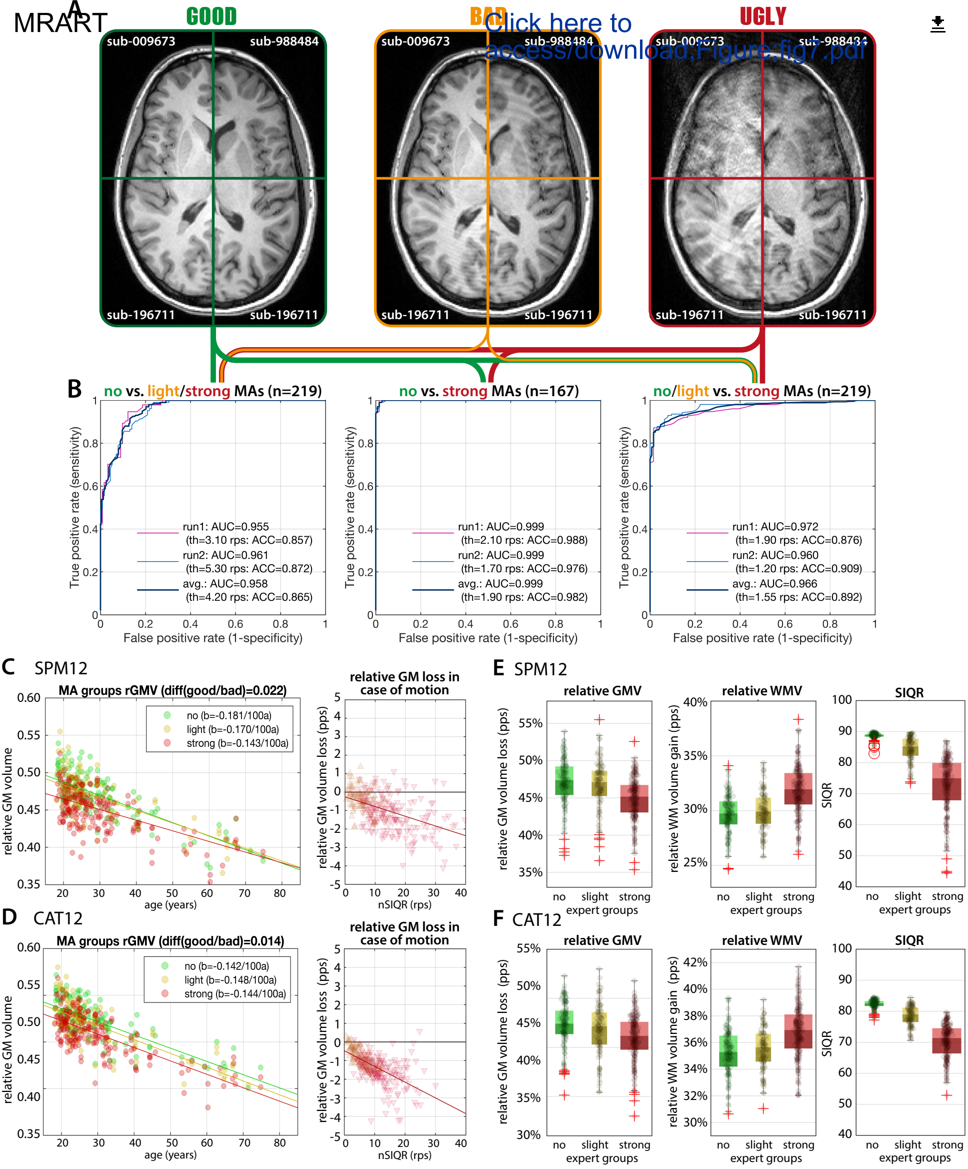

 Click here to access/download;Figure;fig8.jpg

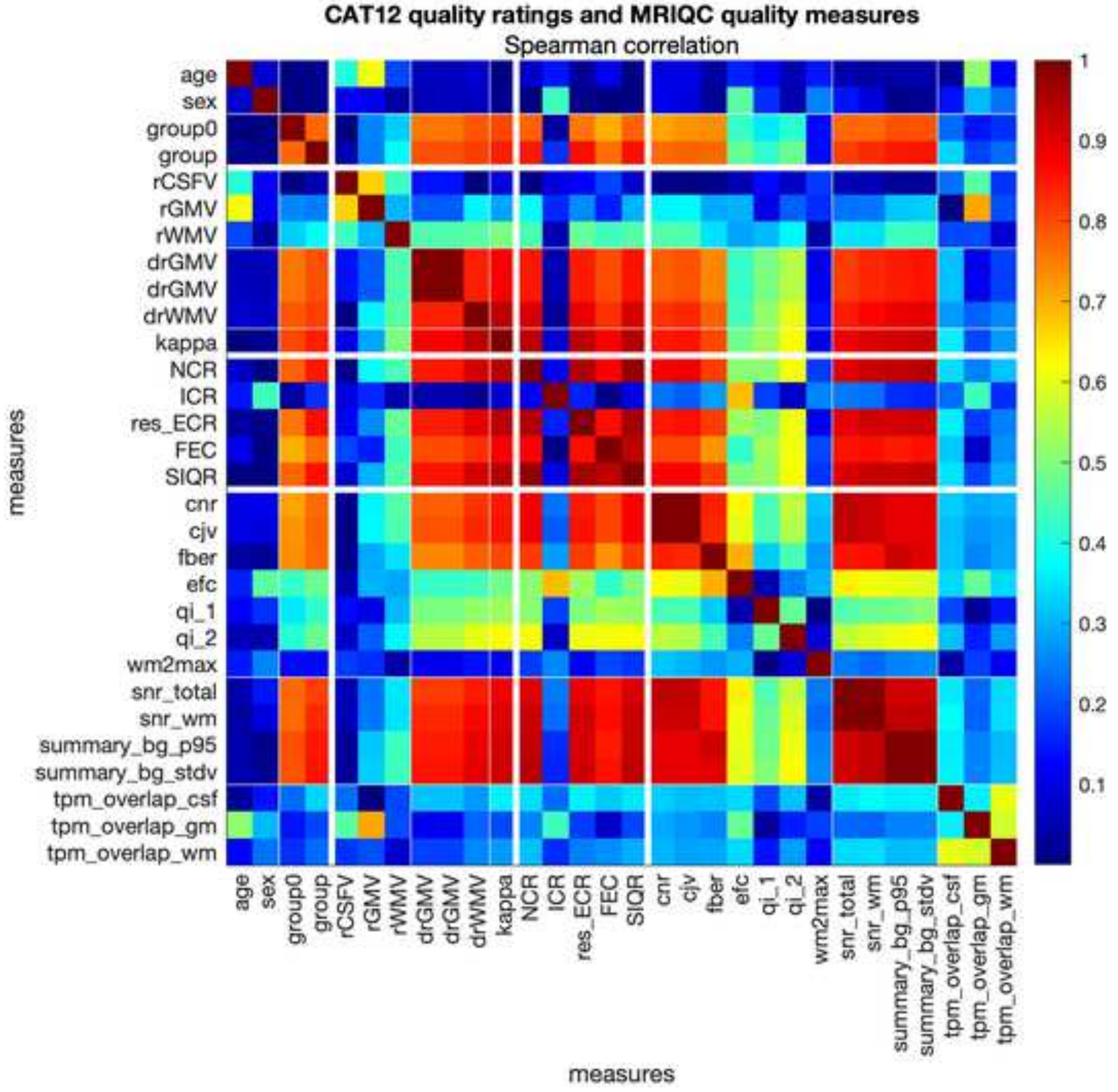

Click here to access/download;Figure;fig9.pdf

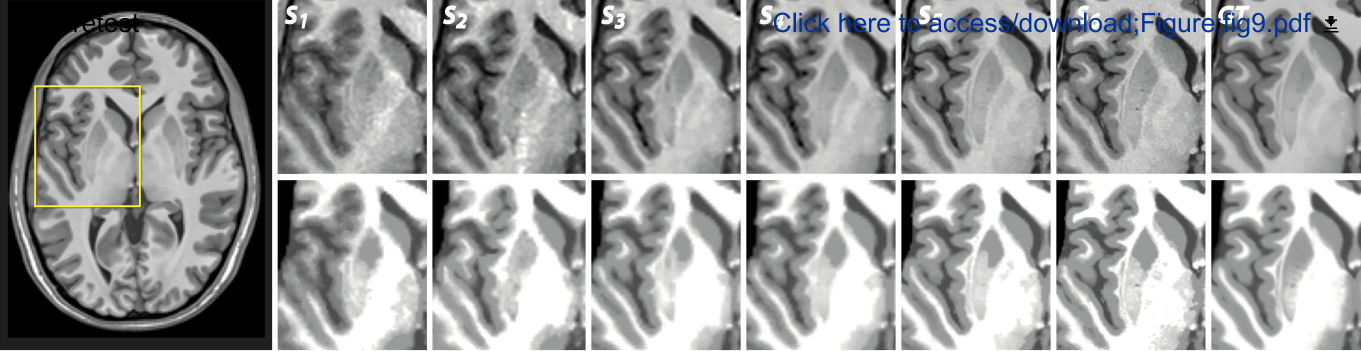

Image	S_1	S_2	S_3	S_4	S_5	S_6	GT
Duration (s)	36	53	119	197	369	611	-
Rx/Ry (mm):	1	1	1.25	1.25	0.75	0.50	0.50
Rz (mm):	2	2	1	1	1	1	0.50
Kappa (avg. (CSF,GM,WM)):	0.776	0.807	0.872	0.876	0.874	0.859	-
rGMV:	45.2 %	45.7%	45.8%	46.2%	46.4 %	45.3 %	45.2 %
SIQR:	69.27 (D^+)	74.65(C)	84.85(B)	85.29 (B)	85.52 (B^+)	82.56 (B^-)	83.15 (B^-)

Click here to access/download;Figure;fig10.png

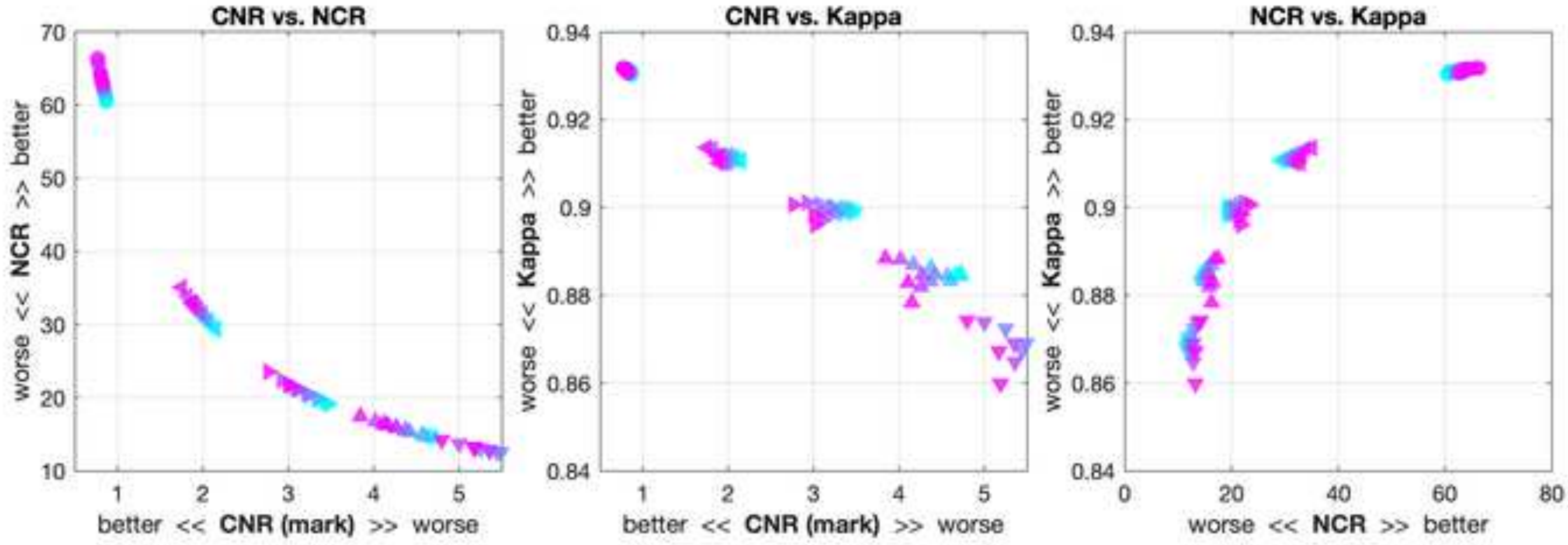

Click here to access/download;Figure

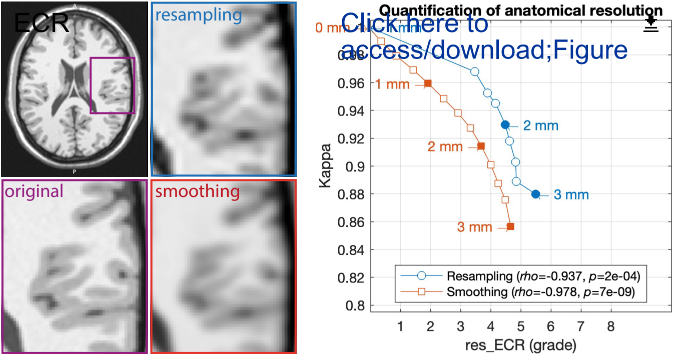

SIQR power

Click here to access/download
Supplementary Material
figS1.pdf

BWP details

Click here to access/download
Supplementary Material
figS2.pdf

Rusak details

Click here to access/download
Supplementary Material
figS3.pdf

IXI further QRs

Click here to access/download
Supplementary Material
figS4.png

Click here to access/download
Supplementary Material
figS5.pdf

MRIQC full correlation table

Click here to access/download
Supplementary Material
figS6.jpg

Full table from fig5

Click here to access/download
Supplementary Material
tabS1.pdf

 Click here to access/download;Personal Cover;QC Cover letter R1.pdf

Scott Edmunds,
Editor-in-Chief
GigaScience Press, BGI Hong Kong

Jena, 8 September 2025

Dear Dr. Edmunds,

We present you with a revised version of our original manuscript 'The Good, the Bad, and the Ugly: Segmentation-Based Quality Control of Structural Magnetic Resonance Images' this time entitled '*Segmentation-Based Quality Control of Structural MRI using the CAT12 Toolbox*'. The revised manuscript has not been published elsewhere and is not under consideration by any other publisher.

With the increasing number of openly available MRI datasets, the possibility of manually checking the quality of each scan is limited. Therefore, scientists have come up with tools to automatically control the quality of the images. In this article, we propose a segmentation-based quality control framework for the structural magnetic resonance images (MRI) as a part of the open-source CAT12 Toolbox (Gaser et al., 2024). Following the reviewers' suggestions, we have tried to improve the clarity of the manuscript and its figures, extended the analyses to include data on children, and provided additional evidence of our tool's effectiveness. It is our hope that the revised article will be regarded as an improvement on the initial version and that it will be reconsidered for publication.

We would greatly appreciate the consideration of our manuscript by your journal.

Sincerely,

Dr. Robert Dahnke
Dr. Polona Kalc
Dr. Gabriel Ziegler
PD Dr. Julian Grosskreuz
Prof. Dr. Christian Gaser

Scott Edmunds,
Editor-in-Chief
GigaScience Press, BGI Hong Kong

Jena, 28 February 2025

Dear Dr. Edmunds,

We would like to submit our original manuscript "*The Good, the Bad, and the Ugly: Segmentation-Based Quality Control of Structural Magnetic Resonance Images*" for consideration by the GigaScience journal. The manuscript has been uploaded on the BiorXiv server, however, it has not been published and it is not under consideration elsewhere. Moreover, there are no conflicts of interest to be disclosed. All the signed authors approved the final version of the manuscript before the submission. Please address all correspondence regarding this manuscript to Dr. Robert Dahnke (robert.dahnke@uni-jena.de).

Our manuscript represents a technical note on the quality control framework for the structural magnetic resonance images (MRI). Neuroimaging research is a fast-growing field, attracting various profiles of scientists and clinical practitioners. Quality assurance is an important step in every research and clinical setting. However, with the increasing number of openly available MRI datasets, the possibility of manually checking the quality of each scan is limited. Therefore, scientists have come up with tools to automatically control the quality of the images. Here we present a segmentation-based quality control framework, which is a part of the open-source CAT12 Toolbox (Gaser et al., 2024), however, it can also be run independently as a Statistical Parametric Mapping (SPM) software batch with input from other MRI-segmentation software. The technical note on the CAT12 Toolbox, published last year in GigaScience, has been a highly cited article as the toolbox has a large number of users worldwide. Therefore, we believe that this manuscript, which introduces, validates and provides further instructions on (the use of) our quality control pipeline, would be highly relevant to the GigaScience readership as well.

We would greatly appreciate the consideration of our manuscript by your journal.

Sincerely,

Dr. Robert Dahnke
Prof. Dr. Christian Gaser
Dr. Gabriel Ziegler
PD Dr. Julian Grosskreuz
Dr. Polona Kalc

Reviewer response:

Reviewer #1:

The article presents a valuable effort towards standardising quality control methods and their evaluation. However, too many choices seem arbitrary without sufficient justification, and too many sections are unclear. Overall, the quality of the work cannot be fully assessed in the current state of the manuscript, and major revisions are needed to correct that. There is also not enough comparison (one) with other methods and no way of evaluating whether these measures are relevant to actual downstream imaging uses. Additionally, the article's goal is highly unclear and led me to think the segmentation measures were part of the QC pipeline until I read the discussion ... Nothing until the discussion explains that the segmentation measures are used to evaluate the single SIQR score output of the QC pipeline.

Response: Thank you very much for your feedback. Although the use of the segmentation in the conceptualisation of the QC tool is stated in the manuscript's title, we acknowledge that the playful reference to the movie title probably obscured the scope of this work. We have therefore decided to change the title of the revised manuscript.

We also agree that various arbitrary choices were made during the development of the tool, and we would like to point out that these choices were motivated by practical experience with data, which is why there are limited references for many of them. Nevertheless, we have tried to address most of the points raised in your review and have rewritten various parts of the manuscript accordingly.

Comments:

"All measures and tools are part of the Computational Anatomy Toolbox (CAT; https://neuro-jena.github.io//cat, Gaser et al., 2024) of the Statistical Parametric Mapping (SPM; http://www.fil.ion.ucl.ac.uk/spm, Ashburner et al. 2002) software and also available as a standalone version (https://neuro-jena.github.io/enigma-cat12/#standalone)." I cannot really expect everyone to avoid Matlab tools. Still, Matlab is a drag to the development of scalable tools nowadays (every system admin's nightmare is to have to try to make Matlab tools run on high-performance computing servers).

Response: We agree with you that the mentioned dependency on Matlab is not in line with open science principles. We are therefore currently developing tools that do not rely on Matlab. Nevertheless, we would like to point out that both SPM and CAT12 (and the QC tool) can be run as standalone versions (also on a high-performing clusters), and that the concept described here (simple

use case specified grading, specific evaluation aspects) can be transferred to other tools, programming languages or platforms. However, we feel that the complete change of the programming language and platform would be beyond the scope of this manuscript.

"such as noise, inhomogeneities, and resolution (Figure 1B)." At this point in the article, it's a bit unclear how that works in Figure 1B.

Response: We have tried to improve the clarity in the revised part. Figure 1B represents how two segmentation algorithms are affected by varying levels of noise, inhomogeneities and resolution from the BWP dataset (revised part in italic):

"(B) The segmentation accuracy can be quantified by the kappa similarity statistic (Cohen, 1960), here presented for two segmentation approaches on simulated images (AubertBroche et al., 2006), *where larger levels of noise, inhomogeneity, or lower resolutions result in a worse overlap with the full-resolution image without interference.*"

"It is assessed within optimized cerebrospinal fluid (CSF) and white matter (WM) regions." Then, the NCR relies on the segmentation, right? What if the segmentation fails?

Response: Correct. As you pointed out, the NCR and all other measures rely on segmentation. All image assessments rely to some extent on some kind of segmentation, which defines regions to quantify the measure and scaling. This can fail or bias the (quality) analysis. However, tissue segmentations are broadly available, accurate, and robust, and severe segmentation failures in CAT12 can be identified using covariance analysis of the segmentation as mentioned in the Section "Software" (Figure 4) and Discussion (p. 21):

"Multivariate outlier detection schemes that are typically applied based on the processed data of a sample in the normalized feature space, using similarity analysis of normalized GM data (e.g. the Gram matrix or kernels) in CAT12 (see Software section), can be used to detect outliers with preprocessing problems or highly deviating anatomy."

In addition, if the segmentation fails completely, the specific scan cannot really be used in the analysis and its quality measure is not needed anyway.

Oh, most of the measures actually rely on the segmentation. Are segmentation errors accounted for in the tool? I am thinking specifically about "abnormal" brains that can be difficult for segmentation algorithms. At least at this point of the article, it's not clear.

Response: Yes, as the title of the paper and the header of this section state, our measures rely on segmentation. Hopefully, changing the title will make this clearer from the outset. Nevertheless, we have attempted to clarify this at the end of the section "Segmentation-based Image Quality Assessment":

“The [BWP] dataset includes images with varying levels of noise, inhomogeneities, and resolution. These image properties affect the segmentation accuracy of MRI processing algorithms (Figure 1B), and are therefore useful indicators of the quality of input data.“

"To accommodate various international rating systems, we have adopted a linear percentage and a corresponding (alpha-)numeric scaling." this doesn't match the complexity of the following explanation about the rather arbitrary range. I think a much more international and understandable rating would have been a 0 to 1 range. A 0.5 to 10.5 range is not helping users at all. As the rating is linear, I am struggling to see the added value of this choice.

Response: Thank you very much for sharing with us your view. We would disagree that our range is arbitrary. In fact, by examining the school grading systems across the world it was evident that the underlying match between percentage and grades is relatively consistent (though with regional differences), with numerical grades often falling between 1-10 (though the positive and negative endpoints differ based on the countries' historical background) that are partially also represented by letter grades or alternatively a percentage systems (range 0% to 100% or in other words 0 to 1). Typically, 50-60% of the points determine the threshold to pass the test. Our grading system therefore leverages the known classification. As the tool or its versions has been in use for some years and many users are familiar with it, we are concerned that changing it would cause more confusion than anything else. In addition, we use a root-mean-square like approach to combine the different ratings, which rely on this scaling to weight worse values stronger.

We further specified the rating system and referenced the extended description in the Method part.

"Although the BWP does not include the simulation of motion artifacts, these are in general comparable to an increase of noise in the BWP dataset by 2 percentage points." Maybe that should be justified with a reference? "in general" might be a bit light to justify not having a direct measure for something presented as important (motion artefacts) in the introduction and goal of the tool.

I think the absence of a noise estimation in the QC ratings should be more thoroughly justified.

Response: Thank you for mentioning this. We agree with you that it would be useful to have a reference to support our claim. However, as we are not aware of any specific research examining this particular topic, we have relied on our experience with data here. Additionally, although our measure development and evaluation relied on BWP, which does not include motion artifacts, we addressed this issue by testing our measures on the MR-ART dataset, which does include them (pp. 10-11). Moreover, to demonstrate our statement, we added a boxplot representing differences in SIQR between conditions with various amounts of motion in the MR-ART dataset (Figure 7).

Regarding the comment about the absence of noise estimation in the QC rating, we would like to point out that the noise **is** considered in our rating as “noise-to-contrast ratio” (NCR).

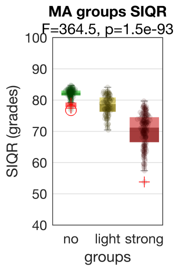

"To balance the sensitivity to different quality measures while ensuring that the necessary quality conditions are met, we apply an exponentially weighted averaging approach — similar to the root mean square (RMS) but using the fourth power and fourth root." Why is there no justification or references for these arbitrary choices? Why not the fifth root or tenth root? Why the square root and not an exponential or any other function?

Response: Thank you for raising this issue. While we agree with you that our choice seems arbitrary from the text, it actually stems from our tests. Artifacts cannot be compensated by other image features and we therefore focused on the exponential (root-mean-square-error-like) weighting to increase the differentiation of the outliers. In case of motion artifacts, we observed that even heavy motion only slightly reduced the average rating, so we used a higher power to increase the weighting of a single negative rating. The effect of motion artifacts on image analyses is mostly driven by the outcome of the processing, which we quantified in the BWP (and later also the MR-ART dataset) by Kappa and a systematic underestimation of GM. We tested several exponential weighting options. In the composite measure presented in the unrevised manuscript (where the inhomogeneity measure was included), there was a clear preference for the weighting of power 4 (see the image and table R1 below). However, in the revised version (with exclusion of the inhomogeneity rating), the strong preference for the weighting 4 is not as obvious. Nevertheless, the correlations alone are not a clear indicator. They are varying by tissue class, the segmentation, the combined ratings, as well as the dataset. We have focused here on the MR-ART dataset; however, many other datasets are available, but could not be included in the article. Therefore, we here provided the evidence in this dataset, while taking into account further tests that were run with datasets that were not included in this manuscript. Below is the figure representing the differentiation between groups of scans with different degrees of motion artifacts, with higher power weightings differentiating the groups better. Nevertheless, too high weightings became too sensitive for the worst feature.

As stated before, advanced users have the option to use the individual measures and get their own impression of the data. We here wanted to provide a simple human readable composite score to assess the quality of a scan.

Below is the figure representing the differentiation between groups of scans with different degrees of motion artifacts, with higher power weightings differentiating the groups better.

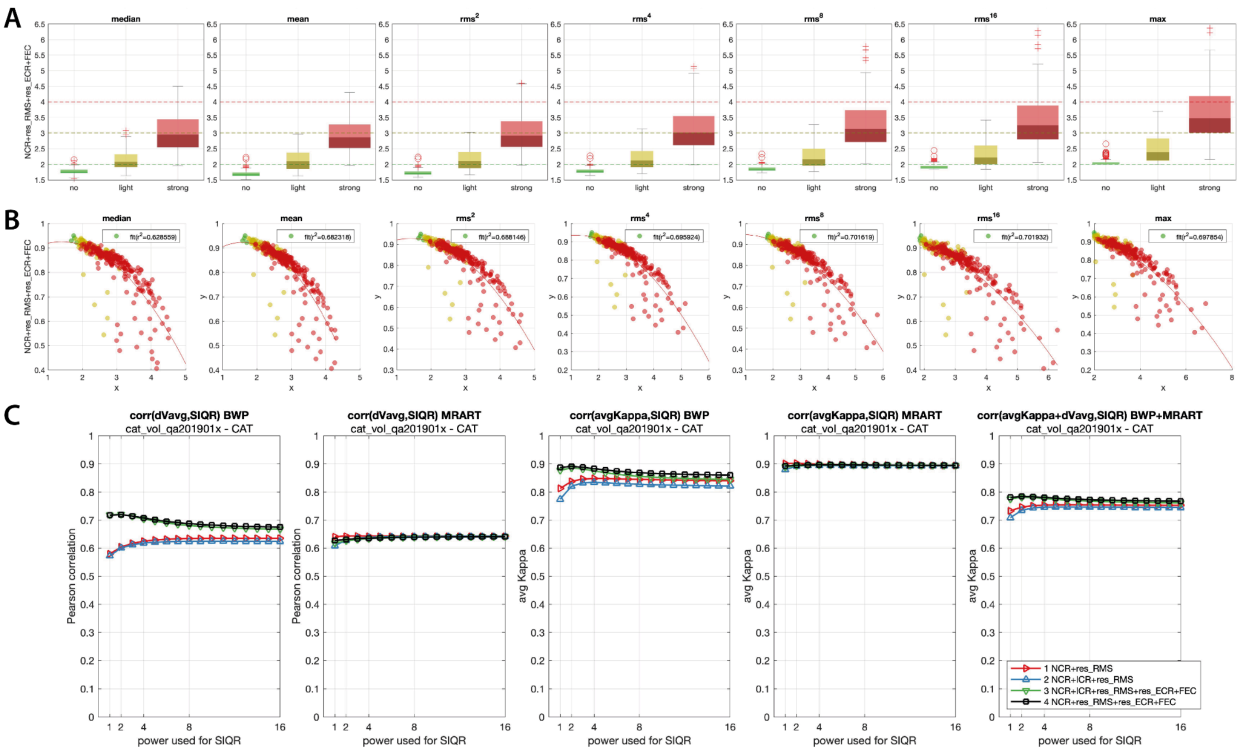

To sum up:

"To quantify the effect of interferences, we estimated the volume difference (ΔV) and the Kappa value to the artifact-free case (averaged over all tissue classes), where volume changes and Kappa statistics should be highly associated with the quality rating (Table 2). We finally selected the power 4 function as it is more sensitive to outliers."

Table R1: Results from version 201901

	Vol-BWP-avg	Vol-MRART-avg	Kappa-BWP-avg	Kappa-MRART-avg	avg
median	0.45575	0.63246	0.75774	0.89138	0.68434
mean	0.45575	0.63246	0.75774	0.89138	0.68434
rms^2	0.46668	0.63406	0.77304	0.89229	0.69152
**rms^4**	**0.48033**	**0.63088**	**0.78391**	**0.88453**	**0.69491**
rms^8	0.49132	0.62275	0.78591	0.86738	0.69184
rms^{16}	0.49507	0.61576	0.78415	0.85265	0.68691
max	0.49616	0.6112	0.78241	0.84319	0.68324

"Sample Normalization for Outlier Detection" It is unclear whether this is systematically applied or not. Is it a separate measure, or is it aggregated into another score? That measure could be relevant in

many cases but could also be really bad in some specific cases (for example, historical data where the "ideal" quality would probably be well below standards.

Response: We agree that this section could be described better and have introduced changes to the text to make it clearer (see pp. 9). The sample normalization results in another sample-specific rating (NSIQR - normalised SIQR) used to test how well the outliers (e.g. in the MR-ART dataset) can be identified. The rating (as described in the outlier detection) is part of the "Check Sample Homogeneity Tool" for sample evaluation before statistical analysis, and is also stored in the XML files for each subject with an extra field that saves basic information about the data (path, datetime, number).

Overall, our measures are defined by the typical ranges that structural MRI currently supports and that could potentially be used for tissue segmentation and surface reconstruction. As the BWP can be seen as a historical element, the principle definitions of noise, inhomogeneity and resolution still fit to today's structural data. We have tested here with a broad variability of data available from larger and smaller projects. However, the challenging part are ultra-high (7 Tesla) resolution images, where protocols are still in development and some public datasets suffer from interferences (e.g. extreme bias) that will probably play only a minor role in future standard acquisitions.

"raw (co-registered)" Well, it is not raw if it's co-registered. I suggest reformulation to avoid confusion with actual raw images.

Response: Thank you for pointing out this issue, we have omitted the wording in the revised manuscript. Although raw data (defined as the output of the Dicom conversion) present the default, researchers often use the term "raw" even if it includes preprocessing (e.g., resliced/resampled, defacing, skull-stripping, denoised, bias-corrected, or intensity normalized data) in different contexts.

The "Evaluation Concept and Data" section is very unclear. The need for a training-testing scheme is not explained, and the scheme itself is very arbitrary (choosing odd and even numbered files ordered by filenames). How does that splitting strategy help with generalisation? Why that specific split? Why not another? How do we know that split is not biased?

Response: We agree that this description appears arbitrary and we refined this section. Briefly, the split was used to define and scale the measures on another subset of data. We opted for a simple odd-even split since the BWP parameters are included in the filenames. This means that selecting every second data point provides a balanced and comprehensive sample that includes similar, but not identical, cases.

Finally, the selection of 6 scans also seems completely arbitrary. Overall, this section does not provide enough information to justify the seemingly arbitrary choices.

Response: Thanks for pointing this out. The dataset used for this analysis is highly diverse with regards to different scanning parameters and was not acquired by us. Six scans with different scan times, resolution, and parallel imaging, but similar with regard to other imaging parameters (TR/RE

times) were selected only to illustrate the working of the QC on an example. We have added our selection criteria for the scans in the description of the samples.

"Of note, obvious subject/scan-specific motion artifacts generally increase the scans' rating for about 1 grade, which corresponds to a decrease of 10 rps (and +0.5 grade / -5 rps for light artifacts), in comparison to the typical rating achieved by the majority of scans of the same protocol." This is incredibly vague! How are readers supposed to evaluate the quality control measures with this information?

Response: We agree that from this statement, the users cannot evaluate the quality measures, nevertheless, we have tried to provide evidence in our manuscript to showcase the validity and reliability of the measures. The readers can rely on the numerical output of the toolbox to decide about inclusion or exclusion of the scan. The above statement was meant as a general observation and not as evidence, as we currently have no measure or classification for motion artifacts.

Discussion:

"as this is more relevant for segmentation and surface reconstruction (Ashburner et al., 2005)." A lot of work has been done in these domains in 20 years; this reference, however solid, is not enough to justify that choice. This might not be relevant with the methods developed in the last 20 years.

Response: Thank you for this observation. We updated the references in this part of the manuscript:

"Moreover, the proposed intensity-based measures are normalised by (minimum) tissue contrast rather than signal intensity, as the *separation between brain tissues, especially the GM and WM, is essential* for segmentation and surface reconstruction *(Fischl et al. 2012, Gaser et al., 2025)*."

"with a power of 4 rather than 2, to place greater emphasis on the more problematic aspects of image quality." Still not enough to justify that choice. The authors failed to convince me that one single score is better than reporting all the measures significantly, as different quality measures will influence different tasks. A very practical example is the fact that the vast majority of acquisitions in clinical settings, the resolution is anisotropic (though less with T1 images nowadays, historical datasets will still have it). This anisotropy is not necessarily an issue for human diagnosis, for example; however, aggregating all the scores in one might hide that a low-quality measurement might not affect the specific downstream task. Coupled with the lack of justification for the factor scalings, this choice of a single score is a significant negative point for the tool.

Response: Indeed, we agree that for particular use cases, the specific (raw/unscaled) measures are preferable and can also be used. We do not object to that and provide the unscaled measures as an output in xml files. However, the composite measure was designed to meet the needs of the non-advanced users of structural preprocessing tools (for T1w-based segmentation and surface reconstruction) to get a simple estimate of the quality of their data to identify critical cases. We extended the discussion:

"Nevertheless, raw quality measures are also available in the XML files, allowing advanced users to perform detailed inspections." (p.24)

With regard to the lack of justification for scaling, we have addressed this in the Averaging section in the Method part.

Data availability:

Where can the sources of these specific tools be accessed?

Response: The raw data is available from the original sources, whereas processed data is available from the Gigascience server. We improved the data and code availability report in the revised manuscript.

Reviewer #2:

Technical Note GIGA-D-25-00085 introduces a segmentation-based quality control (QC) framework for T1-weighted structural MRI integrated into the CAT12 toolbox. The approach defines five interpretable image quality metrics—noise-to-contrast ratio (NCR), inhomogeneity-to-contrast ratio (ICR), resolution score (RES), edge-to-contrast ratio (ECR), and full-brain Euler characteristic (FEC)—which are combined into a composite Structural Image Quality Rating (SIQR). The tool aims to provide a standardized, interpretable scoring system for identifying poor-quality scans, with validation across simulated datasets and real-world imaging data.

Strengths

The manuscript addresses a critical need in neuroimaging by presenting an automated, interpretable, and practical framework for quality control of T1-weighted structural MRI. By integrating multiple segmentation-derived metrics into a single Structural Image Quality Rating (SIQR), the approach enables fast, standardized assessment of image quality. The tool is embedded in the widely used CAT12/SPM ecosystem, facilitating adoption, and it is validated across a range of synthetic and real-world datasets. The scoring system is designed with user accessibility in mind, offering a clear

grading scale and robust detection of motion-related artifacts, making it particularly well-suited for use in large-scale research and clinical imaging settings.

Weaknesses

1. **Ambiguity of scope and segmentation dependency.**

A fundamental issue with the manuscript is its failure to clearly define the proposed QC framework's intended scope. If it is intended as a general-purpose image quality assessment tool, then several limitations become critical: its reliance on accurate tissue segmentation (1), its omission of background signal (2), its restricted validation within the CAT12 pipeline (3), and its lack of demonstrated interoperability with other workflows or populations. The method's reliability across different segmentation tools (e.g., FreeSurfer, FSL, SynthSeg) or in anatomically atypical populations (e.g., pediatric, lesioned brains) is untested (4). Conversely, if the framework is intended as a CAT12-specific internal QC tool, then the presentation is misleading. The inclusion of cross-tool benchmarks (e.g., MRIQC) (5), the use of generalized grading schemes, and the claims of robustness (6) give the impression of broader applicability. In this narrower interpretation, some concerns (e.g., pipeline generalization) would be less pressing, but others—such as the MRIQC comparison—become more problematic and unjustified. The manuscript would benefit greatly from explicitly stating whether the goal is a broadly applicable QC solution or a targeted add-on for CAT12 workflows.

Response: We appreciate your constructive feedback. In the revised version, we tried to implement the points that you raised as much as we could. The scope of our manuscript was not to present the tool as a general-purpose image quality assessment tool and we have hopefully made this clearer in the revised version. We outline early on that the tool is useful only for evaluation of structural T1-weighted images used for tissue segmentation and surface reconstruction within the SPM/CAT12 ecosystem. We address your other concerns below:

1) Reliance on accurate tissue segmentation

   We see the potential disadvantage of relying on tissue segmentation. We acknowledge that the segmentation can fail, and we have simulated extreme cases of segmentation problems to test their effect on the quality measures (results available in the Supplementary material). These perturbances affect the variation in the quality measures; however, this variation is generally smaller than that caused by typical (light) motion artifacts. We have mentioned the potential problems with segmentation also in the Discussion, where we pointed out that severe segmentation problems can be identified by using covariance analysis of the segmentation.

("Multivariate outlier detection schemes that are typically applied based on the processed data of a sample in the normalized feature space, using similarity analysis of normalized GM data (e.g. the Gram matrix or kernels) in CAT12 (see Software section), can be used to detect outliers with preprocessing problems or highly deviating anatomy. However, the proposed image quality assessments are specifically designed to measure differences of image quality (in native space) rather than segmentation accuracy (in normalized space) or anatomical properties, such as stroke lesions, and can therefore be used in addition to previously mentioned outlier detection schemes to identify cases where image artifacts could bias analysis." p.21)

2) Omission of background

   We agree that the omission of background does not allow our QC tool to find certain artifacts that are prominent in the background. However, the main purpose of our QC tool is to determine the usefulness of the image for the prospective brain analysis. If the artifacts such as ghosting and wrap-around affect large areas of brain tissue, they are accounted for in the quality measure, but not if they are only in the background as this is of no use to the final analysis. As we mentioned in Discussion: *"For instance, in cases when locally limited or mild artefacts affect regions that are not relevant to the study (e.g., if the study focuses on frontal regions, cerebellar artefacts from jaw movements are acceptable) or whenever lower preprocessing accuracy is acceptable (e.g., for local alignment of brain surfaces or atlases for other modalities)."*

3) Restricted validation to CAT12

   Although the analysis was focused on CAT12, we also presented the results in SPM segmentation (Figure 7) as both of these tools come from a similar ecosystem. Since our scope was not to provide an overarching image quality control tool, we did not focus on comparison with segmentation from other tools. We specified a scope in the revised manuscript.

4) Test in other populations and lesioned brains

   The test in lesioned brain was performed in the ATLAS database (Figure 6B). We also tested the toolbox internally on data of children, however, we did not present the results in the current manuscript. We added the analysis on children in the revised version (Figure 6C & D) and used the children sample to better explain our outlier detection scheme.

5) Inclusion of comparison to MRIQC

We acknowledge that the comparison to the MRIQC might have appeared unfair and that was not our intention (we expand upon this below at #4). We have corrected this section in the manuscript.

6) Claims about generalized grading schemes, robustness

   We believe that the claims about the usefulness and robustness still hold within the limited scope of the SPM/CAT framework and hope the revised manuscript properly addresses your concerns.

2. **Lack of compliance with GigaScience reproducibility standards.**

The manuscript does not currently meet GigaScience's data and code availability requirements. The code used to generate results and figures is not publicly accessible—only available upon request—which directly conflicts with the journal's expectations for open, reproducible research. Similarly, while the data are drawn from public sources, the manuscript lacks direct links, accession numbers, or DOIs for the datasets used, and provides no clarity on data preprocessing or analysis scripts. There is also no reference to licensing for the CAT12 toolbox or the code used in the study, and no reproducibility capsule (e.g., containerized environment, workflow script) is offered. These omissions limit the transparency and reusability of the work and must be addressed to comply with the FAIR principles and GigaScience's editorial policies.

Response: The code and data are available, but the Gigascience FTP was set up during the submission process and were therefore not included in the main text. We improved this in the revised version.

3. **Mischaracterization of background-based IQMs.**

In the "SIQR measure development" section, the manuscript states: "Image quality measures are commonly estimated from the image background (Mortamed et al., 2008; Esteban et al., 2017)." This statement is factually incorrect and conceptually misleading. First, the citation is incorrect—Mortamed should be Mortamet (2009). Second, it misrepresents tools like MRIQC, where most quality metrics are computed within brain tissue, including CJV, SNR, and contrast-based measures. Third, the authors entirely omit recent work (e.g., Pizarro et al., 2016; Provins et al., 2025\) showing that artifacts such as ghosting, wrap-around, and motion often manifest more clearly in the background, due to the nature of Fourier reconstruction. By excluding background regions, the proposed method may miss artifacts that are visible but lie outside the segmented brain, and the trade-offs of this design decision are not discussed. The rationale based on defacing is only partial: defacing typically removes the face, not the broader background, where artifact signals often dominate. The statement

as written oversimplifies QC practices and signals a bias toward justifying the framework's internal constraints rather than engaging with the full methodological landscape.

References:

Provins, C., … Esteban, O. (2025). Removing facial features from structural MRI images biases visual quality assessment PLOS Biology. doi:[10.1371/journal.pbio.3003149](https://journals.plos.org/plosbiology/article?id=10.1371/journal.pbio.3003149) (OA).

Pizarro RA, et al. (2016). Automated quality assessment of structural magnetic resonance brain images based on a supervised machine learning algorithm. Front Neuroinf. 10\. doi:[10.3389/fninf.2016.00052](https://doi.org/10.3389/fninf.2016.00052).

Response: Thank you for the correction, we have updated the statement in the revised manuscript. Nevertheless, we would argue that even if the artifacts that you mention are more clearly visible in the background, if they affect the brain, they are also visible in the brain tissue segmentation and our measures account for that. As we have shown in the revised version of the manuscript, the SIQR measure is highly associated with the summary statistics of the background from MRIQC. Moreover, it is not only defacing, but also other varying properties of the available structural images that limit the extraction of quality measures from the background (e.g., different backgrounds, for instance in MP2RAGE protocols, skull-stripped data etc.).

4. **Underdeveloped and opaque benchmarking against MRIQC.**

The benchmarking against MRIQC is reported only in the Results section, with no corresponding description in the Methods. It is surprising that MRIQC is not mentioned by name until page 14, despite the Esteban et al. (2017) reference appearing earlier in a different context. This suggests that the treatment of MRIQC—a widely adopted, general-purpose QC tool—has not been as thorough or fair as would be desirable. Key methodological details are missing: the authors do not explain how MRIQC was executed, how specific features (e.g., snr_wm, cjv) were selected, or whether a multivariate classifier was considered. Given that MRIQC's full model leverages multiple features simultaneously, limiting the comparison to univariate metrics weakens the validity of the claim that SIQR outperforms existing approaches. A more balanced, transparent benchmarking setup would strengthen the manuscript considerably. This benchmarking also mentions an "SPM12-based" QC performance but does not clarify how and why this comparison is made.

Response: Thank you for pointing this out. Perhaps this became unclear during the analysis and writing-up process, but as we did not intend to provide an alternative to MRIQC in the form of a general-purpose tool, the comparison was not included in the Method section. The analysis was primarily intended to test the performance of our measures in the MR-ART dataset with the available expert ratings. However, we noticed that the MRIQC measures were also available and additionally assessed the validity against the measures from a related tool. We agree that the initial version of the

manuscript presented an unfair and underreported comparison, so we have updated and rephrased the relevant section.

Regarding the use of SPM, we included it to check if the measure is useful also for another input segmentation, where we selected the Unified Segmentation from SPM as it is directly available in the same ecosystem. This allowed us to test the expected atrophy in case of motion also for another tool.

5. **No analysis of failure cases.**

The manuscript does not present examples of false positives or false negatives—cases where SIQR fails to align with visual inspection or known ground truth. Without understanding when and why the metric fails, users cannot judge the risk of misclassification or apply it conservatively in sensitive datasets.

Response: Thank you for this suggestion. We have included this part in the Supplement.

Minor Issues

* Figure 7 could benefit from clearer annotation of thresholds and misclassified cases to help interpret the ROC curves.

Response: Thank you for your comment. We have tried to improve the readability of the figure.

* While the title *"The Good, the Bad, and the Ugly"* is a play on the classic western film, this informal or humorous reference may be perceived as inappropriate in a scientific context—especially for a methods paper intended to support standardization and reproducibility. The title does not convey the technical scope or scientific contribution of the work, which may undermine its visibility and perceived rigor. A more descriptive and neutral title—e.g., "Segmentation-Based Quality Control of Structural MRI using the CAT12 Toolbox"—would better reflect the content and purpose of the manuscript.

Response: We appreciate this helpful comment. We changed the title to better represent the content of the paper.

* While the authors validate their approach against synthetic degradations and segmentation-derived kappa scores, they do not sufficiently leverage human expert QC ratings. Greater engagement with visual QC standards would make the case for SIQR's practical value more compelling.

Response: Expert ratings from MR-ART were used and we have stated this more clearly in the revised manuscript.

I was given access to the supporting data but chose not to proceed with reproducibility checks at this stage, as the manuscript does not currently meet GigaScience's basic standards for code and data transparency. I look forward to reviewing a revised version that clearly defines the scope of the method, improves methodological transparency, and brings the manuscript into compliance with the journal's reproducibility and FAIR data principles.

Best regards,

Oscar Esteban, Ph. D.

Research and Teaching FNS Fellow

Dept. of Radiology, CHUV, University of Lausanne

Reviewer #3:

The paper describes an alternative way to QC T1w images with 2 major innovations: a different set of metrics not relying on background and a global score that combines those metrics. In addition, all of this is integrated in a well maintained toolbox allowing easy usage.

I only have suggestions (ie it does not have to be all done) as the overall paper is well written, easy to follow and analyses well conducted.

Thank you very much for your positive feedback. We have included your suggestions in the Supplement.

P6 NCR: it can be nice to demonstrate how it performs compared to traditional CNR (mean of the white matter intensity values minus the mean of the gray matter intensity values divided by the standard deviation of the values outside the brain) -- differs markedly because of background difference for sure, since you have plenty of test images you could show that more clearly (later in the method, based on what criteria/reason 'local' is defined as 5*5*5?)

Response: Thank you for your suggestion. We have added the comparison of CNR/NCR to the Kappa statistics to the Methods section (Figure 10).

P7 ECR should capture something similar to Entropy Focus Criterion, would be nice to provide a direct comparison

Response: Indeed, the measures are associated (rho = -0.5292) but the ICR was even a bit higher correlated (rho = -0.6852). We added the correlation matrix to the Supplement (Figure S6).

P8 typo, you meant equation 2

Response: Thank you for pointing it out. We have corrected the typo.

P8 SIQR I'm guessing you have experimented with the power function - maybe a side note to share your experience of why or how it works better than eg square

Response: We added the evaluation and further description to the supplement (Figure S1).

Dr Cyril Pernet

Reviewer #4:

Reproducibility report for: The Good, the Bad, and the Ugly: Segmentation-Based Quality Control of Structural Magnetic Resonance Images

Journal: GigaScience

ID number/DOI: GIGA-D-25-00085

Reviewer(s): Laura Caquelin, Department of Clinical Neuroscience, Karolinska Institutet, Sweden [Worked on reproducing the results and wrote the report]

Tobias Wängberg, Department of Clinical Neuroscience, Karolinska Institutet, Sweden [Worked on reproducing the results]

----

1. Summary of the Study

The study addresses how variability in magnetic resonance images quality, especially from motion artifacts or scanner differences, can affect structural image analysis. It proposes a quality assessment framework for T1-weighted images based on tissue classification and standardized image quality

measures. The method is shown to be robust across datasets and conditions, helping to detect outliers and control for motion-related artifacts.

----

2. Scope of reproducibility

According to our assessment the primary objective is: to develop and validate a standardized framework for assessing the quality of structural (T1-weighted) MRI images, enabling the detection of artifacts on simulated data.

- Outcome: Quantitative quality ratings derived from image properties such as noise-to-contrast ratio (NCR), inhomogeneity-to-contrast ratio (ICR), resolution score (RES), and edge-to-contrast ratio (ECR) and Full-brain Euler characteristic (FEC) combined into a Structural Image Quality Rating (SIQR).

- Analysis method outcome: Not precised in the manuscript, but with the Matlab script we identified that the quality scores were correlated using Spearman's rank correlation, and statistical significance was assessed using p-values computed using MATLAB's built-in method.

- Main result: Results are presented in Figure 5. "The evaluation on the BWP test dataset showed that most quality ratings have a very high correlation (rho > .950, $p < .001$) with their corresponding perturbation and a very low correlation (rho < |0.1|) with the other tested perturbations (see table in Figure 5A & C). This suggests considerable specificity of the proposed quality measures. The combined SIQR score also showed a very strong association with the segmentation quality kappa (rho = -.913, $p < .001$) and brain tissue volumes ($rho_{CSF/GM/WM}$ = -.472/-.484/.736, $p_{CSF/GM/WM} < .001$) (Figure 5B). [...]
The edge-based resolution measure ECR, on the other hand, generally performed better (rho = .828, $p < .001$), but was more affected by noise (rho = .306, $p < .001$) and inhomogeneity (rho = .223, $p < .001$) than other scores."

----

3. Availability of Materials

a. Data

- Data availability: Open
- Data completeness: Complete, all data necessary to reproduce main results are available
- Access Method: Private journal dropbox but also available on Github repository
  - Repository: https://github.com/ChristianGaser/cat12

-Data quality: Structured

b. Code

- Code availability: Share in the private journal dropbox but also open
- Programming Language(s): Matlab
- Repository link: https://github.com/ChristianGaser/cat12
- License: GPL-2.0 License
- Repository status: Public
- Documentation: Readme file

----

4. Computational environment of reproduction analysis

- Operating system for reproduction: MacOS 15.5 (reviewer 1) and MacOS 15.1 (reviewer 2)
- Programming Language(s): Matlab
- Code implementation approach: Using shared code
- Version environment for reproduction: Matlab R2024b Update 6 (24.2.2923080) - Trial version

----

5. Results

5.1 Original study results

- Results 1: Figure 5 C (see screenshot)

5.2 Steps for reproduction

->Finding how to reproduce the results

- Issue 1: The methods section lacks sufficient detail regarding the statistical methodology, and the relevant information is not fully provided in the GitHub repository.

-- Resolved: A message has been sent to the authors requesting further clarification on the methodology and additional resources (scripts/data) needed to reproduce the results. The script to reproduce the results is "cat_tst_qa_bwpmaintest.m".

-> Reproduce the results using the "cat_tst_qa_bwpmaintest.m" script.

- Issue 2: To run the script "cat_tst_qa_bwpmaintest.m", the "eva_vol_calcKappa" function is missing.

-- Resolved: The script was shared and added to the Github repository.

- Issue 3: While running the script, the following error message encountered:

Assigning to 0 elements using a simple assignment statement is not supported. Consider using comma-separated list assignment.

Error in cat_tst_qa_bwpmaintest (line 481)

```
default.QS{find(cellfun('isempty',strfind(default.QS(:,2),'FEC'))==0),4} = [100, 850];
```

-- Resolved: This error stops the execution of the script. After discussion with the authors, the exact cause of the error encountered at line 480 was not directly identified. We exchanged and compared our environments at the point just before the error occurred and observed notable differences between them. Our environment is almost empty. The authors identified that the default variable is missing from our environment, even though it is referenced at line 437 by a call to the cat_stat_marks function.

We confirmed that all required dependencies were installed (including Statistics toolbox, SPM and CAT12), and that we had access to all the necessary data.

To ensure the issue was not due to user error, the code was independently executed by two reviewers. The error was consistently reproduced in both cases. About the setup, I specified to the authors:

"To summarize my setup:

* I have installed SPM, CAT, and the Statistics Toolbox.
* I downloaded all datasets from the GigaScience server.

*   I also downloaded the IXI T1 data, but I've only kept the version available on the GigaScience server in my working directory. Is the version from GigaScience sufficient? I had presumed that this dataset was pre-processed and ready to use, so I ignored the time-consuming pre-processing step. Your last email seems to confirm this point."

The authors answered that:

« Yes, this is correct. However, both directories have to be combined so that the original IXI images and the processing files are included. »

In an attempt to proceed, we modified the portion of the code that triggered the error:

```
######
% FEC
  FECpos = find(cellfun('isempty',strfind(default.QS(:,2),'FEC'))==0);
  try
    warning off;
    [Q.fit.FEC, Q.fit.FECstat]  = robustfit(Q.FECgt(M,1),Q.FECo(M,1));
    warning on;
    if ~isempty(FECpos)
      default.QS{FECpos,4} = round([Q.fit.FEC(1) + Q.fit.FEC(2), Q.fit.FEC(1) + Q.fit.FEC(2) * 6], -1);
    end

  catch
    Q.fit.FEC = [nan nan]; Q.fit.FECstat = struct('coeffcorr',nan(2,2),'p',nan(2,2));
    if ~isempty(FECpos)
      default.QS{FECpos,4} = [100 850];
    end
  end
#####
```

Following this adjustment, the end of the script "cat_tst_qa_bwpmaintest.m" ran without issue and generated output results:

Finally, the error was identified after numerous exchanges with the authors. The function "cat_stat_marks", available in the Github repository, was not shared in the FTP server. With this

function added, the script runs correctly. Please note that the link to the Github repository where the software code can be found is not specified in the manuscript.

-> Compare the results reproduced and the original results

- Issue 4: Discrepancy between reproduced results, output results provided by the authors and the original results shown in Figure 5C.

-- Unresolved: We reproduced the figures and the corresponding output table using the modified "cat_tst_qa_bwpmaintest.m" script. We ran the script using the only default QC version selected in the script ("cat_vol_qa201901x"). By comparing our output with the result files shared by the authors, we were able to confirm that we had executed the correct pipeline.

However, we encountered a discrepancy: neither the generated file in our run (tst_cat_col_qa201901x_irBWPC_HC_T1_pn9_rf100pC_vx200x200x200rptable.csv) nor the corresponding file provided by the authors (outputs from BWPmain_full_202504) matched the numerical values presented in Figure 5C of the manuscript.

We contacted the authors to clarify whether the default QC version used in the script was indeed the one produce the figure. In response, they confirmed:

"All figures should show the results of this QC version although I had the plan to run a final check update after the reviewer comments (the figures are finally arranged in Adobe Illustrator)."

Therefore, although the correct version of the QC was used, the differences in the results shown in Figure 5C remain unexplained. This issue is still unresolved.

Response: Thank you for pointing this out. As mentioned in the initial communication with the editor, we estimated the values for the table from the images preprocessed with an earlier CAT12 version. Unfortunately, a part of preprocessed scans was accidentally deleted during the upload of the images to the Gigascience server, and had to be reprocessed, for which a current CAT12 version was used. The differences in the values were therefore expected (albeit not to a drastic extent), but we decided to correct the figures after the review process. We now updated the values in the figures and tables. Of note, the QC version that the reviewers used is the same as used for the manuscript.

5.3 Statistical comparison Original vs Reproduced results

- Results: Screenshot of reproduced tst_cat_vol_qa201901x_irBWPC_HC_T1_pn9_rf100pC_vx200x200x200_rptable.csv table

- Comments: Several p-values in the reproduced results appear as exactly 0 (0.00000000e+00), which is unlikely from a statistical point of view. It is possible that these values are just extremely small and were rounded down. However, this could also point a problem in the script. Further investigation would be needed to determine the cause.

Response: Thank you for this observation. The result of 0.00000000e+00 signifies a statistically significant result. Due to the smallness of the value, however, the number cannot be represented by a computer (i.e., arithmetic underflow). This is a common phenomenon in statistical programs. Because the probability of getting such or more extreme results when the null hypothesis is true cannot be 0, we reported this result as p<.0001.

- Errors detected: Values in Figure 5C do not correspond to those provided by the authors in the FTP server in the files (tst_cat_vol_qa201901x_irBWPC_HC_T1_pn9_rf100pC_vx200x200x200_rptable.csv). Multiple inconsistencies were observed, suggesting potential errors in the manuscript figure or mismatches between file versions (see file Comparison_original_rptable_vs_fig5C_data.csv for comparison).

Response: Thank you for pointing this out. As mentioned before, we estimated the values for the table from the images preprocessed with an earlier CAT12 version, but had to reprocess the images due to the accidental deletion of files during upload. A newer version of CAT12 was used for the preprocessing for the images on the server. The differences in the values were therefore expected (albeit not to a drastic extent), but we decided to correct the figures after the review process. We now updated the values in the figures and tables.

(Screenshot of Figure 5C)

(Screenshot of the original output corresponding to the Figure 5C)

- Statistical Consistency: The reproduced correlation table (tst_cat_vol_qa201901x_irBWPC_HC_T1_pn9_rf100pC_vx200x200x200_rptable.csv). differs from the original in terms of r-values and p-values.

Compared to the Figure 5C, the reproduced r-values do not all match those shown in the figure. P-values cannot be directly compared to Figure 5C, as they are represented by a color gradient without a scale or legend, making direct comparison impossible.

Response: Thank you for pointing this out - the same comment applies as above. We added the full correlation table with rho and p-values in the supplement.

----

6. Conclusion

- Summary of the computational reproducibility review

The computational reproducibility of the main result we identified for the study is partially achieved. After several technical issues related to missing functions, I was able to execute the script to reproduce values of Figure 5C ("cat_tst_qa_bwpmaintest.m") and obtain ouput results. However, discrepancies were observed when comparing the reproduced results (tst_cat_col_qa201901x_irBWPC_HC_T1_pn9_rf100pC_vx200x200x200rptable.csv) to both:

- the output file provided by the authors, and
- the original results presented in figure 5C of the manuscript.

Notably, the output file provided by the authors and the results in figure 5C do not match either, indicating potential errors or file versions mismatches.

Additionally, many p-values in the reproduced results are equal to 0, which suggests a formatting issue or a problem in the script. Figure 5C also lacks a scale, legend detail, or supplementary data to make possible to verify p-values (assuming the color gradient represents the p-values).

- Recommendations for authors

We strongly recommend the authors to:

-- Ensure all essential code and functions are included in the shared repositories. Some necessary files were not included in the FTP server provided with the paper. Although the GitHub repository (https://github.com/ChristianGaser/cat12) was shared with the journal, but it is not referenced in the manuscript, making it difficult for external users to locate.

Thank you very much for your comment. The repository is referenced already in the Introduction (p. 4, line 83) as the quality measure is connected to the CAT12 ecosystem.

-- Add detailed documentation of the statistical methods: the current manuscript lacks sufficient information regarding the statistical methodology used, at least for the purpose of the reproducibility review. Please, include detailed explanation of statistical tests, packages and parameter settings (e.g. QC version) to improve reproducibility. Thank you, we now explicitly mentioned the use of Spearman correlation in the Method section.

-- Clarify the versioning and outputs for the figures: there is a lack of clarity regarding which specific data outputs were used to generate figure 5C. Providing metadata or links to the exact output file used would help to resolve this issue.

As noted above, the images on the server were preprocessed with another version of CAT12 as was used for writing the article. We have updated the values in the revised manuscript.

-- Provide raw numerical data behind figures: figure 5C seems to display p-values using a color gradient but no scale or legend is provided. Sharing the raw data used would allow the comparison and the reproducibility of the figure.

Thank you for your remark, we added the correlation analysis output to the Supplement.

-- Improve the clarity of execution instructions and address potential p-values issues: the issue with p-values showing up as exactly 0 in the reproduced results might be caused by differences in the environment setup, such as missing variables, different software versions, or skipped steps before running the script. Improving the instructions for setting up the environment and running the would help prevent issues and facilitate reproducibility.

Response: Thank you for your suggestion. Please check the comment on the arithmetic underflow above.
