# Supplementary material for: Segmentation-based quality control of structural MRI using the CAT12 toolbox
Source: Gigascience. 2025 Nov 29;14:giaf146. doi: 10.1093/gigascience/giaf146 (PMC12758382; doi:10.1093/gigascience/giaf146)
Supplement: giaf146_Authors_Response_To_Reviewer_Comments_Original_Submission [file giaf146_authors_response_to_reviewer_comments_original_submission.pdf]

Author's Response To Reviewer Comments

See also revision letter PDF.

Reviewer response:
Reviewer #1:
The article presents a valuable effort towards standardising quality control methods and their evaluation. However, too many choices seem arbitrary without sufficient justification, and too many sections are unclear. Overall, the quality of the work cannot be fully assessed in the current state of the manuscript, and major revisions are needed to correct that. There is also not enough comparison (one) with other methods and no way of evaluating whether these measures are relevant to actual downstream imaging uses. Additionally, the article's goal is highly unclear and led me to think the segmentation measures were part of the QC pipeline until I read the discussion ... Nothing until the discussion explains that the segmentation measures are used to evaluate the single SIQR score output of the QC pipeline.
Response: Thank you very much for your feedback. Although the use of the segmentation in the conceptualisation of the QC tool is stated in the manuscript's title, we acknowledge that the playful reference to the movie title probably obscured the scope of this work. We have therefore decided to change the title of the revised manuscript. We also agree that various arbitrary choices were made during the development of the tool, and we would like to point out that these choices were motivated by practical experience with data, which is why there are limited references for many of them. Nevertheless, we have tried to address most of the points raised in your review and have rewritten various parts of the manuscript accordingly.
Comments:
"All measures and tools are part of the Computational Anatomy Toolbox (CAT; https://neuro-jena.github.io//cat, Gaser et al., 2024) of the Statistical Parametric Mapping (SPM; http://www.fil.ion.ucl.ac.uk/spm, Ashburner et al. 2002) software and also available as a standalone version (https://neuro-jena.github.io/enigma-cat12/#standalone)." I cannot really expect everyone to avoid Matlab tools. Still, Matlab is a drag to the development of scalable tools nowadays (every system admin's nightmare is to have to try to make Matlab tools run on high-performance computing servers).
Response: We agree with you that the mentioned dependency on Matlab is not in line with open science principles. We are therefore currently developing tools that do not rely on Matlab. Nevertheless, we would like to point out that both SPM and CAT12 (and the QC tool) can be run as standalone versions (also on a high-performing clusters), and that the concept described here (simple use case specified grading, specific evaluation aspects) can be transferred to other tools, programming languages or platforms. However, we feel that the complete change of the programming language and platform would be beyond the scope of this manuscript.

"such as noise, inhomogeneities, and resolution (Figure 1B)." At this point in the article, it's a bit unclear how that works in Figure 1B.
Response: We have tried to improve the clarity in the revised part. Figure 1B represents how two segmentation algorithms are affected by varying levels of noise, inhomogeneities and resolution from the BWP dataset (revised part in italic):
"(B) The segmentation accuracy can be quantified by the kappa similarity statistic (Cohen, 1960), here presented for two segmentation approaches on simulated images (AubertBroche et al., 2006), where larger levels of noise, inhomogeneity, or lower resolutions result in a worse overlap with the full-resolution image without interference."

"It is assessed within optimized cerebrospinal fluid (CSF) and white matter (WM) regions." Then, the NCR relies on the segmentation, right? What if the segmentation fails?
Response: Correct. As you pointed out, the NCR and all other measures rely on segmentation. All image assessments rely to some extent on some kind of segmentation, which defines regions to quantify the measure and scaling. This can fail or bias the (quality) analysis. However, tissue segmentations are broadly available, accurate, and robust, and severe segmentation failures in CAT12 can be identified using covariance analysis of the segmentation as mentioned in the Section "Software" (Figure 4) and Discussion (p. 21):
"Multivariate outlier detection schemes that are typically applied based on the processed data of a sample in the normalized feature space, using similarity analysis of normalized GM data (e.g. the Gram matrix or kernels) in CAT12 (see Software section), can be used to detect outliers with preprocessing problems or highly deviating anatomy."

In addition, if the segmentation fails completely, the specific scan cannot really be used in the analysis and its quality measure is not needed anyway.

Oh, most of the measures actually rely on the segmentation. Are segmentation errors accounted for in the tool? I am thinking specifically about "abnormal" brains that can be difficult for segmentation algorithms. At least at this point of the article, it's not clear.
Response: Yes, as the title of the paper and the header of this section state, our measures rely on segmentation. Hopefully, changing the title will make this clearer from the outset. Nevertheless, we have attempted to clarify this at the end of the section "Segmentation-based Image Quality Assessment":
"The [BWP] dataset includes images with varying levels of noise, inhomogeneities, and resolution. These image properties affect the segmentation accuracy of MRI processing algorithms (Figure 1B), and are therefore useful indicators of the quality of input data."

"To accommodate various international rating systems, we have adopted a linear percentage and a corresponding (alpha-)numeric scaling." this doesn't match the complexity of the following explanation about the rather arbitrary range. I think a much more international and understandable rating would have been a 0 to 1 range. A 0.5 to 10.5 range is not helping users at all. As the rating is linear, I am struggling to see the added value of this choice.
Response: Thank you very much for sharing with us your view. We would disagree that our range is arbitrary. In fact, by examining the school grading systems across the world it was evident that the underlying match between percentage and grades is relatively consistent (though with regional differences), with numerical grades often falling between 1-10 (though the positive and negative endpoints differ based on the countries' historical background) that are partially also represented by letter grades or alternatively a percentage systems (range 0% to 100% or in other words 0 to 1). Typically, 50-60% of the points determine the threshold to pass the test. Our grading system therefore leverages the known classification. As the tool or its versions has been in use for some years and many users are familiar with it, we are concerned that changing it would cause more confusion than anything else. In addition, we use a root-mean-square like approach to combine the different ratings, which rely on this scaling to weight worse values stronger.
We further specified the rating system and referenced the extended description in the Method part.

"Although the BWP does not include the simulation of motion artifacts, these are in general comparable to an increase of noise in the BWP dataset by 2 percentage points." Maybe that should be justified with a reference? "in general" might be a bit light to justify not having a direct measure for something presented as important (motion artefacts) in the introduction and goal of the tool.
I think the absence of a noise estimation in the QC ratings should be more thoroughly justified.
Response: Thank you for mentioning this. We agree with you that it would be useful to have a reference to support our claim. However, as we are not aware of any specific research examining this particular topic, we have relied on our experience with data here. Additionally, although our measure development and evaluation relied on BWP, which does not include motion artifacts, we addressed this issue by testing our measures on the MR-ART dataset, which does include them (pp. 10-11). Moreover, to demonstrate our statement, we added a boxplot representing differences in SIQR between conditions with various amounts of motion in the MR-ART dataset (Figure 7).
Regarding the comment about the absence of noise estimation in the QC rating, we would like to point out that the noise is considered in our rating as "noise-to-contrast ratio" (NCR).

"To balance the sensitivity to different quality measures while ensuring that the necessary quality conditions are met, we apply an exponentially weighted averaging approach — similar to the root mean square (RMS) but using the fourth power and fourth root." Why is there no justification or references for these arbitrary choices? Why not the fifth root or tenth root? Why the square root and not an exponential or any other function?
Response: Thank you for raising this issue. While we agree with you that our choice seems arbitrary from the text, it actually stems from our tests. Artifacts cannot be compensated by other image features and we therefore focused on the exponential (root-mean-square-error-like) weighting to increase the differentiation of the outliers. In case of motion artifacts, we observed that even heavy motion only slightly reduced the average rating, so we used a higher power to increase the weighting of a single negative rating. The effect of motion artifacts on image analyses is mostly driven by the outcome of the processing, which we quantified in the BWP (and later also the MR-ART dataset) by Kappa and a systematic underestimation of GM. We tested several exponential weighting options. In the composite measure presented in the unrevised manuscript (where the inhomogeneity measure was included), there was a clear preference for the weighting of power 4 (see the image and table R1 below). However, in the revised

version (with exclusion of the inhomogeneity rating), the strong preference for the weighting 4 is not as obvious. Nevertheless, the correlations alone are not a clear indicator. They are varying by tissue class, the segmentation, the combined ratings, as well as the dataset. We have focused here on the MR-ART dataset; however, many other datasets are available, but could not be included in the article. Therefore, we here provided the evidence in this dataset, while taking into account further tests that were run with datasets that were not included in this manuscript. Below is the figure representing the differentiation between groups of scans with different degrees of motion artifacts, with higher power weightings differentiating the groups better. Nevertheless, too high weightings became too sensitive for the worst feature.
As stated before, advanced users have the option to use the individual measures and get their own impression of the data. We here wanted to provide a simple human readable composite score to assess the quality of a scan. Below is the figure representing the differentiation between groups of scans with different degrees of motion artifacts, with higher power weightings differentiating the groups better.

To sum up:
“To quantify the effect of interferences, we estimated the volume difference (ΔV) and the Kappa value to the artifact-free case (averaged over all tissue classes), where volume changes and Kappa statistics should be highly associated with the quality rating (Table 2). We finally selected the power 4 function as it is more sensitive to outliers.”

Table R1: Results from version 201901

	Vol-BWP-avg	Vol-MRART-avg	Kappa-BWP-avg	Kappa-MRART-avg	avg
median	0.45575	0.63246	0.75774	0.89138	0.68434
mean	0.45575	0.63246	0.75774	0.89138	0.68434
rms^2	0.46668	0.63406	0.77304	0.89229	0.69152
rms^4	0.48033	0.63088	0.78391	0.88453	0.69491
rms^8	0.49132	0.62275	0.78591	0.86738	0.69184
rms^{16}	0.49507	0.61576	0.78415	0.85265	0.68691
max	0.49616	0.6112	0.78241	0.84319	0.68324

"Sample Normalization for Outlier Detection" It is unclear whether this is systematically applied or not. Is it a separate measure, or is it aggregated into another score? That measure could be relevant in many cases but could also be really bad in some specific cases (for example, historical data where the "ideal" quality would probably be well below standards.
Response: We agree that this section could be described better and have introduced changes to the text to make it clearer (see pp. 9). The sample normalization results in another sample-specific rating (NSIQR - normalised SIQR) used to test how well the outliers (e.g. in the MR-ART dataset) can be identified. The rating (as described in the outlier detection) is part of the “Check Sample Homogeneity Tool” for sample evaluation before statistical analysis, and is also stored in the XML files for each subject with an extra field that saves basic information about the data (path, datetime, number).
Overall, our measures are defined by the typical ranges that structural MRI currently supports and that could potentially be used for tissue segmentation and surface reconstruction. As the BWP can be seen as a historical element, the principle definitions of noise, inhomogeneity and resolution still fit to today's structural data. We have tested here with a broad variability of data available from larger and smaller projects. However, the challenging part are ultra-high (7 Tesla) resolution images, where protocols are still in development and some public datasets suffer from interferences (e.g. extreme bias) that will probably play only a minor role in future standard acquisitions.

"raw (co-registered)" Well, it is not raw if it's co-registered. I suggest reformulation to avoid confusion with actual raw images.
Response: Thank you for pointing out this issue, we have omitted the wording in the revised manuscript. Although raw data (defined as the output of the Dicom conversion) present the default, researchers often use the term “raw”

even if it includes preprocessing (e.g., resliced/resampled, defacing, skull-stripping, denoised, bias-corrected, or intensity normalized data) in different contexts.

The "Evaluation Concept and Data" section is very unclear. The need for a training-testing scheme is not explained, and the scheme itself is very arbitrary (choosing odd and even numbered files ordered by filenames). How does that splitting strategy help with generalisation? Why that specific split? Why not another? How do we know that split is not biased?
Response: We agree that this description appears arbitrary and we refined this section. Briefly, the split was used to define and scale the measures on another subset of data. We opted for a simple odd-even split since the BWP parameters are included in the filenames. This means that selecting every second data point provides a balanced and comprehensive sample that includes similar, but not identical, cases.

Finally, the selection of 6 scans also seems completely arbitrary. Overall, this section does not provide enough information to justify the seemingly arbitrary choices.
Response: Thanks for pointing this out. The dataset used for this analysis is highly diverse with regards to different scanning parameters and was not acquired by us. Six scans with different scan times, resolution, and parallel imaging, but similar with regard to other imaging parameters (TR/RE times) were selected only to illustrate the working of the QC on an example. We have added our selection criteria for the scans in the description of the samples.

"Of note, obvious subject/scan-specific motion artifacts generally increase the scans' rating for about 1 grade, which corresponds to a decrease of 10 rps (and +0.5 grade / -5 rps for light artifacts), in comparison to the typical rating achieved by the majority of scans of the same protocol." This is incredibly vague! How are readers supposed to evaluate the quality control measures with this information?
Response: We agree that from this statement, the users cannot evaluate the quality measures, nevertheless, we have tried to provide evidence in our manuscript to showcase the validity and reliability of the measures. The readers can rely on the numerical output of the toolbox to decide about inclusion or exclusion of the scan. The above statement was meant as a general observation and not as evidence, as we currently have no measure or classification for motion artifacts.

Discussion:
"as this is more relevant for segmentation and surface reconstruction
(Ashburner et al., 2005)." A lot of work has been done in these domains in 20 years; this reference, however solid, is not enough to justify that choice. This might not be relevant with the methods developed in the last 20 years.
Response: Thank you for this observation. We updated the references in this part of the manuscript:
“Moreover, the proposed intensity-based measures are normalised by (minimum) tissue contrast rather than signal intensity, as the separation between brain tissues, especially the GM and WM, is essential for segmentation and surface reconstruction (Fischl et al. 2012, Gaser et al., 2025).“

"with a power of 4 rather than 2, to place greater emphasis on the more problematic aspects of image quality." Still not enough to justify that choice. The authors failed to convince me that one single score is better than reporting all the measures significantly, as different quality measures will influence different tasks. A very practical example is the fact that the vast majority of acquisitions in clinical settings, the resolution is anisotropic (though less with T1 images nowadays, historical datasets will still have it). This anisotropy is not necessarily an issue for human diagnosis, for example; however, aggregating all the scores in one might hide that a low-quality measurement might not affect the specific downstream task. Coupled with the lack of justification for the factor scalings, this choice of a single score is a significant negative point for the tool.
Response: Indeed, we agree that for particular use cases, the specific (raw/unscaled) measures are preferable and can also be used. We do not object to that and provide the unscaled measures as an output in xml files. However, the composite measure was designed to meet the needs of the non-advanced users of structural preprocessing tools (for T1w-based segmentation and surface reconstruction) to get a simple estimate of the quality of their data to identify critical cases. We extended the discussion:
“Nevertheless, raw quality measures are also available in the XML files, allowing advanced users to perform detailed inspections.” (p.24)
With regard to the lack of justification for scaling, we have addressed this in the Averaging section in the Method

part.

Data availability:
Where can the sources of these specific tools be accessed?
Response: The raw data is available from the original sources, whereas processed data is available from the Gigascience server. We improved the data and code availability report in the revised manuscript.

Reviewer #2:
Technical Note GIGA-D-25-00085 introduces a segmentation-based quality control (QC) framework for T1-weighted structural MRI integrated into the CAT12 toolbox. The approach defines five interpretable image quality metrics—noise-to-contrast ratio (NCR), inhomogeneity-to-contrast ratio (ICR), resolution score (RES), edge-to-contrast ratio (ECR), and full-brain Euler characteristic (FEC)—which are combined into a composite Structural Image Quality Rating (SIQR). The tool aims to provide a standardized, interpretable scoring system for identifying poor-quality scans, with validation across simulated datasets and real-world imaging data.
Strengths
The manuscript addresses a critical need in neuroimaging by presenting an automated, interpretable, and practical framework for quality control of T1-weighted structural MRI. By integrating multiple segmentation-derived metrics into a single Structural Image Quality Rating (SIQR), the approach enables fast, standardized assessment of image quality. The tool is embedded in the widely used CAT12/SPM ecosystem, facilitating adoption, and it is validated across a range of synthetic and real-world datasets. The scoring system is designed with user accessibility in mind, offering a clear grading scale and robust detection of motion-related artifacts, making it particularly well-suited for use in large-scale research and clinical imaging settings.

Weaknesses

1. **Ambiguity of scope and segmentation dependency.**
A fundamental issue with the manuscript is its failure to clearly define the proposed QC framework's intended scope. If it is intended as a general-purpose image quality assessment tool, then several limitations become critical: its reliance on accurate tissue segmentation (1), its omission of background signal (2), its restricted validation within the CAT12 pipeline (3), and its lack of demonstrated interoperability with other workflows or populations. The method's reliability across different segmentation tools (e.g., FreeSurfer, FSL, SynthSeg) or in anatomically atypical populations (e.g., pediatric, lesioned brains) is untested (4). Conversely, if the framework is intended as a CAT12-specific internal QC tool, then the presentation is misleading. The inclusion of cross-tool benchmarks (e.g., MRIQC) (5), the use of generalized grading schemes, and the claims of robustness (6) give the impression of broader applicability. In this narrower interpretation, some concerns (e.g., pipeline generalization) would be less pressing, but others—such as the MRIQC comparison—become more problematic and unjustified. The manuscript would benefit greatly from explicitly stating whether the goal is a broadly applicable QC solution or a targeted add-on for CAT12 workflows.

Response: We appreciate your constructive feedback. In the revised version, we tried to implement the points that you raised as much as we could. The scope of our manuscript was not to present the tool as a general-purpose image quality assessment tool and we have hopefully made this clearer in the revised version. We outline early on that the tool is useful only for evaluation of structural T1-weighted images used for tissue segmentation and surface reconstruction within the SPM/CAT12 ecosystem. We address your other concerns below:
Reliance on accurate tissue segmentation
We see the potential disadvantage of relying on tissue segmentation. We acknowledge that the segmentation can fail, and we have simulated extreme cases of segmentation problems to test their effect on the quality measures (results available in the Supplementary material). These perturbances affect the variation in the quality measures; however, this variation is generally smaller than that caused by typical (light) motion artifacts. We have mentioned the potential problems with segmentation also in the Discussion, where we pointed out that severe segmentation problems can be identified by using covariance analysis of the segmentation.
("Multivariate outlier detection schemes that are typically applied based on the processed data of a sample in the normalized feature space, using similarity analysis of normalized GM data (e.g. the Gram matrix or kernels) in CAT12 (see Software section), can be used to detect outliers with preprocessing problems or highly deviating anatomy. However, the proposed image quality assessments are specifically designed to measure differences of image quality (in native space) rather than segmentation accuracy (in normalized space) or anatomical properties,

such as stroke lesions, and can therefore be used in addition to previously mentioned outlier detection schemes to identify cases where image artifacts could bias analysis." p.21)

Omission of background
We agree that the omission of background does not allow our QC tool to find certain artifacts that are prominent in the background. However, the main purpose of our QC tool is to determine the usefulness of the image for the prospective brain analysis. If the artifacts such as ghosting and wrap-around affect large areas of brain tissue, they are accounted for in the quality measure, but not if they are only in the background as this is of no use to the final analysis. As we mentioned in Discussion: "For instance, in cases when locally limited or mild artefacts affect regions that are not relevant to the study (e.g., if the study focuses on frontal regions, cerebellar artefacts from jaw movements are acceptable) or whenever lower preprocessing accuracy is acceptable (e.g., for local alignment of brain surfaces or atlases for other modalities)."

Restricted validation to CAT12
Although the analysis was focused on CAT12, we also presented the results in SPM segmentation (Figure 7) as both of these tools come from a similar ecosystem. Since our scope was not to provide an overarching image quality control tool, we did not focus on comparison with segmentation from other tools. We specified a scope in the revised manuscript.

Test in other populations and lesioned brains
The test in lesioned brain was performed in the ATLAS database (Figure 6B). We also tested the toolbox internally on data of children, however, we did not present the results in the current manuscript. We added the analysis on children in the revised version (Figure 6C & D) and used the children sample to better explain our outlier detection scheme.

Inclusion of comparison to MRIQC
We acknowledge that the comparison to the MRIQC might have appeared unfair and that was not our intention (we expand upon this below at #4). We have corrected this section in the manuscript.

Claims about generalized grading schemes, robustness
We believe that the claims about the usefulness and robustness still hold within the limited scope of the SPM/CAT framework and hope the revised manuscript properly addresses your concerns.

2. **Lack of compliance with GigaScience reproducibility standards.**
The manuscript does not currently meet GigaScience's data and code availability requirements. The code used to generate results and figures is not publicly accessible—only available upon request—which directly conflicts with the journal's expectations for open, reproducible research. Similarly, while the data are drawn from public sources, the manuscript lacks direct links, accession numbers, or DOIs for the datasets used, and provides no clarity on data preprocessing or analysis scripts. There is also no reference to licensing for the CAT12 toolbox or the code used in the study, and no reproducibility capsule (e.g., containerized environment, workflow script) is offered. These omissions limit the transparency and reusability of the work and must be addressed to comply with the FAIR principles and GigaScience's editorial policies.
Response: The code and data are available, but the Gigascience FTP was set up during the submission process and were therefore not included in the main text. We improved this in the revised version.

3. **Mischaracterization of background-based IQMs**.
In the "SIQR measure development" section, the manuscript states: "Image quality measures are commonly estimated from the image background (Mortamed et al., 2008; Esteban et al., 2017)." This statement is factually incorrect and conceptually misleading. First, the citation is incorrect—Mortamed should be Mortamet (2009). Second, it misrepresents tools like MRIQC, where most quality metrics are computed within brain tissue, including CJV, SNR, and contrast-based measures. Third, the authors entirely omit recent work (e.g., Pizarro et al., 2016; Provins et al., 2025\) showing that artifacts such as ghosting, wrap-around, and motion often manifest more clearly in the background, due to the nature of Fourier reconstruction. By excluding background regions, the proposed method may miss artifacts that are visible but lie outside the segmented brain, and the trade-offs of this design decision are not discussed. The rationale based on defacing is only partial: defacing typically removes the face, not the broader background, where artifact signals often dominate. The statement as written oversimplifies QC practices

and signals a bias toward justifying the framework's internal constraints rather than engaging with the full methodological landscape.

References:
Provins, C., … Esteban, O. (2025). Removing facial features from structural MRI images biases visual quality assessment PLOS Biology. doi:[10.1371/journal.pbio.3003149](https://journals.plos.org/plosbiology/article?id=10.1371/journal.pbio.3003149) (OA).
Pizarro RA, et al. (2016). Automated quality assessment of structural magnetic resonance brain images based on a supervised machine learning algorithm. Front Neuroinf. 10\. doi:[10.3389/fninf.2016.00052](https://doi.org/10.3389/fninf.2016.00052).
Response: Thank you for the correction, we have updated the statement in the revised manuscript. Nevertheless, we would argue that even if the artifacts that you mention are more clearly visible in the background, if they affect the brain, they are also visible in the brain tissue segmentation and our measures account for that. As we have shown in the revised version of the manuscript, the SIQR measure is highly associated with the summary statistics of the background from MRIQC. Moreover, it is not only defacing, but also other varying properties of the available structural images that limit the extraction of quality measures from the background (e.g., different backgrounds, for instance in MP2RAGE protocols, skull-stripped data etc.).
4. **Underdeveloped and opaque benchmarking against MRIQC.**
The benchmarking against MRIQC is reported only in the Results section, with no corresponding description in the Methods. It is surprising that MRIQC is not mentioned by name until page 14, despite the Esteban et al. (2017) reference appearing earlier in a different context. This suggests that the treatment of MRIQC—a widely adopted, general-purpose QC tool—has not been as thorough or fair as would be desirable. Key methodological details are missing: the authors do not explain how MRIQC was executed, how specific features (e.g., snr_wm, cjv) were selected, or whether a multivariate classifier was considered. Given that MRIQC's full model leverages multiple features simultaneously, limiting the comparison to univariate metrics weakens the validity of the claim that SIQR outperforms existing approaches. A more balanced, transparent benchmarking setup would strengthen the manuscript considerably. This benchmarking also mentions an "SPM12-based" QC performance but does not clarify how and why this comparison is made.
Response: Thank you for pointing this out. Perhaps this became unclear during the analysis and writing-up process, but as we did not intend to provide an alternative to MRIQC in the form of a general-purpose tool, the comparison was not included in the Method section. The analysis was primarily intended to test the performance of our measures in the MR-ART dataset with the available expert ratings. However, we noticed that the MRIQC measures were also available and additionally assessed the validity against the measures from a related tool. We agree that the initial version of the manuscript presented an unfair and underreported comparison, so we have updated and rephrased the relevant section.
Regarding the use of SPM, we included it to check if the measure is useful also for another input segmentation, where we selected the Unified Segmentation from SPM as it is directly available in the same ecosystem. This allowed us to test the expected atrophy in case of motion also for another tool.

5. **No analysis of failure cases.**
The manuscript does not present examples of false positives or false negatives—cases where SIQR fails to align with visual inspection or known ground truth. Without understanding when and why the metric fails, users cannot judge the risk of misclassification or apply it conservatively in sensitive datasets.
Response: Thank you for this suggestion. We have included this part in the Supplement.

Minor Issues
* Figure 7 could benefit from clearer annotation of thresholds and misclassified cases to help interpret the ROC curves.
Response: Thank you for your comment. We have tried to improve the readability of the figure.

* While the title *"The Good, the Bad, and the Ugly"* is a play on the classic western film, this informal or humorous reference may be perceived as inappropriate in a scientific context—especially for a methods paper intended to support standardization and reproducibility. The title does not convey the technical scope or scientific contribution of the work, which may undermine its visibility and perceived rigor. A more descriptive and neutral title—e.g., "Segmentation-Based Quality Control of Structural MRI using the CAT12 Toolbox"—would better reflect the content and purpose of the manuscript.

Response: We appreciate this helpful comment. We changed the title to better represent the content of the paper.

* While the authors validate their approach against synthetic degradations and segmentation-derived kappa scores, they do not sufficiently leverage human expert QC ratings. Greater engagement with visual QC standards would make the case for SIQR's practical value more compelling.
Response: Expert ratings from MR-ART were used and we have stated this more clearly in the revised manuscript.

I was given access to the supporting data but chose not to proceed with reproducibility checks at this stage, as the manuscript does not currently meet GigaScience's basic standards for code and data transparency. I look forward to reviewing a revised version that clearly defines the scope of the method, improves methodological transparency, and brings the manuscript into compliance with the journal's reproducibility and FAIR data principles.

Best regards,

Oscar Esteban, Ph. D.
Research and Teaching FNS Fellow
Dept. of Radiology, CHUV, University of Lausanne

Reviewer #3:
The paper describes an alternative way to QC T1w images with 2 major innovations: a different set of metrics not relying on background and a global score that combines those metrics. In addition, all of this is integrated in a well maintained toolbox allowing easy usage.

I only have suggestions (ie it does not have to be all done) as the overall paper is well written, easy to follow and analyses well conducted.
Thank you very much for your positive feedback. We have included your suggestions in the Supplement.

P6 NCR: it can be nice to demonstrate how it performs compared to traditional CNR (mean of the white matter intensity values minus the mean of the gray matter intensity values divided by the standard deviation of the values outside the brain) -- differs markedly because of background difference for sure, since you have plenty of test images you could show that more clearly (later in the method, based on what criteria/reason 'local' is defined as 5*5*5?)
Response: Thank you for your suggestion. We have added the comparison of CNR/NCR to the Kappa statistics to the Methods section (Figure 10).

P7 ECR should capture something similar to Entropy Focus Criterion, would be nice to provide a direct comparison
Response: Indeed, the measures are associated (rho = -0.5292) but the ICR was even a bit higher correlated (rho = -0.6852). We added the correlation matrix to the Supplement (Figure S6).

P8 typo, you meant equation 2
Response: Thank you for pointing it out. We have corrected the typo.

P8 SIQR I'm guessing you have experimented with the power function - maybe a side note to share your experience of why or how it works better than eg square
Response: We added the evaluation and further description to the supplement (Figure S1).

Dr Cyril Pernet

Reviewer #4:
Reproducibility report for: The Good, the Bad, and the Ugly: Segmentation-Based Quality Control of Structural Magnetic Resonance Images

Journal: GigaScience
ID number/DOI: GIGA-D-25-00085
Reviewer(s): Laura Caquelin, Department of Clinical Neuroscience, Karolinska Institutet, Sweden [Worked on reproducing the results and wrote the report]
Tobias Wängberg, Department of Clinical Neuroscience, Karolinska Institutet, Sweden [Worked on reproducing the results]

----

1. Summary of the Study
The study addresses how variability in magnetic resonance images quality, especially from motion artifacts or scanner differences, can affect structural image analysis. It proposes a quality assessment framework for T1-weighted images based on tissue classification and standardized image quality measures. The method is shown to be robust across datasets and conditions, helping to detect outliers and control for motion-related artifacts.

----

2. Scope of reproducibility

According to our assessment the primary objective is: to develop and validate a standardized framework for assessing the quality of structural (T1-weighted) MRI images, enabling the detection of artifacts on simulated data.

- Outcome: Quantitative quality ratings derived from image properties such as noise-to-contrast ratio (NCR), inhomogeneity-to-contrast ratio (ICR), resolution score (RES), and edge-to-contrast ratio (ECR) and Full-brain Euler characteristic (FEC) combined into a Structural Image Quality Rating (SIQR).

- Analysis method outcome: Not precised in the manuscript, but with the Matlab script we identified that the quality scores were correlated using Spearman's rank correlation, and statistical significance was assessed using p-values computed using MATLAB's built-in method.

- Main result: Results are presented in Figure 5. "The evaluation on the BWP test dataset showed that most quality ratings have a very high correlation (rho > .950, p < .001) with their corresponding perturbation and a very low correlation (rho < |0.1|) with the other tested perturbations (see table in Figure 5A & C). This suggests considerable specificity of the proposed quality measures. The combined SIQR score also showed a very strong association with the segmentation quality kappa (rho = -.913, p < .001) and brain tissue volumes (rhoCSF/GM/WM = -.472/-.484/.736, pCSF/GM/WM < .001) (Figure 5B). […]
The edge-based resolution measure ECR, on the other hand, generally performed better (rho = .828, p < .001), but was more affected by noise (rho = .306, p < .001) and inhomogeneity (rho = .223, p < .001) than other scores."

----

3. Availability of Materials
a. Data
- Data availability: Open
- Data completeness: Complete, all data necessary to reproduce main results are available
- Access Method: Private journal dropbox but also available on Github repository
- Repository: https://github.com/ChristianGaser/cat12
-Data quality: Structured
b. Code
- Code availability: Share in the private journal dropbox but also open
- Programming Language(s): Matlab
- Repository link: https://github.com/ChristianGaser/cat12
- License: GPL-2.0 License
- Repository status: Public
- Documentation: Readme file

----

4. Computational environment of reproduction analysis

- Operating system for reproduction: MacOS 15.5 (reviewer 1) and MacOS 15.1 (reviewer 2)
- Programming Language(s): Matlab
- Code implementation approach: Using shared code
- Version environment for reproduction: Matlab R2024b Update 6 (24.2.2923080) - Trial version

----

5. Results

5.1 Original study results
- Results 1: Figure 5 C (see screenshot)

5.2 Steps for reproduction

->Finding how to reproduce the results
- Issue 1: The methods section lacks sufficient detail regarding the statistical methodology, and the relevant information is not fully provided in the GitHub repository.
-- Resolved: A message has been sent to the authors requesting further clarification on the methodology and additional resources (scripts/data) needed to reproduce the results. The script to reproduce the results is "cat_tst_qa_bwpmaintest.m".

-> Reproduce the results using the "cat_tst_qa_bwpmaintest.m" script.
- Issue 2: To run the script "cat_tst_qa_bwpmaintest.m", the "eva_vol_calcKappa" function is missing.
-- Resolved: The script was shared and added to the Github repository.
- Issue 3: While running the script, the following error message encountered:
Assigning to 0 elements using a simple assignment statement is not supported. Consider using comma-separated list assignment.

Error in cat_tst_qa_bwpmaintest (line 481)
default.QS{find(cellfun('isempty',strfind(default.QS(:,2),'FEC'))==0),4} = [100, 850];
-- Resolved: This error stops the execution of the script. After discussion with the authors, the exact cause of the error encountered at line 480 was not directly identified. We exchanged and compared our environments at the point just before the error occurred and observed notable differences between them. Our environment is almost empty. The authors identified that the default variable is missing from our environment, even though it is referenced at line 437 by a call to the cat_stat_marks function.
We confirmed that all required dependencies were installed (including Statistics toolbox, SPM and CAT12), and that we had access to all the necessary data.
To ensure the issue was not due to user error, the code was independently executed by two reviewers. The error was consistently reproduced in both cases. About the setup, I specified to the authors:
"To summarize my setup:
* I have installed SPM, CAT, and the Statistics Toolbox.
* I downloaded all datasets from the GigaScience server.
* I also downloaded the IXI T1 data, but I've only kept the version available on the GigaScience server in my working directory. Is the version from GigaScience sufficient? I had presumed that this dataset was pre-processed and ready to use, so I ignored the time-consuming pre-processing step. Your last email seems to confirm this point."

The authors answered that:
« Yes, this is correct. However, both directories have to be combined so that the original IXI images and the processing files are included. »

In an attempt to proceed, we modified the portion of the code that triggered the error:
######
% FEC

```
FECpos = find(cellfun('isempty',strfind(default.QS(:,2),'FEC'))==0);
try
warning off;
[Q.fit.FEC, Q.fit.FECstat] = robustfit(Q.FECgt(M,1),Q.FECo(M,1));
warning on;
if ~isempty(FECpos)
default.QS{FECpos,4} = round([Q.fit.FEC(1) + Q.fit.FEC(2), Q.fit.FEC(1) + Q.fit.FEC(2) * 6], -1);
end

catch
Q.fit.FEC = [nan nan]; Q.fit.FECstat = struct('coeffcorr',nan(2,2),'p',nan(2,2));
if ~isempty(FECpos)
default.QS{FECpos,4} = [100 850];
end
end
#####
```

Following this adjustment, the end of the script "cat_tst_qa_bwpmaintest.m" ran without issue and generated output results:

Finally, the error was identified after numerous exchanges with the authors. The function "cat_stat_marks", available in the Github repository, was not shared in the FTP server. With this function added, the script runs correctly. Please note that the link to the Github repository where the software code can be found is not specified in the manuscript.

-> Compare the results reproduced and the original results
- Issue 4: Discrepancy between reproduced results, output results provided by the authors and the original results shown in Figure 5C.
-- Unresolved: We reproduced the figures and the corresponding output table using the modified "cat_tst_qa_bwpmaintest.m" script. We ran the script using the only default QC version selected in the script ("cat_vol_qa201901x"). By comparing our output with the result files shared by the authors, we were able to confirm that we had executed the correct pipeline.
However, we encountered a discrepancy: neither the generated file in our run (tst_cat_col_qa201901x_irBWPC_HC_T1_pn9_rf100pC_vx200x200x200rptable.csv) nor the corresponding file provided by the authors (outputs from BWPmain_full_202504) matched the numerical values presented in Figure 5C of the manuscript.
We contacted the authors to clarify whether the default QC version used in the script was indeed the one produce the figure. In response, they confirmed:

"All figures should show the results of this QC version although I had the plan to run a final check update after the reviewer comments (the figures are finally arranged in Adobe Illustrator)."

Therefore, although the correct version of the QC was used, the differences in the results shown in Figure 5C remain unexplained. This issue is still unresolved.
Response: Thank you for pointing this out. As mentioned in the initial communication with the editor, we estimated the values for the table from the images preprocessed with an earlier CAT12 version. Unfortunately, a part of preprocessed scans was accidentally deleted during the upload of the images to the Gigascience server, and had to be reprocessed, for which a current CAT12 version was used. The differences in the values were therefore expected (albeit not to a drastic extent), but we decided to correct the figures after the review process. We now updated the values in the figures and tables. Of note, the QC version that the reviewers used is the same as used for the manuscript.

5.3 Statistical comparison Original vs Reproduced results
- Results: Screenshot of reproduced tst_cat_vol_qa201901x_irBWPC_HC_T1_pn9_rf100pC_vx200x200x200_rptable.csv table

- Comments: Several p-values in the reproduced results appear as exactly 0 (0.00000000e+00), which is unlikely from a statistical point of view. It is possible that these values are just extremely small and were rounded down. However, this could also point a problem in the script. Further investigation would be needed to determine the cause.
Response: Thank you for this observation. The result of 0.00000000e+00 signifies a statistically significant result. Due to the smallness of the value, however, the number cannot be represented by a computer (i.e., arithmetic underflow). This is a common phenomenon in statistical programs. Because the probability of getting such or more extreme results when the null hypothesis is true cannot be 0, we reported this result as p<.0001.

- Errors detected: Values in Figure 5C do not correspond to those provided by the authors in the FTP server in the files (tst_cat_vol_qa201901x_irBWPC_HC_T1_pn9_rf100pC_vx200x200x200_rptable.csv). Multiple inconsistencies were observed, suggesting potential errors in the manuscript figure or mismatches between file versions (see file Comparison_original_rptable_vs_fig5C_data.csv for comparison).
Response: Thank you for pointing this out. As mentioned before, we estimated the values for the table from the images preprocessed with an earlier CAT12 version, but had to reprocess the images due to the accidental deletion of files during upload. A newer version of CAT12 was used for the preprocessing for the images on the server. The differences in the values were therefore expected (albeit not to a drastic extent), but we decided to correct the figures after the review process. We now updated the values in the figures and tables.
(Screenshot of Figure 5C)

(Screenshot of the original output corresponding to the Figure 5C)

- Statistical Consistency: The reproduced correlation table (tst_cat_vol_qa201901x_irBWPC_HC_T1_pn9_rf100pC_vx200x200x200_rptable.csv). differs from the original in terms of r-values and p-values.
Compared to the Figure 5C, the reproduced r-values do not all match those shown in the figure. P-values cannot be directly compared to Figure 5C, as they are represented by a color gradient without a scale or legend, making direct comparison impossible.
Response: Thank you for pointing this out - the same comment applies as above. We added the full correlation table with rho and p-values in the supplement.
----

6. Conclusion
- Summary of the computational reproducibility review
The computational reproducibility of the main result we identified for the study is partially achieved. After several technical issues related to missing functions, I was able to execute the script to reproduce values of Figure 5C ("cat_tst_qa_bwpmaintest.m") and obtain ouput results. However, discrepancies were observed when comparing the reproduced results (tst_cat_col_qa201901x_irBWPC_HC_T1_pn9_rf100pC_vx200x200x200rptable.csv) to both:
- the output file provided by the authors, and
- the original results presented in figure 5C of the manuscript.
Notably, the output file provided by the authors and the results in figure 5C do not match either, indicating potential errors or file versions mismatches.
Additionally, many p-values in the reproduced results are equal to 0, which suggests a formatting issue or a problem in the script. Figure 5C also lacks a scale, legend detail, or supplementary data to make possible to verify p-values (assuming the color gradient represents the p-values).

- Recommendations for authors
We strongly recommend the authors to:
-- Ensure all essential code and functions are included in the shared repositories. Some necessary files were not included in the FTP server provided with the paper. Although the GitHub repository (https://github.com/ChristianGaser/cat12) was shared with the journal, but it is not referenced in the manuscript, making it difficult for external users to locate.
Thank you very much for your comment. The repository is referenced already in the Introduction (p. 4, line 83) as the quality measure is connected to the CAT12 ecosystem.

-- Add detailed documentation of the statistical methods: the current manuscript lacks sufficient information regarding the statistical methodology used, at least for the purpose of the reproducibility review. Please, include

detailed explanation of statistical tests, packages and parameter settings (e.g. QC version) to improve reproducibility. Thank you, we now explicitly mentioned the use of Spearman correlation in the Method section.
-- Clarify the versioning and outputs for the figures: there is a lack of clarity regarding which specific data outputs were used to generate figure 5C. Providing metadata or links to the exact output file used would help to resolve this issue.
As noted above, the images on the server were preprocessed with another version of CAT12 as was used for writing the article. We have updated the values in the revised manuscript.

-- Provide raw numerical data behind figures: figure 5C seems to display p-values using a color gradient but no scale or legend is provided. Sharing the raw data used would allow the comparison and the reproducibility of the figure.
Thank you for your remark, we added the correlation analysis output to the Supplement.

-- Improve the clarity of execution instructions and address potential p-values issues: the issue with p-values showing up as exactly 0 in the reproduced results might be caused by differences in the environment setup, such as missing variables, different software versions, or skipped steps before running the script. Improving the instructions for setting up the environment and running the would help prevent issues and facilitate reproducibility.
Response: Thank you for your suggestion. Please check the comment on the arithmetic underflow above.

Editorial Manager®

Cookie Preference Center

We use cookies which are necessary to make our site work. We may also use additional cookies to analyze, improve and personalize our content and your digital experience. For more information, see our Cookie Policy.

You may choose not to allow some types of cookies. However, blocking some types may impact your experience of our site and the services we are able to offer. See the different category headings below to find out more or change your settings.

Allow all
Manage Consent Preferences

Strictly Necessary Cookies

Always active
These cookies are necessary for the website to function and cannot be switched off in our systems. They are usually only set in response to actions made by you which

amount to a request for services, such as setting your privacy preferences, logging in or filling in forms. You can set your browser to block or alert you about these cookies, but some parts of the site will not then work.

Functional Cookies

These cookies enable the website to provide enhanced functionality and personalisation. They may be set by us or by third party providers whose services we have added to our pages. If you do not allow these cookies then some or all of these services may not function properly.

Performance Cookies

These cookies allow us to count visits and traffic sources so we can measure and improve the performance of our site. They help us to know which pages are the most and least popular and see how visitors move around the site.

Targeting Cookies

These cookies may be set through our site by our advertising partners. They may be used by those companies to build a profile of your interests and show you relevant adverts on other sites. If you do not allow these cookies, you will experience less targeted advertising.

Cookie List

Clear

Apply Cancel

Consent Leg.Interest

Confirm my choices

Powered by onetrus
