# Supplementary material for: Segmentation-based quality control of structural MRI using the CAT12 toolbox
Source: Gigascience. 2025 Nov 29;14:giaf146. doi: 10.1093/gigascience/giaf146 (PMC12758382; doi:10.1093/gigascience/giaf146)
Supplement: giaf146_Reviewer_1_Report_Original_Submission — Chris Foulon -- 5/3/2025 [file giaf146_reviewer_1_report_original_submission.pdf]

Reviewer Report

Title: Segmentation-Based Quality Control of Structural MRI using the CAT12 Toolbox

Version: Original Submission **Date:** 5/3/2025

Reviewer name: Chris Foulon

Reviewer Comments to Author:

The article presents a valuable effort towards standardising quality control methods and their evaluation. However, too many choices seem arbitrary without sufficient justification, and too many sections are unclear. Overall, the quality of the work cannot be fully assessed in the current state of the manuscript, and major revisions are needed to correct that. There is also not enough comparison (one) with other methods and no way of evaluating whether these measures are relevant to actual downstream imaging uses. Additionally, the article's goal is highly unclear and led me to think the segmentation measures were part of the QC pipeline until I read the discussion ... Nothing until the discussion explains that the segmentation measures are used to evaluate the single SIQR score output of the QC pipeline.Comments:"All measures and tools are part of the Computational Anatomy Toolbox (CAT; https://neuro-jena.github.io//cat, Gaser et al., 2024) of the Statistical Parametric Mapping (SPM; http://www.fil.ion.ucl.ac.uk/spm, Ashburner et al. 2002) software and also available as a standaloneversion (https://neuro-jena.github.io/enigma-cat12/#standalone)." I cannot really expect everyone to avoid Matlab tools. Still, Matlab is a drag to the development of scalable tools nowadays (every system admin's nightmare is to have to try to make Matlab tools run on high-performance computing servers)."such as noise, inhomogeneities, and resolution (Figure 1B)." At this point in the article, it's a bit unclear how that works in Figure 1B."It is assessed within optimized cerebrospinal fluid (CSF) and white matter (WM) regions." Then, the NCR relies on the segmentation, right? What if the segmentation fails?Oh, most of the measures actually rely on the segmentation. Are segmentation errors accounted for in the tool? I am thinking specifically about "abnormal" brains that can be difficult for segmentation algorithms. At least at this point of the article, it's not clear."To accommodate various international rating systems, we have adopted a linear percentage and a corresponding (alpha-)numeric scaling." this doesn't match the complexity of the following explanation about the rather arbitrary range. I think a much more international and understandable rating would have been a 0 to 1 range. A 0.5 to 10.5 range is not helping users at all. As the rating is linear, I am struggling to see the added value of this choice."Although the BWP does not include the simulation of motion artifacts, these are in general comparable to an increase of noise in the BWP dataset by 2 percentage points." Maybe that should be justified with a reference? "in general" might be a bit light to justify not having a direct measure for something presented as important (motion artefacts) in the introduction and goal of the tool.I think the absence of a noise estimation in the QC ratings should be more thoroughly justified."To balance the sensitivity to different quality measures while ensuring that the necessary quality conditions are met, we apply an exponentially weighted averaging approach â€” similar to the root mean square (RMS) but using the fourth power and fourth root." Why is there no justification or references for these arbitrary choices? Why not the fifth root or tenth root? Why the square root and not an exponential or

any other function?"Sample Normalization for Outlier Detection" It is unclear whether this is systematically applied or not. Is it a separate measure, or is it aggregated into another score? That measure could be relevant in many cases but could also be really bad in some specific cases (for example, historical data where the "ideal" quality would probably be well below standards."raw (co-registered)" Well, it is not raw if it's co-registered. I suggest reformulation to avoid confusion with actual raw images.The "Evaluation Concept and Data" section is very unclear. The need for a training-testing scheme is not explained, and the scheme itself is very arbitrary (choosing odd and even numbered files ordered by filenames). How does that splitting strategy help with generalisation? Why that specific split? Why not another? How do we know that split is not biased?Finally, the selection of 6 scans also seems completely arbitrary. Overall, this section does not provide enough information to justify the seemingly arbitrary choices."Of note, obvious subject/scan-specific motion artifacts generally increase the scans' rating for about 1 grade, which corresponds to a decrease of 10 rps (and +0.5 grade / -5 rps for light artifacts), in comparison to the typical rating achieved by the majority of scans of the same protocol." This is incredibly vague! How are readers supposed to evaluate the quality control measures with this information?Discussion:"as this is more relevant for segmentation and surface reconstruction(Ashburner et al., 2005)." A lot of work has been done in these domains in 20 years; this reference, however solid, is not enough to justify that choice. This might not be relevant with the methods developed in the last 20 years."with a power of 4 rather than 2, to place greater emphasis on the more problematic aspects of image quality." Still not enough to justify that choice. The authors failed to convince me that one single score is better than reporting all the measures significantly, as different quality measures will influence different tasks. A very practical example is the fact that the vast majority of acquisitions in clinical settings, the resolution is anisotropic (though less with T1 images nowadays, historical datasets will still have it). This anisotropy is not necessarily an issue for human diagnosis, for example; however, aggregating all the scores in one might hide that a low-quality measurement might not affect the specific downstream task. Coupled with the lack of justification for the factor scalings, this choice of a single score is a significant negative point for the tool.Data availability:Where can the sources of these specific tools be accessed?

Level of Interest

Please indicate how interesting you found the manuscript: Choose an item.

Quality of Written English

Please indicate the quality of language in the manuscript: Choose an item.

Declaration of Competing Interests

Please complete a declaration of competing interests, considering the following questions:

- Have you in the past five years received reimbursements, fees, funding, or salary from an organisation that may in any way gain or lose financially from the publication of this manuscript, either now or in the future?
- Do you hold any stocks or shares in an organisation that may in any way gain or lose financially from the publication of this manuscript, either now or in the future?
- Do you hold or are you currently applying for any patents relating to the content of the manuscript?
- Have you received reimbursements, fees, funding, or salary from an organization that holds or has applied for patents relating to the content of the manuscript?
- Do you have any other financial competing interests?
- Do you have any non-financial competing interests in relation to this paper?

If you can answer no to all of the above, write 'I declare that I have no competing interests' below. If your reply is yes to any, please give details below.

I declare that I have no competing interests

I agree to the open peer review policy of the journal. I understand that my name will be included on my report to the authors and, if the manuscript is accepted for publication, my named report including any attachments I upload will be posted on the website along with the authors' responses. I agree for my report to be made available under an Open Access Creative Commons CC-BY license (http://creativecommons.org/licenses/by/4.0/). I understand that any comments which I do not wish to be included in my named report can be included as confidential comments to the editors, which will not be published.

Choose an item.

To further support our reviewers, we have joined with Publons, where you can gain additional credit to further highlight your hard work (see: https://publons.com/journal/530/gigascience). On publication of this paper, your review will be automatically added to Publons, you can then choose whether or not to claim your Publons credit. I understand this statement.

Yes Choose an item.
