# Supplementary material for: Segmentation-based quality control of structural MRI using the CAT12 toolbox
Source: Gigascience. 2025 Nov 29;14:giaf146. doi: 10.1093/gigascience/giaf146 (PMC12758382; doi:10.1093/gigascience/giaf146)
Supplement: giaf146_Reviewer_4_Report_Revision_1 — Laura Caquelin -- 9/24/2025 [file giaf146_reviewer_4_report_revision_1.pdf]

Reviewer Report

Title: Segmentation-Based Quality Control of Structural MRI using the CAT12 Toolbox

Version: Revision 1 **Date:** 9/24/2025

Reviewer name: Laura Caquelin

Reviewer Comments to Author:

Reproducibility report for: Segmentation-Based Quality Control of Structural MRI using the CAT12 Toolbox (New title)Journal: GigaScienceID number/DOI: GIGA-D-25-00085_R1Reviewer(s): Laura Caquelin, Department of Clinical Neuroscience, Karolinska Institutet, Sweden [Worked on reproducing the results and wrote the report]--1. ContextThis report corresponds to a second assessment of the computational reproducibility of the article GIGA-D-25-00085, following a revision by the authors after the first round of review. Some of the results have been modified by the authors in the revision.The scope of the computational reproducibility review is to reproduce the results presented in Figure 5c relative to the quality scores derived from image properties such as noise-to-contrast ratio (NCR), inhomogeneity-to-contrast ratio (ICR), resolution score (RES), and edge-to-contrast ratio (ECR) and Full-brain Euler characteristic (FEC) combined into a Structural Image Quality Rating (SIQR). The quality scores were correlated using Spearman's rank correlation, and statistical significance was assessed using p-values computed using MATLAB's built-in method.--2. Changes since the first reviewAuthors made some changes to the manuscript according to the comments of the first computational reproducibility review:- Figure 5, and especially Figure 5C (which corresponds to the scope of reproducibility), was modified. As specified by the authors, the original figure was generated using images preprocessed with an earlier version of CAT12. Some of these images were accidentally deleted and then reprocessed with a newer version of CAT12. The updated reprocessed images were used for the reproducibility review, which explains the difference in values compared to the original figure.- The full correlation table with rho and p-values was added to the supplement as Table S1.--3. Availability of Materialsa. Data- Data availability: Open- Data completeness: Complete, all data necessary to reproduce main results are available- Access Method: Private journal dropbox but also available on Github repository- Repository: https://github.com/ChristianGaser/cat12- Data quality: Structuredb. Code- Code availability: Share in the private journal dropbox but also open- Programming Language(s): Matlab- Repository link: https://github.com/ChristianGaser/cat12- License: GPL-2.0 License- Repository status: Public- Documentation: Readme file--4. Computational environment of reproduction analysis- Operating system for reproduction: MacOS 15.6.1- Programming Language(s): Matlab- Code implementation approach: Using shared code- Version environment for reproduction: Matlab R2025b (25.2.0.299804) - Trial version--5. Results5.1 Original study results- Results: Figure 5C was updated, along with the corresponding text in the manuscript. Additionally, Table S1 was added to the supplementary material, providing all rho and p-values.See Figure 5C screenshot.Text related to the

figure 5C: "The evaluation on the BWP test dataset showed that most quality ratings have a very high correlation (Spearman's rho > .950, p < .001) with their corresponding perturbation and a very low correlation (Spearman's rho < |0.1|) with the other tested perturbations (see table in Figure 5A & C)." (page 14).See Table S1 screenshot.5.2 Steps for reproductionAs the values in the new figure 5C and table S1 differed for some of the values obtained in the first round of review, we reproduced again the analyses to compare the reproduced and original results.-> Set up environment to run the "cat_tst_qa_bwpmaintest.m" script.--> Download spm by following instructions in the provided link: https://www.fil.ion.ucl.ac.uk/spm/docs/installation/--> Download CAT12 by following instructions in the provided link: https://neuro-jena.github.io/enigma-cat12/#standalone. Noted that the automatic link to download the file for mac doesn't work, I downloaded the latest one called "cat12_latest_R2017b_MCR_Mac.zip" here https://www.neuro.uni-jena.de/cat12/ .--> Download data and script files in the journal FTP server in the Dahnke2025_QCr1 folder.- Issue 1: All datasets were successfully downloaded from the FTP server. However, the archive Dahnke2025_BWP.zip could not be decompressed.-- Unresolved: The file was downloaded several times to check whether the issue was due to an incomplete download, but this did not resolve the problem. The file from Dahnke2025_QC was then used to run the script, although the authors indicated that Dahnke2025_QC is obsolete.-> Reproduce the results using the "cat_tst_qa_bwpmaintest.m" script on matlab- Issue 2: File paths were local and did not match the current environment-- Resolved: The paths were modified to match the local setup used for this reproducibility review.- Issue 3: Error line 79 "BWPgt/p0phantom_1.0mm_normal_cor.nii" does not exist." The BWPgt was not present in the Dahnke2025_QCr1 folder.-- Resolved: The BWPgt folder was instead download from the Dahnke2025_QC folder.- Issue 4: After around 20 minutes, error in the script "robustfit requires Statistics and Machine Learning Toolbox." The use of this tool is specified in the pipeline_instruction.txt but not in the require at the beginning of the script.-- Resolved: Statistics and Machine Learning Toolbox was downloaded.5.3 Statistical comparison Original vs Reproduced results- Results:Screenshot of reproduced tst_bwpmaintest_cat_vol_qa201901x_rptable_CAT.csv file.- Comments: The reproduced results were obtained using older versions of the BWP and BWPgt datasets, which may explain the observed differences.- Errors detected: No error was found when comparing Figure 5C with the original output.- Statistical Consistency: Some differences remain between the original and reproduced results. These inconsistencies are probably due to the versions of the BWP and BWPgt data used during reproduction.---6. Conclusion- Summary of the computational reproducibility reviewThe computational reproducibility of the main result identified in the study is partially confirmed. We recommend that the authors ensure all necessary data is available in the FTP folder, and specifically verify the BWP file.- Recommendations for authorsIn the first round of review, we noted:-- Ensure all essential code and functions are included in the shared repositories. The authors still need to address this comment, especially this point:"The GitHub repository (https://github.com/ChristianGaser/cat12) was shared with the journal, but it is not referenced in the manuscript, making it difficult for external users to locate." We acknowledge that the GitHub repository folder was shared via the FTP server, and according to the authors' response, the repository is referenced in the Introduction (p. 4, line 83).While we understand the authors' position, the GitHub link https://github.com/ChristianGaser/cat12 currently corresponds to reference [69] in the referenced manuscript. A more direct and explicit link in the main text would improve the clarity and

accessibility of the code and thus enhance the reproducibility of the paper.To enhance reproducibility, a README or instruction file specifying which script produces each figure and result would greatly help make the study easier to reproduce.-- Add detailed documentation of the statistical methods. Authors modified the manuscript to specify the statistical method used.-- Clarify the versioning and outputs for the figures. Authors modified the figure 5C and the results match the outputs.-- Provide raw numerical data behind figures. Authors provided raw data in the supplementary Table S1.-- Improve the clarity of execution instructions and address potential p-values issues. I agree with the authors' explanation that "due to the smallness of the value, the number cannot be represented by a computer." However, for some reproduced p-values that are 0, the original values are not that small (for example, around 10â�»Â^{2} or 10â�»Â^{3} for FEC). That's why I wondered if the issue might be related to something else. Analyses need to be re-run using the new BWP and BWPgt datasets.

Level of Interest

Please indicate how interesting you found the manuscript: Choose an item.

Quality of Written English

Please indicate the quality of language in the manuscript: Choose an item.

Declaration of Competing Interests

Please complete a declaration of competing interests, considering the following questions:

- Have you in the past five years received reimbursements, fees, funding, or salary from an organisation that may in any way gain or lose financially from the publication of this manuscript, either now or in the future?
- Do you hold any stocks or shares in an organisation that may in any way gain or lose financially from the publication of this manuscript, either now or in the future?
- Do you hold or are you currently applying for any patents relating to the content of the manuscript?
- Have you received reimbursements, fees, funding, or salary from an organization that holds or has applied for patents relating to the content of the manuscript?
- Do you have any other financial competing interests?
- Do you have any non-financial competing interests in relation to this paper?

If you can answer no to all of the above, write 'I declare that I have no competing interests' below. If your reply is yes to any, please give details below.

1. No 2. No 3. No 4. No 5. No 6. No

I agree to the open peer review policy of the journal. I understand that my name will be included on my report to the authors and, if the manuscript is accepted for publication, my named report including any attachments I upload will be posted on the website along with the authors' responses. I agree for my report to be made available under an Open Access Creative Commons CC-BY license (http://creativecommons.org/licenses/by/4.0/). I understand that any comments which I do not wish to be included in my named report can be included as confidential comments to the editors, which will not be published.

Choose an item.

To further support our reviewers, we have joined with Publons, where you can gain additional credit to further highlight your hard work (see: https://publons.com/journal/530/gigascience). On publication of this paper, your review will be automatically added to Publons, you can then choose whether or not to claim your Publons credit. I understand this statement.

Yes Choose an item.
