# Supplementary material for: Segmentation-based quality control of structural MRI using the CAT12 toolbox
Source: Gigascience. 2025 Nov 29;14:giaf146. doi: 10.1093/gigascience/giaf146 (PMC12758382; doi:10.1093/gigascience/giaf146)
Supplement: giaf146_Reviewer_4_Report_Revision_2 — Laura Caquelin -- 11/7/2025 [file giaf146_reviewer_4_report_revision_2.pdf]

**Reviewer Report**

**Title: Segmentation-Based Quality Control of Structural MRI using the CAT12 Toolbox**

**Version: Revision 2** **Date:** 11/7/2025

**Reviewer name: Laura Caquelin**

**Reviewer Comments to Author:**

Reproducibility report for: Segmentation-Based Quality Control of Structural MRI using the CAT12 ToolboxJournal: GigaScienceID number/DOI: GIGA-D-25-00085_R2Reviewer(s): Laura Caquelin, Department of Clinical Neuroscience, Karolinska Institutet, Sweden [Worked on reproducing the results and wrote the report]Tobias WÃ¤ngberg, Department of Clinical Neuroscience, Karolinska Institutet, Sweden[Run the code independently]-------------------------------------------------------------------------------------1. ContextThis report corresponds to a third assessment of the computational reproducibility of the article GIGA-D-25-00085, following a second revision by the authors.The scope of the computational reproducibility review is to reproduce the results presented in Figure 5c relative to the quality scores derived from image properties. The quality scores were correlated using Spearman's rank correlation, and statistical significance was assessed using p-values computed using MATLAB's built-in method.--------------------------------------------------------------------------------------2. Changes since the second reviewAuthors made some changes according to the comments of the second computational reproducibility review:- Uploading BWP and BWPgt folder in Dahnke2025_QCr1 folder share in the FileZilla.- Improving the pipeline_instructions.txt and description in the cat_tst_qa_main.m script.Authors also reprocessed the BWP analysis and obtained the same results as reported in the manuscript.--------------------------------------------------------------------------------------3. Availability of Materialsa. Data- Data availability: Open- Data completeness: Complete, all data necessary to reproduce main results are available- Access Method: Private journal dropbox but also available on Github repository- Repository: https://github.com/ChristianGaser/cat12-Data quality: Structuredb. Code- Code availability: Shared in the private journal dropbox but also open- Programming Language(s): Matlab- Repository link: https://github.com/ChristianGaser/cat12- License: GPL-2.0 License- Repository status: Public- Documentation: Readme file-------------------------------------------------------------------------------------4. Computational environment of reproduction analysisReviewer 1 (Laura):- Operating system for reproduction: MacOS 15.6.1- Programming Language(s): Matlab- Code implementation approach: Using shared code- Version environment for reproduction: Matlab R2025b (25.2.0.299804) - Trial versionReviewer 2 (Tobias):- Operating system for reproduction: MacOS 15.5- Programming Language(s): Matlab- Code implementation approach: Using shared code- Version environment for reproduction: Matlab R2025b (25.2.0.299804) - Trial version-------------------------------------------------------------------------------------5. Results5.1 Original study results- Results: Figure 5C, text and Table S1 remain the same as the one in the previous manuscript version.See Figure 5C screenshot.Text related to figure 5C: "The evaluation on the BWP test dataset showed that most quality ratings have a very high correlation (Spearman's rho > .950, p < .001) with their corresponding perturbation and a very low correlation (Spearman's rho < |0.1|) with the other tested perturbations (see table in Figure 5A &

C)." (page 14).See Table S1 screenshot.5.2 Steps for reproductionAs the last BWP data files were shared, the analyses were rerun to compare the reproduced and original results.-> Set up environment to run the "cat_tst_qa_bwpmaintest.m" script.--> Download spm by following instructions in the provided link: https://www.fil.ion.ucl.ac.uk/spm/docs/installation/--> Download CAT12 by following instructions in the provided link from Github. On the https://neuro-jena.github.io/cat/index.html#DOWNLOAD link, the catQC subfolder does not exist and then the cat_tst_qa_bwpmaintest.m is not avaible.This time, we did not use the standalone cat version.--> Download IXI T1 data from: http://biomedic.doc.ic.ac.uk/brain-development/downloads/IXI/IXI-T1.tar.--> Download data and script files in the journal FTP server in the Dahnke2025_QCr1 folder.-> Reproduce the results using the "cat_tst_qa_bwpmaintest.m" script on matlab--> According to the script instructions we download the Statistics and Machine Learning Toolbox and changed the file paths.--> The code executed successfully.As the results obtained were the same as those reproduced in the first review, we decided to call in a second reviewer to run the code independently.5.3 Statistical comparison Original vs Reproduced results- Results:See screenshot of reproduced tst_bwpmaintest_cat_vol_qa201901x_rptable_CAT.csv file by reviewer 1 (Laura).See screenshot of reproduced tst_bwpmaintest_cat_vol_qa201901x_rptable_CAT.csv file by reviewer 2 (Tobias).- Comments: The results of the two reviewers are identical.- Errors detected: -- Statistical Consistency: Some differences remain between the original and reproduced results. Mostly minor numerical errors (percentage of error of rho value < 10% and relative error of p-value < 2) that do not affect the conclusions drawn. (See script "Comparison_original_VS_reproduced_results_R2.R" for the calculation method). Only one rho value (RES/Kappa) presents a major numerical error with a difference of more than 10% (-0.1237 reported vs. -0.1579 reproduced).-------------------------------------------------------------------------------------6. ConclusionAfter three rounds of computational reproducibility review, the reproduced results still differ slightly from the original ones. Most differences are minor numerical variations that do not change the overall conclusions of the outcome. Only one correlation value (RES/Kappa) shows a major numerical difference of more than 10%. The other score correlations and their statistical significance are consistent across all reviews.Despite improvements made to the analysis scripts and documentation, the exact original results could not be fully reproduced. Therefore, we conclude that the Figure 5c and Table S1 results are not fully reproducible, with small numerical discrepancies remaining even after multiple independent reproductions. I would suggest re-implementing the analysis in a reproducible computational environment.

**Level of Interest**

Please indicate how interesting you found the manuscript: Choose an item.

**Quality of Written English**

Please indicate the quality of language in the manuscript: Choose an item.

**Declaration of Competing Interests**

Please complete a declaration of competing interests, considering the following questions:

- Have you in the past five years received reimbursements, fees, funding, or salary from an organisation that may in any way gain or lose financially from the publication of this manuscript, either now or in the future?
- Do you hold any stocks or shares in an organisation that may in any way gain or lose financially from the publication of this manuscript, either now or in the future?
- Do you hold or are you currently applying for any patents relating to the content of the manuscript?
- Have you received reimbursements, fees, funding, or salary from an organization that holds or has applied for patents relating to the content of the manuscript?
- Do you have any other financial competing interests?
- Do you have any non-financial competing interests in relation to this paper?

If you can answer no to all of the above, write 'I declare that I have no competing interests' below. If your reply is yes to any, please give details below.

1. No 2. No 3. No 4. No 5. No 6. No

I agree to the open peer review policy of the journal. I understand that my name will be included on my report to the authors and, if the manuscript is accepted for publication, my named report including any attachments I upload will be posted on the website along with the authors' responses. I agree for my report to be made available under an Open Access Creative Commons CC-BY license (http://creativecommons.org/licenses/by/4.0/). I understand that any comments which I do not wish to be included in my named report can be included as confidential comments to the editors, which will not be published.

Choose an item.

To further support our reviewers, we have joined with Publons, where you can gain additional credit to further highlight your hard work (see: https://publons.com/journal/530/gigascience). On publication of this paper, your review will be automatically added to Publons, you can then choose whether or not to claim your Publons credit. I understand this statement.

Yes Choose an item.
