# Supplementary material for: Segmentation-based quality control of structural MRI using the CAT12 toolbox
Source: Gigascience. 2025 Nov 29;14:giaf146. doi: 10.1093/gigascience/giaf146 (PMC12758382; doi:10.1093/gigascience/giaf146)
Supplement: giaf146_Revision_2_Report_Original_Submission — Oscar Esteban -- 5/5/2025 [file giaf146_revision_2_report_original_submission.pdf]

Reviewer Report

Title: Segmentation-Based Quality Control of Structural MRI using the CAT12 Toolbox

Version: Original Submission **Date:** 5/5/2025

Reviewer name: Oscar Esteban

Reviewer Comments to Author:

Technical Note GIGA-D-25-00085 introduces a segmentation-based quality control (QC) framework for T1-weighted structural MRI integrated into the CAT12 toolbox. The approach defines five interpretable image quality metricsâ€”noise-to-contrast ratio (NCR), inhomogeneity-to-contrast ratio (ICR), resolution score (RES), edge-to-contrast ratio (ECR), and full-brain Euler characteristic (FEC)â€—which are combined into a composite Structural Image Quality Rating (SIQR). The tool aims to provide a standardized, interpretable scoring system for identifying poor-quality scans, with validation across simulated datasets and real-world imaging data.## **Strengths**The manuscript addresses a critical need in neuroimaging by presenting an automated, interpretable, and practical framework for quality control of T1-weighted structural MRI. By integrating multiple segmentation-derived metrics into a single Structural Image Quality Rating (SIQR), the approach enables fast, standardized assessment of image quality. The tool is embedded in the widely used CAT12/SPM ecosystem, facilitating adoption, and it is validated across a range of synthetic and real-world datasets. The scoring system is designed with user accessibility in mind, offering a clear grading scale and robust detection of motion-related artifacts, making it particularly well-suited for use in large-scale research and clinical imaging settings.## **Weaknesses**1. **Ambiguity of scope and segmentation dependency.** A fundamental issue with the manuscript is its failure to clearly define the proposed QC framework's intended scope. If it is intended as a general-purpose image quality assessment tool, then several limitations become critical: its reliance on accurate tissue segmentation, its omission of background signal, its restricted validation within the CAT12 pipeline, and its lack of demonstrated interoperability with other workflows or populations. The method's reliability across different segmentation tools (e.g., FreeSurfer, FSL, SynthSeg) or in anatomically atypical populations (e.g., pediatric, lesioned brains) is untested. Conversely, if the framework is intended as a CAT12-specific internal QC tool, then the presentation is misleading. The inclusion of cross-tool benchmarks (e.g., MRIQC), the use of generalized grading schemes, and the claims of robustness give the impression of broader applicability. In this narrower interpretation, some concerns (e.g., pipeline generalization) would be less pressing, but othersâ€”such as the MRIQC comparisonâ€—become more problematic and unjustified. The manuscript would benefit greatly from explicitly stating whether the goal is a broadly applicable QC solution or a targeted add-on for CAT12 workflows.2. **Lack of compliance with GigaScience reproducibility standards.** The manuscript does not currently meet GigaScience's data and code availability requirements. The code used to generate results and figures is not publicly accessibleâ€—only available upon requestâ€—which directly conflicts with the journal's expectations for open, reproducible research. Similarly, while the data are drawn from public sources, the manuscript lacks direct links, accession numbers, or DOIs for the datasets used, and provides no clarity on data preprocessing or analysis scripts. There is also no reference to licensing for

the CAT12 toolbox or the code used in the study, and no reproducibility capsule (e.g., containerized environment, workflow script) is offered. These omissions limit the transparency and reusability of the work and must be addressed to comply with the FAIR principles and GigaScience's editorial policies.3. **Mischaracterization of background-based IQMs**. In the "SIQR measure development" section, the manuscript states: "Image quality measures are commonly estimated from the image background (Mortamed et al., 2008; Esteban et al., 2017)." This statement is factually incorrect and conceptually misleading. First, the citation is incorrectâ€"Mortamed should be Mortamet (2009). Second, it misrepresents tools like MRIQC, where most quality metrics are computed within brain tissue, including CJV, SNR, and contrast-based measures. Third, the authors entirely omit recent work (e.g., Pizarro et al., 2016; Provins et al., 2025\) showing that artifacts such as ghosting, wrap-around, and motion often manifest more clearly in the background, due to the nature of Fourier reconstruction. By excluding background regions, the proposed method may miss artifacts that are visible but lie outside the segmented brain, and the trade-offs of this design decision are not discussed. The rationale based on defacing is only partial: defacing typically removes the face, not the broader background, where artifact signals often dominate. The statement as written oversimplifies QC practices and signals a bias toward justifying the framework's internal constraints rather than engaging with the full methodological landscape.**References:**Provins, C., … Esteban, O. (2025). Removing facial features from structural MRI images biases visual quality assessment PLOS Biology. doi:[10.1371/journal.pbio.3003149](https://journals.plos.org/plosbiology/article?id=10.1371/journal.pbio.3003149) (OA).Pizarro RA, et al. (2016). Automated quality assessment of structural magnetic resonance brain images based on a supervised machine learning algorithm. Front Neuroinf. 10\. doi:[10.3389/fninf.2016.00052](https://doi.org/10.3389/fninf.2016.00052).4. **Underdeveloped and opaque benchmarking against MRIQC.** The benchmarking against MRIQC is reported only in the Results section, with no corresponding description in the Methods. It is surprising that MRIQC is not mentioned by name until page 14, despite the Esteban et al. (2017) reference appearing earlier in a different context. This suggests that the treatment of MRIQCâ€"a widely adopted, general-purpose QC toolâ€"has not been as thorough or fair as would be desirable. Key methodological details are missing: the authors do not explain how MRIQC was executed, how specific features (e.g., snr_wm, cjv) were selected, or whether a multivariate classifier was considered. Given that MRIQC's full model leverages multiple features simultaneously, limiting the comparison to univariate metrics weakens the validity of the claim that SIQR outperforms existing approaches. A more balanced, transparent benchmarking setup would strengthen the manuscript considerably. This benchmarking also mentions an "SPM12-based" QC performance but does not clarify how and why this comparison is made.5. **No analysis of failure cases.** The manuscript does not present examples of false positives or false negativesâ€"cases where SIQR fails to align with visual inspection or known ground truth. Without understanding when and why the metric fails, users cannot judge the risk of misclassification or apply it conservatively in sensitive datasets.# **Minor Issues*** Figure 7 could benefit from clearer annotation of thresholds and misclassified cases to help interpret the ROC curves.* While the title *"The Good, the Bad, and the Ugly"* is a play on the classic western film, this informal or humorous reference may be perceived as inappropriate in a scientific contextâ€"especially for a methods paper intended to support standardization and reproducibility. The title does not convey the technical scope or scientific contribution of the work, which may undermine its visibility and perceived rigor. A more descriptive and

neutral titleâ€”e.g., "Segmentation-Based Quality Control of Structural MRI using the CAT12 Toolbox"â€”would better reflect the content and purpose of the manuscript.* While the authors validate their approach against synthetic degradations and segmentation-derived kappa scores, they do not sufficiently leverage human expert QC ratings. Greater engagement with visual QC standards would make the case for SIQR's practical value more compelling.I was given access to the supporting data but chose not to proceed with reproducibility checks at this stage, as the manuscript does not currently meet GigaScience's basic standards for code and data transparency. I look forward to reviewing a revised version that clearly defines the scope of the method, improves methodological transparency, and brings the manuscript into compliance with the journal's reproducibility and FAIR data principles.Best regards,**Oscar Esteban, Ph. D.**Research and Teaching FNS FellowDept. of Radiology, CHUV, University of Lausanne

Level of Interest

Please indicate how interesting you found the manuscript: Choose an item.

Quality of Written English

Please indicate the quality of language in the manuscript: Choose an item.

Declaration of Competing Interests

Please complete a declaration of competing interests, considering the following questions:

- Have you in the past five years received reimbursements, fees, funding, or salary from an organisation that may in any way gain or lose financially from the publication of this manuscript, either now or in the future?
- Do you hold any stocks or shares in an organisation that may in any way gain or lose financially from the publication of this manuscript, either now or in the future?
- Do you hold or are you currently applying for any patents relating to the content of the manuscript?
- Have you received reimbursements, fees, funding, or salary from an organization that holds or has applied for patents relating to the content of the manuscript?
- Do you have any other financial competing interests?
- Do you have any non-financial competing interests in relation to this paper?

If you can answer no to all of the above, write 'I declare that I have no competing interests' below. If your reply is yes to any, please give details below.

I declare that I have no competing interests

I agree to the open peer review policy of the journal. I understand that my name will be included on my report to the authors and, if the manuscript is accepted for publication, my named report including any attachments I upload will be posted on the website along with the authors' responses. I agree for my report to be made available under an Open Access Creative Commons CC-BY license (http://creativecommons.org/licenses/by/4.0/). I understand that any comments which I do not wish to be included in my named report can be included as confidential comments to the editors, which will not be published.

Choose an item.

To further support our reviewers, we have joined with Publons, where you can gain additional credit to further highlight your hard work (see: https://publons.com/journal/530/gigascience). On publication of this paper, your review will be automatically added to Publons, you can then choose whether or not to claim your Publons credit. I understand this statement.

Yes Choose an item.
