# Supplementary material for: Segmentation-based quality control of structural MRI using the CAT12 toolbox
Source: Gigascience. 2025 Nov 29;14:giaf146. doi: 10.1093/gigascience/giaf146 (PMC12758382; doi:10.1093/gigascience/giaf146)
Supplement: giaf146_Revision_3_Report_Original_Submission — Cyril Pernet, PhD -- 5/11/2025 [file giaf146_revision_3_report_original_submission.pdf]

Reviewer Report

Title: Segmentation-Based Quality Control of Structural MRI using the CAT12 Toolbox

Version: Original Submission **Date:** 5/11/2025

Reviewer name: Cyril Pernet, PhD

Reviewer Comments to Author:

The paper describes an alternative way to QC T1w images with 2 major innovations: a different set of metrics not relying on background and a global score that combines those metrics. In addition, all of this is integrated in a well maintained toolbox allowing easy usage.I only have suggestions (ie it does not have to be all done) as the overall paper is well written, easy to follow and analyses well conducted.P6 NCR: it can be nice to demonstrate how it performs compared to traditional CNR (mean of the white matter intensity values minus the mean of the gray matter intensity values divided by the standard deviation of the values outside the brain) -- differs markedly because of background difference for sure, since you have plenty of test images you could show that more clearly (later in the method, based on what criteria/reason 'local' is defined as 5*5*5?)P7 ECR should capture something similar to Entropy Focus Criterion, would be nice to provide a direct comparisonP8 typo, you meant equation 2P8 SIQR I'm guessing you have experimented with the power function - maybe a side note to share your experience of why or how it works better than eg squareDr Cyril Pernet

Level of Interest

Please indicate how interesting you found the manuscript: Choose an item.

Quality of Written English

Please indicate the quality of language in the manuscript: Choose an item.

Declaration of Competing Interests

Please complete a declaration of competing interests, considering the following questions:

- Have you in the past five years received reimbursements, fees, funding, or salary from an organisation that may in any way gain or lose financially from the publication of this manuscript, either now or in the future?
- Do you hold any stocks or shares in an organisation that may in any way gain or lose financially from the publication of this manuscript, either now or in the future?
- Do you hold or are you currently applying for any patents relating to the content of the manuscript?
- Have you received reimbursements, fees, funding, or salary from an organization that holds or has applied for patents relating to the content of the manuscript?

- Do you have any other financial competing interests?
- Do you have any non-financial competing interests in relation to this paper?

If you can answer no to all of the above, write 'I declare that I have no competing interests' below. If your reply is yes to any, please give details below.

I declare that I have no competing interests

I agree to the open peer review policy of the journal. I understand that my name will be included on my report to the authors and, if the manuscript is accepted for publication, my named report including any attachments I upload will be posted on the website along with the authors' responses. I agree for my report to be made available under an Open Access Creative Commons CC-BY license (http://creativecommons.org/licenses/by/4.0/). I understand that any comments which I do not wish to be included in my named report can be included as confidential comments to the editors, which will not be published.

Choose an item.

To further support our reviewers, we have joined with Publons, where you can gain additional credit to further highlight your hard work (see: https://publons.com/journal/530/gigascience). On publication of this paper, your review will be automatically added to Publons, you can then choose whether or not to claim your Publons credit. I understand this statement.

Yes Choose an item.
