# Supplementary material for: Segmentation-based quality control of structural MRI using the CAT12 toolbox
Source: Gigascience. 2025 Nov 29;14:giaf146. doi: 10.1093/gigascience/giaf146 (PMC12758382; doi:10.1093/gigascience/giaf146)
Supplement: giaf146_Revision_4_Report_Original_Submission — Laura Caquelin -- 6/10/2025 [file giaf146_revision_4_report_original_submission.pdf]

Reviewer Report

Title: Segmentation-Based Quality Control of Structural MRI using the CAT12 Toolbox

Version: Original Submission **Date:** 6/10/2025

Reviewer name: Laura Caquelin

Reviewer Comments to Author:

Reproducibility report for: The Good, the Bad, and the Ugly: Segmentation-Based Quality Control of Structural Magnetic Resonance ImagesJournal: GigaScienceID number/DOI: GIGA-D-25-00085Reviewer(s): Laura Caquelin, Department of Clinical Neuroscience, Karolinska Institutet, Sweden [Worked on reproducing the results and wrote the report]Tobias WÃ¤ngberg, Department of Clinical Neuroscience, Karolinska Institutet, Sweden [Worked on reproducing the results]--1. Summary of the StudyThe study addresses how variability in magnetic resonance images quality, especially from motion artifacts or scanner differences, can affect structural image analysis. It proposes a quality assessment framework for T1-weighted images based on tissue classification and standardized image quality measures. The method is shown to be robust across datasets and conditions, helping to detect outliers and control for motion-related artifacts.--2. Scope of reproducibilityAccording to our assessment the primary objective is: to develop and validate a standardized framework for assessing the quality of structural (T1-weighted) MRI images, enabling the detection of artifacts on simulated data.- Outcome: Quantitative quality ratings derived from image properties such as noise-to-contrast ratio (NCR), inhomogeneity-to-contrast ratio (ICR), resolution score (RES), and edge-to-contrast ratio (ECR) and Full-brain Euler characteristic (FEC) combined into a Structural Image Quality Rating (SIQR).- Analysis method outcome: Not precised in the manuscript, but with the Matlab script we identified that the quality scores were correlated using Spearman's rank correlation, and statistical significance was assessed using p-values computed using MATLAB's built-in method.- Main result: Results are presented in Figure 5. "The evaluation on the BWP test dataset showed that most quality ratings have a very high correlation (rho > .950, p < .001) with their corresponding perturbation and a very low correlation (rho < |0.1|) with the other tested perturbations (see table in Figure 5A & C). This suggests considerable specificity of the proposed quality measures. The combined SIQR score also showed a very strong association with the segmentation quality kappa (rho = -.913, p < .001) and brain tissue volumes (rhoCSF/GM/WM = -.472/-.484/.736, pCSF/GM/WM < .001) (Figure 5B). […]The edge-based resolution measure ECR, on the other hand, generally performed better (rho = .828, p < .001), but was more affected by noise (rho = .306, p < .001) and inhomogeneity (rho = .223, p < .001) than other scores."--3. Availability of Materialsa. Data- Data availability: Open- Data completeness: Complete, all data necessary to reproduce main results are available- Access Method: Private journal dropbox but also available on Github repository- Repository: https://github.com/ChristianGaser/cat12-Data quality: Structuredb. Code- Code availability: Share in the private journal dropbox but also open- Programming Language(s): Matlab- Repository link: https://github.com/ChristianGaser/cat12- License: GPL-2.0 License- Repository status: Public-

Documentation: Readme file---4. Computational environment of reproduction analysis- Operating system for reproduction: MacOS 15.5 (reviewer 1) and MacOS 15.1 (reviewer 2)- Programming Language(s): Matlab- Code implementation approach: Using shared code- Version environment for reproduction: Matlab R2024b Update 6 (24.2.2923080) - Trial version---5. Results5.1 Original study results- Results 1: Figure 5 C (see screenshot)5.2 Steps for reproduction->Finding how to reproduce the results- Issue 1: The methods section lacks sufficient detail regarding the statistical methodology, and the relevant information is not fully provided in the GitHub repository.-- Resolved: A message has been sent to the authors requesting further clarification on the methodology and additional resources (scripts/data) needed to reproduce the results. The script to reproduce the results is "cat_tst_qa_bwpmaintest.m".-> Reproduce the results using the "cat_tst_qa_bwpmaintest.m" script.- Issue 2: To run the script "cat_tst_qa_bwpmaintest.m", the "eva_vol_calcKappa" function is missing.-- Resolved: The script was shared and added to the Github repository.- Issue 3: While running the script, the following error message encountered:Assigning to 0 elements using a simple assignment statement is not supported. Consider using comma-separated list assignment.Error in cat_tst_qa_bwpmaintest (line 481)default.QS{find(cellfun('isempty',strfind(default.QS(:,2),'FEC'))==0),4} = [100, 850];-- Resolved: This error stops the execution of the script. After discussion with the authors, the exact cause of the error encountered at line 480 was not directly identified. We exchanged and compared our environments at the point just before the error occurred and observed notable differences between them. Our environment is almost empty. The authors identified that the default variable is missing from our environment, even though it is referenced at line 437 by a call to the cat_stat_marks function.We confirmed that all required dependencies were installed (including Statistics toolbox, SPM and CAT12), and that we had access to all the necessary data.To ensure the issue was not due to user error, the code was independently executed by two reviewers. The error was consistently reproduced in both cases. About the setup, I specified to the authors:"To summarize my setup:* I have installed SPM, CAT, and the Statistics Toolbox.* I downloaded all datasets from the GigaScience server.* I also downloaded the IXI T1 data, but I've only kept the version available on the GigaScience server in my working directory. Is the version from GigaScience sufficient? I had presumed that this dataset was pre-processed and ready to use, so I ignored the time-consuming pre-processing step. Your last email seems to confirm this point."The authors answered that:Â« Yes, this is correct. However, both directories have to be combined so that the original IXI images and the processing files are included. Â»In an attempt to proceed, we modified the portion of the code that triggered the error:######% FECFECpos = find(cellfun('isempty',strfind(default.QS(:,2),'FEC'))==0);trywarning off;[Q.fit.FEC, Q.fit.FECstat] = robustfit(Q.FECgt(M,1),Q.FECo(M,1));warning on;if ~isempty(FECpos)default.QS{FECpos,4} = round([Q.fit.FEC(1) + Q.fit.FEC(2), Q.fit.FEC(1) + Q.fit.FEC(2) * 6], -1);endcatchQ.fit.FEC = [nan nan]; Q.fit.FECstat = struct('coeffcorr',nan(2,2),'p',nan(2,2));if ~isempty(FECpos)default.QS{FECpos,4} = [100 850];endend#####Following this adjustment, the end of the script "cat_tst_qa_bwpmaintest.m" ran without issue and generated output results:Finally, the error was identified after numerous exchanges with the authors. The function "cat_stat_marks", available in the Github repository, was not shared in the FTP server. With this function added, the script runs correctly. Please note that the link to the Github repository where the software code can be found is not specified in the manuscript.-> Compare the results reproduced and the original results- Issue 4:

Discrepancy between reproduced results, output results provided by the authors and the original results shown in Figure 5C.-- Unresolved: We reproduced the figures and the corresponding output table using the modified "cat_tst_qa_bwpmaintest.m" script. We ran the script using the only default QC version selected in the script ("cat_vol_qa201901x"). By comparing our output with the result files shared by the authors, we were able to confirm that we had executed the correct pipeline.However, we encountered a discrepancy: neither the generated file in our run (tst_cat_col_qa201901x_irBWPC_HC_T1_pn9_rf100pC_vx200x200x200rptable.csv) nor the corresponding file provided by the authors (outputs from BWPmain_full_202504) matched the numerical values presented in Figure 5C of the manuscript.We contacted the authors to clarify whether the default QC version used in the script was indeed the one produce the figure. In response, they confirmed:"All figures should show the results of this QC version although I had the plan to run a final check update after the reviewer comments (the figures are finally arranged in Adobe Illustrator)."Therefore, although the correct version of the QC was used, the differences in the results shown in Figure 5C remain unexplained. This issue is still unresolved.5.3 Statistical comparison Original vs Reproduced results- Results: Screenshot of reproducedtst_cat_vol_qa201901x_irBWPC_HC_T1_pn9_rf100pC_vx200x200x200_rptable.csv table- Comments: Several p-values in the reproduced results appear as exactly 0 (0.00000000e+00), which is unlikely from a statistical point of view. It is possible that these values are just extremely small and were rounded down. However, this could also point a problem in the script. Further investigation would be needed to determine the cause.- Errors detected: Values in Figure 5C do not correspond to those provided by the authors in the FTP server in the files (tst_cat_vol_qa201901x_irBWPC_HC_T1_pn9_rf100pC_vx200x200x200_rptable.csv). Multiple inconsistencies were observed, suggesting potential errors in the manuscript figure or mismatches between file versions (see file Comparison_original_rptable_vs_fig5C_data.csv for comparison).(Screenshot of Figure 5C)(Screenshot of the original output corresponding to the Figure 5C)- Statistical Consistency: The reproduced correlation table (tst_cat_vol_qa201901x_irBWPC_HC_T1_pn9_rf100pC_vx200x200x200_rptable.csv). differs from the original in terms of r-values and p-values.Compared to the Figure 5C, the reproduced r-values do not all match those shown in the figure. P-values cannot be directly compared to Figure 5C, as they are represented by a color gradient without a scale or legend, making direct comparison impossible.---6. Conclusion- Summary of the computational reproducibility reviewThe computational reproducibility of the main result we identified for the study is partially achieved. After several technical issues related to missing functions, I was able to execute the script to reproduce values of Figure 5C ("cat_tst_qa_bwpmaintest.m") and obtain ouput results. However, discrepancies were observed when comparing the reproduced results (tst_cat_col_qa201901x_irBWPC_HC_T1_pn9_rf100pC_vx200x200x200rptable.csv) to both:- the output file provided by the authors, and- the original results presented in figure 5C of the manuscript.Notably, the output file provided by the authors and the results in figure 5C do not match either, indicating potential errors or file versions mismatches.Additionally, many p-values in the reproduced results are equal to 0, which suggests a formatting issue or a problem in the script. Figure 5C also lacks a scale, legend detail, or supplementary data to make possible to verify p-values (assuming the color gradient represents the p-values).- Recommendations for authorsWe strongly recommend the authors to:--

Ensure all essential code and functions are included in the shared repositories. Some necessary files were not included in the FTP server provided with the paper. Although the GitHub repository (https://github.com/ChristianGaser/cat12) was shared with the journal, but it is not referenced in the manuscript, making it difficult for external users to locate.-- Add detailed documentation of the statistical methods: the current manuscript lacks sufficient information regarding the statistical methodology used, at least for the purpose of the reproducibility review. Please, include detailed explanation of statistical tests, packages and parameter settings (e.g. QC version) to improve reproducibility.-- Clarify the versioning and outputs for the figures: there is a lack of clarity regarding which specific data outputs were used to generate figure 5C. Providing metadata or links to the exact output file used would help to resolve this issue.-- Provide raw numerical data behind figures: figure 5C seems to display p-values using a color gradient but no scale or legend is provided. Sharing the raw data used would allow the comparison and the reproducibility of the figure.-- Improve the clarity of execution instructions and address potential p-values issues: the issue with p-values showing up as exactly 0 in the reproduced results might be caused by differences in the environment setup, such as missing variables, different software versions, or skipped steps before running the script. Improving the instructions for setting up the environment and running the would help prevent issues and facilitate reproducibility.

Level of Interest

Please indicate how interesting you found the manuscript: Choose an item.

Quality of Written English

Please indicate the quality of language in the manuscript: Choose an item.

Declaration of Competing Interests

Please complete a declaration of competing interests, considering the following questions:

- Have you in the past five years received reimbursements, fees, funding, or salary from an organisation that may in any way gain or lose financially from the publication of this manuscript, either now or in the future?
- Do you hold any stocks or shares in an organisation that may in any way gain or lose financially from the publication of this manuscript, either now or in the future?
- Do you hold or are you currently applying for any patents relating to the content of the manuscript?
- Have you received reimbursements, fees, funding, or salary from an organization that holds or has applied for patents relating to the content of the manuscript?
- Do you have any other financial competing interests?
- Do you have any non-financial competing interests in relation to this paper?

If you can answer no to all of the above, write 'I declare that I have no competing interests' below. If your reply is yes to any, please give details below.

1.No 2.No 3.No 4.No 5.No 6.No

I agree to the open peer review policy of the journal. I understand that my name will be included on my report to the authors and, if the manuscript is accepted for publication, my named report including any attachments I upload will be posted on the website along with the authors' responses. I agree for my report to be made available under an Open Access Creative Commons CC-BY license (http://creativecommons.org/licenses/by/4.0/). I understand that any comments which I do not wish to be included in my named report can be included as confidential comments to the editors, which will not be published.

Choose an item.

To further support our reviewers, we have joined with Publons, where you can gain additional credit to further highlight your hard work (see: https://publons.com/journal/530/gigascience). On publication of this paper, your review will be automatically added to Publons, you can then choose whether or not to claim your Publons credit. I understand this statement.

Yes Choose an item.
